# Copper-Catalyzed 1,2-Diazidation and 1,2-Azidooxygenation of 1,3-Dienes: Three Divergent Protocols Using Zhdankin’s Reagent

**DOI:** 10.1021/acs.joc.5c02179

**Published:** 2025-12-11

**Authors:** Adriana E. Barni, Megan A. George, Jacob R. Pangborn, Brett N. Hemric

**Affiliations:** Department of Chemistry and Biochemistry, 7832 University of Tampa, Tampa, Florida 33606, United States

## Abstract

A series of three divergent protocols for the copper-catalyzed azidation of 1,3-dienes using iodine­(III) Zhdankin’s reagent is reported. This strategy features protocols for 1,2-diazidation, two-component 1,2-azidooxygenation, and three-component 1,2-azidooxygenation of 1,3-dienes in highly chemo-, regio-, and site-selective fashion under mild conditions. Excellent to fair yields are reported for a variety of activated and unactivated 1,3-diene systems with a range of substitution patterns. Additionally, mechanistic investigations are included to provide insights into the underpinnings for the divergence of these protocols and the component requirements.

## Graphical abstract

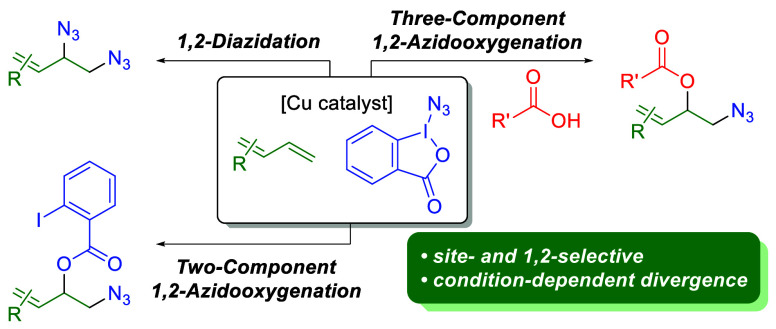

## Introduction

Azides offer a versatile synthetic handle for the incorporation of nitrogen/amino synthons into molecular scaffolds,^1^ as well as for use in bioorthogonal reactions,^2^ such as the Staudinger ligation^3^ and azide–alkyne “click” reactions.^4^ As such, rapid and robust methods for the installation of azides into organic molecules are in high demand. The addition of azides to olefins provides a potentially advantageous route for the installation of azide groups due to the ability to install two functional groups in a single, expeditious step. From this, it is unsurprising that the addition of azides to alkenes has seen significant interest and development. There has been a plethora of creative strategies developed for vicinal azido-functionalization of alkenes,^5^ such as 1,2-diazidation,^6^ 1,2-azidoamination,^6e,7^ 1,2-azidooxygenation,^6a,6b,6e,8^ 1,2-azidohydroxylation,^9^ 1,2-azidoalkynylation,^10^ 1,2-azidocyanation,^11^ 1,2-azidoarylation,^12^ and 1,2-azidohalogenation^13^ (Scheme 1A). Despite the significant development of vicinal alkene azidations, the addition of azides to conjugated 1,3-dienes remains sparsely investigated, with only a few 1,3-dienes being included in the substrate scope for select alkene azidation reactions.^6c,6d,9a,14^ Much of the reason for this deficiency can be found in the inherent selectivity challenges in the 1,2-difunctionalization of 1,3-dienes (Scheme 1B). In addition to possessing two distinct alkene addition sites for addition of the electrophile, 1,3-dienes demonstrate resonance of cation/radical intermediates, allowing the formation of both 1,2- and 1,4-addition products. This high number of possible reaction outcomes often leads to complex product mixtures and unselective reactions.

**Scheme 1:**
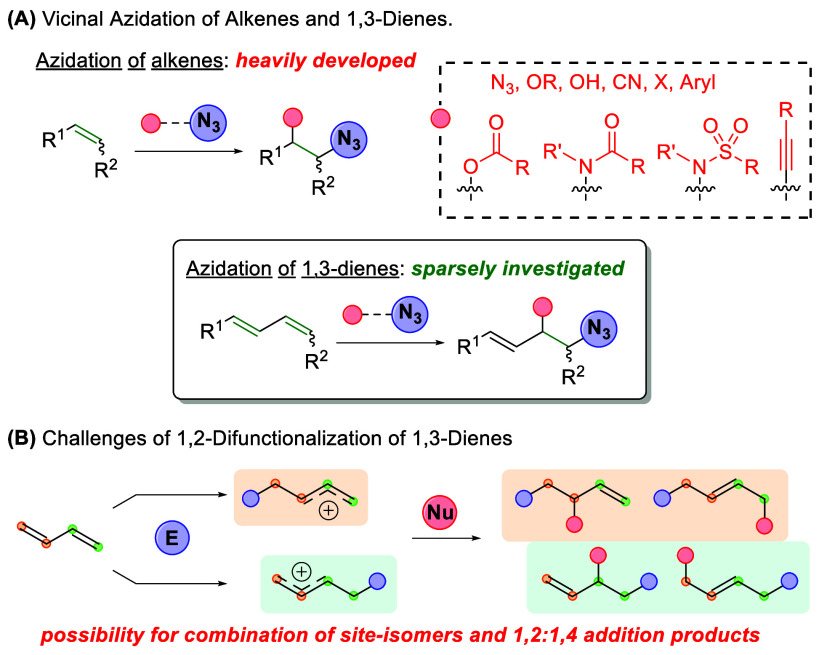
Vicinal Azidation of Olefins and Challenges with Vicinal Difunctionalization of 1,3-Dienes

Hypervalent iodine­(III) reagents, such as iodoxolones and iodoxolanes, are valuable reagents for the electrophilic functionalization of carbon scaffolds due to their ease of preparation, significant reactivity, compatibility with various transition metals, and range of possible functional group variations.^15^ Although the azide variant of this iodine­(III) iodoxolone scaffold, Zhdankin’s reagent,^16^ has seen considerable use in the azidation of alkenes,^5f,6a,6b,6e,7a–7c,8a,8c,12b^ there has yet to be any study devoted to its application for the azidation of 1,3-dienes. To the best of our knowledge, iodine­(III) iodoxolones of any substitution have been utilized with 1,3-dienes in only two studies. First, Togni’s reagent was utilized with a copper catalyst to provide (trifluoromethyl)­oxygenation in excellent yields, with the 1,2- or 1,4- selectivity dependent upon the 1,3-diene utilized (Scheme 2A).^17^ Second, iodine­(III) alkyne reagents were combined with indoles, a palladium catalyst, and indium to provide 1,2-alkynylindolation in fair yields (Scheme 2B).^18^ Given our lab’s interest in 1,3-diene reactivity and the notable lack of examples for the use of iodoxolones in 1,3-diene functionalization, we sought to further investigate this reaction platform. It was hypothesized that Zhdankin’s reagent could serve as an active iodine­(III) iodoxolone reagent for the addition to 1,3-dienes while simultaneously incorporating a valuable azido functional group handle.

**Scheme 2:**
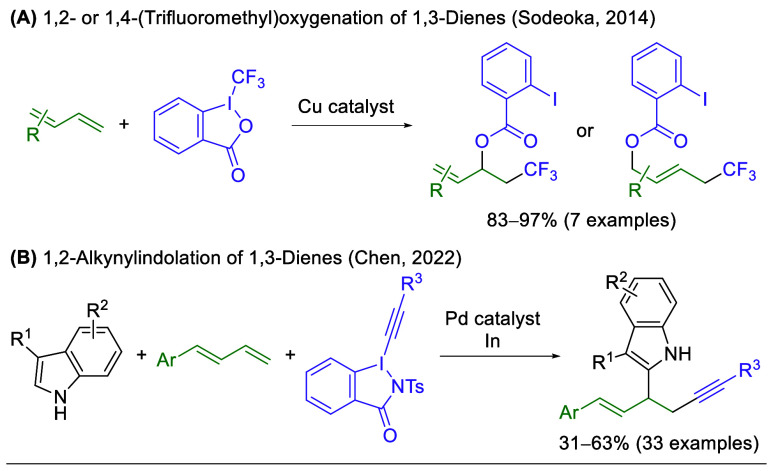
Prior Examples of Iodine­(III) Iodoxolones in 1,3-Diene Difunctionalization

Notably, while this work was being undertaken, several recent reports were published that studied the addition of azides onto 1,3-dienes in both 1,2-azidooxygenation and 1,2-diazidation protocols. Chen and co-workers developed a photocatalyzed system using an iodine­(III) azide reagent and a chiral photocatalyst to accomplish an asymmetric 1,2-azidooxygenation (Scheme 3A).^19^ Very recently, nearly simultaneous reports from both the Song and Xu groups disclosed protocols for 1,2-diazidation of 1,3-dienes using either copper or cobalt catalysis with trimethylsilylazide and an exogenous oxidant (Scheme 3B).^20^ Herein, this work describes the azidation of 1,3-dienes using Zhdankin’s reagent in three distinct chemo-, regio-, site-, and 1,2:1,4 selective protocols for the 1,2-diazidation, two-component 1,2-azidooxygenation, and three-component 1,2-azidooxygenation of 1,3-dienes using Zhdankin’s reagent (Scheme 3C).

**Scheme 3:**
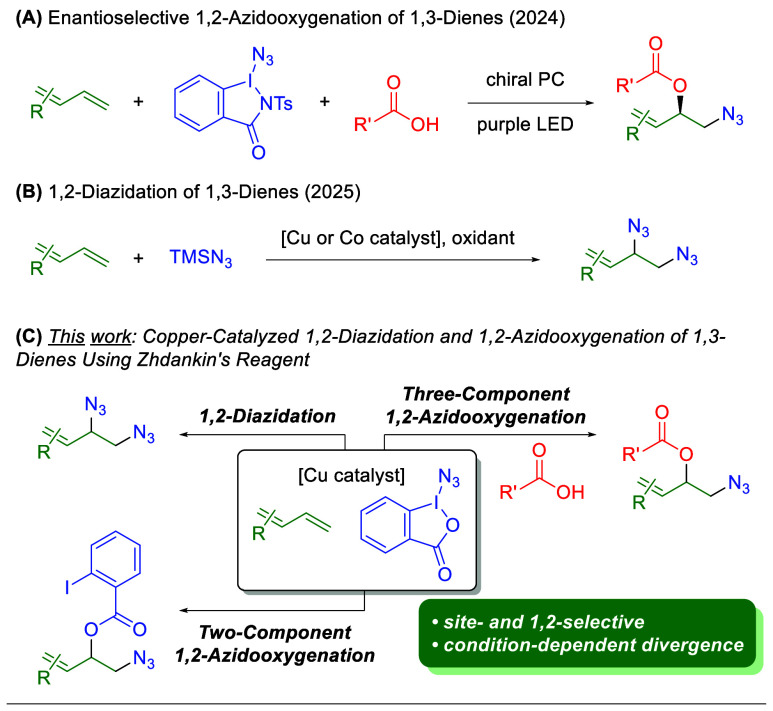
Recent Efforts on Azidation of 1,3-Dienes

## Results and Discussion

Optimization began by combining 1-phenyl-1,3-butadiene (**1a**) and the azido iodine­(III) iodoxolone (Zhdankin’s reagent, **2**) as standard substrates with catalytic copper­(II) acetate at 60 °C (Table 1). Initial testing revealed lower yields when the 1,3-diene is used as the limiting reagent (entry 1). However, the use of Zhdankin’s reagent as the limiting reagent (2 equiv) with excess 1,3-diene provides clear elevation of the yield (entries 2–3). Although many polar solvents are competent in the reaction conditions, methanol provides the best yield (entries 4–7). It was also found that the catalyst loading can be reduced with only a minor reduction in the yield (entries 8–10). As such, 5 mol % of copper­(II) acetate was chosen as the optimal loading.

**Table 1:**
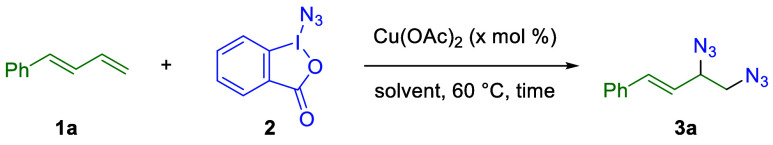
Optimization of the 1,2-Diazidation of 1,3-Dienes^a^

entry	1a (equiv)	2 (equiv)	Cu(OAc)_2_ (mol %)	solvent	time (h)	**3a** (%)^b^
1	1	3	20	THF	5	38
2	1.5	2	20	THF	5	55
3	3	2	20	THF	5	50
4	1.5	2	20	THF	2	77
5	1.5	2	20	DCE	2	69
6	1.5	2	20	PhMe	2	13
7	1.5	2	20	MeOH	2	96
8	1.5	2	10	MeOH	2	89
9	1.5	2	5	MeOH	2	85 (92)^c^
10	1.5	2	1	MeOH	2	76
11	1.5	2	0	MeOH	2	12

a Reactions on a 0.1 mmol scale.

b Reaction yields determined by ^1^H NMR of the crude reaction with dibromomethane as a quantitative internal standard.

c Isolated yield on a 0.3 mmol scale in parenthesis.

With optimized conditions in hand, the substrate scope of this 1,2-diazidation reaction was examined (Table 2). When the electronic effects of the aryl ring of the 1-phenyl-1,3-butadiene were modulated, it was found that electron-neutral, electron-withdrawing, and electron-donating groups at a variety of substitution positions all provide comparable, high yields (**3a–g**). Additionally, the reaction scaled to 1.0 mmol with a slight reduction in the yield. Notably, when the aromatic system on the 1,3-diene is replaced with an aliphatic chain, a nearly equal mixture of 1,2- and 1,4-addition products is detected (**3h**). This result demonstrates the importance of the aryl ring in the original 1,3-diene substrate in controlling the ratio of 1,2:1,4 addition products, likely due to the stability of the conjugated alkene in those 1,2-products. In contrast, the aliphatic systems produce alkenes of similar stability in both the 1,2- and 1,4-products. Furthermore, various disubstituted 1,3-butadiene systems were also tested. 1,1-Diphenyl-1,3-butadiene and 1-phenyl-2-methyl-1,3-butadiene provide comparable yields to the 1-aryl-1,3-butadiene systems (**3i–j**). Slightly reduced yields were noted for 1,3- and 1,4-substitution patterns (**3k–l**) compared with previous systems. There is a clear trend in this substrate series of decreasing yields as the substitution is moved closer to the addition point of the azide group. However, when terminally substituted, aliphatic 1,1,4,4-tetramethyl-1,3-butadiene is used, excellent yields are observed along with a mixture of the 1,2- and 1,4-products (**3m**), aligning with previous observations with aliphatic systems. Finally, styrene produces a high yield and demonstrates the compatibility of this 1,2-diazidation protocol in activated alkene systems (**3n**).

**Table 2:**
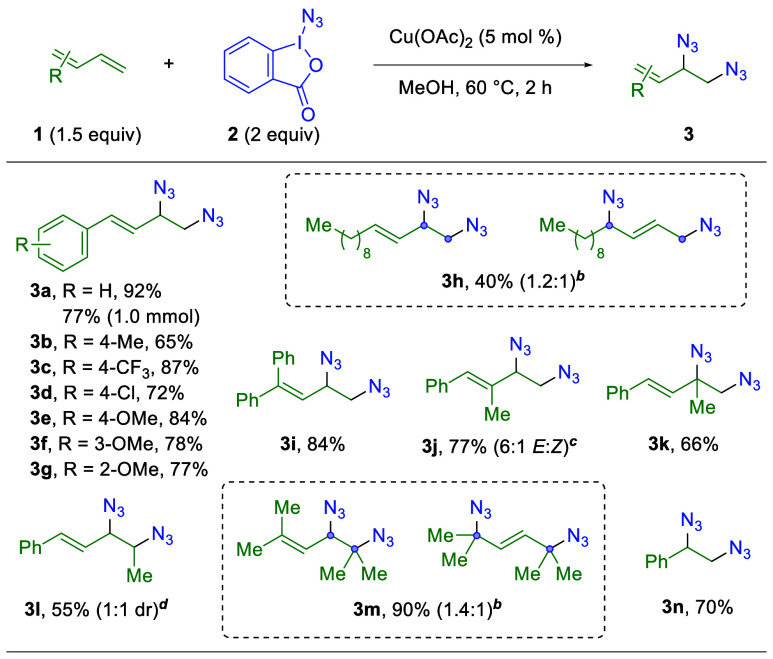
Substrate Scope of 1,3-Dienes in the 1,2-Diazidation of 1,3-Dienes^a^

a Isolated yields on a 0.3 mmol scale.

b Reflects the ratio of 1,2:1,4 addition products, measured by ^1^H NMR of the crude reaction.

c *E*:*Z* ratio determined by ^1^H NMR of the crude reaction.

d dr = diastereomeric ratio, determined by the ^1^H NMR of the crude reaction.

During the optimization of the 1,2-diazidation reaction above, it was noted that copper triflate salts uniquely provided a 1,2-azidooxygenation product (in addition to the desired 1,2-diazidation product) via trapping by the 2-iodobenzoate liberated from Zhdankin’s reagent (see Supporting Information Table S1). A brief optimization campaign centered on producing this 1,2-azidooxygenation product (Supporting Information Table S2) revealed that a 3:1 ratio of 1,3-diene (**1**) to Zhdankin’s reagent (**2**) with 10 mol % copper­(II) triflate in 1,2-dichloroethane provides the best yield (Table 3). Although the yields are low for electronically neutral 1-aryl-1,3-butadiene substrates (**4a–b**), an electron-withdrawing *para*-trifluoromethyl group provides a drastically elevated yield (**4c**). Notably, an electron-donating *para*-methoxy group produces no product (**4d**) and exclusively the 1,2-diazidation product. The use of aliphatic, monosubstituted 1,3-dienes fails to produce the desired product (**4e**). Furthermore, this reaction is amenable to some disubstituted substrates, with 1,1-diphenyl (**4f**) and 1-phenyl-2-methyl (**4g**) 1,3-butadiene systems providing comparable yields to the original substrate (**4a**). However, no product is detected for the 1-phenyl-3-methyl-1,3-butadiene system (**4h**) and 1-phenyl-4,4-dimethyl-1,3-butadiene (**4i**) provides a lower yield, likely due to its sterically hindered nature. As observed previously, aliphatic-substituted substrates, such as disubstituted 2,3-dimethyl-1,3-butadiene, are not viable in the reaction (**4j**). Finally, styrene produces a nearly identical yield (**4k**) to the analogous 1,3-diene case (**4a**), demonstrating the comparable outcome for 1,3-dienes and activated alkenes in this reaction.

**Table 3:**
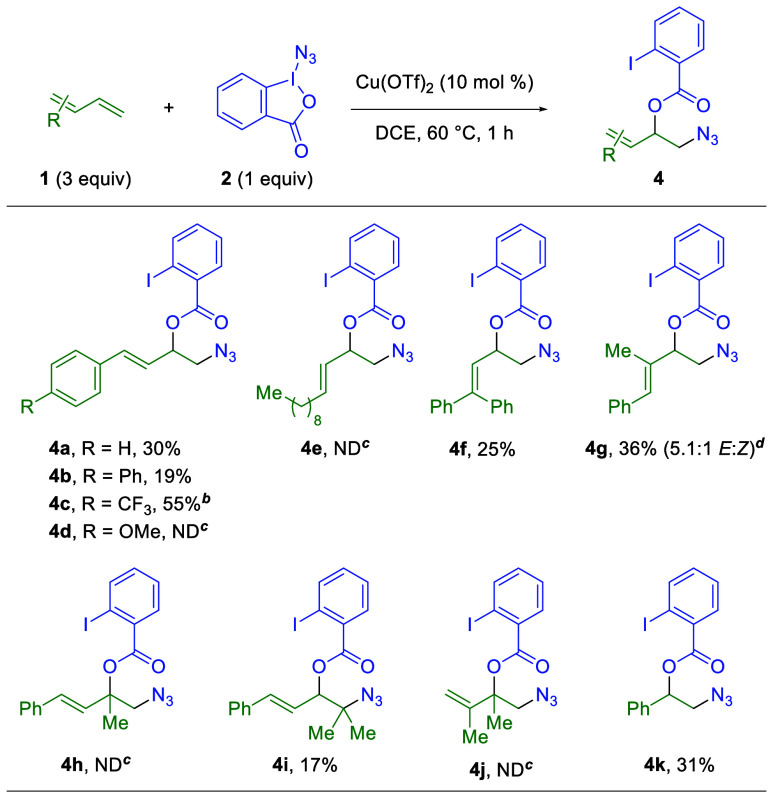
Substrate Scope of 1,3-Dienes in the Two-Component 1,2-Azidooxygenation of 1,3-Dienes^a^

a Isolated yields on a 0.3 mmol scale.

b >20:1 ratio of 1,2:1,4 products (12.9:1 after chromatography).

c ND = not detected by ^1^H NMR of the crude reaction.

d *E*:*Z* ratio determined by ^1^H NMR of the crude reaction.

With the concept of a 1,2-azidooxygenation reaction established, it was considered whether the addition of an exogenous carboxylic acid for a three-component reaction could provide elevated yields of the 1,2-azidooxygenated product over the two-component protocol. As such, pentafluorobenzoic acid (**5a**) was added to the reaction of 1-phenyl-1,3-butadiene (**1a**) and Zhdankin’s reagent (**2**) with 20 mol % copper­(II) acetate (Table 4). It was quickly established that excess Zhdankin reagent (**2**) provides poor yields when compared to excess acid or 1,3-diene (entries 1–3), despite the mass balance in the reactions being unreacted 1,3-diene. Furthermore, acetonitrile was determined to be a superior solvent compared to a selection of polar and nonpolar solvents (entries 4–8). Additionally, decreasing either the acid or 1,3-diene amounts affords reduced yields (entries 9–10). As such, 3 equiv of the acid and 1,3-diene with copper­(II) acetate in acetonitrile was established as the optimal condition, with no 1,2-diazidation products detected.

**Table 4:**
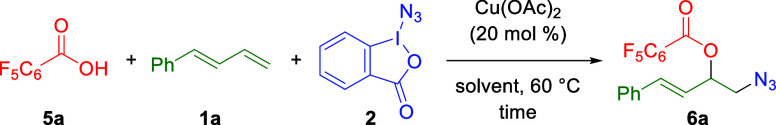
Optimization of 1,2-Azidooxygenation of 1,3-Dienes^a^

entry	5a (equiv)	1a (equiv)	2 (equiv)	solvent	time (h)	**6a** (%)^***b***^
1	1	1	3	DCE	2	3
2	1	3	1	DCE	2	17
3	3	1	1	DCE	2	17
4	3	3	1	DCE	5	29
5	3	3	1	THF	5	4
6	3	3	1	PhMe	5	34
7	3	3	1	MeOH	5	10
8	3	3	1	MeCN	5	49 (47)^***c***^
9	3	2	1	MeCN	5	45
10	2	3	1	MeCN	5	35

a Reactions on a 0.1 mmol scale.

b Reaction yields determined by ^1^H NMR of the crude reaction with dibromomethane as a quantitative internal standard.

c Isolated yield on a 0.3 mmol scale.

With regard to the carboxylic acid scope (Table 5), it was found that the reaction using 2,4-dinitrobenzoic acid (**6b**) provides comparable yields to that of pentafluorobenzoic acid (**6a**). Also, both substrates provide identical yields on a 1.0 mmol scale-up, with no loss of reaction efficiency. Notably, when higher p*K*_a_ acids (above 2.0) were tested, the reaction failed to produce appreciable products (**6c–d**). The same trend was noted with aliphatic carboxylic acids, as dichloroacetic acid produced comparable yields (**6e**) to pentafluoro and 2,4-dinitrobenzoic acids (**6a–b**), but acetic acid failed to produce measurable product (**6f**). This strict dependence on acidity suggests some mechanistic importance of a protonated intermediate compared to the 1,2-diazidation reaction (Table 2).

**Table 5:**
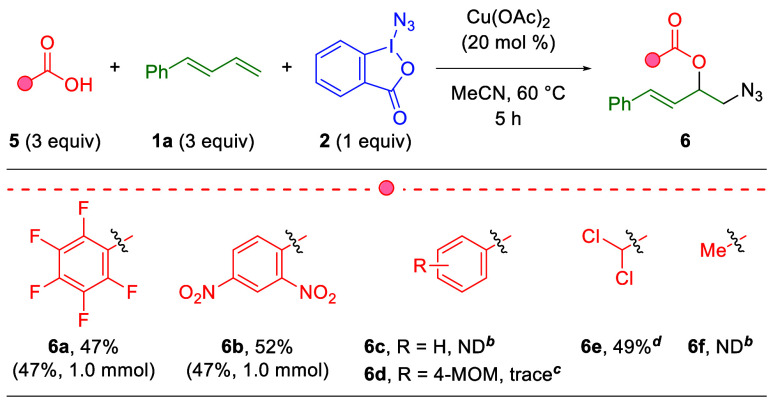
Substrate Scope of Carboxylic Acids in the Three-Component 1,2-Azidooxygenation of 1,3-Diene^a^

a Isolated yields on a 0.3 mmol scale.

b ND = not detected by ^1^H NMR of the crude reaction.

c trace = <5%, as seen by quantitative ^1^H NMR of the crude reaction.

d Contained a 15.3:1 ratio of **6e**:**4a**.

Next, the substrate scope for 1,3-dienes was examined in this three-component 1,2-azidooxygenation using 2,4-dinitrobenzoic acid as the model exogenous carboxylic acid (Table 6). Appending the 1-aryl-1,3-butadiene substrate with electron-neutral and electron-withdrawing substituents produces comparable yields (**7b–d**) to the unsubstituted case (**6b**). However, no product is observed when electron-donating methoxy groups are appended in *para*- or *ortho-*substitution (**7e**, **7g**). Despite this, a *meta*-methoxy group creates no such reduction in yield (**7f**), demonstrating a clear aversion to electron-rich conjugation systems on the reaction product. Furthermore, replacement of the 1-aryl substitution on the 1,3-diene with an aliphatic chain results in no perceptible product (**7h**). Like previous 1,2-diazidation and two-component 1,2-azidooxygenation cases (Tables 2 and 3), the reaction proved to be amenable to 1,1- and 1,2-disubstituted 1,3-diene systems, as 1,1-diphenyl (**7i**) and 1-phenyl-2-methyl (**7j**) substrates produce the 1,2-azidooxygenation product in good yields. Following the previous two-component 1,2-azidooxygenation trend in Tables 3, 1-phenyl-3-methyl-1,3-butadiene produces no product (**7k**) and 1-phenyl-4,4-dimethyl-1,3-butadiene affords the product in a notably reduced yield (**7l**). However, aliphatic disubstituted 1,3-dienes such as isoprene and 2,3-dimethyl-1,3-butadiene produce fair yields (**7m–n**), a result not seen in the previous two-component 1,2-azidooxygenation method. Finally, styrene produces almost no product (**7o**), although the yield can be partially restored by utilizing electron-rich 4-methoxystyrene (**7p**).

**Table 6:**
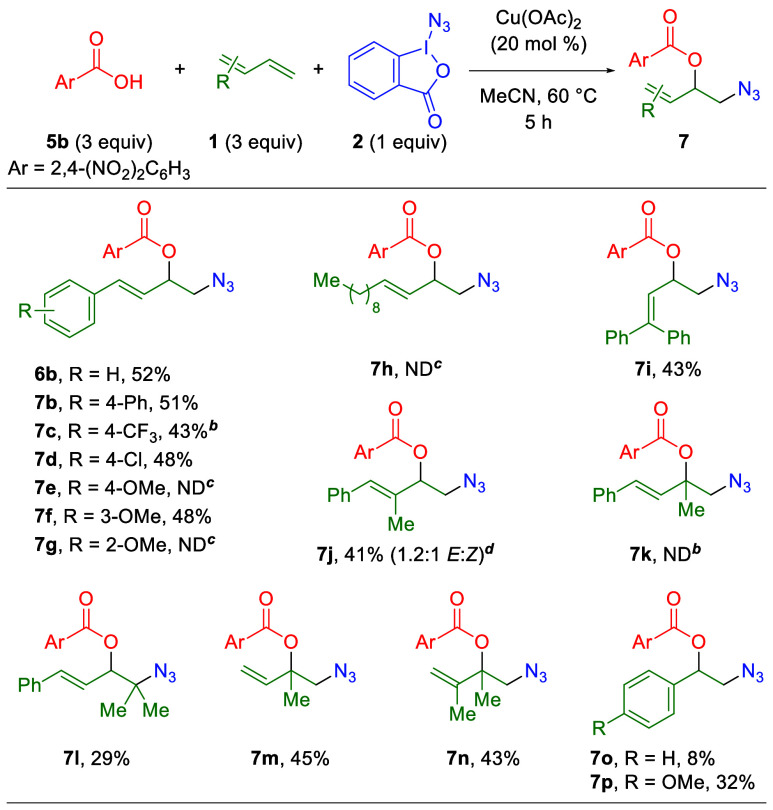
Substrate Scope of 1,3-Dienes in the Three-Component 1,2-Azidooxygenation of 1,3-Diene^a^

a Isolated yields on a 0.3 mmol scale.

b 1.2:1 ratio of 1,2:1,4 products.

c ND = not detected by ^1^H NMR.

d *E*:*Z* ratio determined by ^1^H NMR of the crude reaction.

Given the parallel compatibility of the first two protocols (Tables 2 and 3) for both 1,3-dienes and activated alkenes, competition experiments were performed utilizing both 1-phenyl-1,3-butadiene (**1a**) and styrene (**1r**) (Scheme 3). Despite their compatibility with styrene when used as the exclusive olefin component, both the 1,2-diazidation and two-component 1,2-azidooxygenation protocols strongly prefer the 1,3-diene adducts, as only trace or no styrene adduct is observed (Scheme 4AB). However, the addition of styrene reduces the yield of the 1,2-diazidation product reaction, while the 1,2-azidooxygenation product is achieved in yields similar to those of the styrene-free reaction. Predictably, the three-component 1,2-azidooxygenation protocol also prefers the 1,3-diene adduct, but with essentially no reduction in the yield of the 1,3-diene product when compared to the original (styrene-free) protocol (Scheme 4C).

**Scheme 4:**
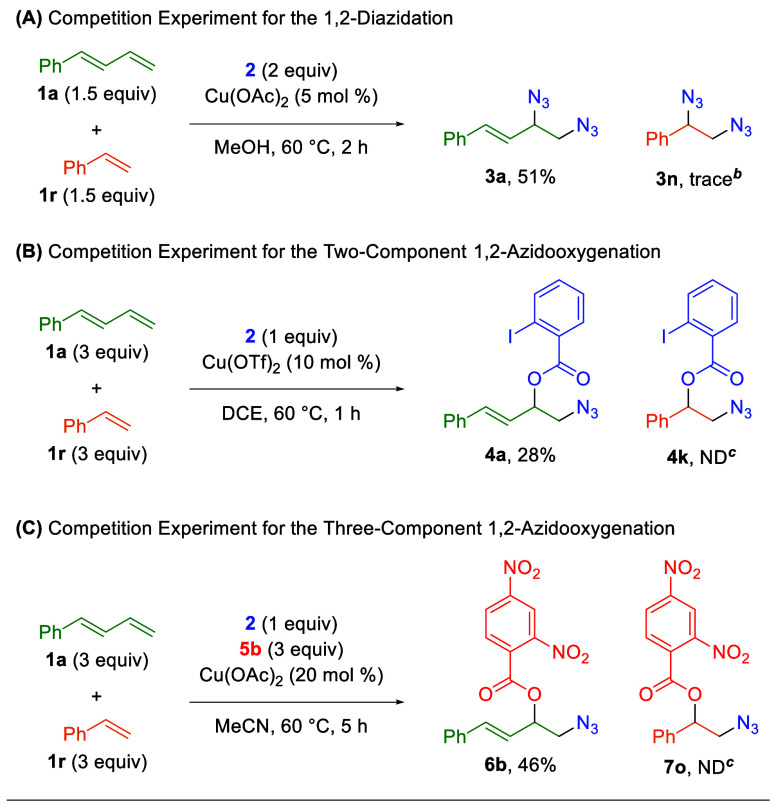
Competition Experiments Between 1,3-Dienes and Alkenes in the Azidation Reactions^a^

a Isolated yields on a 0.3 mmol scale. **2** = Zhdankin’s reagent, **5b** = 2,4-dinitrobenzoic acid.
b Trace = <5% by quantitative ^1^H NMR of the crude reaction.
c ND = not detected by ^1^H NMR of the crude reaction.

The mechanism of these reactions was probed using a variety of radical scavengers (Scheme 5). In the 1,2-diazidation reaction, addition of TEMPO inhibits product formation (**3a**) and provides a 1,2-azidooxyaminyl 1,3-diene adduct (**8**), produced following azido radical addition to the 1,3-diene (Scheme 5A). This demonstrates the radical nature of the 1,2-diazidation reaction, which likely proceeds through liberation of an azido radical upon interaction of the Zhdankin reagent (**2**) with the copper catalyst. This radical then adds to the end of the conjugated diene π-system.

**Scheme 5:**
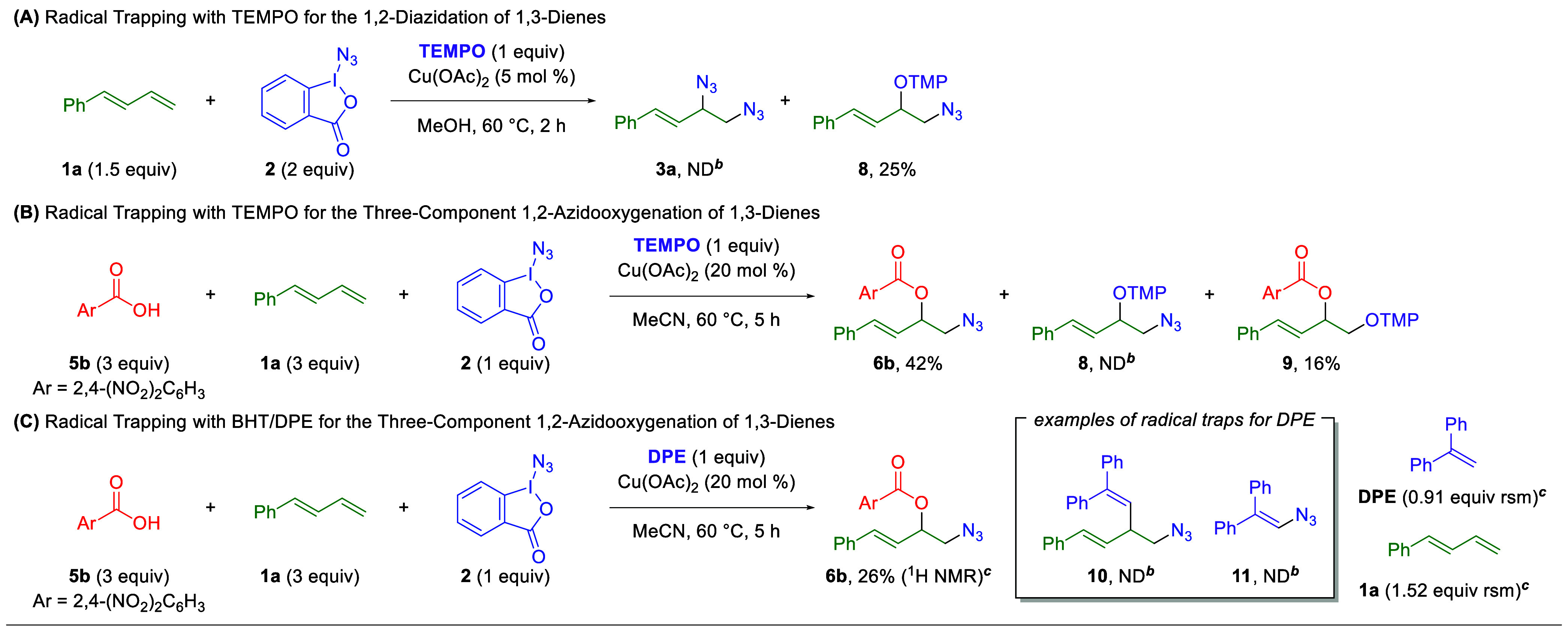
Radical Trapping Experiments^a^

a Isolated yields on a 0.3 mmol scale. Ar = 2,4-dinitrobenzene.
b ND = Not detected by ^1^H NMR of the crude reaction.
c Yield determined by ^1^H NMR of the crude reaction with dibromomethane as an internal standard.

However, inclusion of TEMPO in the three-component 1,2-azidooxygenation protocol does not produce the radical-trapped 1,2-azidooxyaminyl adduct (**8**, Scheme 5B), but it does produce a comparable yield of the 1,2-azidooxygenation product (**6b**) as the standard conditions and a small amount of the 1,2-dioxygenation adduct (**9**) demonstrated in our previous work.^21^ Additional radical trapping agents such as DPE or BHT were tested (Scheme 5C), but no radical trapping products were observed in either case, either through trapping on the diene (e.g., **10**) or through trapping of the azide group (e.g., **11**). Additionally, a small amount of the 1,2-azidooxygenation product was observed when DPE was used. The formation of the 1,2-azidooxygenation product in the presence of radical scavengers suggests that the 1,2-azidooxygenation reaction does not proceed via a radical pathway, in direct contrast with the 1,2-diazidation reaction.

In the three-component 1,2-azidooxygenation reaction (Table 5), it was noted that only carboxylic acids of lower p*K*_a_ display reactivity similar to our previous work on 1,2-dioxygenation of 1,3-dienes.^21^ Given the lack of an ostensible purpose for the acidity under these reaction conditions, further investigations were undertaken to understand this requirement (Scheme 6). When sodium 2,4-dinitrobenzoate (**5b’**) is used in the reaction in place of the corresponding acid, <3% of the 1,2-azidooxygenation product is detected, confirming the necessity of the acid for the desired reaction (Scheme 6A). This result is despite the fact that UV–vis studies suggest that the acid is not initially deprotonated in the presence of Zhdankin’s reagent (Supporting Information, Figure S7). Additionally, when dichloroacetic acid (**5e**) is combined with equimolar sodium 2,4-dinitrobenzoate (**5b’**), both ester products (**6e** and **6b**) are found in nearly equal amounts (Scheme 6B). This experiment demonstrates the viability of carboxylate starting materials when another proton source is present.

**Scheme 6:**
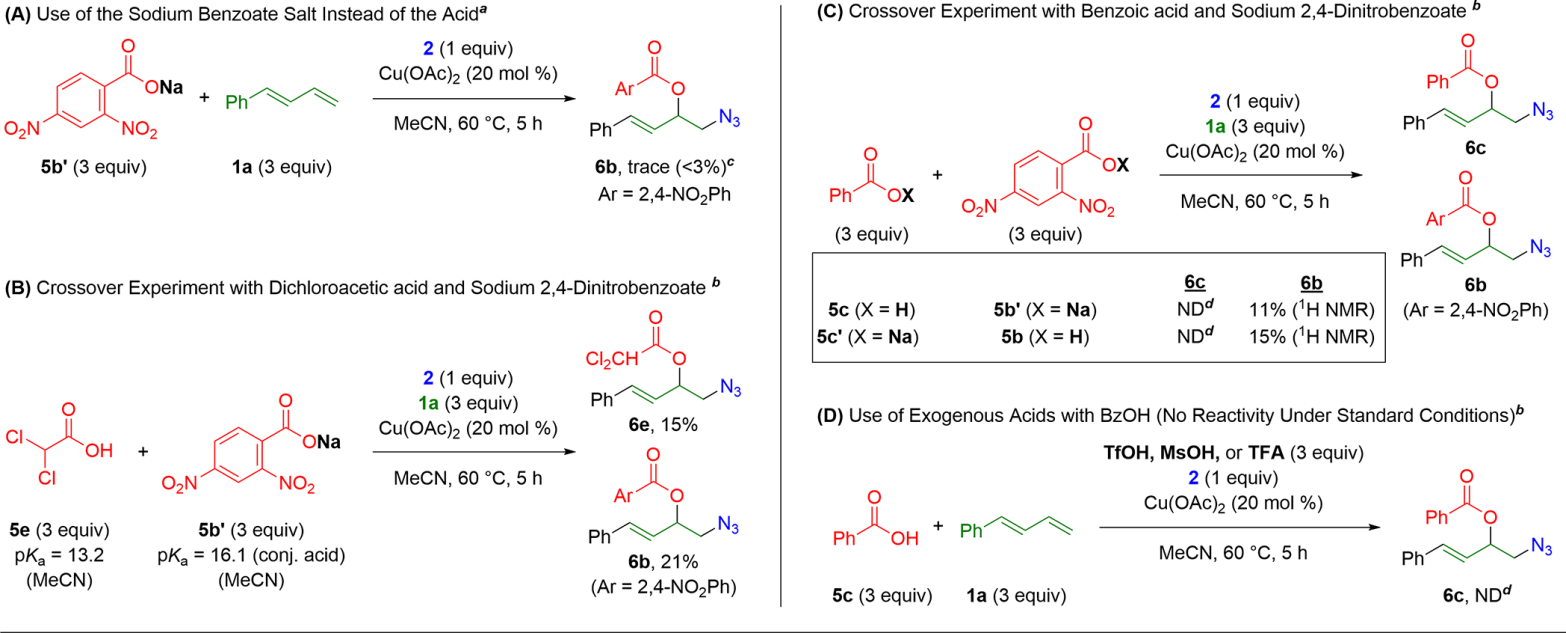
Mechanistic Insights on the Three-Component 1,2-Azidooxygenation of 1,3-Dienes[Fn sch6-fn1]

a
**2** = Zhdankin’s reagent. **1a** = 1-phenyl-1,3-butadiene.
b Run on a 0.3 mmol scale. 1,2-Diazidation product **3a** was observed in 39% yield by ^1^H NMR of the crude reaction.
c Trace = <3% by ^1^H NMR of the crude reaction.
d Run on a 0.1 mmol scale.
e ND = not detected by ^1^H NMR of the crude reaction.
f Reaction yields determined by ^1^H NMR of the crude reaction with dibromomethane as an internal standard.

However, this concept was further tested with additional crossover experiments involving benzoic acid (Scheme 6C). When benzoic acid (**5c**, nonviable in standard 1,2-azidooxygenation conditions) is combined with sodium 2,4-dinitrobenzoate (**5b’**), a small amount of the 2,4-dinitrobenzoate adduct is formed (**6b**), but none of the benzoate adduct is detected (**6c**). When the inverse was tested (sodium benzoate **5c’** with 2,4-dinitrobenzoic acid **5b**), a nearly identical result occurs. This insinuates that the initial source of the proton is inconsequential in determining the reaction outcome. Given the p*K*_a_ differences between the benzoic and 2,4-dinitrobenzoic acids, it is likely that the proton rests on the benzoate group in these reactions, but small equilibrium amounts of 2,4-dinitrobenzoic acid are formed to achieve the protonation state necessary for the reaction.

Accordingly, it was considered that exogenous, non-nucleophilic acids might be capable of serving as the requisite acid, allowing nucleophilic trapping by benzoic acid (Scheme 6D). However, when TfOH, MsOH, or TFA is added to the reaction mixture, no product is detected (Scheme 6D). This suggests that either a) the acidic environment required is incredibly specific and carboxylic acids of a specific p*K*_a_ range are the only source suited to provide this requisite acidity or b) benzoic acid is nonviable as a nucleophile to facilitate the reaction (rather than just noncompetitive). Ultimately, despite these results (Scheme 6) and the absence of radical-trapped products (Scheme 5B,C), it is still difficult to provide a definitive mechanism for the 1,2-azidooxygenation. However, the presence of the 1,2-azidooxygenation product, even in the presence of radical scavengers, advocates against a radical pathway. (See Supporting Information Figures S8 and S9 for possible mechanistic rationale.) Work is underway in our laboratory to further investigate this mechanism and the basis for the acid dependence.

Finally, it was noted that although the 1,2-diazidation condition provides comparable yields for both 1-phenyl-1,3-butadiene and styrene (Table 2, **3a** vs **3n**), the 1,2-azidooxygenation reaction produces only appreciable products with 1-phenyl-1,3-butadiene but not with styrene (Table 6, **6b** vs **7o**). To better understand this discrepancy and how it might relate to potential mechanistic differences, time course measurements were undertaken for all four cases (Scheme 7). In all cases, the Zhdankin reagent (**2**) was fully consumed within the first 15 min despite the product yield continuing to increase beyond this point. This suggests that the reaction of the Zhdankin reagent with the copper catalyst leads to rapid consumption of the material, despite the potential mechanistic differences between the 1,2-diazidation and 1,2-azidooxygenation reactions. Notably, the majority of 1,2-addition product formation for all four cases was also completed within the first 0.5 h of the reaction, mirroring the rapid consumption of the Zhdankin reagent. For the 1,2-diazidation reactions, a similar reaction profile was observed for the reaction with both 1-phenyl-1,3-butadiene and styrene (Scheme 7A,B). In both cases, the olefin source decreases until the 1–2 h mark before plateauing at approximately 0.13 equiv (∼8%) of the starting olefin. Similarly, the product formation reaches a maximum value around the same 1 h time point. In the 1,2-azidooxygenation reaction of 1-phenyl-1,3-butadiene, the 1,3-diene consumption stalls around 1.5 equiv (50%) of the starting olefin (Scheme 7C). Additionally, the product formation plateaued at 50% yield around the 1 h mark. In the case of the addition to styrene (Scheme 7D), although a full equivalent of the styrene is consumed by 3 h (with corresponding consumption plateau), almost no product is formed. This could suggest that the one equivalent of the azide reagent is adding onto the styrene but not continuing through to product formation.

**Scheme 7:**
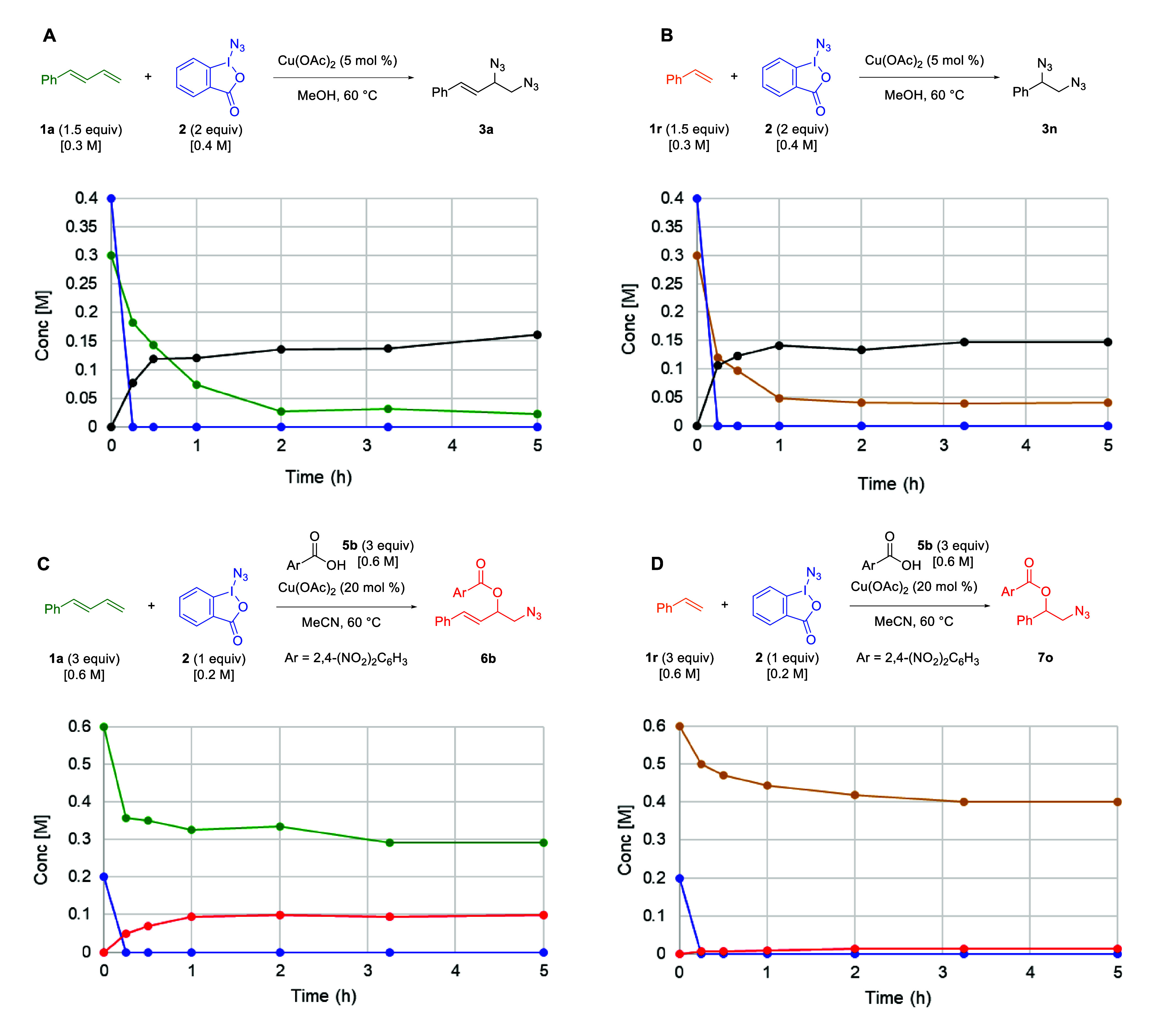
Time Course (Kinetic) Plots for the 1,2-Diazidation (A and B) and 1,2-Azidooxygenation (C and D) Reactions for 1,3-Dienes and Alkenes

## Conclusion

In conclusion, we report three divergent protocols for the azidation of 1,3-dienes: 1,2-diazidation, two-component 1,2-azidooxygenation, and three-component 1,2-azidooxygenation. These rapid reactions proceed in fair to excellent yields with chemo-, regio-, and site-selectivity and have been demonstrated on a wide variety of 1,3-dienes. Additionally, trends in the formation of 1,2- versus 1,4-addition products have been noted. Future work will involve further investigation of the three-component 1,2-azidooxygenation protocol, specifically with regard to its acid requirements.

## Supplementary Material



## Data Availability

The data underlying this study are available in the published article and its Supporting Information.

## Notes

Experimental procedures and full characterization data for all new compounds with copies of ^1^H, ^13^C, and ^19^F spectra, time-course monitoring plots, UV–vis studies, and possible mechanistic rationale (PDF)
