# Supplementary material for: Copper-Catalyzed 1,2-Diazidation and 1,2-Azidooxygenation of 1,3-Dienes: Three Divergent Protocols Using Zhdankin’s Reagent
Source: J Org Chem. 2025 Dec 11;90(51):18188–96. doi: 10.1021/acs.joc.5c02179 (PMC12751017; doi:10.1021/acs.joc.5c02179)
Supplement: Supplementary file 1 [file jo5c02179_si_001.pdf]

SUPPORTING INFORMATION for

Copper-Catalyzed 1,2-Diazidation and 1,2-Azidooxygenation of 1,3-Dienes: Three Divergent Protocols Using Zhdankin's Reagent

Adriana E. Barni, Megan A. George, Jacob R. Pangborn, and Brett N. Hemric*

Department of Chemistry and Biochemistry, University of Tampa, Tampa, FL 33606

Email: bhemric@ut.edu

Table of Contents **Page**

1. General Methods S2
2. Condition Optimization
   2a. 1,2-Diazidation of 1,3-Dienes S2
   2b. Two-Component 1,2-Azidooxygenation of 1,3-Dienes S4
   2c. Three-Component 1,2-Azidooxygenation of 1,3-Dienes S5
3. Synthesis of Starting Materials S7
4. Protocol and Characterization of Novel Compounds
   4a. 1,2-Diazidation of 1,3-Dienes S8
   4b. Two-Component 1,2-Azidooxygenation of 1,3-Dienes S12
   4c. Three-Component 1,2-Azidooxygenation of 1,3-Dienes S15
5. Competition Experiments S20
6. Controls and Mechanistic Investigation Experiments S21
7. Time-Course Monitoring Experiments S24
8. UV-Visible Spectroscopy Studies S27
9. Possible Mechanistic Rationale S28
10. References S29
11. ^{1}H, $^{13}C\{^{1}H\}$, and ^{19}F NMR Spectra S30

1. General Methods

General Procedures

Stir bars were cleaned stirring in concentrated nitric acid overnight, rinsed with water and acetone, dried in an oven at 120 °C overnight and cooled/stored in a desiccator filled with Drierite. Optimization and substrate screens were performed in 1- or 2-Dram glass vials with Teflon-coated micro stir bar. All other reactions were performed in round-bottom flasks with rubber septa and Teflon-coated stir bars, unless otherwise noted. Plastic syringes were used for the transfer of pure solvents and glass pipets were used for transfer of crude reaction solutions. Analytical thin-layer chromatography (TLC) was performed using aluminum plates coated with a 0.25 mm layer of 230–400 mesh silica gel with fluorescent indicator (254 nm). TLC plates were visualized by exposure to ultraviolet light and treatment with vanillin or $KMnO_4$ stain. Organic solutions were concentrated under reduced pressure using a rotary evaporator. Flash column chromatography was performed using 60 Å silica gel or a Teledyne ISCO Combiflash RF+ with RediSep Gold Silica Gel Flash Columns.

Materials

Commercial reagents and anhydrous solvents were used as received.

Instrumentation

Nuclear magnetic resonance spectra were recorded on a JEOL 400 MHz cryoprobe at room temperature unless otherwise noted. Chemical shifts for ^{1}H NMR are reported in parts per million (ppm, δ) and referenced to residual protium in $CDCl_3$ (δ 7.26). Chemical shifts for $^{13}C\{^{1}H\}$ NMR are reported as total carbons in parts per million (ppm, δ) and referenced to the carbon resonances of $CDCl_3$ (δ 77.0) unless otherwise noted. Chemical shifts for ^{19}F NMR are reported in parts per million (ppm, δ) and referenced to the fluorine resonance of an added $PhCF_3$ standard (δ –63.3) unless otherwise noted. NMR values are reported as follows: chemical shift, multiplicity (s = singlet, d = doublet, t = triplet, q = quartet, p = pentet, m = multiplet, br = broad), coupling constant (Hz), and integration. Infrared spectroscopic data was obtained on a Thermo Nicolet iS10 FTIR and is reported in wavenumbers (cm^{-1}). High-resolution mass spectra of compounds were obtained using a Thermo Scientific Orbitrap Exploris 120 electrospray ionization mass spectrometer, introduced through direct injection (10 or 100 µL/min flow rate, 80 psi nitrogen gas curtain). For nitro-containing compounds run in negative mode, NaCl was spiked in as an anionizing reagent to stabilize the parent mass. Several high-resolution mass spectra were obtained through the Duke University Mass Spectrometry Facility using a liquid chromatography-electrospray ionization mass spectrometer with TOF analysis. For some of these samples, LiBr was spiked in as a cationizing agent to stabilize the parent mass. UV-Vis spectra were obtained on an Agilent Cary 300 double-beam UV-Vis spectrophotometer using semi-microscale quartz cuvettes.

2. Condition Optimization

2a. 1,2-Diazidation of 1,3-Dienes

General Example of Optimization Screening Conditions

To a 1-dram vial equipped with a Teflon-coated stir bar was added copper(**II**) acetate (0.9 mg, 0.005 mmol, 0.05 equiv). Methanol (0.5 mL) was added, followed by sequential addition of 1-phenyl-1,3-butadiene **1a** (19.5 mg, 0.15 mmol, 1.5 equiv) and Zhdankin's reagent **2** (57.8 mg, 0.2 mmol, 2.0 equiv). The vial was capped and stirred at 60 °C in an aluminum heating block for 2 h. The resulting crude mixture was filtered through activated, neutral Al_2O_3 (Brockman Grade **I**, 58–60 Å mesh powder) and concentrated *in vacuo* to yield the crude product. Yields were determined by quantitative ^{1}H NMR spectroscopy through addition of $CDCl_3$ (0.5 mL) and dibromomethane (7.0 µL, 0.1 mmol via a 10 µL microsyringe) to the crude reaction. The resulting solution was analyzed by ^{1}H NMR with a 45° pulse angle and 4 second relaxation delay with 16 scans. The resulting spectra were analyzed in MestReNova, with the dibromomethane singlet peak (found at 4.905 ppm) set to a relative integration of 2.0.

Table S1. Condition Survey for the 1,2-Diazidation of 1,3-Dienes[a]

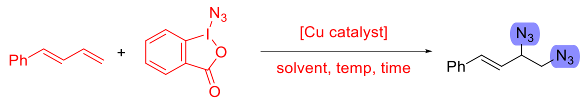

1a 2 3a

entry	1a (equiv)	2	Cu catalyst	(mol %)	solvent	[M]	temp (°C)	time (h)	3a (%)[b]	rsm 1a (equiv)[b]
1	1	3	$Cu(OAc)_2$	20	DCE	0.2	60	5	0	0
2	1	3	$Cu(OAc)_2$	20	PhMe	0.2	60	5	11	0
3	1	3	$Cu(OAc)_2$	20	MeCN	0.2	60	5	25	0
4	1	3	$Cu(OAc)_2$	20	MeOH	0.2	60	5	0	0
5	1	3	$Cu(OAc)_2$	20	THF	0.2	60	5	38	0
6	1	2	$Cu(OAc)_2$	20	THF	0.2	60	5	45	0
7	1.5	2	$Cu(OAc)_2$	20	THF	0.2	60	5	55	0.29
8	2	2	$Cu(OAc)_2$	20	THF	0.2	60	5	53	0.68
9	3	2	$Cu(OAc)_2$	20	THF	0.2	60	5	50	0.77
10	5	2	$Cu(OAc)_2$	20	THF	0.2	60	5	60	1.89
11	1.5	2	$Cu(OAc)_2$	20	THF	0.5	60	5	55	0.07
12	1.5	2	$Cu(OAc)_2$	20	THF	0.33	60	5	41	0.05
13	1.5	2	$Cu(OAc)_2$	20	THF	0.2	60	5	52	0.18
14	1.5	2	$Cu(OAc)_2$	20	THF	0.1	60	5	54	0.30
15	1.5	2	$Cu(OAc)_2$	20	THF	0.2	rt	5	7	1.06
16	1.5	2	$Cu(OAc)_2$	20	THF	0.2	40	5	51	0.34
17	1.5	2	$Cu(OAc)_2$	20	THF	0.2	60	5	58	0.28
18	1.5	2	$Cu(OAc)_2$	20	THF	0.2	80	5	56	0.19
19	1.5	2	$Cu(OTf)_2$	20	THF	0.2	60	5	36[c]	0
20	1.5	2	$[Cu(OTf)]_2$•PhMe	20	THF	0.2	60	5	39[d]	0
21	1.5	2	$Cu(OAc)_2$	20	THF	0.2	60	5	68	0.22
22	1.5	2	CuOAc	20	THF	0.2	60	5	68	0.11
23	1.5	2	$CuCl_2$	20	THF	0.2	60	5	52	0.23
24	1.5	2	CuCl	20	THF	0.2	60	5	69	0.09
25	1.5	2	$Cu(acac)_2$	20	THF	0.2	60	5	70	0.04
26	1.5	2	$CuCO_3$•$Cu(OH)_2$	20	THF	0.2	60	5	40	0.08
27	1.5	2	CuI	20	THF	0.2	60	5	53	0.17
28	1.5	2	$Cu(MeCN)_4BF_4$	20	THF	0.2	60	5	42	0
29	1.5	2	CuPc	20	THF	0.2	60	5	0	0
30	1.5	2	$Cu(eh)_2$	20	THF	0.2	60	5	61	0.15
31	1.5	2	CuTc	20	THF	0.2	60	5	61	0.24
32	1.5	2	$Cu(OAc)_2$	20	THF	0.2	60	2	77	0.35
33	1.5	2	$Cu(OAc)_2$	20	DCE	0.2	60	2	69	0
34	1.5	2	$Cu(OAc)_2$	20	PhMe	0.2	60	2	13	1.04
35	1.5	2	$Cu(OAc)_2$	20	MeCN	0.2	60	2	77	0.23
36	1.5	2	$Cu(OAc)_2$	20	MeOH	0.2	60	2	96	0
37	1.5	2	$Cu(OAc)_2$	10	MeOH	0.2	60	2	89	0.01
38	1.5	2	$Cu(OAc)_2$	5	MeOH	0.2	60	2	85	0.02
39	1.5	2	$Cu(OAc)_2$	1	MeOH	0.2	60	2	76	0
40	1.5	2	$Cu(OAc)_2$	0	MeOH	0.2	60	2	12	0.06

[a]Run on 0.1 mmol scale. [b]Determined with quantitative ^{1}H NMR with dibromomethane (7.0 μL, 0.1 mmol). [c]Provided 21% of azido-oxygenation product (**4a**). [d]Provided 25% of azido-oxygenation product (**4a**).

2b. Two-Component 1,2-Azidooxygenation of 1,3-Dienes

General Example of Optimization Screening Conditions

To a 1-dram vial equipped with a Teflon-coated stir bar was added copper(II) trifluoromethanesulfonate (3.6 mg, 0.01 mmol, 0.1 equiv). 1,2-Dichloroethane (0.5 mL) was added, followed by sequential addition of the 1-phenyl-1,3-butadiene **1a** (39.1 mg, 0.3 mmol, 3.0 equiv) and Zhdankin's reagent **2** (28.9 mg, 0.1 mmol, 1.0 equiv). The vial was capped and stirred at 60 °C in an aluminum heating block for 1 h. The resulting crude mixture was filtered through activated, neutral Al_2O_3 (Brockman Grade I, 58–60 Å mesh powder) and concentrated *in vacuo* to yield the crude product. Yields were determined by quantitative ^1H NMR spectroscopy through addition of $CDCl_3$ (0.5 mL) and dibromomethane (7.0 μL, 0.1 mmol via a 10 μL microsyringe) to the crude reaction. The resulting solution was analyzed by ^1H NMR with a 45° pulse angle and 4 second relaxation delay with 16 scans. The resulting spectra were analyzed in MestReNova, with the dibromomethane singlet peak (found at 4.905 ppm) set to a relative integration of 2.0.

Table S2. Condition Survey for the Two-Component 1,2-Azidooxygenation of 1,3-Dienes[a]

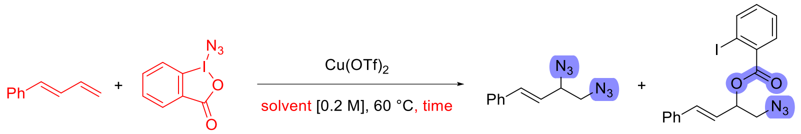

1a **2** **3a** **4a**

entry	1a (equiv)	2 (equiv)	Cu(OTf)$_2$ (mol %)	ligand (10 mol %)	solvent	time (h)	3a (%)[b]	4a (%)[b]	3a+4a (%)[b]	3a:4a	rsm 1a (equiv)[b]
1	1	2	20	–	DCE	4	18	3	21	6.0 : 1	0
2	1	1	20	–	DCE	4	30	4	34	7.5 : 1	0
3	2	1	20	–	DCE	4	15	17	32	1 : 1.1	0
4	3	1	20	–	DCE	4	12	22	34	1 : 1.8	0.40
5	5	1	20	–	DCE	4	10	36	46	1 : 3.6	2.10
6	10	1	20	–	DCE	4	4	40	44	1 : 10	7.90
7	3	1	20	–	DCE	4	17	28	45	1 : 1.6	0.44
8	3	1	20	–	THF	4	12	25	37	1 : 2.1	1.64
9	3	1	20	–	$PhCF_3$	4	4	10	14	1 : 2.5	0.65
10	3	1	20	–	PhMe	4	11	17	28	1 : 1.5	0.94
11	3	1	20	–	MeCN	4	10	13	23	1 : 1.3	0.34
12	3	1	1	–	DCE	4	13	10	23	1.3 : 1	1.60
13	3	1	5	–	DCE	4	10	19	29	1 : 1.9	1.40
14	3	1	10	–	DCE	4	11	23	34	1 : 2.1	0.86
15	3	1	20	–	DCE	4	9	20	29	1 : 2.2	0.31
16	3	1	50	–	DCE	4	6	0	6	–	0
17	3	1	10	Bpy	DCE	4	13	15	28	1 : 1.2	1.14
18	3	1	10	Phen	DCE	4	8	16	24	1 : 2.0	1.23
19	3	1	10	TMEDA	DCE	4	14	7	21	2.0 : 1	1.60
20	3	1	10	Xantphos	DCE	4	16	24	40	1 : 1.5	1.61
21	3	1	10	BINAP	DCE	4	0	0	0	–	0
22	3	1	10	DPPE	DCE	4	9	28	37	1 : 3.1	1.41
23	3	1	10	–	DCE	0.25	14	24	38	1 : 1.7	1.92
24	3	1	10	–	DCE	0.5	10	23	33	1 : 2.3	1.35
25	3	1	10	–	DCE	1	19	34	53	1 : 1.8	1.55
26	3	1	10	–	DCE	4	12	26	38	1 : 2.2	0.89
27	3	1	10	–	DCE	8	9	23	32	1 : 2.6	075
28	3	1	10	–	DCE	16	11	26	37	1 : 2.4	0.87
29	3	1	10	–	DCE	24	15	31	46	1 : 2.1	0.76

[a]Run on 0.1 mmol scale. [b]Determined with quantitative ^1H NMR with dibromomethane (7.0 μL, 0.1 mmol).

2c. Three-Component 1,2-Azidooxygenation of 1,3-Dienes

General Example of Optimization Screening Conditions

To a 2-dram vial equipped with a Teflon-coated stir bar was added copper(**II**) acetate (3.6 mg, 0.02 mmol, 0.2 equiv) and pentafluorobenzoic acid **5a** (63.6 mg, 0.3 mmol, 3.0 equiv). Acetonitrile (0.5 mL) was added, followed by sequential addition of the 1-phenyl-1,3-butadiene **1a** (39.1 mg, 0.3 mmol, 3.0 equiv) and Zhdankin's reagent **2** (28.9 mg, 0.1 mmol, 1.0 equiv). The vial was capped and stirred at 60 °C in an aluminum heating block for 5 h. The resulting crude mixture was filtered through activated, neutral Al_2O_3 (Brockman Grade **I**, 58–60 Å mesh powder) and concentrated *in vacuo* to yield the crude product. Yields were determined by quantitative ^1H NMR spectroscopy through addition of $CDCl_3$ (0.5 mL) and dibromomethane (7.0 µL, 0.1 mmol via a 10 µL microsyringe) to the crude reaction. The resulting solution was analyzed by ^1H NMR with a 45° pulse angle and 4 second relaxation delay with 16 scans. The resulting spectra were analyzed in MestReNova, with the dibromomethane singlet peak (found at 4.905 ppm) set to a relative integration of 2.0.

Table S3. Condition Survey for the Three-Component 1,2-Azidooxygenation of 1,3-Dienes[a]

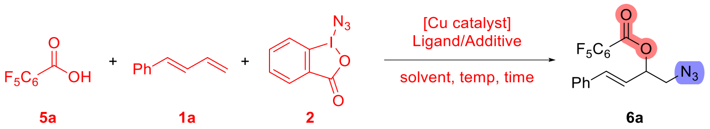

entry	5a (equiv)	1a (equiv)	2 (equiv)	Cu catalyst	(mol %)	ligand/additive (equiv)	solvent	[M]	temp (°C)	time (h)	6a (%)[b]	rsm 1a (equiv)[b]
1	3	1	1	$Cu(OAc)_2$	20	–	DCE	0.2	60	2	17	
2	1	3	1	$Cu(OAc)_2$	20	–	DCE	0.2	60	2	17	
3	1	1	3	$Cu(OAc)_2$	20	–	DCE	0.2	60	2	3	
4	3	1	1	$Cu(OAc)_2$	20	–	THF	0.2	60	2	0	0.13
5	3	1	1	$Cu(OAc)_2$	20	–	EtOH	0.2	60	2	5	0.08
6	3	1	1	$Cu(OAc)_2$	20	–	MeCN	0.2	60	2	15	0
7	3	1	1	$Cu(OAc)_2$	20	–	PhMe	0.2	60	2	11	0
8	3	1	1	$Cu(OAc)_2$	20	–	$PhCF_3$	0.2	60	2	13	0
9	3	1	1	$Cu(OAc)_2$	20	–	MeCN	0.2	60	1	29	0.03
10	3	1	1	$Cu(OAc)_2$	20	–	MeCN	0.2	60	2	31	0.03
11	3	1	1	$Cu(OAc)_2$	20	–	MeCN	0.2	60	5	39	0.03
12	3	1	1	$Cu(OAc)_2$	20	–	MeCN	0.2	60	24	40	0
13	10	3	1	$Cu(OAc)_2$	20	–	MeCN	0.2	60	5	50	1.13
15	5	3	1	$Cu(OAc)_2$	20	–	MeCN	0.2	60	5	56	1.05
16	3	3	1	$Cu(OAc)_2$	20	–	MeCN	0.2	60	5	49	1.11
17	3	2	1	$Cu(OAc)_2$	20	–	MeCN	0.2	60	5	45	0.42
18	3	1	1	$Cu(OAc)_2$	20	–	MeCN	0.2	60	5	42	0
19	2	3	1	$Cu(OAc)_2$	20	–	MeCN	0.2	60	5	35	1.17
20	2	2	1	$Cu(OAc)_2$	20	–	MeCN	0.2	60	5	37	0.57
21	2	1	1	$Cu(OAc)_2$	20	–	MeCN	0.2	60	5	38	0.03
22	3	3	1	$Cu(OTf)_2$	20	–	MeCN	0.2	60	5	0	1.11
23	3	3	1	$[Cu(OTf)]_2$•PhMe	20	–	MeCN	0.2	60	5	12	0.02
24	3	3	1	$Cu(acac)_2$	20	–	MeCN	0.2	60	5	33	1.01
25	3	3	1	$Cu(OAc)_2$	20	–	MeCN	0.2	60	5	51	1.21
26	3	3	1	CuOAc	20	–	MeCN	0.2	60	5	43	1.01
27	3	3	1	$CuCl_2$	20	–	MeCN	0.2	60	5	41	0.79
28	3	3	1	CuCl	20	–	MeCN	0.2	60	5	43	1.20
29	3	3	1	$CuCO_3$•$Cu(OH)_2$	20	–	MeCN	0.2	60	5	37	1.17

Table S3 Continued

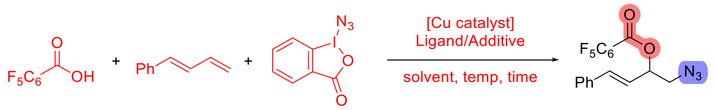

5a **1a** **2** **6a**

entry	5a (equiv)	1a (equiv)	2 (equiv)	Cu catalyst	(mol %)	ligand/additive (equiv)	solvent	[M]	temp (°C)	time (h)	6a (%)[b]	rsm **1a** (equiv)
30	3	3	1	CuI	20	–	MeCN	0.2	60	5	17	2.26
31	3	3	1	$Cu(MeCN)_4BF_4$	20	–	MeCN	0.2	60	5	46	1.19
32	3	3	1	CuPc	20	–	MeCN	0.2	60	5	16	1.56
33	3	3	1	$Cu(eh)_2$	20	–	MeCN	0.2	60	5	49	1.00
34	3	3	1	CuTc	20	–	MeCN	0.2	60	5	48	1.26
35	3	3	1	$Cu(OAc)_2$	0	–	MeCN	0.2	60	5	0	1.25
36	3	3	1	$Cu(OAc)_2$	1	–	MeCN	0.2	60	5	28	0.93
37	3	3	1	$Cu(OAc)_2$	5	–	MeCN	0.2	60	5	33	1.22
38	3	3	1	$Cu(OAc)_2$	10	–	MeCN	0.2	60	5	36	0.96
39	3	3	1	$Cu(OAc)_2$	20	–	MeCN	0.2	60	5	58	1.23
40	3	3	1	$Cu(OAc)_2$	40	–	MeCN	0.2	60	5	47	1.26
41	3	3	1	$Cu(OAc)_2$	20	none	MeCN	0.2	60	5	48	1.32
42	3	3	1	$Cu(OAc)_2$	20	Pyr (0.4)	MeCN	0.2	60	5	37	1.23
43	3	3	1	$Cu(OAc)_2$	20	Bpy (0.2)	MeCN	0.2	60	5	29	1.27
44	3	3	1	$Cu(OAc)_2$	20	Phen (0.2)	MeCN	0.2	60	5	36	1.24
45	3	3	1	$Cu(OAc)_2$	20	TMEDA (0.2)	MeCN	0.2	60	5	33	1.29
46	3	3	1	$Cu(OAc)_2$	20	PPh_3 (0.4)	MeCN	0.2	60	5	43	1.17
47	3	3	1	$Cu(OAc)_2$	20	Xantphos (0.2)	MeCN	0.2	60	5	37	1.11
48	3	3	1	$Cu(OAc)_2$	20	BINAP (0.2)	MeCN	0.2	60	5	38	1.26
49	3	3	1	$Cu(OAc)_2$	20	DPPE (0.2)	MeCN	0.2	60	5	36	1.41
50	3	3	1	$Cu(OAc)_2$	20	–	MeCN	0.2	rt	5	5	1.26
51	3	3	1	$Cu(OAc)_2$	20	–	MeCN	0.2	40	5	30	1.23
52	3	3	1	$Cu(OAc)_2$	20	–	MeCN	0.2	60	5	55	1.25
53	3	3	1	$Cu(OAc)_2$	20	–	MeCN	0.2	80	5	49	1.05
54	3	3	1	$Cu(OAc)_2$	20	–	MeCN	0.2	100	5	51	1.50
55	3	3	1	$Cu(OAc)_2$	20	–	MeCN	0.2	40	18	41	0.78
56	3	3	1	$Cu(OAc)_2$	20	–	DCE	0.2	60	5	29	1.33
57	3	3	1	$Cu(OAc)_2$	20	–	THF	0.2	60	5	4	1.36
58	3	3	1	$Cu(OAc)_2$	20	–	MeOH	0.2	60	5	10	1.19
59	3	3	1	$Cu(OAc)_2$	20	–	MeCN	0.2	60	5	47	1.34
60	3	3	1	$Cu(OAc)_2$	20	–	PhMe	0.2	60	5	34	1.43
61	3	3	1	$Cu(OAc)_2$	20	–	$PhCF_3$	0.2	60	5	30	1.16
62	3	3	1	$Cu(OAc)_2$	20	–	HFIP	0.2	60	5	0	0
63	3	3	1	$Cu(OAc)_2$	20	–	MeCN	0.1	60	5	49	1.40
64	3	3	1	$Cu(OAc)_2$	20	–	MeCN	0.2	60	5	48	1.04
65	3	3	1	$Cu(OAc)_2$	20	–	MeCN	0.3	60	5	38	0.66
66	3	3	1	$Cu(OAc)_2$	20	–	MeCN	0.5	60	5	43	0.80
67	3	3	1	$Cu(OAc)_2$	20	–	MeCN	1.0	60	5	46	0.78
68	3	3	1	$Cu(OAc)_2$	20	None	MeCN	0.2	60	5	49	1.00
69	3	3	1	$Cu(OAc)_2$	20	DBU (1)	MeCN	0.2	60	5	46	1.09
70	3	3	1	$Cu(OAc)_2$	20	Proton sponge (1)	MeCN	0.2	60	5	4	1.79
71	3	3	1	$Cu(OAc)_2$	20	Hünig's base (1)	MeCN	0.2	60	5	18	1.36
72	3	3	1	$Cu(OAc)_2$	20	Cs_2CO_3 (1)	MeCN	0.2	60	5	0	2.47
73	3	3	1	$Cu(OAc)_2$	20	DABCO (1)	MeCN	0.2	60	5	6	1.34
74	3	3	1	$Cu(OAc)_2$	20	Quinoline (1)	MeCN	0.2	60	5	32	1.05
75	3	3	1	$Cu(OAc)_2$	20	PPTS (1)	MeCN	0.2	60	5	23	0.94

[a]Run on 0.1 mmol scale. [b]Determined with quantitative ^{1}H NMR with dibromomethane (7.0 μL, 0.1 mmol).

3. Synthesis of Starting Materials

3.1 Zhdankin Reagent

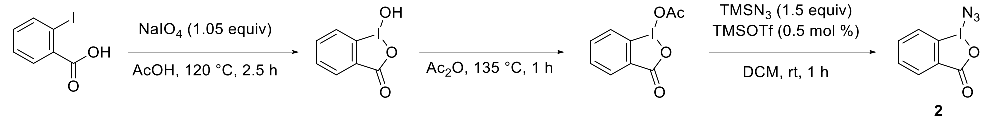

Zhdankin's reagent (**2**) was synthesized as previously reported.[1]

3.2 Carboxylic acids

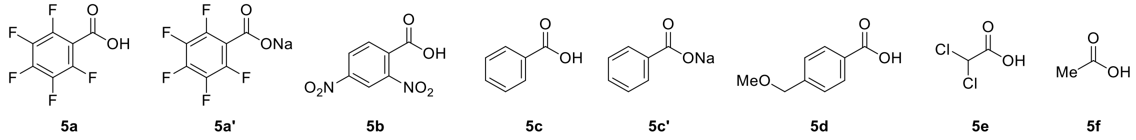

Acids **5a, 5b, 5c, 5c', 5e,** and **5f** were purchased from commercial suppliers and used as received. Substrates **5a'** and **5d** was synthesized as previously reported.[2]

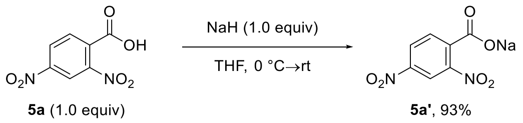

Sodium 2,4-dinitrobenzoate (5b'). To a stirring solution of anhydrous sodium hydride (240.0 mg, 50.0 mmol, 1.0 equiv) in THF (40 mL) at 0 °C slowly was added 2,4-dinitrobenzoic acid (2.12 g, 10.0 mmol, 1.0 equiv). Rapid gas evolution was observed and an off-white precipitate was formed. After 5 min, the reaction was allowed to stir at room temperature for 1 h. The suspension was concentrated in vacuo to afford **5b'** as a tan solid (2.19 g, 100%). **^{1}H NMR** (D_2O, 400 MHz): δ 8.91 (s, 1H), 8.54 (d, *J* = 8.4 Hz, 1H), 7.70 (d, *J* = 8.4 Hz); **^{13}C{^{1}H} NMR** (D_2O, 100 MHz): δ 173.0, 147.6, 145.2, 141.5, 129.9, 129.6, 120.8; **FTIR** (solid): cm^{-1} 3112, 3059, 2873, 1655, 1632, 1605, 1595, 1521, 1394, 1347, 1059, 906, 826, 796, 727, 692, 665; **HRMS** (ESI^-, m/z): Calcd for $C_6H_3N_2O_4^-$ ($[M–CO_2Na]^-$): 167.0098; found: 167.0098.

3.3 1,3-Dienes

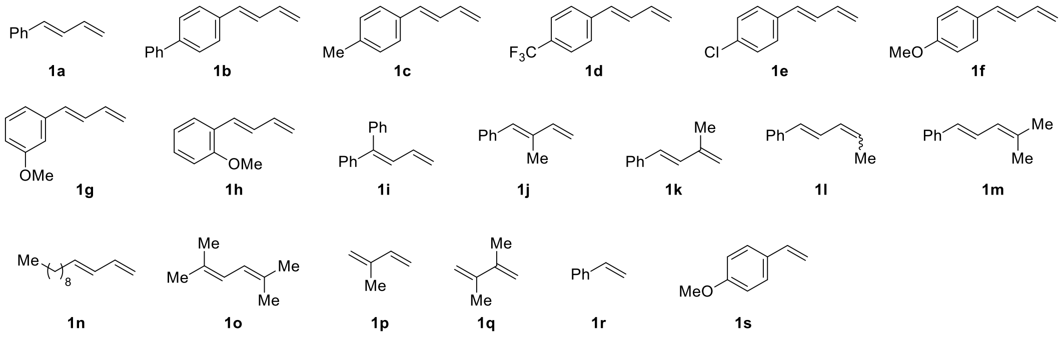

Olefins **1o–s** were purchased from commercial suppliers and used as received.

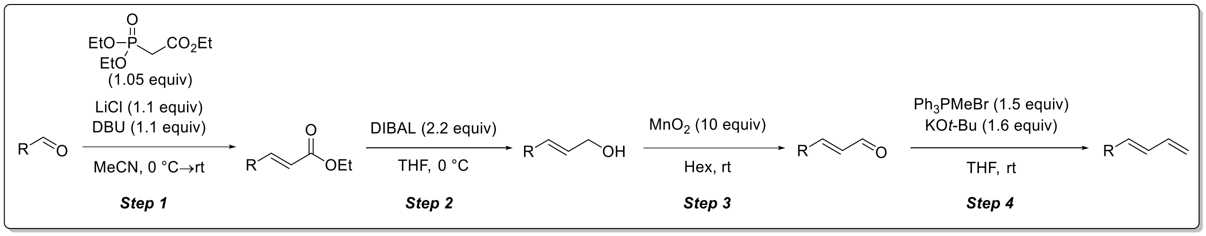

Olefins **1a**, **1e**, **1f**, **1h**, **1j**, **1k**, and **1n** were synthesized as previously reported using step 4 above.[2]

Olefins **1b**, **1c**, **1d**, and **1g**. were synthesized as previously reported using steps 1–4 above.[2]

Olefin **1l** was synthesized as previously reported using a modified step 4 below.[2]

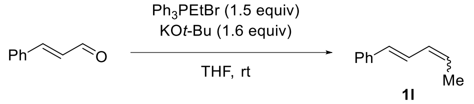

1l

Olefin **1m** was synthesized as previously reported using a modified step 4 below.[2]

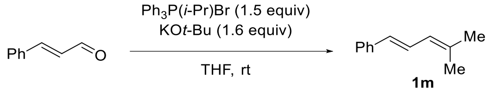

1m

Olefin **1i** was synthesized as previously reported using steps 5–6 below.[2]

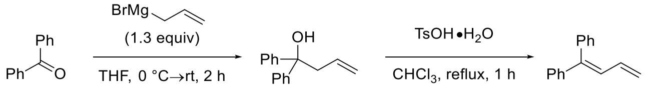

Step 5 **Step 6** **1i**

4. Protocols and Characterization of Novel Compounds

4a. Diene 1,2-Diazidation

Standard Condition I: 1,2-Diazidation of 1,3-dienes

To a 1-dram vial equipped with a Teflon-coated stir bar was added copper(II) acetate (2.7 mg, 0.015 mmol, 0.05 equiv). Methanol (1.5 mL) was added, followed by sequential addition of the 1,3-diene **1** (0.45 mmol, 1.5 equiv) and Zhdankin's reagent **2** (173.4 mg, 0.6 mmol, 2.0 equiv). The vial was capped and stirred at 60 °C in an aluminum heating block for 2 h. The resulting crude mixture was filtered through activated, neutral Al_2O_3 (Brockman Grade I, 58–60 Å mesh powder) and concentrated *in vacuo* to yield the crude product. Purification was performed as noted below.

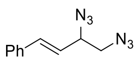

3a

(*E*)-(3,4-Diazidobut-1-en-1-yl)benzene (3a). Synthesized using Standard Condition I. Isolated by flash column chromatography (silica gel, 100% hexanes to 10% ethyl acetate–hexanes) as a clear oil (59.4 mg, 92%). Spectra matched previous reports.[3] (^{1}H NMR, ^{13}C{^{1}H} NMR, and IR included for characterization agreement and assessment of purity.) **^{1}H NMR** ($CDCl_3$, 400 MHz): δ 7.43 (dd, *J* = 8.1, 1.5 Hz, 2H), 7.36 (t, *J* = 7.6 Hz, 2H), 7.31 (t, *J* = 7.1 Hz, 1H), 6.74 (d, *J* = 15.8 Hz, 1H), 6.14 (dd, *J* = 15.8, 8.1 Hz, 1H), 4.27 (ddd, *J* = 8.0, 6.9, 5.0 Hz, 1H), 3.42 (dd, *J* = 12.6, 5.0 Hz, 1H), 3.38 (dd, *J* = 12.5, 6.9 Hz, 1H); **^{13}C{^{1}H} NMR**

($CDCl_3$, 100 MHz): δ 135.5, 135.3, 128.7 (2C), 128.6, 126.8 (2C), 122.9, 63.9, 54.4; **FTIR** (thin film): cm^{-1} 3028, 2925, 2091. 1495, 1449, 1274, 1244, 968, 750, 693.

<u>1.0 mmol scale</u>: To a 2-dram vial equipped with a Teflon-coated stir bar was added copper(**II**) acetate (9.1 mg, 0.05 mmol, 0.05 equiv). Methanol (5.0 mL) was added, followed by sequential addition of 1-phenyl-1,3-butadiene **1a** (195.3 mg, 1.5 mmol, 1.5 equiv) and Zhdankin's reagent **2** (578.1 mg, 2.0 mmol, 2.0 equiv). The vial was capped and stirred at 60 °C in an aluminum heating block for 2 h. The resulting crude mixture was filtered through activated, neutral Al_2O_3 (Brockman Grade **I**, 58–60 Å mesh powder) and concentrated *in vacuo* to yield the crude product. Isolated by flash column chromatography (silica gel, 100% hexanes to 5% ethyl acetate–hexanes) as a clear oil (164.3 mg, 77%).

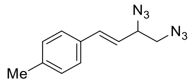

3b

(*E*)-1-(3,4-Diazidobut-1-en-1-yl)-4-methylbenzene (3b). Synthesized using Standard Condition I. Isolated by flash column chromatography (silica gel, 100% hexanes to 10% ethyl acetate–hexanes) as a clear oil (44.7 mg, 65%). Spectra matched previous reports.[3] (R_f, ^{1}H NMR, ^{13}C{^{1}H} NMR, and IR included for characterization agreement and assessment of purity.) $\boldsymbol{R_f}$ = 0.77 (25% EtOAc–hexanes); **^{1}H NMR** ($CDCl_3$, 400 MHz): δ 7.32 (d, *J* = 7.9 Hz, 2H), 7.17 (d, *J* = 7.9 Hz, 2H), 6.71 (d, *J* = 15.8 Hz, 1H), 6.08 (dd, *J* = 15.8, 8.1 Hz, 1H), 4.26 (td, *J* = 7.5, 5.2 Hz, 1H), 3.41 (dd, *J* = 12.6, 5.0 Hz, 1H), 3.37 (dd, *J* = 12.6, 7.0 Hz, 1H), 2.36 (s, 3H); **^{13}C{^{1}H} NMR** ($CDCl_3$, 100 MHz): δ 138.7, 135.5, 132.5, 129.4 (2C), 126.7 (2C), 121.8, 64.0, 54.5, 21.2; **FTIR** (thin film): cm^{-1} 3027, 2923, 2090, 1514, 1247, 970, 802;

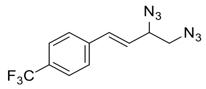

3c

(*E*)-1-(3,4-Diazidobut-1-en-1-yl)-4-(trifluoromethyl)benzene (3c). Synthesized using Standard Condition I. Isolated by flash column chromatography (silica gel, 100% hexanes to 10% ethyl acetate–hexanes) as a clear oil (73.4 mg, 87%). $\boldsymbol{R_f}$ = 0.71 (25% EtOAc–hexanes); **^{1}H NMR** ($CDCl_3$, 400 MHz): δ 7.61 (d, *J* = 8.1 Hz, 2H), 7.51 (d, *J* = 8.2 Hz, 2H), 6.77 (d, *J* = 15.8 Hz, 1H), 6.23 (dd, *J* = 15.8, 7.8 Hz, 1H), 4.32–4.25 (m, 1H), 3.45 (dd, *J* = 12.9, 4.9 Hz, 1H), 3.40 (dd, *J* = 12.8, 6.8 Hz, 1H); **^{13}C{^{1}H} NMR** ($CDCl_3$, 100 MHz): δ 138.7, 133.8, 130.3 (q, $^2J_{C\text{-}F}$ = 32.5 Hz) 126.9 (2C), 125.7, 125.7 (q, $^3J_{C\text{-}F}$ = 3.8 Hz, 2C), 123.9 (q, $^1J_{C\text{-}F}$ = 272.0 Hz), 63.5, 54.3; **^{19}F NMR** ($CDCl_3$, 376.5 MHz): δ –63.2 (s, 3F); **FTIR** (thin film): cm^{-1} 3046, 2930, 2094, 1616, 1321, 1267, 1163, 1109, 1066, 1016, 970, 818; **HRMS** (ESI^+, m/z): Calcd for $C_{11}H_9F_3N_6Li^+$ ($[M+Li^7]^+$): 289.0995; found: 289.1002.

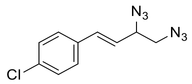

3d

(*E*)-1-Chloro-4-(3,4-diazidobut-1-en-1-yl)benzene (3d). Synthesized using Standard Condition I. Isolated by flash column chromatography (silica gel, 100% hexanes to 15% ethyl acetate–hexanes) as a clear oil (53.9 mg, 72%). Spectra matched previous reports.[3] (^{1}H NMR, ^{13}C{^{1}H} NMR, and IR included for characterization agreement and assessment of purity.) **^{1}H NMR** ($CDCl_3$, 400 MHz): δ 7.35 (d, *J* = 9.0 Hz, 2H), 7.32 (d, *J* = 8.6 Hz, 2H), 6.69 (d, *J* = 15.8 Hz, 1H), 6.10 (dd, *J* = 15.8, 7.9 Hz, 1H), 4.26 (ddd, *J* = 8.0, 6.8, 5.1 Hz, 1H), 3.42 (dd, *J* = 12.6, 6.8 Hz, 1H), 3.37 (dd, *J* = 12.5, 4.9 Hz, 1H); **^{13}C{^{1}H} NMR** ($CDCl_3$, 100 MHz): δ 134.3, 134.2, 133.8, 128.9 (2C), 128.0 (2C), 123.6, 63.7, 54.4; **FTIR** (thin film): cm^{-1} 2926, 2089, 1490, 1240, 1089, 1012, 967, 806.

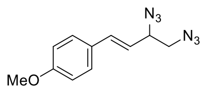

3e

(*E*)-1-(3,4-Diazidobut-1-en-1-yl)-4-methoxybenzene (3e). Synthesized using Standard Condition I. Isolated by flash column chromatography (silica gel, 100% hexanes to 10% ethyl acetate–hexanes) as a clear oil (61.8 mg, 84%). Spectra matched previous report.[3a] (^{1}H NMR, ^{13}C{^{1}H} NMR, and IR included for characterization agreement and assessment of purity.) **^{1}H NMR** ($CDCl_3$, 400 MHz): δ 7.36 (d, *J* = 8.4 Hz, 2H), 6.88 (d, *J* = 8.4 Hz, 2H), 6.67 (d, *J* = 15.8 Hz, 1H), 5.98 (dd, *J* = 15.7, 8.1 Hz, 1H), 4.27–4.20 (m, 1H), 3.82 (s, 3H), 3.40 (dd, *J* = 12.8, 5.1 Hz, 1H), 3.36 (dd, *J* = 12.6, 7.2 Hz, 1H); **^{13}C{^{1}H} NMR** ($CDCl_3$, 100 MHz): δ 159.9, 135.1, 128.1 (2C), 128.0, 120.5, 114.1 (2C), 64.1, 55.3, 54.6; **FTIR** (thin film): cm^{-1} 2923, 2837, 2091, 1606, 1510, 1244, 1174, 1030, 968. 814.

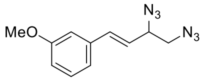

3f

(*E*)-1-(3,4-Diazidobut-1-en-1-yl)-3-methoxybenzene (3f). Synthesized using Standard Condition I. Isolated by flash column chromatography (silica gel, 100% hexanes to 10% ethyl acetate–hexanes) as a clear oil (56.8 mg, 78%). **^{1}H NMR** ($CDCl_3$, 400 MHz): δ 7.27 (t, *J* = 7.9 Hz, 1H), 7.01 (d, *J* = 8.1 Hz, 1H), 6.94 (t, *J* = 2.1 Hz, 1H), 6.86 (dd, *J* = 8.1, 2.6 Hz, 1H), 6.71 (d, *J* = 15.8 Hz, 1H), 6.12 (dd, *J* = 15.8, 8.0 Hz, 1H), 4.29–4.23 (m, 1H), 3.83 (s, 3H), 3.42 (dd, *J* = 12.6, 5.1 Hz, 1H), 3.37 (dd, *J* = 12.5, 7.0 Hz, 1H); **^{13}C{^{1}H} NMR** ($CDCl_3$, 100 MHz): δ 159.8, 136.7, 135.4, 129.7, 123.2, 119.4, 114.2, 112.0, 63.8, 55.2, 53.7; **FTIR** (thin film): cm^{-1} 2932, 2836, 2093, 1598, 1580, 1264, 1157, 1047, 970, 777; **HRMS** (ESI^{+}, m/z): Calcd for $C_{11}H_{12}N_6ONa^{+}$ ($[M+Na]^{+}$): 267.0965; found: 267.0964.

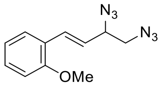

3g

(*E*)-1-(3,4-Diazidobut-1-en-1-yl)-2-methoxybenzene (3g). Synthesized using Standard Condition I. Isolated by flash column chromatography (silica gel, 100% hexanes to 10% ethyl acetate–hexanes) as a clear oil (56.2 mg, 77%). **^{1}H NMR** ($CDCl_3$, 400 MHz): δ 7.45 (dd, *J* = 7.6, 1.1 Hz, 1H), 7.29 (td, *J* = 7.8, 1.3 Hz, 1H), 7.05 (d, *J* = 15.9 Hz, 1H), 6.95 (t, *J* = 7.5 Hz, 1H), 6.90 (d, *J* = 8.3 Hz, 1H), 6.17 (dd, *J* = 16.0, 8.2 Hz, 1H), 4.30–4.24 (m, 1H), 3.87 (s, 3H), 3.42 (dd, *J* = 12.6, 5.0 Hz, 1H), 3.37 (dd, *J* = 12.6, 7.0 Hz, 1H); **^{13}C{^{1}H} NMR** ($CDCl_3$, 100 MHz): δ 157.0, 130.7, 129.7, 127.3, 124.2, 123.3, 120.6, 110.9, 64.5, 55.4, 54.6; **FTIR** (thin film): cm^{-1} 2932, 2838, 2090, 1598, 1489, 1464, 1437, 1242, 1026, 974, 750; **HRMS** (ESI^{+}, m/z): Calcd for $C_{11}H_{12}N_6ONa^{+}$ ($[M+Na]^{+}$): 267.0965; found: 267.0964.

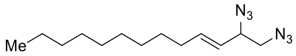

3h

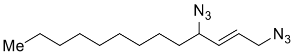

3h'

(*E*)-1,2-Diazidotridec-3-ene (3h) and (*E*)-1,4-Diazidotridec-2-ene (3h’). Synthesized using Standard Condition I. A ratio of 1.2:1 (**3h**:**3h’**) was determined by ^{1}H NMR of the crude reaction. Isolated by flash column chromatography (silica gel, 100% hexanes) as a clear oil and an inseparable 1.2:1 mixture of **3h**:**3h’** (32.0 mg, 40%). R_f = 0.76 (25% EtOAc–hexanes); **^{1}H NMR** ($CDCl_3$, 400 MHz): δ 5.85 (dt, *J* = 14.7, 6.8 Hz, 1H, **3h**), 5.76 (dt, *J* = 15.4, 5.8 Hz, 1H, **3h’**), 5.66 (dd, *J* = 15.5, 7.5 Hz, 1H, **3h’**), 5.39 (dd, *J* = 15.5, 8.2 Hz, 1H, **3h**), 4.04 (q, *J* = 6.8 Hz, 1H, **3h’**), 3.87 (q, *J* = 7.2 Hz, 1H, **3h**), 3.81 (d, *J* = 5.9 Hz, 2H, **3h’**), 3.28 (dd, *J* = 12.7, 5.1 Hz, 1H, **3h**), 3.24 (dd, *J* = 12.6, 6.5 Hz, 1H, **3h**), 2.09 (q, *J* = 7.2 Hz, 2H, **3h**), 1.59–1.18 (m, 30H, **3h**+**3h’**), 0.87 (t, *J* = 6.7 Hz, 6H, **3h**+**3h’**); **^{13}C{^{1}H} NMR** ($CDCl_3$, 100 MHz): δ 138.4, 132.8, 126.5, 123.6, 63.8, 63.7, 54.3, 51.9, 34.3, 32.2, 31.8, 31.8, 29.5, 29.4, 29.4, 29.4, 29.3, 29.2, 29.2, 29.0, 28.8, 25.7, 22.6 (2C), 14.1 (2C); **FTIR** (thin film): cm^{-1} 2924, 2854, 2092, 1465, 1239, 971; **HRMS** (ESI^{+}, m/z): Calcd for $C_{13}H_{24}N_6Li^{+}$ ($[M+{}^{7}Li]^{+}$): 271.2217; found: 271.2222.

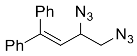

3i

(3,4-Diazidobut-1-ene-1,1-diyl)dibenzene (3i). Synthesized using Standard Condition I. Isolated by flash column chromatography (silica gel, 100% hexanes) as a clear oil (73.1 mg, 84%). ***R_f*** = 0.81 (25% EtOAc–hexanes); **^{1}H NMR** ($CDCl_3$, 400 MHz): δ 7.47–7.38 (m, 3H), 7.35–7.28 (m, 5H), 7.21 (dd, *J* = 8.0, 1.5 Hz, 2H), 6.08 (d, *J* = 9.9 Hz, 1H), 4.26 (dt, *J* = 9.9, 5.9 Hz, 1H), 3.37 (d, *J* = 5.9 Hz, 2H); **^{13}C{^{1}H} NMR** ($CDCl_3$, 100 MHz): δ 148.7, 140.4, 138.1, 129.5 (2C), 128.7 (2C), 128.4, 128.3 (2C), 128.0, 127.5 (2C), 121.7, 60.1, 54.5; **FTIR** (thin film): cm^{-1} 3057, 3025, 2927, 2089, 1492, 1443, 1239, 763, 695; **HRMS** (ESI$^+$, m/z): Calcd for $C_{32}H_{29}N_{12}^+$ ([2M+H]$^+$): 581.2633; found: 581.2639.

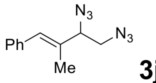

3j

(*E*)-(3,4-Diazido-2-methylbut-1-en-1-yl)benzene (3j). Synthesized using Standard Condition I. A 6:1 *E*:*Z* ratio was determined by ^{1}H NMR of the crude reaction. Isolated by flash column chromatography (silica gel, 100% hexanes to 10% ethyl acetate–hexanes) as a clear oil and a 7:1 *E*:*Z* ratio (52.6 mg, 77%). Spectra matched previous report.[3a] (*R_f*, ^{1}H NMR, ^{13}C{^{1}H} NMR, and IR included for characterization agreement and assessment of purity.) (Minor constitutional isomer not characterized below.) ***R_f*** = 0.75 (25% EtOAc–hexanes); **^{1}H NMR** ($CDCl_3$, 400 MHz): δ 7.37 (dd, *J* = 7.6, 3.2 Hz, 2H), 7.33–7.27 (m, 3H), 6.64 (s, 1H), 4.26–4.20 (m, 1H), 3.43–3.37 (m, 2H), 1.91 (d, *J* = 2.3 Hz, 3H); **^{13}C{^{1}H} NMR** ($CDCl_3$, 100 MHz): δ 136.1, 132.3, 130.9, 129.0 (2C), 128.2 (2C), 127.2, 69.5, 52.8, 13.8; **FTIR** (thin film): cm^{-1} 3026, 2924, 2087, 1442, 1246, 747, 698.

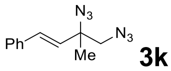

3k

(*E*)-(3,4-Diazido-3-methylbut-1-en-1-yl)benzene (3k). Synthesized using Standard Condition I. Isolated by flash column chromatography (silica gel, 100% hexanes to 5% ethyl acetate–hexanes) as a clear oil (45.4 mg, 66%). Spectra matched previous report.[3b] (^{1}H NMR, ^{13}C{^{1}H} NMR, and IR included for characterization agreement and assessment of purity.) **^{1}H NMR** ($CDCl_3$, 400 MHz): δ 7.44 (d, *J* = 7.5 Hz, 2H), 7.37 (t, *J* = 7.4 Hz, 2H), 7.31 (t, *J* = 7.4 Hz, 1H), 6.72 (d, *J* = 16.1 Hz, 1H), 6.21 (d, *J* = 16.1 Hz, 1H), 3.36 (d, *J* = 12.5 Hz, 1H), 3.32 (d, *J* = 12.5 Hz, 1H), 1.56 (s, 3H); **^{13}C{^{1}H} NMR** ($CDCl_3$, 100 MHz): δ 135.6, 131.9, 128.6 (2C), 128.3, 128.0, 126.7 (2C), 64.7, 59.5, 21.3; **FTIR** (thin film): cm^{-1} 3027, 2978, 2930, 2091, 1493, 1448, 1379, 1246, 968, 746, 692.

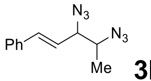

3l

(*E*)-(3,4-Diazidopent-1-en-1-yl)benzene (3l). Synthesized using Standard Condition I. A 1:1 ratio of diastereomers was determined by ^{1}H NMR of the crude reaction. Isolated by flash column chromatography (silica gel, 100% hexanes) as a clear oil and a 1.1:1 mixture of diastereomers (37.5 mg, 55%). Spectra matched previous report.[3b] (^{1}H NMR, ^{13}C{^{1}H} NMR, and IR included for characterization agreement and assessment of purity.) ***R_f*** = 0.75 (25% EtOAc–hexanes); **^{1}H NMR** ($CDCl_3$, 400 MHz): δ 7.47–7.41 (m, 4H [2H d1, 2H d2]), 7.37 (t, *J* = 7.2 Hz, 4H [2H d1, 2H d2]), 7.31 (t, *J* = 7.1 Hz, 2H [1H d1, 1H d2]), 6.71 (d, *J* = 15.8 Hz, 2H [1H d1, 1H d2]), 6.17 (dd, *J* = 15.6, 8.6 Hz, 1H d1) 6.15 (dd, *J* = 15.7, 8.7 Hz, 1H d2), 4.08 (ddd, *J* = 8.4, 4.5, 0.9 Hz, 1H d2), 4.01 (ddd, *J* = 8.6, 6.2, 0.9 Hz, 1H d1), 3.64 (qd, *J* = 6.7, 4.5 Hz, 1H d2), 3.54 (p, *J* = 6.6 Hz, 1H d1), 1.32 (d, *J* = 6.7 Hz, 3H d2), 1.29 (d, *J* = 6.7 Hz, 3H d1); **^{13}C{^{1}H} NMR** ($CDCl_3$, 100 MHz): δ 136.4, 135.9, 135.4, 135.4, 128.7 (2C), 128.7 (2C), 128.6, 128.6, 126.8 (2C), 126.8 (2C), 122.8, 122.1, 68.8, 68.3, 60.0, 60.0, 16.3, 15.6; **FTIR** (thin film): cm^{-1} 2980, 2085, 1248.

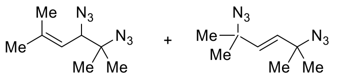

3m **3m'**

4,5-Diazido-2,5-dimethylhex-2-ene (3m) and (*E*)-2,5-Diazido-2,5-dimethylhex-3-ene (3m'). Synthesized using Standard Condition I. A ratio of 1.4:1 (**3m**:**3m'**) was determined by ^{1}H NMR of the crude reaction. Isolated by flash column chromatography (silica gel, 100% hexanes to 5% ethyl acetate–hexanes) as a clear oil and an inseparable 1.3:1 mixture of **3m**:**3m'** (52.4 mg, 90%). ***R$_f$*** = 0.82 (25% EtOAc–hexanes); **^{1}H NMR** ($CDCl_3$, 400 MHz): δ 5.67 (s, 2H, **3m'**), 5.24 (dp [app], *J* = 9.9, 1.4 Hz, 1H, **3m**), 4.06 (d, *J* = 9.8 Hz, 1H, **3m**), 1.85 (d, *J* = 1.4 Hz, 3H, **3m**), 1.74 (d, *J* = 1.5 Hz, 3H, **3m**), 1.36 (s, 6H, **3m'**), 1.28 (s, 3H, **3m**), 1.22 (s, 3H, **3m**); **^{13}C{^{1}H} NMR** ($CDCl_3$, 100 MHz): δ 141.1 (**3m'**), 132.6 (**3m**), 117.8 (**3m**), 66.8 (**3m'**), 63.4 (**3m**), 61.4 (**3m**), 26.6 (**3m'**), 25.9 (**3m**), 23.3, (**3m**), 22.7 (**3m**), 18.6 (**3m**); **FTIR** (thin film): cm^{-1} 2977, 2937, 2092, 1249, 1138; **HRMS** (ESI^{+}, m/z): Calcd for $C_{16}H_{29}N_{12}^{+}$ ([2M+H]$^{+}$): 389.2633; found: 389.2643.

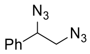 **3n**

(1,2-Diazidoethyl)benzene (3n). Synthesized using Standard Condition I. Isolated by flash column chromatography (silica gel, 100% hexanes to 5% ethyl acetate–hexanes) as a clear oil (39.4 mg, 70%). Spectra matched previous reports.[4] (^{1}H NMR, ^{13}C{^{1}H} NMR, and IR included for characterization agreement and assessment of purity.) **^{1}H NMR** ($CDCl_3$, 400 MHz): δ 7.46–7.38 (m, 3H), 7.34 (dd, *J* = 7.9, 1.8 Hz, 2H), 4.68 (dd, *J* = 8.4, 4.9 Hz, 1H), 3.51 (dd, *J* = 12.8, 8.4 Hz, 1H), 3.44 (dd, *J* = 12.7, 4.9 Hz, 1H); **^{13}C{^{1}H} NMR** ($CDCl_3$, 100 MHz): δ 136.2, 129.1 (2C), 129.0, 126.9 (2C), 65.5, 55.9; **FTIR** (thin film): cm^{-1} 2924, 2089, 1248, 759, 699.

4b. Two-Component Diene 1,2-Azidooxygenation

Standard Condition II: Two-component 1,2-azidooxygenation of 1,3-dienes

To a 1-dram vial equipped with a Teflon-coated stir bar was added copper(II) trifluoromethanesulfonate (10.9 mg, 0.03 mmol, 0.1 equiv). 1,2-Dichloroethane (1.5 mL) was added, followed by sequential addition of the 1,3-diene **1** (0.9 mmol, 3.0 equiv) and Zhdankin's reagent **2** (86.7 mg, 0.3 mmol, 1.0 equiv). The vial was capped and stirred at 60 °C in an aluminum heating block for 1 h. The resulting crude mixture was filtered through activated, neutral Al_2O_3 (Brockman Grade I, 58–60 Å mesh powder) and concentrated *in vacuo* to yield the crude product. Purification was performed as noted below.

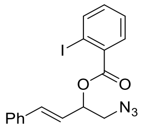 **4a**

(*E*)-1-Azido-4-phenylbut-3-en-2-yl 2-iodobenzoate (4a). Synthesized using Standard Condition II. Isolated by flash column chromatography (silica gel, 100% hexanes to 10% ethyl acetate–hexanes) as a clear oil (37.7 mg, 30%). ***R$_f$*** = 0.68 (25% EtOAc–hexanes); **^{1}H NMR** ($CDCl_3$, 400 MHz): δ 8.02 (d, *J* = 7.9 Hz, 1H), 7.89 (d, *J* = 7.8 Hz, 1H), 7.46–7.39 (m, 3H), 7.37–7.27 (m, 3H), 7.18 (t, *J* = 7.7 Hz, 1H), 6.85 (d, *J* = 16.0 Hz, 1H), 6.29 (dd, *J* = 16.0, 7.4 Hz, 1H), 5.83 (dt, *J* = 6.5, 5.8 Hz, 1H), 3.71–3.61 (m, 2H); **^{13}C{^{1}H} NMR** ($CDCl_3$, 100 MHz): δ 165.3, 141.5, 135.6, 135.3, 134.3, 132.9, 131.1, 128.6 (2C), 128.5, 128.0, 126.8 (2C), 123.0, 94.3, 74.6, 53.9; **FTIR** (thin film): cm^{-1} 3059, 3026, 2926, 2097, 1727, 1582, 1493, 1449, 1430, 1283, 1241, 1131, 1096, 1014, 965, 740, 693; **HRMS** (ESI^{+}, m/z): Calcd for $C_{17}H_{14}IN_3O_2Na^{+}$ ([M+Na]$^{+}$): 442.0023; found: 442.0012.

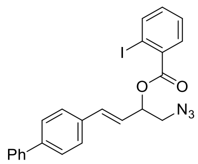

4b

(*E*)-4-([1,1'-Biphenyl]-4-yl)-1-azidobut-3-en-2-yl 2-iodobenzoate (4b). Synthesized using Standard Condition II. Isolated by flash column chromatography (silica gel, 100% hexanes to 15% ethyl acetate–hexanes) as a clear oil (28.8 mg, 19%). R_f = 0.63 (25% EtOAc–hexanes); **^{1}H NMR** ($CDCl_3$, 400 MHz): δ 8.02 (d, *J* = 8.03 Hz, 1H), 7.90 (d, *J* = 8.4 Hz, 1H), 7.63–7.55 (m, 4H), 7.51–7.42 (m, 5H), 7.38–7.34 (m, 1H), 7.19 (t, *J* = 7.6 Hz, 1H), 6.89 (d, *J* = 15.9 Hz, 1H), 6.33 (dd, *J* = 15.9, 7.4 Hz, 1H), 5.88–5.82 (m, 1H), 3.70–3.64 (m, 2H); **^{13}C{^{1}H} NMR** ($CDCl_3$, 100 MHz): δ 165.3, 141.5, 141.2, 140.4, 134.9, 134.6, 134.3, 133.0, 131.1, 128.8 (2C), 128.0, 127.5, 127.3 (2C), 127.2 (2C), 126.9 (2C), 123.0, 94.3, 74.7, 54.0; **FTIR** (thin film): cm^{-1} 3028, 2928, 2098, 1728, 1582, 1486, 1285, 1246, 1131, 1097, 1015, 968, 762, 739, 697; **HRMS** (ESI$^+$, m/z): Calcd for $C_{23}H_{18}IN_3O_2Na^+$ ($[M+Na]^+$): 518.0336; found: 518.0323.

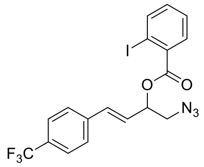

4c

(*E*)-1-Azido-4-(4-(trifluoromethyl)phenyl)but-3-en-2-yl 2-iodobenzoate (4c). Synthesized using Standard Condition II. A ratio of >20:1 for **4c**:**4c'** was determined by ^{1}H NMR of the crude reaction. Isolated by flash column chromatography (silica gel, 100% hexanes to 20% ethyl acetate–hexanes) as a clear oil and an inseparable 12.9:1 mixture of **4c** and **4c'** (79.9 mg, 55%). R_f = 0.62 (25% EtOAc–hexanes); **^{1}H NMR** ($CDCl_3$, 400 MHz): δ 8.01 (d, *J* = 7.9 Hz, 1H), 7.89 (dd, *J* = 7.9, 1.7 Hz, 1H), 7.57 (d, *J* = 8.2 Hz, 2H), 7.50 (d, *J* = 8.2 Hz, 2H), 7.43 (t, *J* = 7.6 Hz, 1H), 7.18 (td, *J* = 7.7, 1.7 Hz, 1H), 6.86 (d, *J* = 16.0 Hz, 1H), 6.37 (dd, *J* = 16.0, 7.1 Hz, 1H), 5.85–5.79 (m, 1H), 3.69 (dd, *J* = 13.0, 6.0 Hz, 1H), 3.64 (dd, *J* = 13.3, 4.7 Hz, 1H); **^{13}C{^{1}H} NMR** ($CDCl_3$, 100 MHz): δ 165.2, 134.1, 133.6, 133.1, 131.1, 130.1 (q, $^2J_{C\text{–}F}$ = 32.6 Hz, 1C), 128.0, 126.9 (2C), 125.8, 125.6 (q, $^3J_{C\text{–}F}$ = 3.9 Hz, 2C), 124.0 (q, $^1J_{C\text{–}F}$ = 272.0 Hz, 1C), 94.3, 74.2, 53.8; **^{19}F NMR** ($CDCl_3$, 376.5 MHz): δ –63.3 (s, 3F); **FTIR** (thin film): cm^{-1} 2917, 2099, 1729, 1323, 1284, 1244, 1164, 1120, 1066, 1015, 968, 740; **HRMS** (ESI$^+$, m/z): Calcd for $C_{18}H_{13}F_3IN_3O_2Na^+$ ($[M+Na]^+$): 509.9897; found: 509.9886.

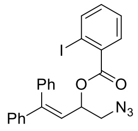

4f

1-Azido-4,4-diphenylbut-3-en-2-yl 2-iodobenzoate (4f). Synthesized using Standard Condition II. Isolated by flash column chromatography (silica gel, 100% hexanes to 10% ethyl acetate–hexanes) as a clear oil (36.6 mg, 25%). R_f = 0.68 (25% EtOAc–hexanes); **^{1}H NMR** ($CDCl_3$, 400 MHz): δ 7.97 (d, *J* = 7.9 Hz, 1H), 7.78 (d, *J* = 7.6 Hz, 1H), 7.45–7.34 (m, 4H), 7.31–7.21 (m, 7H), 7.14 (t, *J* = 7.8 Hz, 1H), 6.20 (d, *J* = 9.1 Hz, 1H), 5.76–5.68 (m, 1H), 3.60–3.49 (m, 2H); **^{13}C{^{1}H} NMR** ($CDCl_3$, 100 MHz): δ 165.9, 147.4, 141.2, 140.8, 138.3, 134.9, 132.7, 131.0, 129.3 (2C), 128.6 (2C), 128.3 (2C), 128.2, 128.0, 127.9, 127.5 (2C), 94.0, 72.8, 54.1; **FTIR** (thin film): cm^{-1} 3056, 3024, 2923, 2097, 1728, 1582, 1494, 1445, 1281, 1245, 1129, 1097, 1015, 764, 741, 699; **HRMS** (ESI$^+$, m/z): Calcd for $C_{23}H_{18}IN_3O_2Na^+$ ($[M+Na]^+$): 518.0336; found: 518.0324.

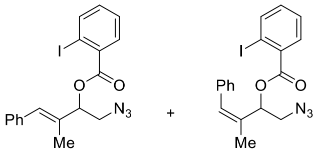

4g (*E*) **4g'** (*Z*) **4g**

(*E*)-1-azido-3-methyl-4-phenylbut-3-en-2-yl 2-iodobenzoate (4g) and (*Z*)-1-azido-3-methyl-4-phenylbut-3-en-2-yl 2-iodobenzoate (4g'). Synthesized using Standard Condition II. A ratio of 5.1:1 (*E*:*Z*) was determined by ^{1}H NMR of the crude reaction. Isolated by flash column chromatography (silica gel, 100% hexanes to 10% ethyl acetate–hexanes) as a clear oil and an inseparable 4.7:1 mixture of *E*:*Z* (46.5 mg, 36%). R_f = 0.64 (25% EtOAc–hexanes); **^{1}H NMR** ($CDCl_3$, 400 MHz): δ 7.99 (d, *J* = 8.1 Hz, 1H, *E*), 7.95 (d, *J* = 7.8 Hz, 1H, *Z*), 7.90 (d, *J* = 7.8 Hz, 1H, *E*), 7.87–7.84 (m, 1H, *Z*), 7.44–7.19 (m, 6H each, *E*+*Z*), 7.18–7.10 (m, 1H each, *E*+*Z*), 6.71 (s, 1H, *E*), 6.42 (s, 1H, *Z*), 5.86 (t, *J* = 7.5 Hz, 1H, *Z*), 5.64 (dd, *J* = 7.4, 4.8 Hz, 1H, *E*), 3.86–3.82 (m, 2H, *Z*), 3.68 (dd, *J* = 13.0, 7.6 Hz, 1H, *E*), 3.57 (dd, *J* = 13.1, 4.6 Hz, 1H, *E*), 1.96 (s, 3H, *E*), 1.67 (s, 3H, *Z*); **^{13}C{^{1}H} NMR** ($CDCl_3$, 100 MHz): δ 165.2 (*E*), 165.0 (*Z*), 141.5 (*E*), 139.5 (*Z*), 137.4 (*Z*), 136.4 (*E*), 134.5 (*Z*), 134.4 (*E*), 141.5 (*E*), 139.5 (*Z*), 137.4 (*Z*), 136.4 (*E*), 134.5 (*Z*), 134.4 (*E*), 132.9 (*E*), 132.9 (*Z*), 132.5 (*E*), 131.0 (*E*), 129.6 (*E*), 129.0 (2C, *E*), 128.5 (*Z*), 128.3 (*Z*), 128.2 (2C, *E*), 128.0 (*E*), 128.0 (*Z*), 127.1 (*Z*), 127.1 (*E*), 120.6 (*Z*), 94.2 (*E*), 80.2 (*Z*), 78.7 (*E*), 52.6 (*E*), 47.6 (*Z*), 14.5 (*E*), 13.6 (*Z*) (note that not all *Z* peaks could be accounted for as some likely overlapped the much larger *E* peaks); **FTIR** (thin film): cm^{-1} 3057, 2918, 2097, 1731, 1583, 1431, 1284, 1246, 1132, 1097, 1015, 741, 699;

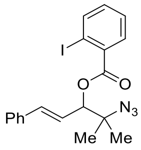

4i

(*E*)-4-Azido-4-methyl-1-phenylpent-1-en-3-yl 2-iodobenzoate (4i). Synthesized using Standard Condition II. Isolated by flash column chromatography (silica gel, 100% hexanes to 10% ethyl acetate–hexanes) as a clear oil (22.6 mg, 17%). R_f = 0.78 (25% EtOAc–hexanes); **^{1}H NMR** ($CDCl_3$, 400 MHz): δ 7.99 (d, *J* = 8.0 Hz, 1H), 7.87 (dd, *J* = 7.9, 1.8 Hz, 1H), 7.44–7.37 (m, 3H), 7.34–7.22 (m, 3H), 7.15 (td, *J* = 7.7, 1.7 Hz, 1H), 6.79 (td, *J* = 15.9 Hz, 1H), 6.27 (dd, *J* = 15.9, 8.1 Hz, 1H), 5.55 (d, *J* = 8.2 Hz, 1H), 1.41 (s, 3H), 1.36 (s, 3H); **^{13}C{^{1}H} NMR** ($CDCl_3$, 100 MHz): δ 165.1, 141.6, 136.5, 135.8, 134.3, 132.9, 131.0, 128.6 (2C), 128.4, 128.0, 126.8 (2C), 122.3, 94.4, 81.2, 63.1, 23.3, 23.1; **FTIR** (thin film): cm^{-1} 3027, 2979, 2107, 1732, 1248, 1132, 1096, 1015, 742; **HRMS** (ESI$^+$, m/z): Calcd for $C_{19}H_{18}IN_3O_2Na^+$ ([M+Na]$^+$): 470.0336; found: 470.0326.

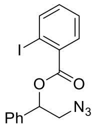

4k

2-Azido-1-phenylethyl 2-iodobenzoate (4k). Synthesized using Standard Condition II. Isolated by flash column chromatography (silica gel, 100% hexanes to 10% ethyl acetate–hexanes) as a clear oil (36.8 mg, 31%). Spectra matched previous report.[4a] (^{1}H NMR included for characterization agreement and assessment of purity.) **^{1}H NMR** ($CDCl_3$, 400 MHz): δ 8.01 (d, *J* = 7.9, 1.2 Hz, 1H), 7.93 (d, *J* = 7.8, 1.7 Hz, 1H), 7.49–7.33 (m, 6H), 7.18 (td, *J* = 7.7, 1.8 Hz, 1H), 6.15 (dd, *J* = 7.8, 4.2 Hz, 1H), 3.80 (dd, *J* = 13.1, 7.8 Hz, 1H), 3.64 (dd, *J* = 13.1, 4.2 Hz, 1H).

4c. Three-Component Diene 1,2-Azidooxygenation

Standard Condition III: Three-component 1,2-azidooxygenation of 1,3-dienes

To a 2-dram vial equipped with a Teflon-coated stir bar was added copper(II) acetate (10.9 mg, 0.06 mmol, 0.2 equiv) and the carboxylic acid **5** (0.9 mmol, 3.0 equiv). Acetonitrile (1.5 mL) was added, followed by sequential addition of the 1,3-diene **1** (0.9 mmol, 3.0 equiv) and Zhdankin's reagent **2** (86.7 mg, 0.3 mmol, 1.0 equiv). The vial was capped and stirred at 60 °C in an aluminum heating block for 5 h. The resulting crude mixture was filtered through activated, neutral Al_2O_3 (Brockman Grade I, 58–60 Å mesh powder) and concentrated *in vacuo* to yield the crude product. Purification was performed as noted below.

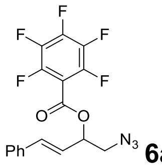

6a

(*E*)-1-Azido-4-phenylbut-3-en-2-yl 2,3,4,5,6-pentafluorobenzoate (6a). Synthesized using Standard Condition III. Isolated by flash column chromatography (silica gel, 100% hexanes to 5% ethyl acetate–hexanes) as a clear oil (53.9 mg, 47%). $\boldsymbol{R_f}$ = 0.71 (25% EtOAc–hexanes); **^{1}H NMR** ($CDCl_3$, 400 MHz): δ 7.42 (d, *J* = 7.7 Hz, 2H), 7.36 (t, *J* = 7.4 Hz, 2H), 7.33–7.28 (m, 1H), 6.85 (d, *J* = 16.0 Hz, 1H), 6.22 (dd, *J* = 16.0, 7.5 Hz, 1H), 5.87–5.80 (m, 1H), 3.68–3.59 (m, 2H); **^{13}C{^{1}H} NMR** ($CDCl_3$, 100 MHz): δ 158.1, 145.6 (d, J_{C-F} = 259.1 Hz, 2C), 143.4 (d, J_{C-F} = 260.1 Hz, 1C), 137.7 (d, J_{C-F} = 256.2 Hz, 2C), 136.1, 135.3, 128.7, 128.7 (2C), 126.8 (2C), 122.0, 107.7 (td, J_{C-F} = 15.4, 4.0 Hz, 1C), 75.8, 53.8; **^{19}F NMR** ($CDCl_3$, 376.5 MHz): δ –138.1 (ddt, *J* = 25.2, 6.0, 5.7 Hz, 2F), –148.3 (tt, *J* = 20.9, 4.6 Hz, 1F), –160.5 (ddt, *J* = 26.4, 20.1, 6.0 Hz, 2F); **FTIR** (thin film): cm^{-1} 2932, 2100, 1736, 1652, 1524, 1494, 1326, 1215, 996, 968, 937, 748, 693; **HRMS** (ESI$^+$, m/z): Calcd for $C_{17}H_{10}F_5N_3O_2Na^+$ ([M+Na]$^+$): 406.0585; found: 406.0583.

<u>1.0 mmol scale</u>: To a 2-dram vial equipped with a Teflon-coated stir bar was added copper(II) acetate (36.3 mg, 0.2 mmol, 0.2 equiv) and pentafluorobenzoic acid **5a** (636.2 mg, 3.0 mmol, 3.0 equiv). Acetonitrile (5.0 mL) was added, followed by sequential addition of 1-phenyl-1,3-butadiene **1a** (390.6 mg, 3.0 mmol, 3.0 equiv) and Zhdankin's reagent **2** (289.3 mg, 1.0 mmol, 1.0 equiv). The vial was capped and stirred at 60 °C in an aluminum heating block for 5 h. The resulting crude mixture was filtered through activated, neutral Al_2O_3 (Brockman Grade I, 58–60 Å mesh powder) and concentrated *in vacuo* to yield the crude product. Isolated by flash column chromatography (silica gel, 100% hexanes to 10% ethyl acetate–hexanes) as a clear oil (179.8 mg, 47%).

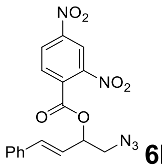

6b

(*E*)-1-Azido-4-phenylbut-3-en-2-yl 2,4-dinitrobenzoate (6b). Synthesized using Standard Condition III. Isolated by flash column chromatography (silica gel, 100% hexanes to 20% ethyl acetate–hexanes) as a clear oil (59.8 mg, 52%). $\boldsymbol{R_f}$ = 0.47 (25% EtOAc–hexanes); **^{1}H NMR** ($CDCl_3$, 400 MHz): δ 8.79 (d, *J* = 2.1 Hz, 1H), 8.51 (dd, *J* = 8.5, 2.1 Hz, 1H), 7.94 (d, *J* = 8.4 Hz, 1H), 7.42 (d, *J* = 7.5 Hz, 2H), 7.37–7.27 (m, 3H), 6.84 (d, *J* = 16.0 Hz, 1H), 6.20 (dd, *J* = 15.9, 7.8 Hz, 1H), 5.87–5.79 (m, 1H), 3.67–3.57 (m, 2H); **^{13}C{^{1}H} NMR** ($CDCl_3$, 100 MHz): δ 162.8, 148.9, 147.7, 136.4, 135.2, 132.6, 131.1, 128.7, 128.7 (2C), 127.7, 126.8 (2C), 121.6, 119.6, 76.6, 53.5; **FTIR** (thin film): cm^{-1} 3105, 2925, 2099, 1736, 1603, 1534, 1345, 1274, 1242, 1109, 1056, 967, 833, 750, 733, 693; **HRMS** (APCI$^-$, m/z): Calcd for $C_{17}H_{13}ClN_5O_6^-$ ([M+Cl]$^-$): 418.0560; found: 418.0560.

1.0 mmol scale: To a 2-dram vial equipped with a Teflon-coated stir bar was added copper(II) acetate (36.3 mg, 0.2 mmol, 0.2 equiv) and 2,4-dinitrobenzoic acid **5b** (636.4 mg, 3.0 mmol, 3.0 equiv). Acetonitrile (5.0 mL) was added, followed by sequential addition of 1-phenyl-1,3-butadiene **1a** (390.6 mg, 3.0 mmol, 3.0 equiv) and Zhdankin's reagent **2** (289.3 mg, 1.0 mmol, 1.0 equiv). The vial was capped and stirred at 60 °C in an aluminum heating block for 5 h. The resulting crude mixture was filtered through activated, neutral Al_2O_3 (Brockman Grade I, 58–60 Å mesh powder) and concentrated *in vacuo* to yield the crude product. Isolated by flash column chromatography (silica gel, 100% hexanes to 30% ethyl acetate–hexanes) as a clear oil (179.9 mg, 47%).

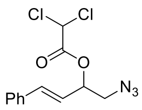

6e

(*E*)-1-Azido-4-phenylbut-3-en-2-yl 2,2-dichloroacetate (6e). Synthesized using Standard Condition III. Isolated by flash column chromatography (silica gel, 100% hexanes to 5% ethyl acetate–hexanes) as a clear oil (44.5 mg, 49%). ***R_f*** = 0.67 (25% EtOAc–hexanes); **^{1}H NMR** ($CDCl_3$, 400 MHz): δ 7.41 (dd, *J* = 8.1, 1.7 Hz, 2H), 7.38–7.28 (m, 3H), 6.81 (d, *J* = 15.9 Hz, 1H), 6.16 (dd, *J* = 15.9, 7.5 Hz, 1H), 5.63 (ddd, *J* = 7.4, 6.1. 4.7 Hz, 1H), 3.61 (dd, *J* = 13.4, 6.4 Hz, 1H), 3.57 (dd, *J* = 13.2, 4.6 Hz, 1H); **^{13}C{^{1}H} NMR** ($CDCl_3$, 100 MHz): δ 163.6, 136.4, 135.3, 129.0, 128.9 (2C), 127.0 (2C), 121.5, 77.1, 64.3, 53.8; **FTIR** (thin film): cm^{-1} 3026, 2099, 1761, 1494, 1450, 1267, 1162, 966, 749, 692; **HRMS** (ESI$^+$, m/z): Calcd for $C_{12}H_{11}Cl_2N_3O_2Li^+$ ([M+Li^7]$^+$): 306.0383; found: 306.0392.

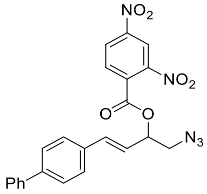

7b

(*E*)-4-([1,1'-Biphenyl]-4-yl)-1-azidobut-3-en-2-yl 2,4-dinitrobenzoate (7b). Synthesized using Standard Condition III. Isolated by flash column chromatography (silica gel, 100% hexanes to 20% ethyl acetate–hexanes) as a light yellow solid (70.6 mg, 51%). ***R_f*** = 0.38 (25% EtOAc–hexanes); **^{1}H NMR** ($CDCl_3$, 400 MHz): δ 8.84 (d, *J* = 2.3 Hz, 1H), 8.54 (dd, *J* = 8.4, 2.2 Hz, 1H), 7.96 (d, *J* = 8.5 Hz, 1H), 7.62–7.57 (m, 4H), 7.51 (d, *J* = 8.2 Hz, 2H), 7.46 (t, *J* = 7.6 Hz, 2H), 7.37 (t, *J* = 7.2 Hz, 1H), 6.90 (d, *J* = 15.9 Hz, 1H), 6.26 (dd, *J* = 15.9, 7.9 Hz, 1H), 5.89–5.83 (m, 1H), 3.67 (dd, *J* = 13.4, 4.7 Hz, 1H), 3.64 (dd, *J* = 13.7, 6.0 Hz, 1H); **^{13}C{^{1}H} NMR** ($CDCl_3$, 100 MHz): δ 162.9, 148.9, 147.7, 141.5, 140.3, 136.2, 134.2, 132.7, 131.1, 128.8 (2C), 127.7, 127.6, 127.4 (2C), 127.3 (2C), 126.8 (2C), 121.5, 119.7, 76.7, 53.5; **FTIR** (thin film): cm^{-1} 3032, 2928, 2102, 1736, 1602, 1536, 1485, 1345, 1273, 1241, 1107, 1056, 1006, 968, 905, 833, 762, 725, 697; **HRMS** (APCI$^-$, m/z): Calcd for $C_{23}H_{17}ClN_5O_6^-$ ([M+Cl]$^-$): 494.0873; found: 494.0874.

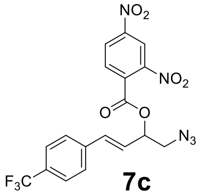

7c

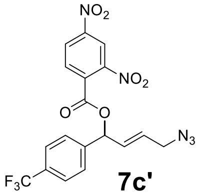

7c'

(*E*)-1-Azido-4-(4-(trifluoromethyl)phenyl)but-3-en-2-yl 2,4-dinitrobenzoate (7c) and (*E*)-4-Azido-1-(4-(trifluoromethyl)phenyl)but-2-en-1-yl 2,4-dinitrobenzoate (7c'). Synthesized using Standard Condition III. A ratio of 1.2:1 for **7c**:**7c'** was determined by ^{1}H NMR of the crude reaction. Isolated by flash column chromatography (silica gel, 100% hexanes to 20% ethyl acetate–hexanes) as a clear oil and an inseparable 2.2:1 mixture of **7c** and **7c'** (58.4 mg, 43%). ***R_f*** = 0.47 (25% EtOAc–hexanes); **^{1}H NMR** ($CDCl_3$, 400 MHz): δ 8.85 (d, *J* = 2.2 Hz, 1H, **7c**), 8.80 (d, *J* = 2.2 Hz, 1H, **7c'**), 8.56 (dd, *J* = 8.5, 2.2 Hz, 1H, **7c**), 8.53 (dd, *J* =

8.6, 2.2 Hz, 1H, **7c'**), 7.97 (d, *J* = 8.4 Hz, 1H, **7c**), 7.93 (d, *J* = 8.4 Hz, 1H, **7c'**), 7.67 (d, *J* = 8.1 Hz, 2H, **7c'**), 7.60 (d, *J* = 8.2 Hz, 2H, **7c**), 7.53 (d, *J* = 7.9 Hz, 2H, **7c**), 7.51 (d, *J* = 7.7 Hz, 2H, **7c'**), 6.88 (d, *J* = 16.0 Hz, 1H, **7c**), 6.58 (d, *J* = 6.4 Hz, 1H, **7c'**), 6.31 (dd, *J* = 16.0, 7.6 Hz, 1H, **7c**), 6.02 (ddt, *J* = 15.3, 6.3, 1.3 Hz, 1H, **7c'**), 5.91 (dtd, *J* = 15.4, 5.7, 1.0 Hz, 1H, **7c'**), 5.88–5.82 (m, 1H), 3.89 (d, *J* = 5.9 Hz, 2H, **7c'**), 3.68 (dd, *J* = 13.3, 4.6 Hz, 1H, **7c**), 3.64 (dd, *J* = 13.3, 6.0 Hz, 1H, **7c**); **^{13}C{^{1}H} NMR** ($CDCl_3$, 100 MHz): δ 162.9 (**7c**), 162.6 (**7c'**), 149.0 (**7c'**), 149.0 (**7c**), 147.9 (**7c'**), 147.7 (**7c**), 140.8 (q, $^4J_{C\text{–}F}$ = 1.1 Hz, 2C, **7c'**), 138.7 (q, $^4J_{C\text{–}F}$ = 1.4 Hz, 2C, **7c**), 134.9 (**7c**), 132.5 (**7c**), 132.2 (**7c'**), 131.3 (**7c'**), 131.2 (**7c**), 131.0 (q, $^2J_{C\text{–}F}$ = 32.7 Hz, **7c'**), 130.4 (q, $^2J_{C\text{–}F}$ = 32.6 Hz, **7c**), 130.1 (**7c'**), 129.2 (**7c'**), 127.8 (**7c**), 127.6 (**7c'**), 127.5 (**7c'**), 127.1 (**7c**, 2C), 125.9 (q, $^3J_{C\text{–}F}$ = 3.8 Hz, 2C, **7c'**), 125.7 (q, $^3J_{C\text{–}F}$ = 3.7 Hz, 2C, **7c**), 124.3 (**7c'**), 124.0 (q, $^1J_{C\text{–}F}$ = 273.0 Hz, **7c**), 123.8 (q, $^1J_{C\text{–}F}$ = 273.0 Hz, **7c'**), 119.8 (**7c**), 119.7 (**7c'**), 77.7 (**7c'**), 76.2 (**7c**), 53.3 (**7c**), 51.6 (**7c'**); **^{19}F NMR** ($CDCl_3$, 376.5 MHz): δ -63.3 (**7c'**, s, 3F), –63.4 (**7c**, s, 3F); **FTIR** (thin film): cm^{-1} 3108, 2927, 2101, 1738, 1604, 1537, 1347, 1322, 1273, 1243, 1164, 1107, 1065, 833, 732; **HRMS** (ESI^+, m/z): Calcd for $C_{18}H_{12}F_3N_5O_6Cl^-$ ($[M+Cl]^-$): 486.0434; found: 486.0419.

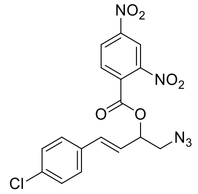

7d

(E)-1-Azido-4-(4-chlorophenyl)but-3-en-2-yl 2,4-dinitrobenzoate (7d). Synthesized using Standard Condition III. Isolated by flash column chromatography (silica gel, 100% hexanes to 20% ethyl acetate–hexanes) as a clear oil (59.9 mg, 48%). ***R$_f$*** = 0.44 (25% EtOAc–hexanes); **^{1}H NMR** ($CDCl_3$, 400 MHz): δ 8.83 (d, *J* = 2.1 Hz, 1H), 8.55 (dd, *J* = 8.4, 2.1 Hz, 1H), 7.96 (d, *J* = 8.4 Hz, 1H), 7.36 (d, *J* = 8.6 Hz, 2H), 7.31 (d, *J* =8.5 Hz, 2H), 6.80 (d, *J* = 15.9 Hz, 1H), 6.18 (d, *J* = 16.0, 7.8 Hz, 1H), 5.85–5.78 (m, 1H), 3.65 (dd, *J* = 13.2, 4.5 Hz, 1H), 3.62 (dd, *J* = 13.2, 6.0 Hz, 1H); **^{13}C{^{1}H} NMR** ($CDCl_3$, 100 MHz): δ 162.9, 149.0, 147.8, 135.3, 134.6, 133.8, 132.6, 131.2, 128.9 (2C), 128.1 (2C), 127.7, 122.3, 119.7, 76.4, 53.5; **FTIR** (thin film): cm^{-1} 3105, 2926, 2099, 1736, 1603, 1534, 1491, 1345, 1273, 1242, 1108, 1056, 967, 907, 833, 730; **HRMS** ($APCI^-$, m/z): Calcd for $C_{17}H_{12}Cl_2N_5O_6^-$ ($[M+Cl]^-$): 452.0170; found: 452.0171.

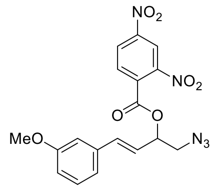

7f

(*E*)-1-Azido-4-(3-methoxyphenyl)but-3-en-2-yl 2,4-dinitrobenzoate (7f). Synthesized using Standard Condition III. Isolated by flash column chromatography (silica gel, 100% hexanes to 20% ethyl acetate–hexanes) as a clear oil (59.6 mg, 48%). ***R$_f$*** = 0.33 (25% EtOAc–hexanes); **^{1}H NMR** ($CDCl_3$, 400 MHz): δ 8.74 (d, *J* = 2.2 Hz, 1H), 8.45 (dd, *J* = 8.4, 2.2 Hz, 1H), 7.87 (d, *J* = 8.4 Hz, 1H), 7.18 (t, *J* = 7.9 Hz, 1H), 6.93 (d, *J* = 7.8 Hz, 1H), 6.86 (t, *J* = 2.0 Hz, 1H), 6.77 (dd, *J* = 8.2, 2.5 Hz, 1H), 6.73 (d, *J* = 16.1 Hz, 1H), 6.11 (dd, *J* = 16.0, 7.8 Hz, 1H), 5.77–5.71 (m, 1H), 3.74 (s, 3H), 3.57 (dd, *J* = 13.4, 4.6 Hz, 1H), 3.53 (dd, *J* = 13.3, 6.0 Hz, 1H); **^{13}C{^{1}H} NMR** ($CDCl_3$, 100 MHz): δ 162.9, 159.8, 148.9, 147.7, 136.6, 136.4, 132.6, 136.4, 132.6, 131.1, 129.7, 127.7, 121.9, 119.7, 119.5, 114.4, 112.1, 76.6, 55.2, 53.5; **FTIR** (thin film): cm^{-1} 2931, 2100, 1738, 1597, 1537, 1514, 1464, 1346, 1243, 1108, 907, 833, 727; **HRMS** ($APCI^-$, m/z): Calcd for $C_{18}H_{15}ClN_5O_7^+$ ($[M+Cl]^-$): 448.0665; found: 448.0667.

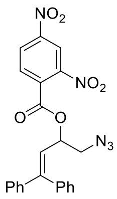
7i

1-Azido-4,4-diphenylbut-3-en-2-yl 2,4-dinitrobenzoate (7i). Synthesized using Standard Condition III. Isolated by flash column chromatography (silica gel, 100% hexanes to 20% ethyl acetate–hexanes) as a clear oil (59.8 mg, 43%). ***R_f*** = 0.56 (25% EtOAc–hexanes); **^{1}H NMR** ($CDCl_3$, 400 MHz): δ 8.78 (d, *J* = 1.9 Hz, 1H), 8.47 (dd, *J* = 8.5, 2.0 Hz, 1H), 7.83 (d, *J* = 8.4 Hz, 1H), 7.44–7.34 (m, 3H), 7.27–7.34 (m, 7H), 6.07 (d, *J* = 9.2 Hz, 1H), 5.70 (dt, *J* = 8.8, 5.3 Hz, 1H), 3.52 (d, *J* = 4.4 Hz, 2H); **^{13}C{^{1}H} NMR** ($CDCl_3$, 100 MHz): δ 162.6, 148.8, 148.7, 147.6, 140.4, 138.2, 132.8, 131.1, 129.2 (2C), 128.6 (2C), 128.4, 128.3 (2C), 128.1, 127.7, 127.5 (2C), 120.5, 119.6, 74.7, 53.7; **FTIR** (thin film): cm^{-1} 3056, 2922, 2099, 1738, 1603, 1536, 1493, 1445, 1345, 1274, 1242, 1108, 1056, 908, 833, 765, 730, 699; **HRMS** (APCI$^-$, m/z): Calcd for $C_{23}H_{17}ClN_5O_6^-$ ([M+Cl]$^-$): 494.0873; found: 494.0875.

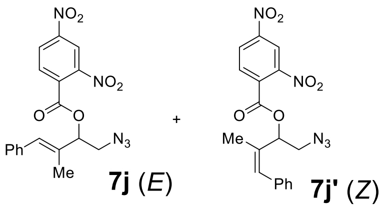
7j (*E*) + **7j'** (*Z*)

(*E*)-1-Azido-3-methyl-4-phenylbut-3-en-2-yl 2,4-dinitrobenzoate (7j) and (*Z*)-1-Azido-3-methyl-4-phenylbut-3-en-2-yl 2,4-dinitrobenzoate (7j'). Synthesized using Standard Condition III. A ratio of 1.2:1 (*E*:*Z*) was determined by ^{1}H NMR of the crude reaction. Isolated by flash column chromatography (silica gel, 100% hexanes to 20% ethyl acetate–hexanes) as a clear oil and an inseparable 1.5:1 mixture of *E*:*Z* (48.8 mg, 41%). ***R_f*** = 0.52 (25% EtOAc–hexanes); **^{1}H NMR** ($CDCl_3$, 400 MHz): δ 8.80 (s, 1H, *E*), 8.77 (s, 1H, *Z*), 8.57–8.47 (m, 1H each, *E*+*Z*), 7.98 (d, *J* = 8.4 Hz, 1H, *E*), 7.93 (d, *J* = 8.4 Hz, 1H, *Z*), 7.40–7.23 (m, 5H each, *E*+*Z*), 6.71 (s, 1H, *E*), 6.48 (s, 1H, *Z*), 5.82 (t, *J* = 7.3 Hz, 1H, *Z*), 5.71 (dd, *J* = 7.8, 4.9 Hz, 1H, *E*), 3.92–3.85 (m, 2H, *Z*), 3.69 (dd, *J* = 13.2, 7.8 Hz, 1H, *E*), 3.56 (dd, *J* = 13.2, 4.6 Hz, 1H, *E*), 1.93 (s, 3H, *E*), 1.66 (s, 3H, *Z*); **^{13}C{^{1}H} NMR** ($CDCl_3$, 100 MHz): δ 162.7 (*E*), 162.4 (*Z*), 149.0 (2C, *E*+*Z*), 148.0 (*Z*), 147.8 (*E*), 138.4 (*Z*), 136.3 (*Z*), 136.0 (*E*), 132.6 (*E*), 132.5 (*Z*), 131.3 (*Z*), 131.2 (2C, *E*), 131.1 (*E*), 129.0 (2C, *E*), 128.7 (*Z*), 128.7 (2C, *Z*) 128.2 (2C, *E*), 127.7 (*E*), 127.5 (*Z*), 127.3 (*E*), 126.9 (2C, *Z*), 121.7 (*Z*), 119.6 (*E*), 119.6 (*E*), 82.1 (*Z*), 80.7 (*E*), 52.2 (*E*), 47.5 (*Z*), 14.1 (*E*), 13.3 (*Z*); **FTIR** (thin film): cm^{-1} 3106, 2925, 2098, 1738, 1604, 1537, 1346, 1277, 1244, 1110, 1057, 834, 734, 700; **HRMS** (ESI$^-$, m/z): Calcd for $C_{18}H_{15}ClN_5O_6^-$ ([M+Cl]$^-$): 432.0716; found: 432.0716.

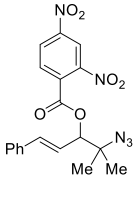
7l

(*E*)-4-Azido-4-methyl-1-phenylpent-1-en-3-yl 2,4-dinitrobenzoate (7l). Synthesized using Standard Condition III. Isolated by flash column chromatography (silica gel, 100% hexanes to 20% ethyl acetate–hexanes) as a clear oil (35.9 mg, 29%). ***R_f*** = 0.15 (10% EtOAc–hexanes); **^{1}H NMR** ($CDCl_3$, 400 MHz): δ 8.78 (s, 1H), 8.49 (d, *J* = 8.5 Hz, 1H), 7.96 (d, *J* = 8.2 Hz, 1H), 7.46 (d, *J* = 8.3 Hz, 2H), 7.36 (t, *J* = 7.2 Hz, 2H), 7.31 (t, *J* = 7.3 Hz, 1H), 6.85 (d, *J* = 15.9 Hz, 1H), 6.20 (dd, *J* = 15.8, 8.6 Hz, 1H), 5.56 (d, *J* = 8.6 Hz, 1H), 1.41 (s, 3H), 1.36 (s, 3H); **^{13}C{^{1}H} NMR** ($CDCl_3$, 100 MHz): δ 162.6, 148.9, 147.9, 137.9, 135.4, 132.5, 131.3, 128.7, 128.7 (2C), 127.5, 126.9 (2C), 120.8, 119.6, 83.4, 62.7, 23.3, 22.5; **FTIR** (thin film): cm^{-1}

3106, 2982, 2106, 1737, 1604, 1537, 1346, 1274, 1244, 1107, 1057, 834, 733, 693; **HRMS** (ESI$^-$, m/z): Calcd for $C_{19}H_{17}ClN_5O_6^-$ ([M+Cl]$^-$): 446.0873; found: 446.0872.

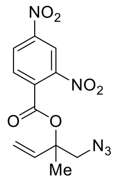

7m

1-Azido-2-methylbut-3-en-2-yl 2,4-dinitrobenzoate (7m). Synthesized using Standard Condition III. Only the indicated constitutional isomer was observed. Isolated by flash column chromatography (silica gel, 100% hexanes to 20% ethyl acetate–hexanes) as a clear oil (43.6 mg, 45%). R_f = 0.53 (25% EtOAc–hexanes); **^{1}H NMR** ($CDCl_3$, 400 MHz): δ 8.80 (s, 1H), 8.54 (d, *J* = 8.0 Hz, 1H), 7.92 (d, *J* = 8.2 Hz, 1H), 6.15 (dd, *J* = 17.5, 11.0 Hz, 1H), 5.39 (d, *J* = 17.6 Hz, 1H), 5.39 (d, *J* = 11.0 Hz, 1H), 3.65 (d, *J* = 12.6 Hz, 1H), 3.54 (d, *J* = 12.7 Hz, 1H), 1.75 (s, 3H); **^{13}C{^{1}H} NMR** ($CDCl_3$, 100 MHz): δ 162.1, 148.8, 147.6, 136.9, 133.3, 131.1, 127.7, 119.6, 117.2, 85.8, 58.0, 20.7; **FTIR** (thin film): cm^{-1} 3107, 2923, 2100, 1736, 1604, 1534, 1346, 1283, 1245, 1132, 1057, 907, 833, 729; **HRMS** (ESI$^-$, m/z): Calcd for $C_{12}H_{11}ClN_5O_6^-$ ([M+Cl]$^-$): 356.0403; found: 356.0402.

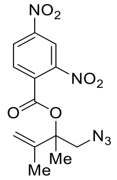

7n

1-Azido-2,3-dimethylbut-3-en-2-yl 2,4-dinitrobenzoate (7n). Synthesized using Standard Condition III. Isolated by flash column chromatography (silica gel, 100% hexanes to 15% ethyl acetate–hexanes) as a clear oil (43.2 mg, 43%). R_f = 0.58 (25% EtOAc–hexanes); **^{1}H NMR** ($CDCl_3$, 400 MHz): δ 8.79 (s, 1H), 8.54 (d, *J* = 8.4 Hz, 1H), 7.95 (d, *J* = 8.5 Hz, 1H), 5.14 (s, 1H), 5.10 (s, 1H), 3.68 (d, *J* = 12.9 Hz, 1H), 3.53 (d, *J* = 12.9 Hz, 1H), 1.87 (s, 3H), 1.82 (s, 3H); **^{13}C{^{1}H} NMR** ($CDCl_3$, 100 MHz): δ 161.6, 148.7, 147.8, 143.1, 133.0, 131.1, 127.5, 119.5, 114.0, 87.6, 57.4, 20.5, 19.0; **FTIR** (thin film): cm^{-1} 3105, 2926, 2099, 1736, 1604, 1535, 1346, 1282, 1245, 1095, 1057, 908, 833, 738; **HRMS** (ESI$^-$, m/z): Calcd for $C_{13}H_{13}ClN_5O_6^-$ ([M+Cl]$^-$): 370.0560; found: 370.0559.

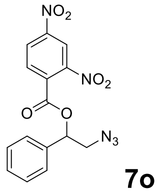

7o

2-Azido-1-phenylethyl 2,4-dinitrobenzoate (7o). Synthesized using Standard Condition III. Isolated by flash column chromatography (silica gel, 100% hexanes to 20% ethyl acetate–hexanes) as a clear oil (8.6 mg, 8%). R_f = 0.53 (25% EtOAc–hexanes); **^{1}H NMR** ($CDCl_3$, 400 MHz): δ 8.83 (d, *J* = 2.2 Hz, 1H), 8.55 (dd, *J* = 8.4, 2.2 Hz, 1H), 7.96 (d, *J* = 8.4 Hz, 1H), 7.46–7.37 (m, 5H), 6.16 (dd, *J* = 7.9, 4.2 Hz, 1H), 3.78 (dd, *J* = 13.3, 7.9 Hz, 1H), 3.63 (dd, *J* = 13.3, 4.3 Hz, 1H); **^{13}C{^{1}H} NMR** ($CDCl_3$, 100 MHz): δ 162.7, 149.1, 147.9, 135.3, 132.4, 131.3, 129.5, 129.0 (2C), 127.6, 126.8 (2C), 119.7, 77.5, 54.6; **FTIR** (thin film): cm^{-1} 3106, 2923, 2106, 1741, 1604, 1538, 1348, 1279, 1245, 1110, 1057, 834, 701; **HRMS** (ESI$^-$, m/z): Calcd for $C_{15}H_{11}ClN_5O_6^-$ ([M+Cl]$^-$): 392.0403; found: 392.0404.

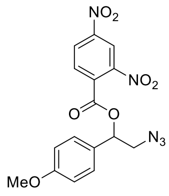

7p

2-Azido-1-(4-methoxyphenyl)ethyl 2,4-dinitrobenzoate (7p). Synthesized using Standard Condition III. Isolated by flash column chromatography (silica gel, 100% hexanes to 20% ethyl acetate–hexanes) as a clear oil (36.7 mg, 32%). $\boldsymbol{R_f}$ = 0.46 (25% EtOAc–hexanes); **^{1}H NMR** ($CDCl_3$, 400 MHz): δ 8.80 (d, J = 1.8 Hz, 1H), 8.53 (dd, J = 8.4, 2.2 Hz, 1H), 7.94 (d, J = 8.4 Hz, 1H), 7.33 (d, J = 8.6 Hz, 2H), 6.93 (d, J = 8.4 Hz, 2H), 6.11 (dd, J = 8.0, 4.4 Hz, 1H), 3.82 (s, 3H), 3.77 (dd, J = 13.3, 8.2 Hz, 1H), 3.59 (dd, J = 13.2, 4.4 Hz, 1H); **^{13}C{^{1}H} NMR** ($CDCl_3$, 100 MHz): δ 162.7, 160.3, 148.9, 147.8, 132.4, 131.2, 128.3 (2C), 127.6, 127.3, 119.6, 114.2 (2C), 55.3, 54.4; **FTIR** (thin film): cm^{-1} 3105, 2934, 2839, 2101, 1737, 1610, 1535, 1513, 1346, 1275, 1242, 1177, 1108, 1056, 1029, 909, 831, 731; **HRMS** (ESI^-, m/z): Calcd for $C_{16}H_{13}ClN_5O_7^-$ ($[M+Cl]^-$): 422.0509; found: 422.0508.

5. Competition Experiments

1,2-Diazidation: 1-Phenyl-1,3-butadiene -vs- Styrene

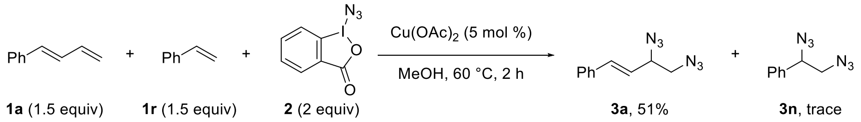

To a 2-dram vial equipped with a Teflon-coated stir bar was added copper(II) acetate (2.7 mg, 0.015 mmol, 0.05 equiv). Methanol (1.5 mL) was added, followed by sequential addition of 1-phenyl-1,3-butadiene **1a** (63.0 µL, 0.45 mmol, 1.5 equiv), styrene **1r** (51.7 µL, 0.45 mmol, 1.5 equiv), and Zhdankin's reagent **2** (173.4 mg, 0.6 mmol, 2.0 equiv). The vial was capped and stirred at 60 °C in an aluminum heating block for 2 h. The resulting crude mixture was filtered through activated, neutral Al_2O_3 (Brockman Grade I, 58–60 Å mesh powder) and concentrated *in vacuo* to yield the crude product. Purification by column chromatography (silica gel, 100% hexanes to 5% ethyl acetate–hexanes) afforded **3a** as a colorless oil (32.8 mg, 51%). Product **3n** was detected in trace (<5%) amounts from the crude ^{1}H NMR using dibromomethane as a quantitative internal standard.

Two-Component 1,2-Azidooxygenation: 1-Phenyl-1,3-butadiene -vs- Styrene

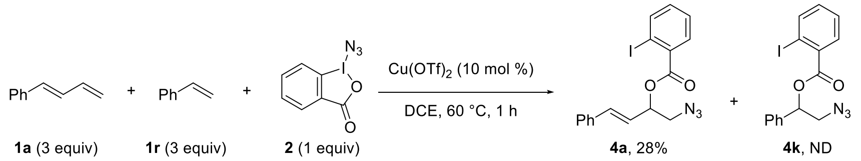

To a 1-dram vial equipped with a Teflon-coated stir bar was added copper(II) trifluoromethanesulfonate (10.9 mg, 0.03 mmol, 0.1 equiv). 1,2-Dichloroethane (1.5 mL) was added, followed by sequential addition of 1-phenyl-1,3-butadiene **1a** (126.0 µL, 0.9 mmol, 3.0 equiv), styrene **1r** (103.5 µL, 0.9 mmol, 3.0 equiv), and Zhdankin's reagent **2** (86.7 mg, 0.3 mmol, 1.0 equiv). The vial was capped and stirred at 60 °C in an aluminum heating block for 1 h. The resulting crude mixture was filtered through activated, neutral Al_2O_3 (Brockman Grade I, 58–60 Å mesh powder) and concentrated *in vacuo* to yield the crude product. Purification by column chromatography (silica gel, 100% hexanes to 20% ethyl acetate–hexanes) afforded **4a** as a colorless oil (35.5 mg, 28%). Product **4k** was not detected in the crude ^{1}H NMR.

Three-Component 1,2-Azidooxygenation: 1-Phenyl-1,3-butadiene -vs- Styrene

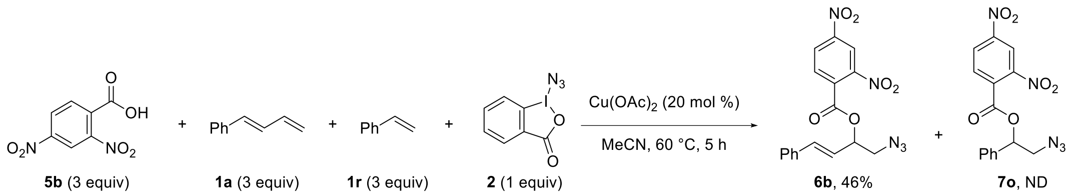

To a 1-dram vial equipped with a Teflon-coated stir bar was added copper(**II**) acetate (10.9 mg, 0.06 mmol, 0.2 equiv). Acetonitrile (1.5 mL) was added, followed by sequential addition of 1-phenyl-1,3-butadiene **1a** (126.0 µL, 0.9 mmol, 3.0 equiv), styrene **1r** (103.5 µL, 0.9 mmol, 3.0 equiv), and Zhdankin's reagent **2** (86.7 mg, 0.3 mmol, 1.0 equiv). The vial was capped and stirred at 60 °C in an aluminum heating block for 5 h. The resulting crude mixture was filtered through activated, neutral Al_2O_3 (Brockman Grade **I**, 58–60 Å mesh powder) and concentrated *in vacuo* to yield the crude product. Purification by column chromatography (silica gel, 100% hexanes to 15% ethyl acetate–hexanes) afforded **6b** as a colorless oil (53.0 mg, 46%). Product **7o** was not detected in the crude ^{1}H NMR.

6. Controls and Mechanistic Investigation Experiments

6A. Radical Scavenger Experiments

1,2-Diazidation with TEMPO

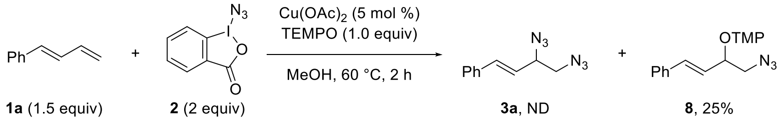

To a 1-dram vial equipped with a Teflon-coated stir bar was added copper(**II**) acetate (2.7 mg, 0.015 mmol, 0.05 equiv) and TEMPO (46.9 mg, 0.3 mmol, 1.0 equiv). Methanol (1.5 mL) was added, followed by sequential addition of 1-phenyl-1,3-butadiene **1a** (63.0 µL, 0.45 mmol, 1.5 equiv) and Zhdankin's reagent **2** (173.4 mg, 0.6 mmol, 2.0 equiv). The vial was capped and stirred at 60 °C in an aluminum heating block for 2 h. The resulting crude mixture was filtered through activated, neutral Al_2O_3 (Brockman Grade **I**, 58–60 Å mesh powder) and concentrated *in vacuo* to yield the crude product. Purification by column chromatography (silica gel, 100% hexanes to 10% ethyl acetate–hexanes) afforded **8** as a colorless oil (25.1 mg, 25%) which matched previous literature report.[5] Product **3a** was not detected in the crude ^{1}H NMR .

Three-Component 1,2-Azidooxygenation with TEMPO

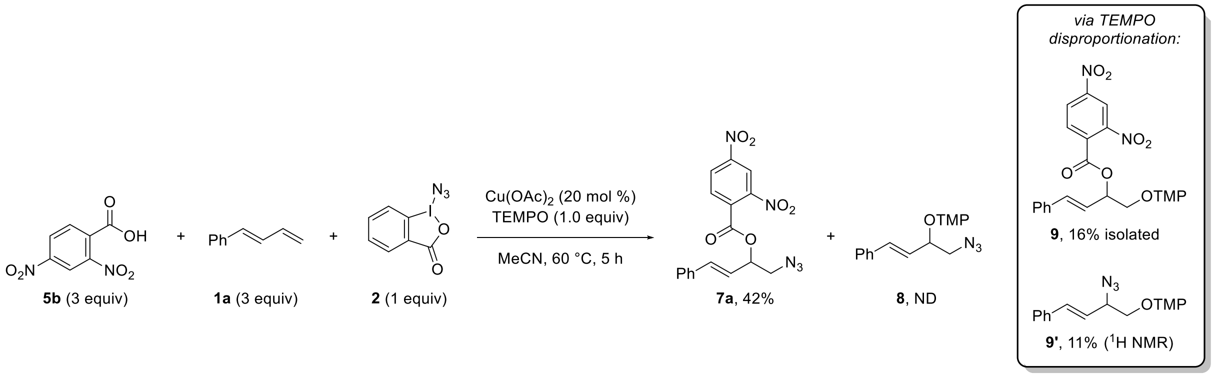

To a 2-dram vial equipped with a Teflon-coated stir bar was added copper(**II**) acetate (10.9 mg, 0.06 mmol, 0.2 equiv), 2,4-dinitrobenzoic acid (190.9 mg, 0.9 mmol, 3.0 equiv), and TEMPO (46.9 mg, 0.3 mmol, 1.0 equiv). Acetonitrile (1.5 mL) was added, followed by sequential addition of 1-phenyl-1,3-butadiene **1a** (126.0 µL, 0.9 mmol, 3.0 equiv) and Zhdankin's reagent **2** (86.7 mg, 0.3 mmol, 1.0 equiv). The vial was capped and stirred at 60 °C in an aluminum heating block for 5 h. The resulting crude mixture was filtered through activated, neutral Al_2O_3 (Brockman Grade **I**, 58–60 Å mesh powder) and concentrated *in vacuo* to yield the crude product. Purification by column chromatography (silica gel, 100% hexanes to 10% ethyl acetate–hexanes) afforded **7a** as a colorless oil (48.7 mg, 42%) and **9** as a colorless oil (26.7 mg, 16%; product from acid-driven disproportionation of TEMPO).[2] Product **9'** (also likely from acid-driven disproportionation of TEMPO) was detected in 11% yield by crude ^{1}H NMR using dibromomethane as a quantitative internal standard. Neither product **7a** nor any putative radical-trapped species (such as **8**) were detected in the crude ^{1}H NMR.

Three-Component 1,2-Azidooxygenation with BHT/DPE

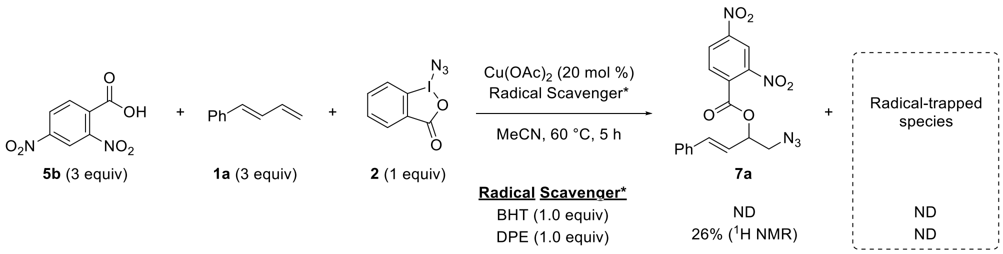

To a 2-dram vial equipped with a Teflon-coated stir bar was added copper(**II**) acetate (10.9 mg, 0.06 mmol, 0.2 equiv), 2,4-dinitrobenzoic acid (190.9 mg, 0.9 mmol, 3.0 equiv), and the radical scavenger (0.3 mmol, 1.0 equiv). Acetonitrile (1.5 mL) was added, followed by sequential addition of 1-phenyl-1,3-butadiene **1a** (126.0 µL, 0.9 mmol, 3.0 equiv) and Zhdankin's reagent **2** (86.7 mg, 0.3 mmol, 1.0 equiv). The vial was capped and stirred at 60 °C in an aluminum heating block for 5 h. The resulting crude mixture was filtered through activated, neutral Al_2O_3 (Brockman Grade **I**, 58–60 Å mesh powder) and concentrated *in vacuo* to yield the crude product. Neither product **7a** nor any putative radical-trapped species were detected in the crude ^{1}H NMR.

6B. Acid Studies on the Three-Component Azidooxygenation of 1,3-Dienes

Three-Component Azidooxygenation with Sodium Benzoate Salt

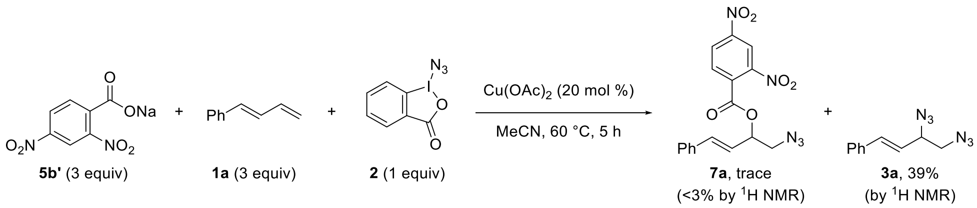

To a 2-dram vial equipped with a Teflon-coated stir bar was added copper(**II**) acetate (10.9 mg, 0.06 mmol, 0.2 equiv) and sodium 2,4-dinitrobenzoate (210.6 mg, 0.9 mmol, 3.0 equiv). Acetonitrile (1.5 mL) was added, followed by sequential addition of 1-phenyl-1,3-butadiene **1a** (126.0 µL, 0.9 mmol, 3.0 equiv) and Zhdankin's reagent **2** (86.7 mg, 0.3 mmol, 1.0 equiv). The vial was capped and stirred at 60 °C in an aluminum heating block for 5 h. The resulting crude mixture was filtered through activated, neutral Al_2O_3 (Brockman Grade **I**, 58–60 Å mesh powder) and concentrated *in vacuo* to yield the crude product. Product **7a** was detected in <3% by quantitative ^{1}H NMR of the crude reaction. (The 1,2-diazidation product **3a** was observed in 39% yield by quantitative ^{1}H NMR of the crude reaction.)

Crossover Acid Experiments

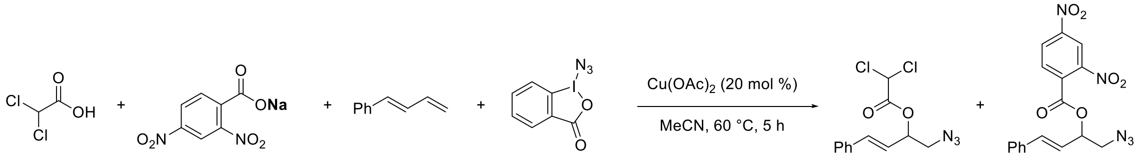

5e (3 equiv) **5b'** (3 equiv) **1a** (3 equiv) **2** (1 equiv) **6e**, 15% **6b**, 21%

To a 1-dram vial equipped with a Teflon-coated stir bar was added copper(**II**) acetate (3.6 mg, 0.02 mmol, 0.2 equiv) and sodium 2,4-dinitrobenzoate (70.2 mg, 0.3 mmol, 3.0 equiv). Acetonitrile (0.5 mL) was added, followed by sequential addition of 1-phenyl-1,3-butadiene **1a** (42.0 µL, 0.3 mmol, 3.0 equiv), dichloroacetic acid (24.7 µL, 0.3 mmol, 3.0 equiv), and Zhdankin's reagent **2** (28.9 mg, 0.1 mmol, 1.0 equiv). The vial was capped and stirred at 60 °C in an aluminum heating block for 5 h. The resulting crude mixture was filtered through activated, neutral Al_2O_3 (Brockman Grade **I**, 58–60 Å mesh powder) and concentrated *in vacuo* to yield the crude product. Purification by column chromatography (silica gel, 100% hexanes to 15% ethyl acetate–hexanes) afforded **6e** as a clear oil (4.6 mg, 15%) and **6b** as a clear oil (8.0 mg, 21%).

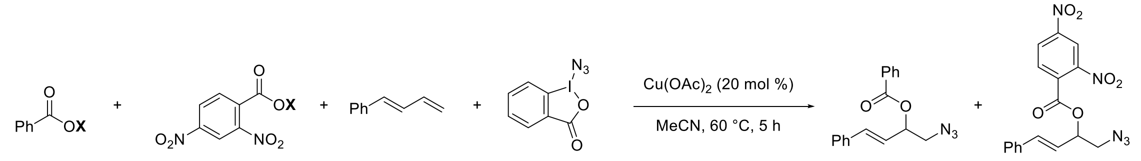

entry	**5c/5c'** (3 equiv)	**5b/5b'** (3 equiv)	**1a** (3 equiv)	**2** (1 equiv)	**6c**	**6b**
1	**5c** (X = **H**)	**5b'** (X = **Na**)			ND	11% (^{1}H NMR)
2	**5c'** (X = **Na**)	**5b** (X = **H**)			ND	15% (^{1}H NMR)
3	**5c** (X = **H**)	**5b** (X = **H**)			ND	40%

To a 1-dram vial equipped with a Teflon-coated stir bar was added copper(**II**) acetate (3.6 mg, 0.02 mmol, 0.2 equiv) and sodium 2,4-dinitrobenzoate (70.2 mg, 0.3 mmol, 3.0 equiv) or 2,4-dinitrobenzoic acid (63.6 mg). Acetonitrile (0.5 mL) was added, followed by sequential addition of 1-phenyl-1,3-butadiene **1a** (42.0 µL, 0.3 mmol, 3.0 equiv), benzoic acid (36.6 mg, 0.3 mmol, 3.0 equiv) or sodium benzoate (43.2 mg), and Zhdankin's reagent **2** (28.9 mg, 0.1 mmol, 1.0 equiv). The vial was capped and stirred at 60 °C in an aluminum heating block for 5 h. The resulting crude mixture was filtered through activated, neutral Al_2O_3 (Brockman Grade **I**, 58–60 Å mesh powder) and concentrated *in vacuo* to yield the crude product. For all runs, **6c** was not detected by the crude ^{1}H NMR. For entry 3, purification by column chromatography (silica gel, 100% hexanes to 20% ethyl acetate–hexanes) afforded **6b** as a white solid (15.5 mg, 40%). Purification attempts for entries 1 and 2 did not create clean products.

Exogenous Acid Study

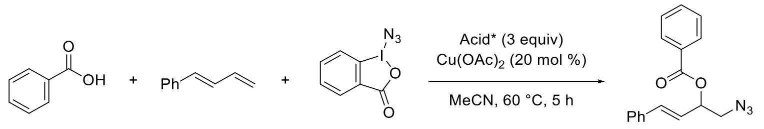

5c (3 equiv) **1a** (3 equiv) **2** (1 equiv) **7a**

**Acid**	**7a**
TfOH	ND
MsOH	ND
TFA	ND

To a 1-dram vial equipped with a Teflon-coated stir bar was added copper(**II**) acetate (3.6 mg, 0.02 mmol, 0.2 equiv) and benzoic acid (36.6 mg, 0.3 mmol, 3.0 equiv). Acetonitrile (0.5 mL) was added, followed by sequential addition of 1-phenyl-1,3-butadiene **1a** (42.0 µL, 0.3 mmol, 3.0 equiv), Zhdankin's reagent **2** (28.9

mg, 0.1 mmol, 1.0 equiv), and acid (0.3 mmol, 3.0 equiv). The vial was capped and stirred at 60 °C in an aluminum heating block for 5 h. The resulting crude mixture was filtered through activated, neutral Al_2O_3 (Brockman Grade **I**, 58–60 Å mesh powder) and concentrated *in vacuo*. Product **7a** was not detected in the crude ^{1}H NMR. (Some minor 1,2-diazidation product was observed in the crude reaction.)

7. Time-Course Monitoring Experiments

7A. 1,2-Diazidation

To a 1-dram vial equipped with a Teflon-coated stir bar was added copper(**II**) acetate (1.8 mg, 0.01 mmol, 0.05 equiv). Methanol (1.0 mL) was added, followed by sequential addition of 1-phenyl-1,3-butadiene/styrene (0.4 mmol, 1.5 equiv), Zhdankin's reagent **2** (115.7 mg, 0.6 mmol, 2.0 equiv), and 1,1,2,2-tetrachloroethane (21.1 µL, 0.20 mmol, 1.0 equiv). The vial was capped and stirred at 60 °C in an aluminum heating block. At the marked timepoints, a 50 µL aliquot was removed from the reaction, filtered through a short plug of activated, neutral Al_2O_3 (Brockman Grade **I**, 58–60 Å mesh powder) with $CDCl_3$ into an NMR tube with a total volume of ca. 0.4 mL The sample was analyzed for yield by ^{1}H NMR by comparison of peak integrations (solvent suppression of both MeOH peaks, 16 scans, 2 second relaxation delay).

Figure S1. 1,2-Diazidation of 1,3-Dienes

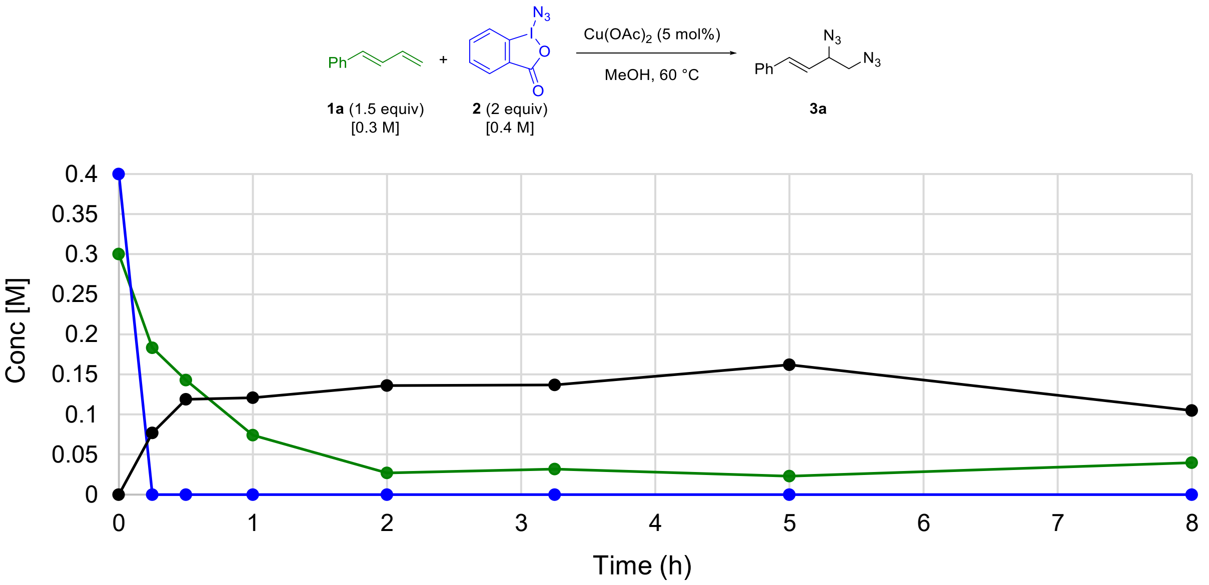

Figure S2. 1,2-Diazidation of Styrene

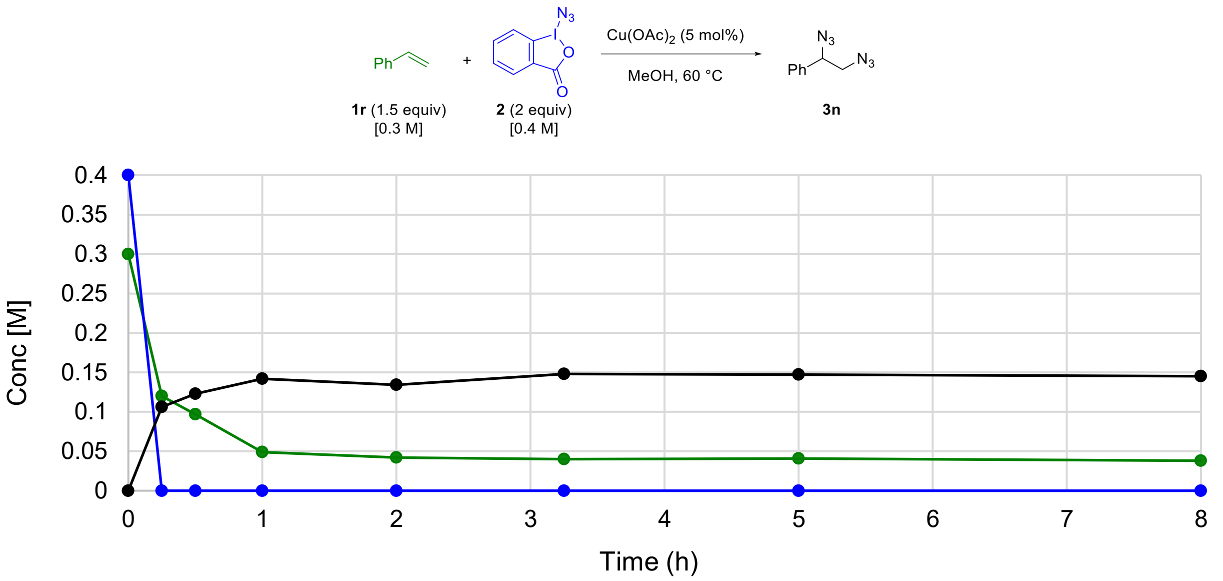

7B. Two-Component 1,2-Azidooxygenation

To a 1-dram vial equipped with a Teflon-coated stir bar was added copper(**II**) trifluoromethanesulfonate (7.2 mg, 0.02 mmol, 0.1 equiv). 1,2-Dichloroethane (1.0 mL) was added, followed by sequential addition of the 1-phenyl-1,3-butadiene/styrene (0.6 mmol, 3.0 equiv), Zhdankin's reagent **2** (57.8 mg, 0.2 mmol, 1.0 equiv) and 1,1,2,2-tetrachloroethane (21.1 µL, 0.20 mmol, 1.0 equiv). The vial was capped and stirred at 60 °C in an aluminum heating block. At the marked timepoints, a 50 µL aliquot was removed from the reaction, filtered through a shor plug of activated, neutral Al_2O_3 (Brockman Grade **I**, 58–60 Å mesh powder) with $CDCl_3$ into an NMR tube with a total volume of ca. 0.4 mL The sample was analyzed for yield by ^1H NMR by comparison of peak integrations (solvent suppression of the DCE peak, 16 scans, 2 s relaxation delay).

Figure S3. Two-Component 1,2-Azidooxygenation of 1,3-Dienes

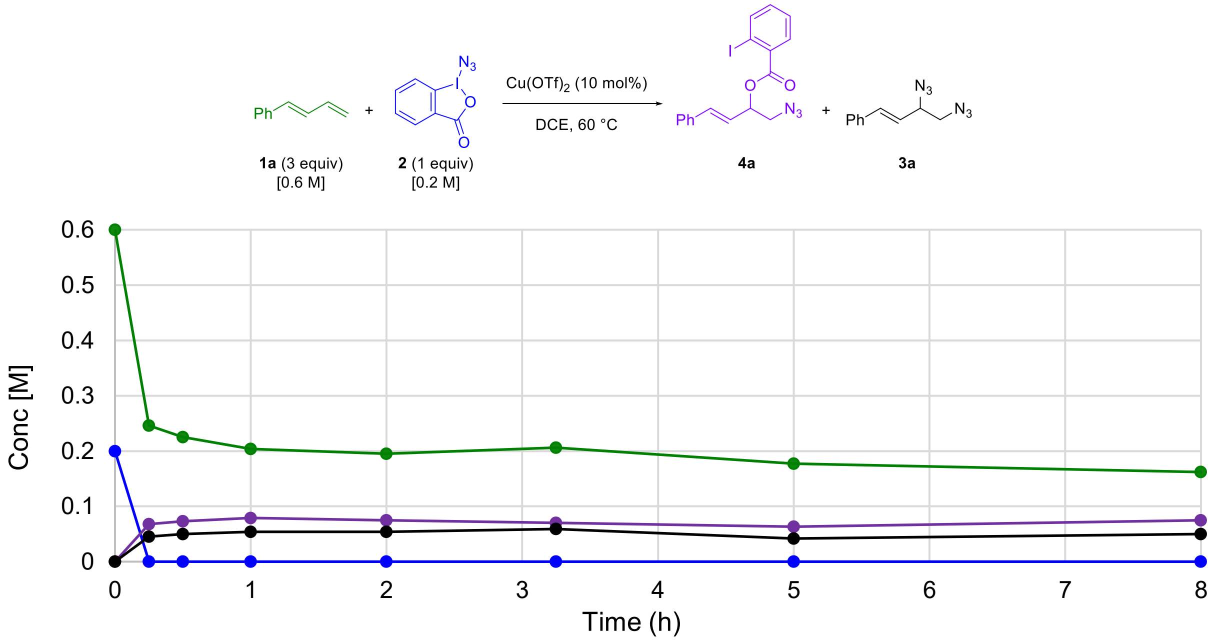

Figure S4. Two-Component 1,2-Azidooxygenation of Styrene

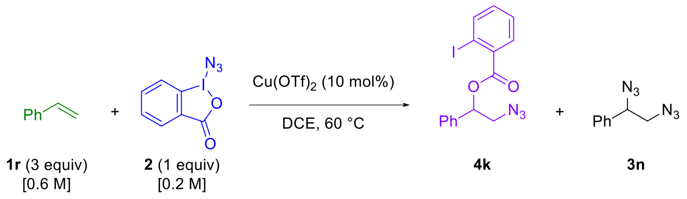

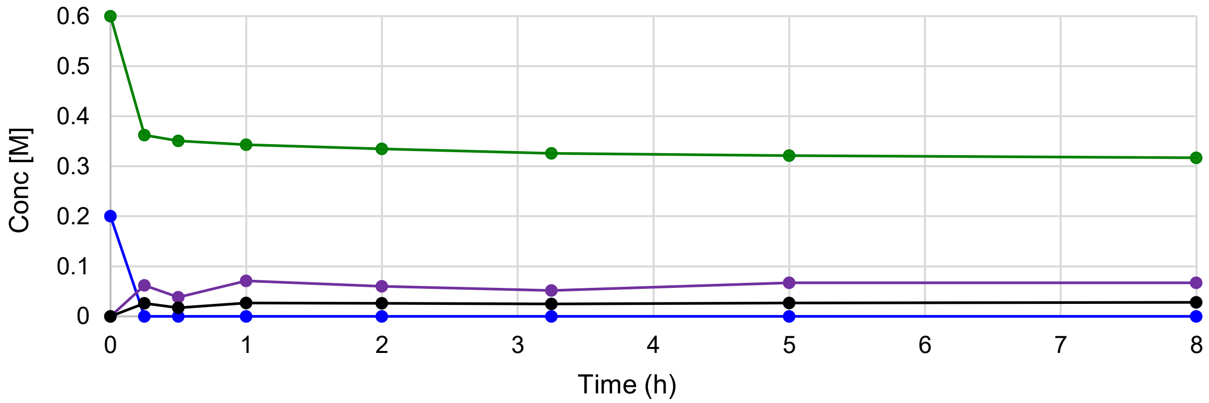

7C. Three-Component 1,2-Azidooxygenation

To a 1-dram vial equipped with a Teflon-coated stir bar was added copper(**II**) acetate (7.3 mg, 0.04 mmol, 0.2 equiv) and 2,4-dinitrobenzoic acid **5b** (127.3 mg, 0.6 mmol, 3.0 equiv). Acetonitrile (1.0 mL) was added, followed by sequential addition of 1-phenyl-1,3-butadiene/styrene (0.6 mmol, 3.0 equiv), Zhdankin's reagent **2** (57.8 mg, 0.2 mmol, 1.0 equiv) and 1,1,2,2-tetrachloroethane (21.1 µL, 0.20 mmol, 1.0 equiv). The vial was capped and stirred at 60 °C in an aluminum heating block. At the marked timepoints, a 50 µL aliquot was removed from the reaction, filtered through a short plug of activated, neutral Al_2O_3 (Brockman Grade **I**, 58–60 Å mesh powder) with $CDCl_3$ into an NMR tube with a total volume of ca. 0.4 mL The sample was analyzed for yield by ^{1}H NMR by comparison of peak integrations (solvent suppression of the MeCN peak, 16 scans, 2 second relaxation delay).

Figure S5. Three-Component 1,2-Azidooxygenation of 1,3-Dienes

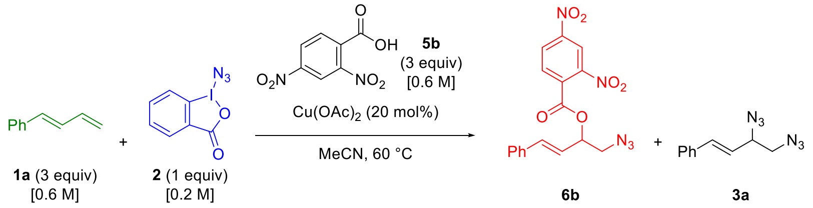

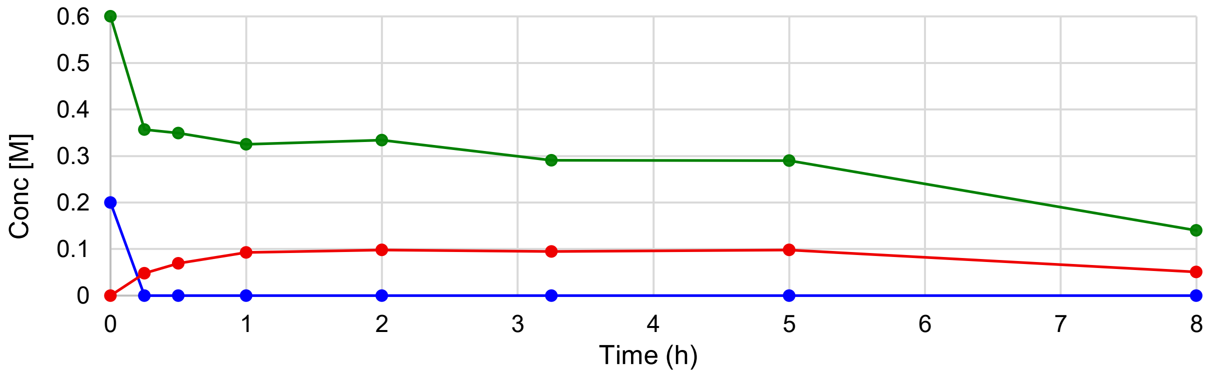

Figure S6. Three-Component 1,2-Azidooxygenation of Styrene

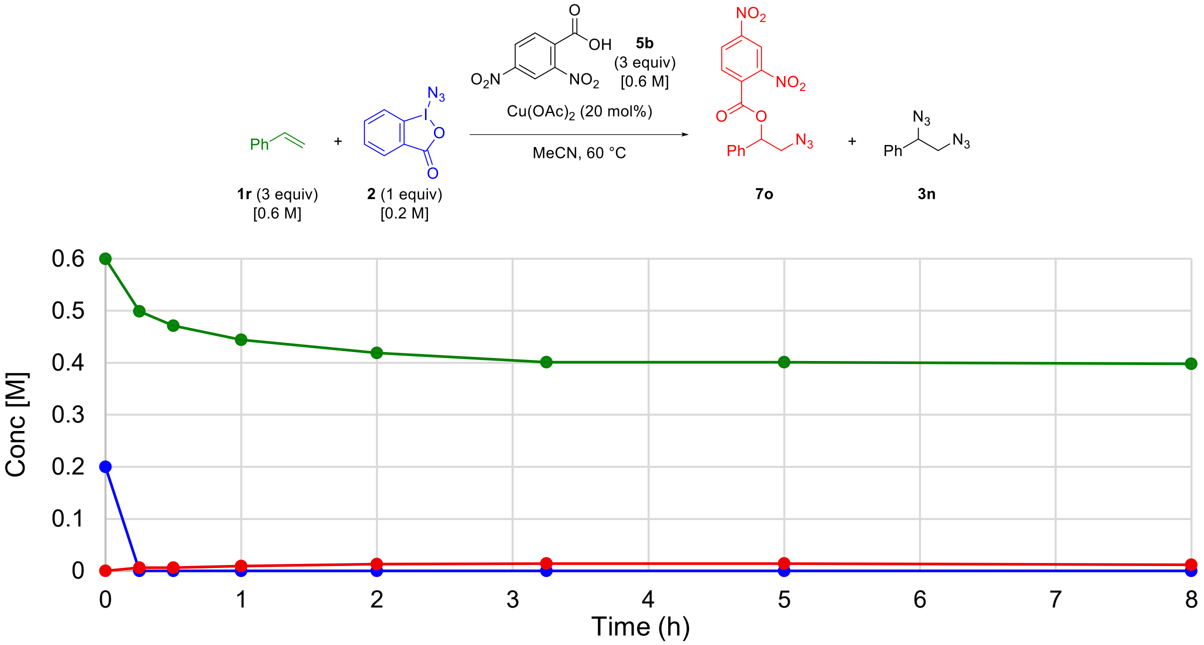

8. UV-Visible Studies

Figure S7. UV-Vis Spectroscopy of pentafluorobenzoic acid (F5BzOH), sodium pentafluorobenzoate (F5BzONa), Zhdankin's reagent (**I(III)**), and the combination of pentafluorobenzoic acid and Zhdankin's reagent (F5BzOH+**I(III)**). MeCN (solvent) removed using double-beam UV-Vis instrument with MeCN blank.

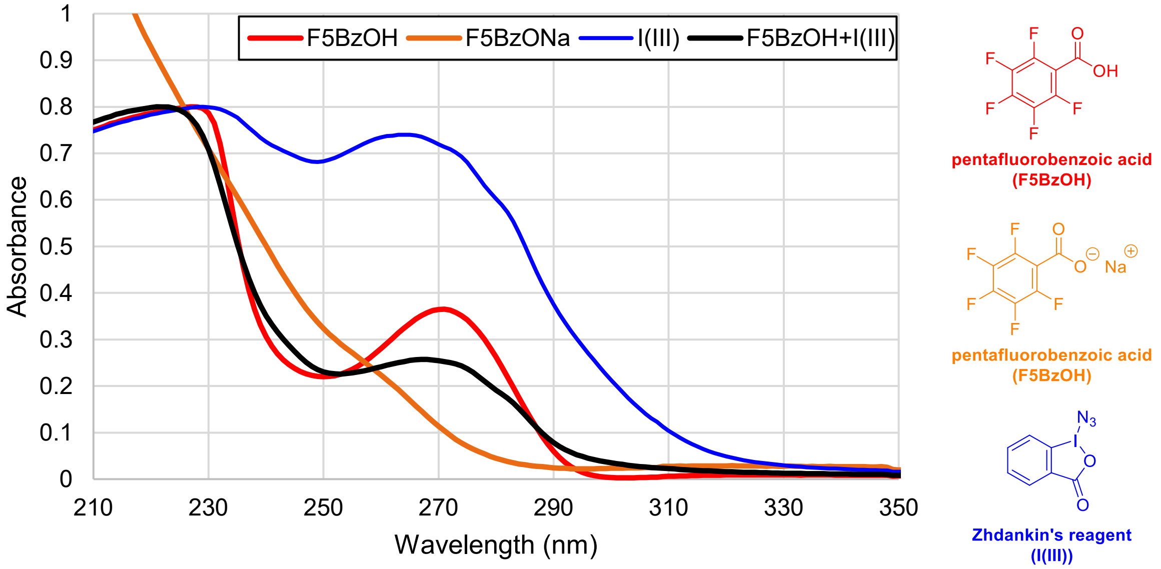

First, the UV-Vis spectrum of pentafluorobenzoic acid (F5BzOH), sodium pentafluorobenzoate (F5BzONa), and Zhdankin's reagent (**I(III)** were compared to the spectrum of pentafluorobenzoic acid combined with Zhdankin's reagent (Figure S7). In this spectrum, the combined F5BzOH and **I(III)**) displayed a similar spectrum to the pentafluorobenzoic acid (F5BzOH), possessing similar absorbances for both the first and

second λmax values of F5BzOH around 270 nm. This result implies that pentafluorobenzoica acid is likely still protonated in the presence of Zhdankin's reagent, although some deprotonation cannot be ruled out.

(To obtain UV-Vis spectra, solutions were made of each standard in MeCN. The samples were diluted. For the combination spectra, the solutions were mixed, then diluted. In all cases in which the values exceeded 1, the values were normalized.)

9. Possible Mechanistic Rationale

Figure S8. Possible Mechanism for the 1,2-Diazidation of 1,3-Dienes.

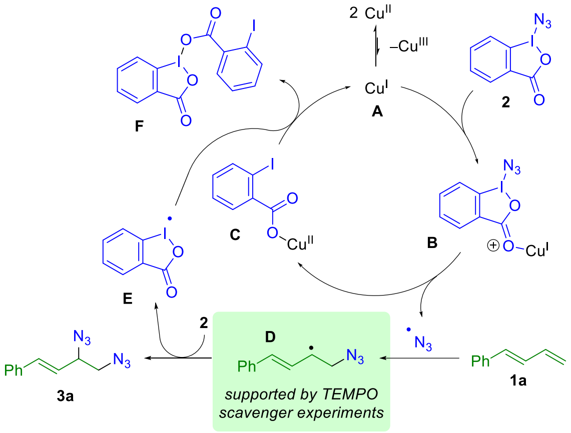

This reaction is proposed to start with disproportionation of copper(II) to generate copper(I). The copper(I) then coordinates with Zhdankin's reagent (**2**), leading to release of an azido radical and copper(II) benzoate (**C**). The azido radical adds to the 1,3-diene (**1a**), generating the allylic radical species (**D**) that is detected from TEMPO trapping experiments (Scheme 5A). Another equivalent of Zhdankin's reagent (**2**) adds an azide group to furnish the product (**3a**) and radical species (**E**, may be in a form other than that depicted). Combination of this radical species with the copper(II) benzoate reductively turns over the copper and generates a product (**F** shown as a possibility).

Figure S9. Possible Mechanism for the 1,2-Azidooxygenation of 1,3-Dienes.

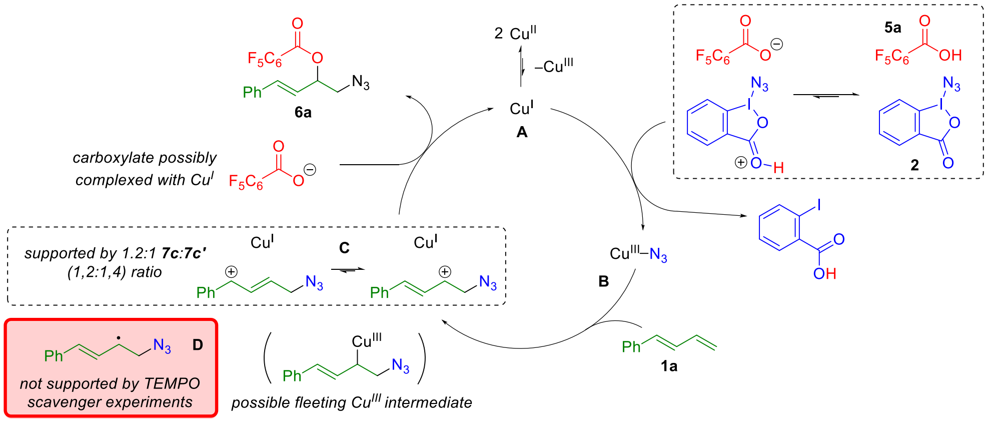

This reaction is proposed to start with disproportionation of copper(II) to generate copper(I). Correspondingly, Zhdankin's reagent (**2**) is protonated in the presence of the strong carboxylic acid (**5a**).

With the Zhdankin's reagent coordination occupied by the proton, the protonated complex reacts with copper(I) to produce a copper(III)–azide species (**B**). This species adds to the 1,3-dienes (**1a**) to produce an allylic carbocation (**C**) and regenerate copper(I). The allylic resonance in this species is supported by the 1,2:1,4-addition products detected in **7c**:**7c'** (4'-CF_3-1-phenyl-1,3-butadiene). It is also possible that a fleeting allylic copper(III) intermediate is present; however, an allylic radical is not anticipated due to the lack of radical trapping in this reaction and the production of the desired product (**6a**) in the presence of TEMPO (Scheme 5B). From carbocation **C**, addition of the carboxylate furnishes the product (**6a**).

10. References

1. Hendrick, C. E.; Bitting, K. J.; Cho, S.; Wang, Q., "Site-Selective Copper-Catalyzed Amination and Azidation of Arenes and Heteroarenes via Deprotonative Zincation." *J. Am. Chem. Soc.* **2017**, *139*, 11622-11628.
2. Baldassarre, S. M.; Sato, H. S.; Louise, A. P.; Summer, L. L.; Wilson, B. P.; Hemric, B. N., "Three-Component 1,2-Dioxygenation of 1,3-Dienes Using Carboxylic Acids and TEMPO." *J. Org. Chem.* **2024**, *89*, 16865-16872.
3. (a) Hu, J.-W.; Zhong, Y.; Song, R.-J., "Copper/iron controlled regioselective 1,2-carboazidation of 1,3-dienes with acetonitrile and azidotrimethylsilane." *Org. Biomol. Chem.* **2025**, *23*, 1437-1442; (b) Zhu, C.-F.; Mai, J.-J.; Li, X.-J.; Shi, M.; Dong, X.; Fu, H.; Shen, M.-H.; Xu, H.-D., "Cobalt-catalyzed regioselective diazidation of 1-aryl-1,3-dienes enabled by a single electron transfer/radical addition/group transfer relay process." *Org. Chem. Front.* **2025**, *12*, 1461-1466.
4. (a) Lu, M.-Z.; Wang, C.-Q.; Loh, T.-P., "Copper-Catalyzed Vicinal Oxyazidation and Diazidation of Styrenes under Mild Conditions: Access to Alkyl Azides." *Org. Lett.* **2015**, *17*, 6110-6113; (b) Fumagalli, G.; Rabet, P. T. G.; Boyd, S.; Greaney, M. F., "Three-Component Azidation of Styrene-Type Double Bonds: Light-Switchable Behavior of a Copper Photoredox Catalyst." *Angew. Chem. Int. Ed.* **2015**, *54*, 11481-11484.
5. Li, G.-Q.; Li, Z.-Q.; Jiang, M.; Zhang, Z.; Qian, Y.; Xiao, W.-J.; Chen, J.-R., "Photoinduced Copper-Catalyzed Asymmetric Three-Component Radical 1,2-Azidooxygenation of 1,3-Dienes." *Angew. Chem. Int. Ed.* **2024**, *63*, e202405560.

11. ^{1}H, $^{13}C\{^{1}H\}$, and ^{19}F NMR Spectra

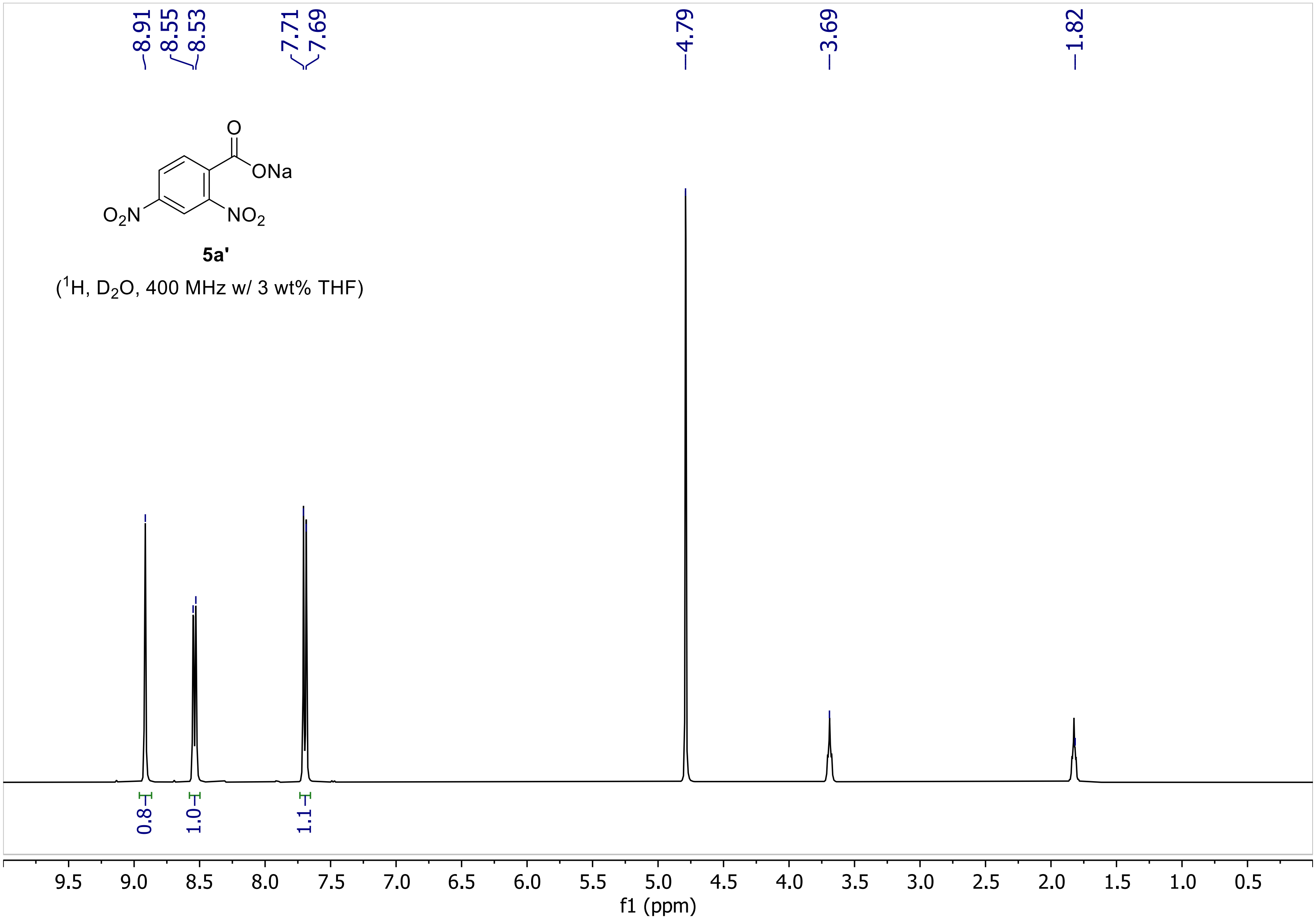

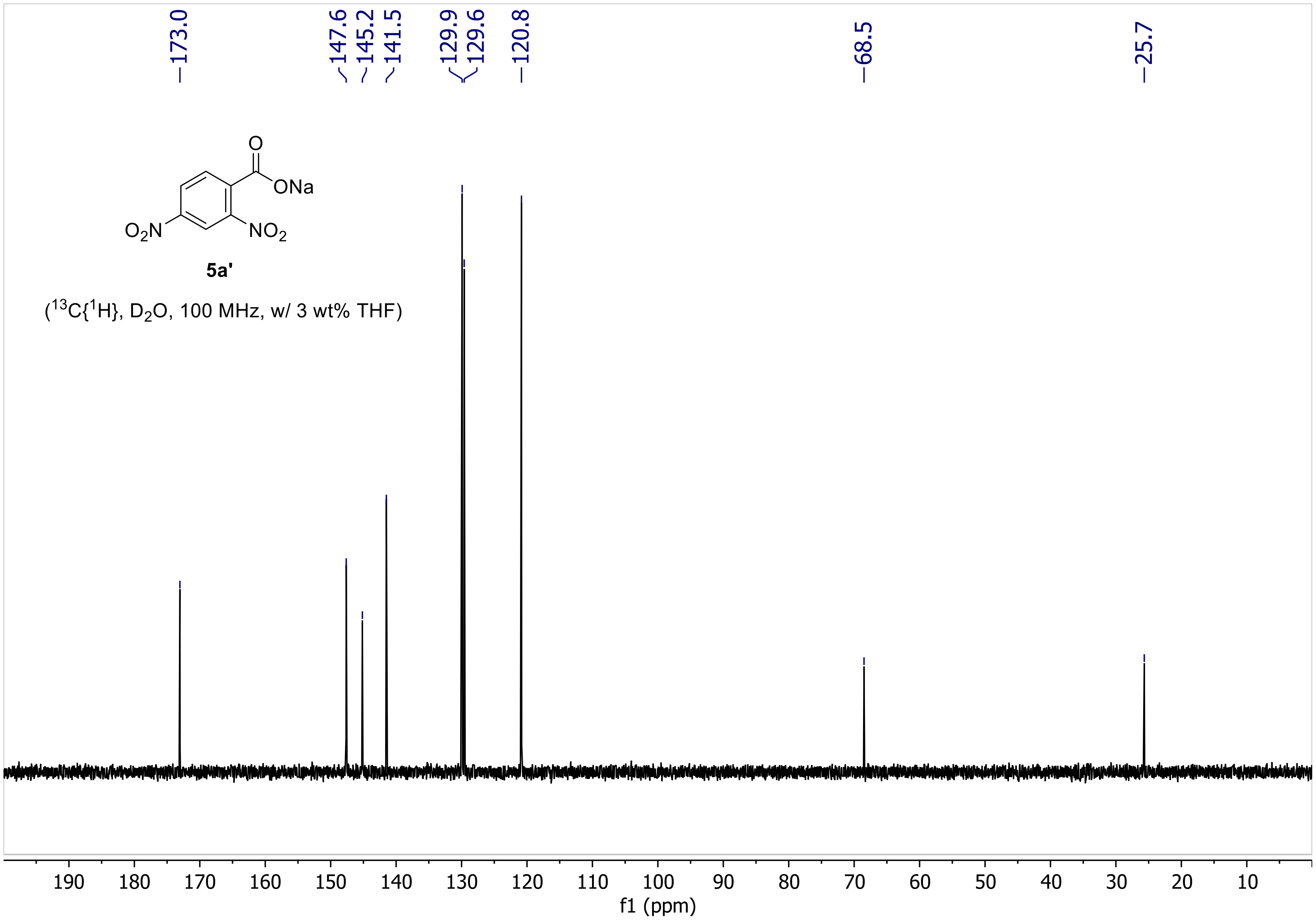
173.0
147.6
145.2
141.5
129.9
129.6
120.8
68.5
25.7
O
ONa
O_2N
NO_2
5a'
($^{13}C\{^1H\}$, D_2O, 100 MHz, w/ 3 wt% THF)
190
180
170
160
150
140
130
120
110
100
90
80
70
60
50
40
30
20
10
f1 (ppm)

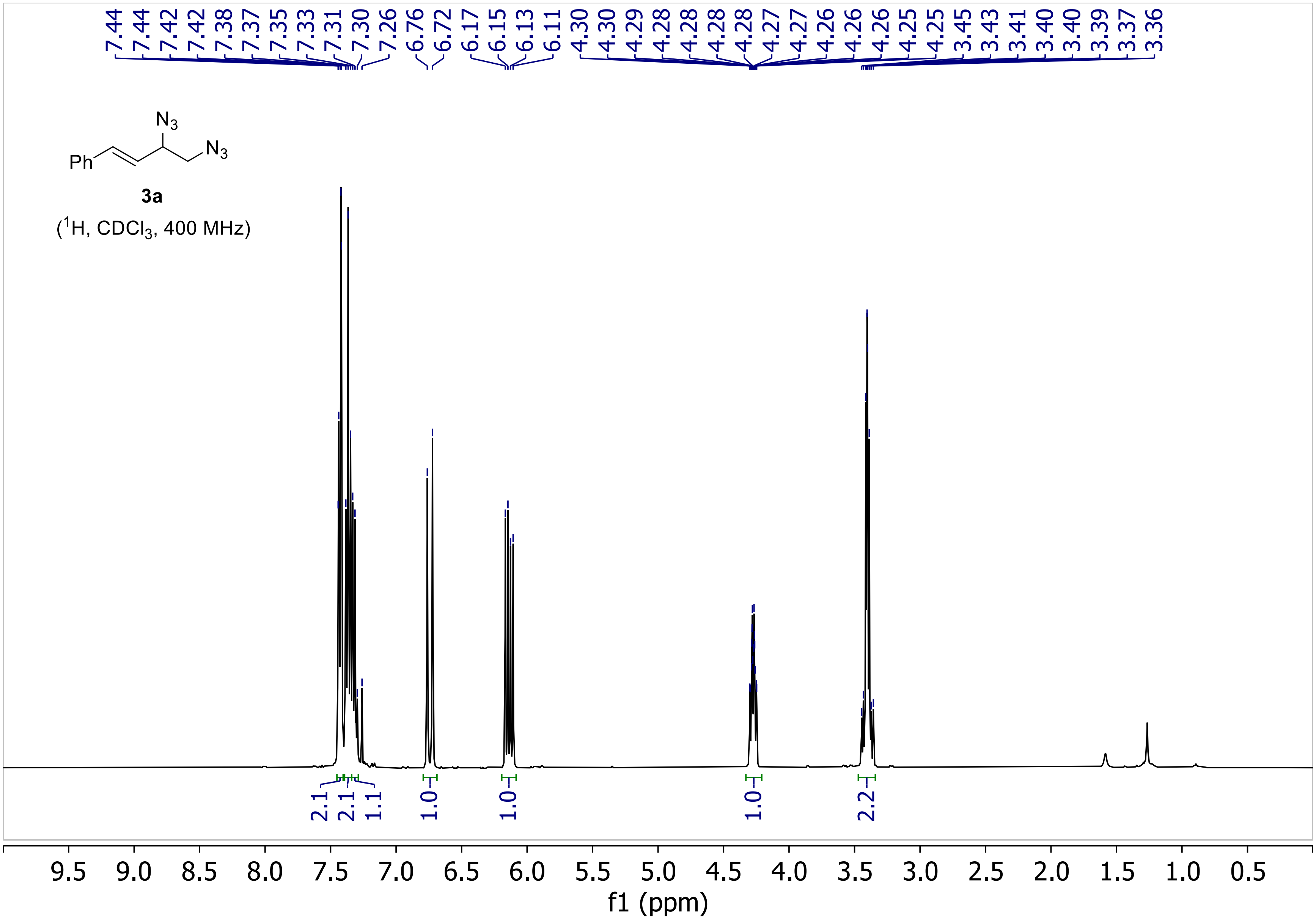
7.44
7.44
7.42
7.42
7.38
7.37
7.35
7.33
7.31
7.30
7.26
6.76
6.72
6.17
6.15
6.13
6.11
4.30
4.30
4.29
4.28
4.28
4.28
4.28
4.27
4.27
4.26
4.26
4.26
4.25
4.25
3.45
3.43
3.41
3.40
3.40
3.39
3.37
3.36
N3
Ph
N3
3a
(1H, CDCl3, 400 MHz)
2.1
2.1
1.1
1.0
1.0
1.0
2.2
9.5 9.0 8.5 8.0 7.5 7.0 6.5 6.0 5.5 5.0 4.5 4.0 3.5 3.0 2.5 2.0 1.5 1.0 0.5
f1 (ppm)

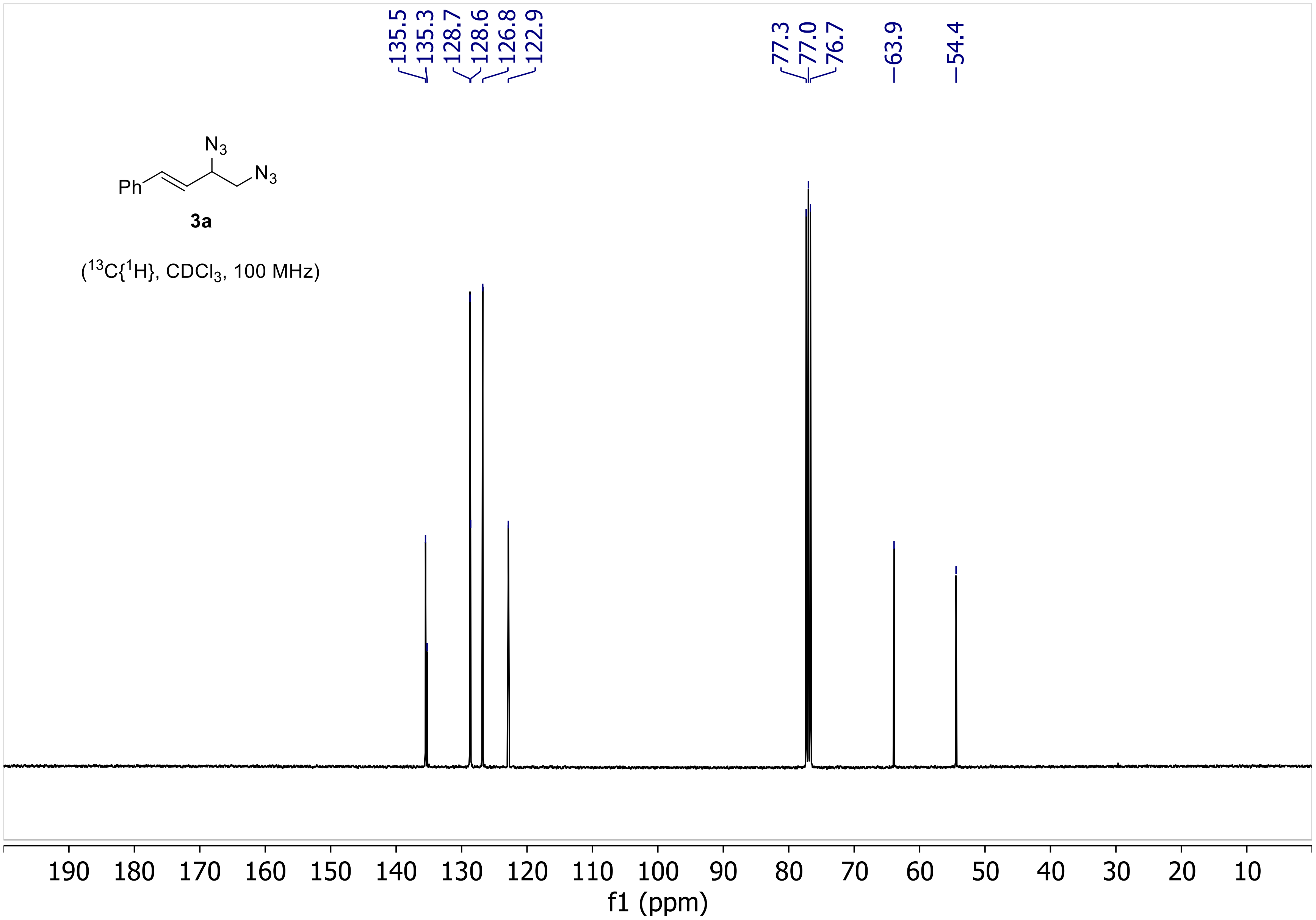
135.5
135.3
128.7
128.6
126.8
122.9
77.3
77.0
76.7
63.9
54.4
N3
Ph
N3
3a
(13C{1H}, CDCl3, 100 MHz)
190 180 170 160 150 140 130 120 110 100 90 80 70 60 50 40 30 20 10
f1 (ppm)

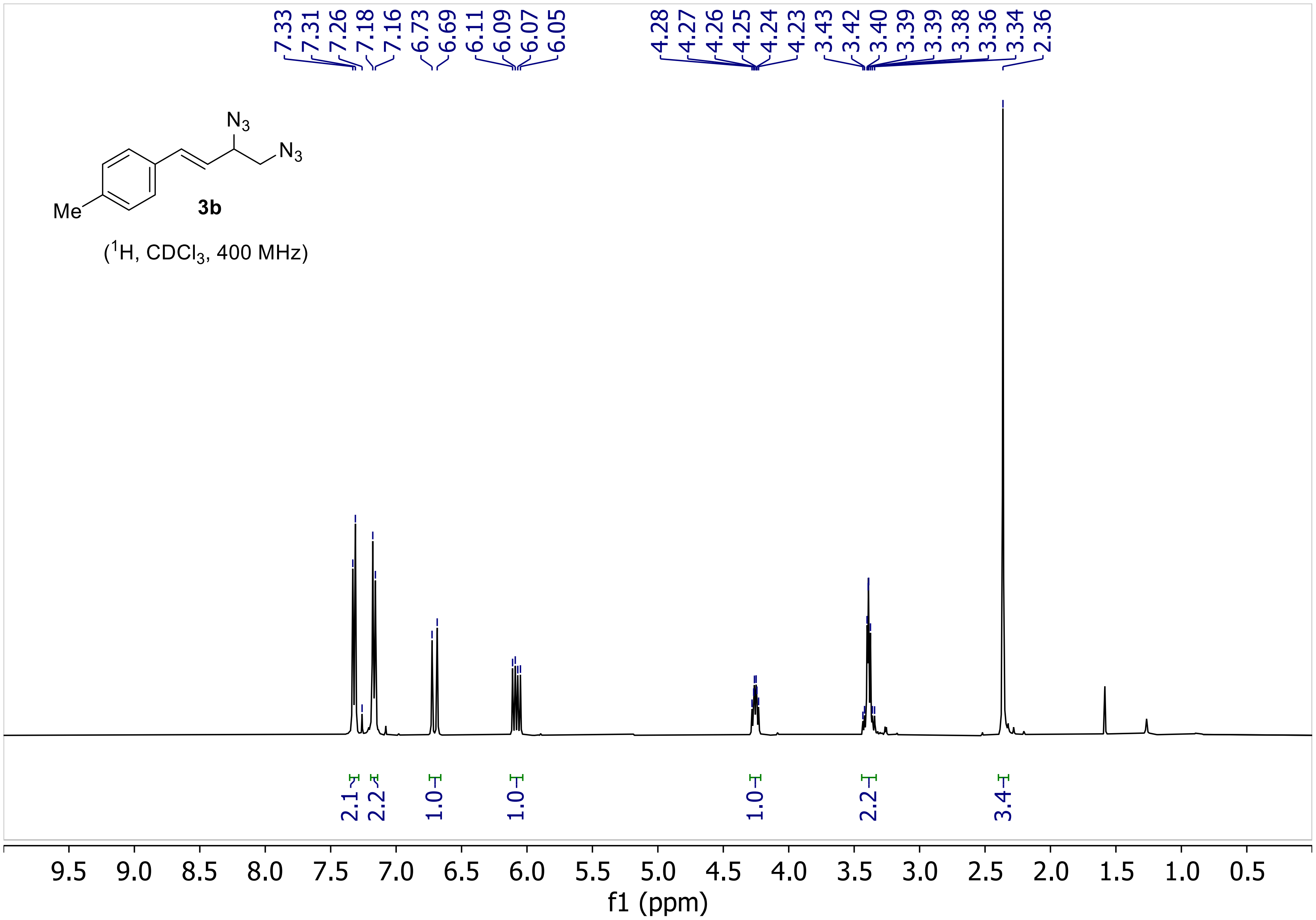

7.33 7.31 7.26 7.18 7.16 6.73 6.69 6.11 6.09 6.07 6.05
4.28 4.27 4.26 4.25 4.24 4.23 3.43 3.42 3.40 3.39 3.39 3.38 3.36 3.34 2.36
N3
N3
Me
3b
(1H, CDCl3, 400 MHz)
2.1 2.2 1.0 1.0 1.0 2.2 3.4
9.5 9.0 8.5 8.0 7.5 7.0 6.5 6.0 5.5 5.0 4.5 4.0 3.5 3.0 2.5 2.0 1.5 1.0 0.5
f1 (ppm)

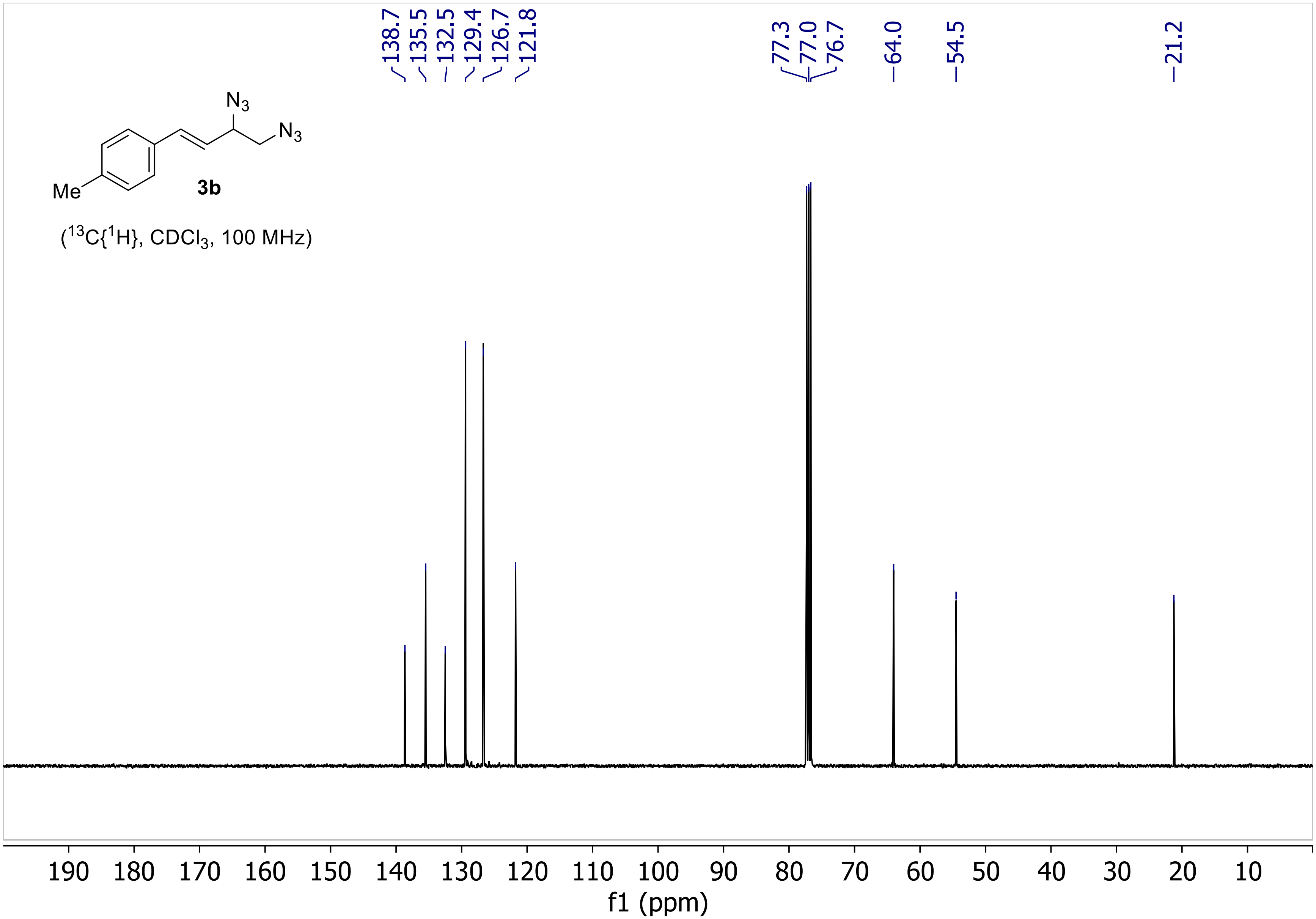

N3
N3
Me
3b
($^{13}C\{^1H\}$, $CDCl_3$, 100 MHz)
138.7
135.5
132.5
129.4
126.7
121.8
77.3
77.0
76.7
64.0
54.5
21.2
190 180 170 160 150 140 130 120 110 100 90 80 70 60 50 40 30 20 10
f1 (ppm)

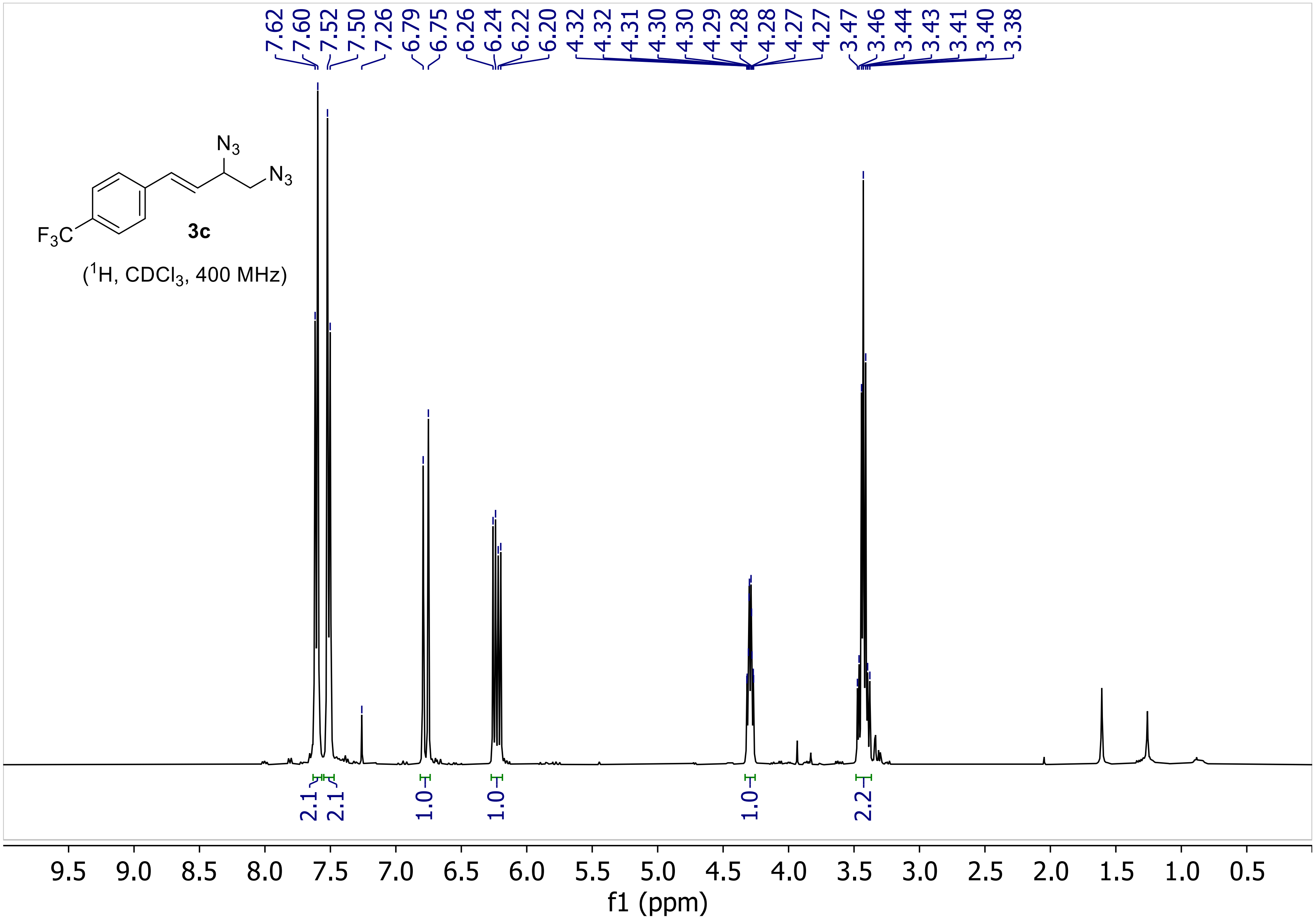
7.62 7.60 7.52 7.50 7.26 6.79 6.75 6.26 6.24 6.22 6.20 4.32 4.32 4.31 4.30 4.30 4.29 4.28 4.28 4.27 4.27 3.47 3.46 3.44 3.43 3.41 3.40 3.38
N3
N3
F3C
3c
(1H, CDCl3, 400 MHz)
2.1 2.1 1.0 1.0 1.0 2.2
9.5 9.0 8.5 8.0 7.5 7.0 6.5 6.0 5.5 5.0 4.5 4.0 3.5 3.0 2.5 2.0 1.5 1.0 0.5
f1 (ppm)

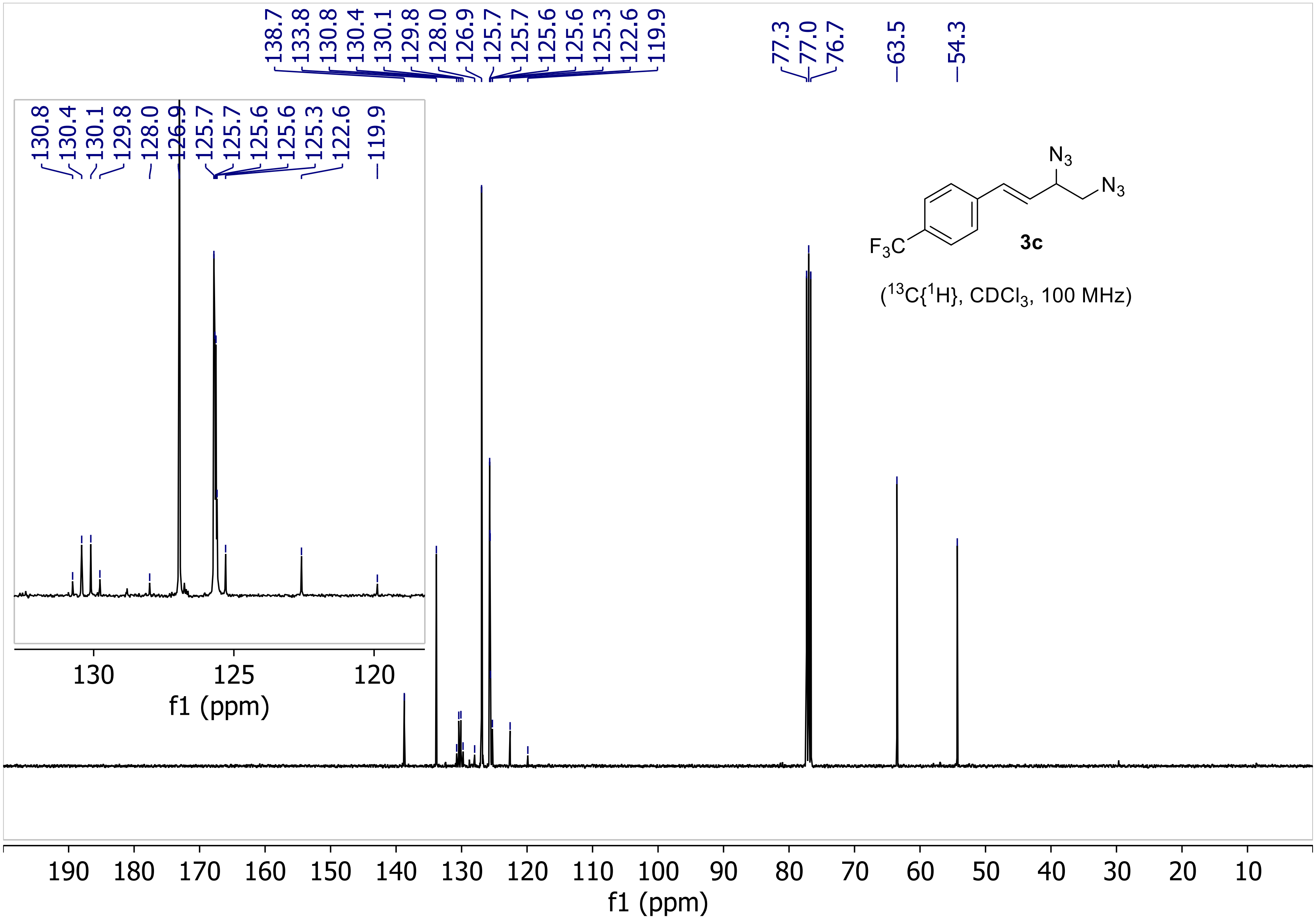

138.7
133.8
130.8
130.4
130.1
129.8
128.0
126.9
125.7
125.7
125.6
125.6
125.3
122.6
119.9
77.3
77.0
76.7
63.5
54.3
N3
N3
F3C
3c
(13C{1H}, CDCl3, 100 MHz)
130
125
120
f1 (ppm)
190 180 170 160 150 140 130 120 110 100 90 80 70 60 50 40 30 20 10
f1 (ppm)

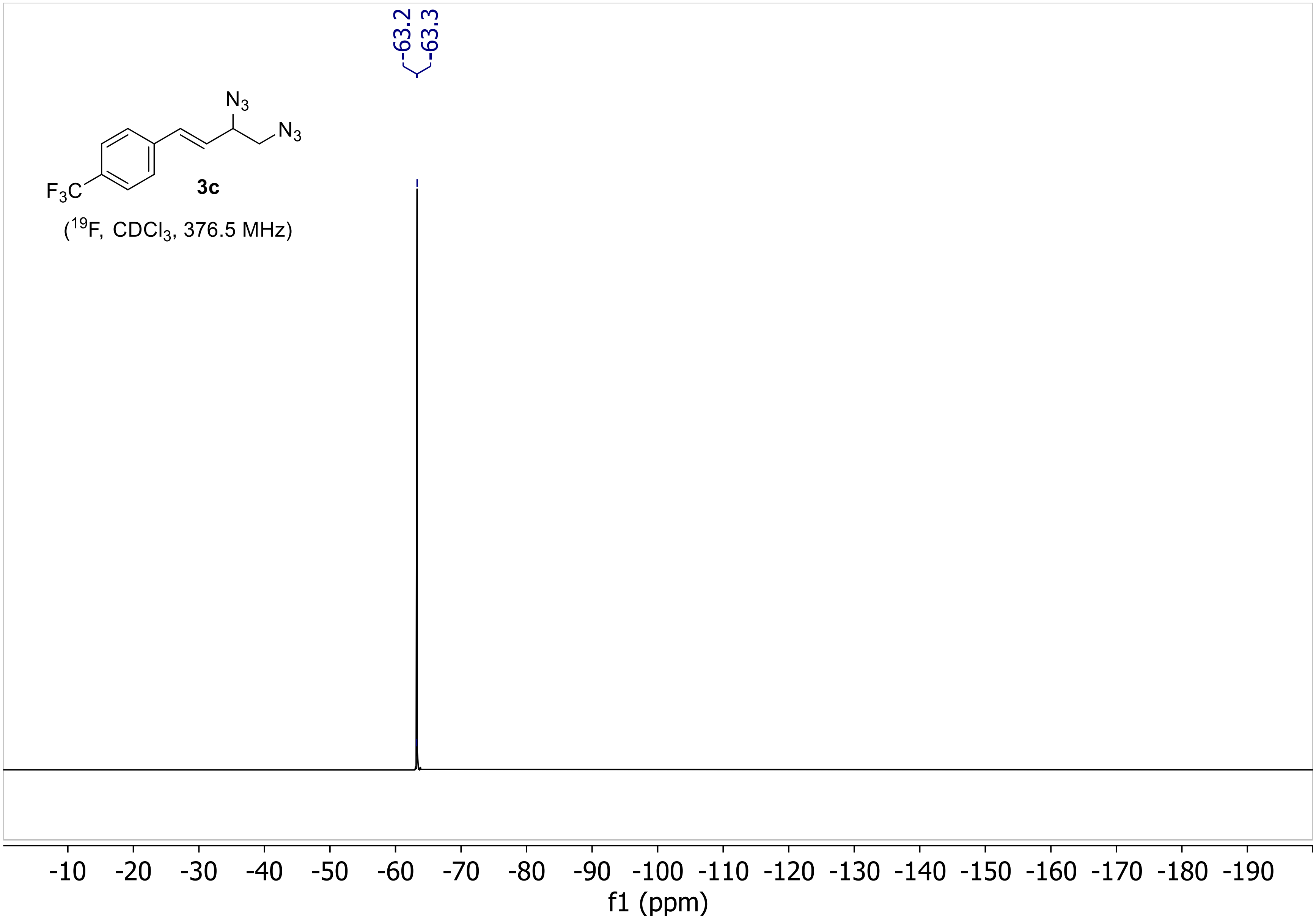
-63.2
-63.3
N_3
N_3
F_3C
3c
(^{19}F, $CDCl_3$, 376.5 MHz)
-10 -20 -30 -40 -50 -60 -70 -80 -90 -100 -110 -120 -130 -140 -150 -160 -170 -180 -190
f1 (ppm)

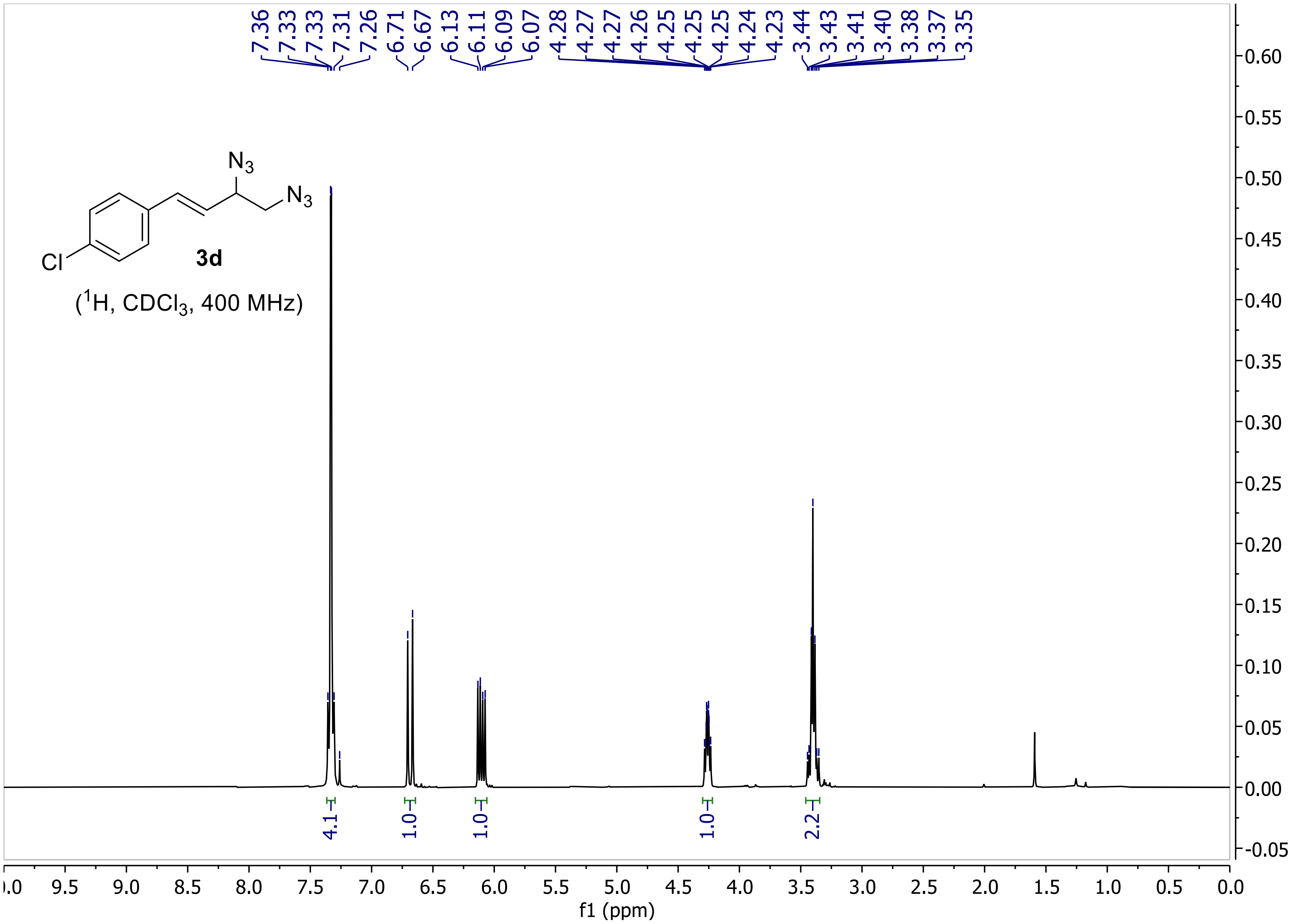

N3
N3
Cl
3d
(1H, CDCl3, 400 MHz)
7.36 7.33 7.33 7.31 7.26 6.71 6.67 6.13 6.11 6.09 6.07 4.28 4.27 4.27 4.26 4.25 4.25 4.25 4.24 4.23 3.44 3.43 3.41 3.40 3.38 3.37 3.35
4.1
1.0
1.0
1.0
2.2
f1 (ppm)

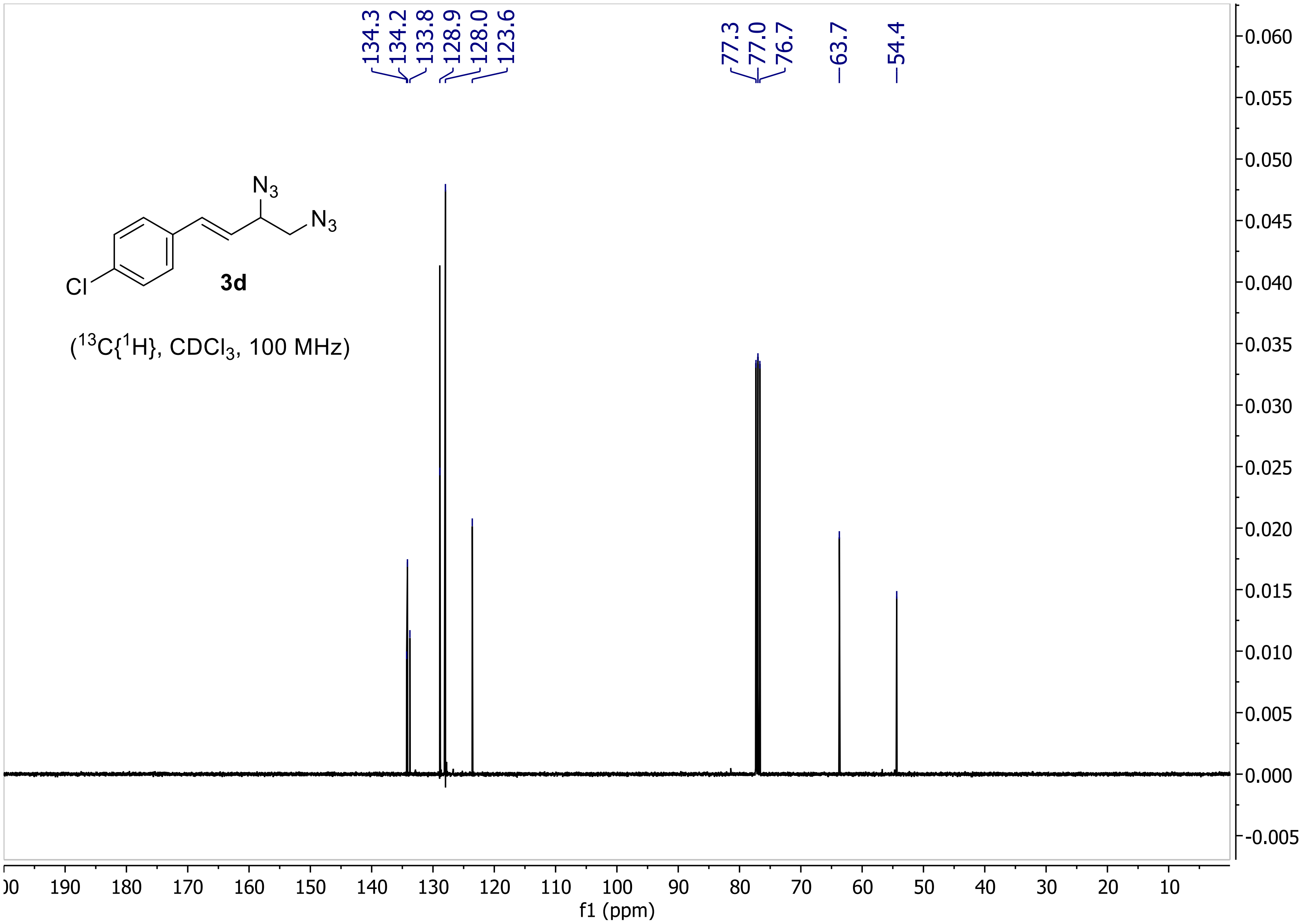
134.3
134.2
133.8
128.9
128.0
123.6
77.3
77.0
76.7
63.7
54.4
N3
N3
Cl
3d
(13C{1H}, CDCl3, 100 MHz)
f1 (ppm)

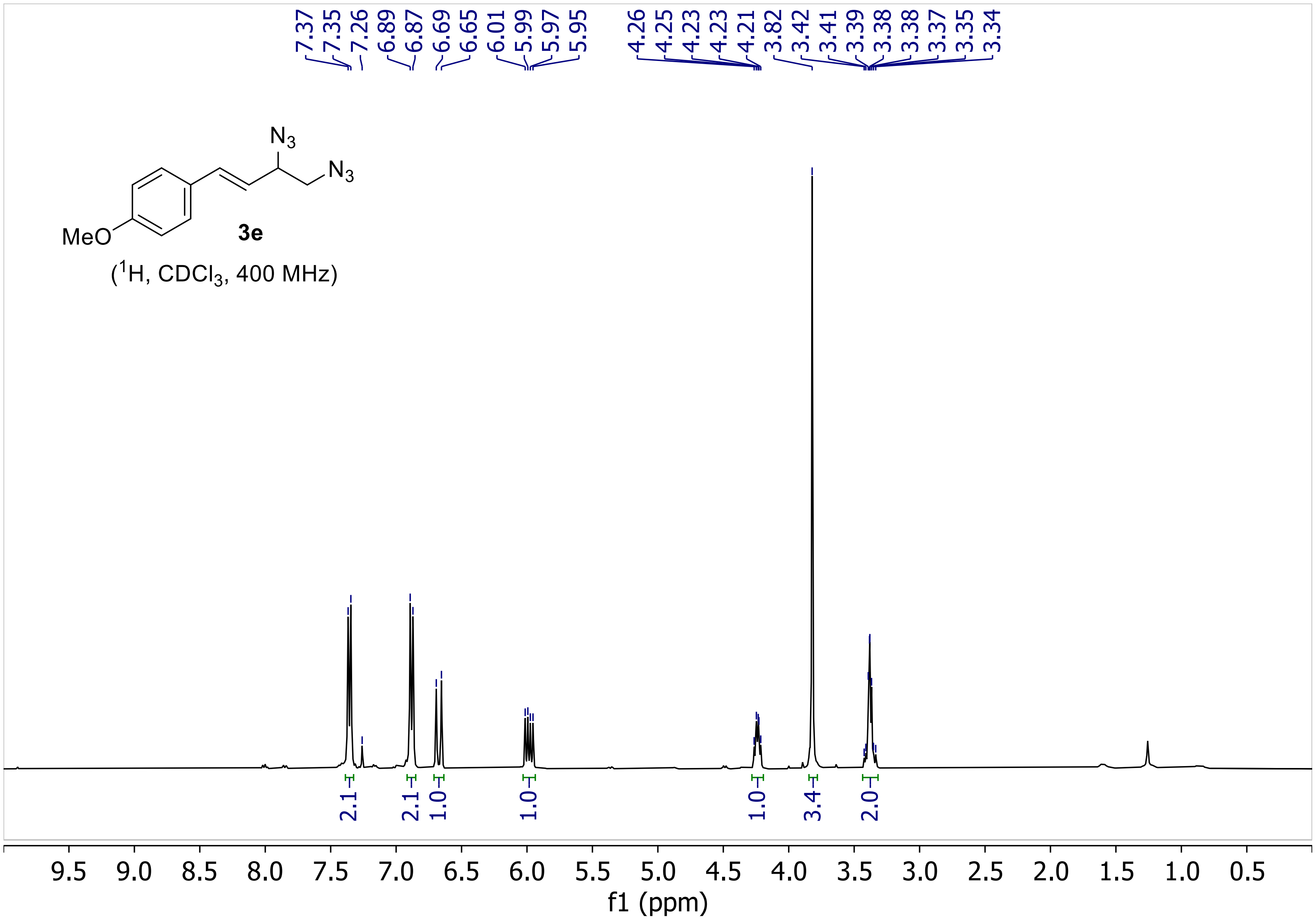
7.37
7.35
7.26
6.89
6.87
6.69
6.65
6.01
5.99
5.97
5.95
4.26
4.25
4.23
4.23
4.21
3.82
3.42
3.41
3.39
3.38
3.38
3.37
3.35
3.34
N3
N3
MeO
3e
(1H, CDCl3, 400 MHz)
2.1
2.1
1.0
1.0
1.0
3.4
2.0
9.5
9.0
8.5
8.0
7.5
7.0
6.5
6.0
5.5
5.0
4.5
4.0
3.5
3.0
2.5
2.0
1.5
1.0
0.5
f1 (ppm)

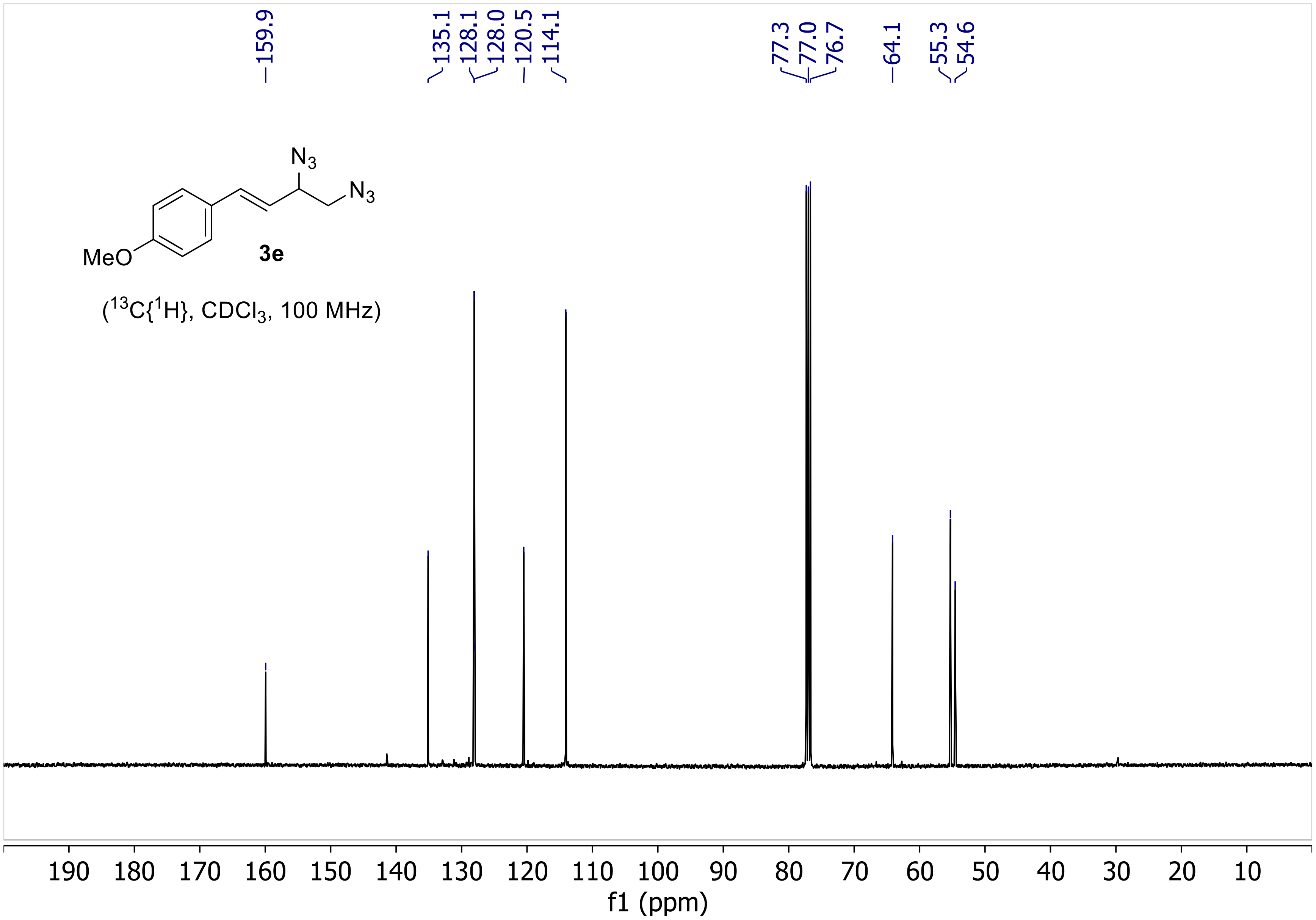
159.9
135.1
128.1
128.0
120.5
114.1
77.3
77.0
76.7
64.1
55.3
54.6
N3
N3
MeO
3e
(13C{1H}, CDCl3, 100 MHz)
190
180
170
160
150
140
130
120
110
100
90
80
70
60
50
40
30
20
10
f1 (ppm)

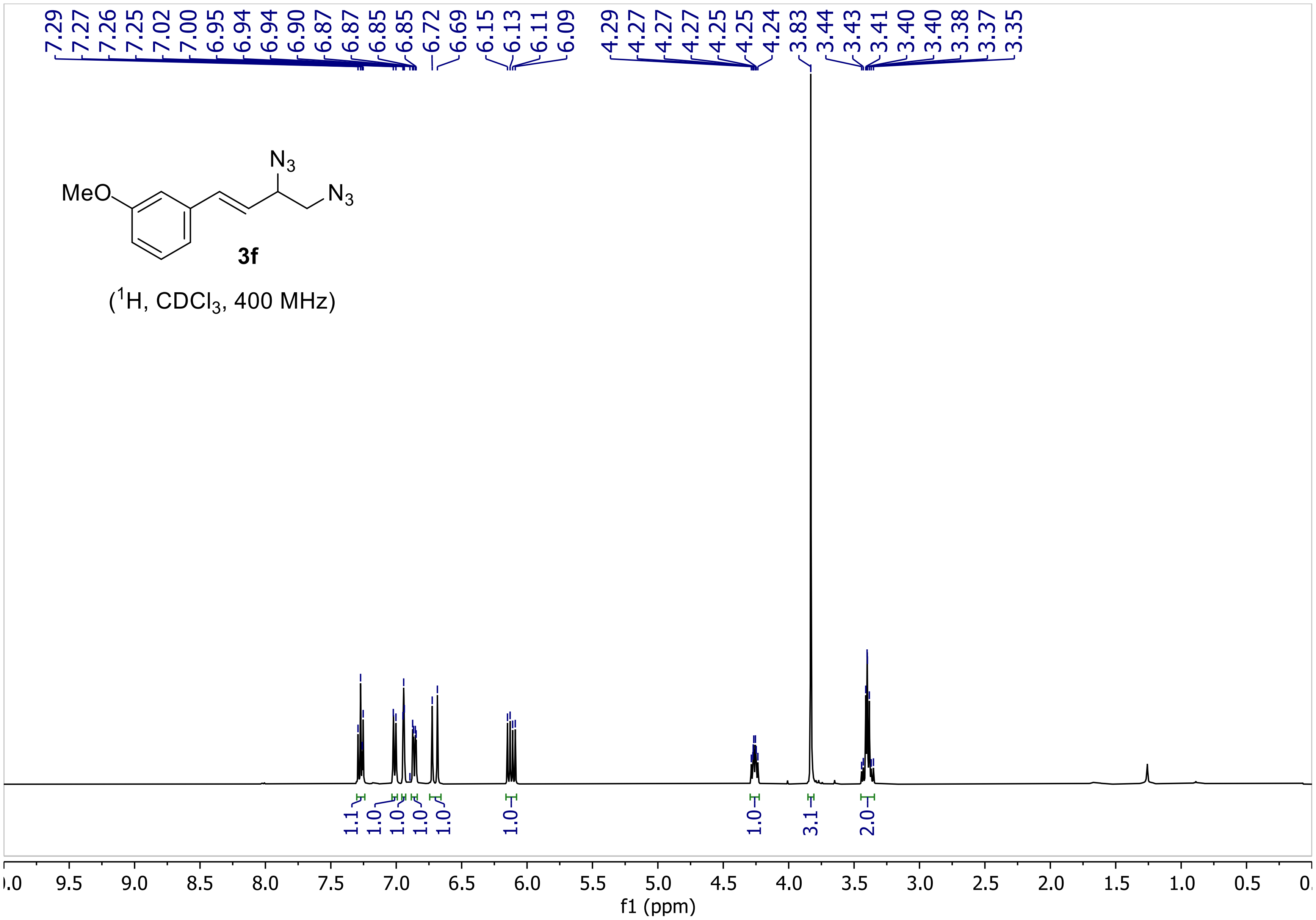
7.29 7.27 7.26 7.25 7.02 7.00 6.95 6.94 6.94 6.90 6.87 6.87 6.85 6.85 6.72 6.69 6.15 6.13 6.11 6.09 4.29 4.27 4.27 4.27 4.25 4.25 4.24 3.83 3.44 3.43 3.41 3.40 3.40 3.38 3.37 3.35
MeO
N3
N3
3f
(1H, CDCl3, 400 MHz)
1.1 1.0 1.0 1.0 1.0 1.0 1.0 3.1 2.0
9.5 9.0 8.5 8.0 7.5 7.0 6.5 6.0 5.5 5.0 4.5 4.0 3.5 3.0 2.5 2.0 1.5 1.0 0.5
f1 (ppm)

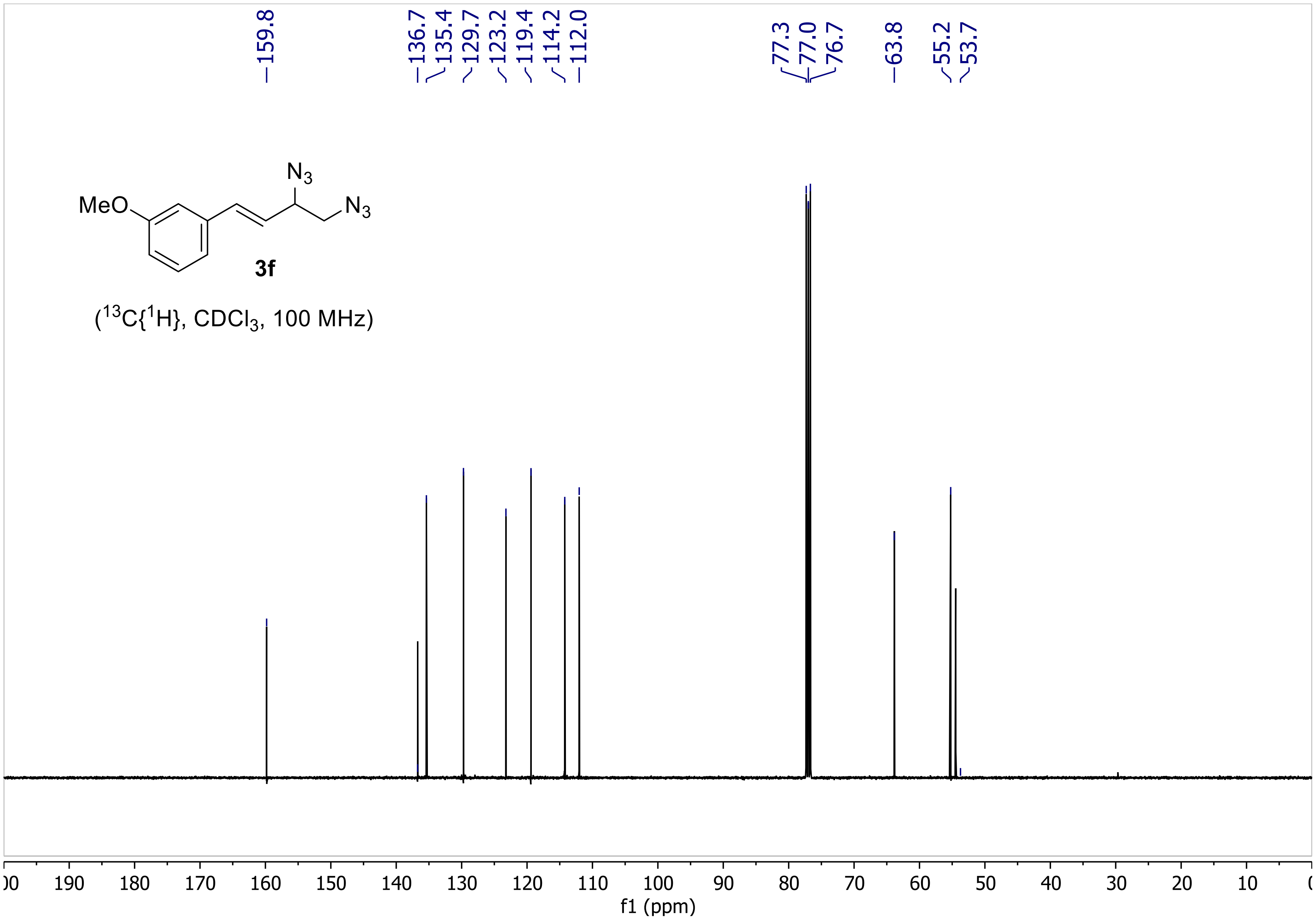
159.8
136.7
135.4
129.7
123.2
119.4
114.2
112.0
77.3
77.0
76.7
63.8
55.2
53.7
MeO
N3
N3
3f
(13C{1H}, CDCl3, 100 MHz)
190
180
170
160
150
140
130
120
110
100
90
80
70
60
50
40
30
20
10
f1 (ppm)

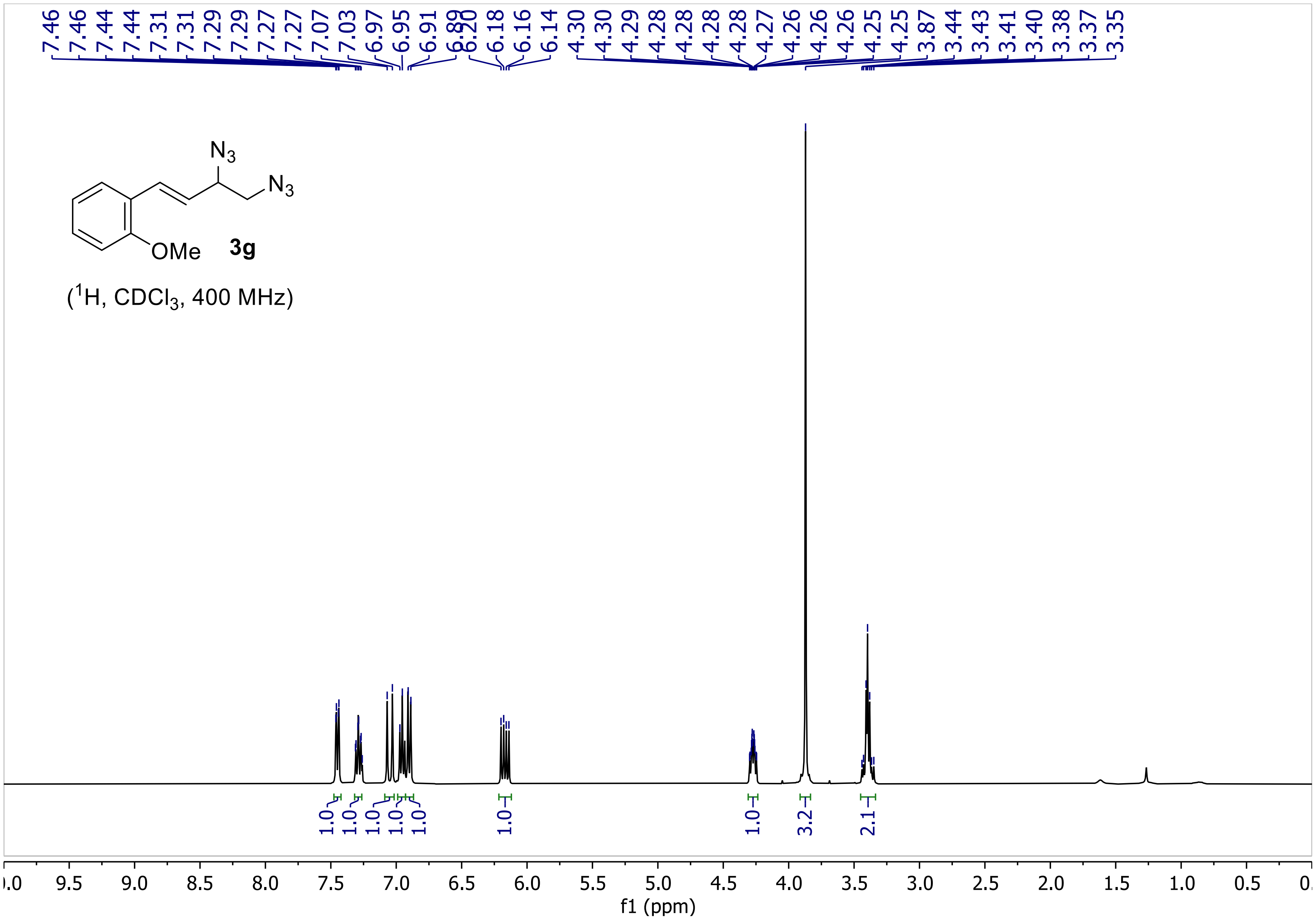

7.46 7.46 7.44 7.44 7.31 7.31 7.29 7.29 7.27 7.27 7.07 7.03 6.97 6.95 6.91 6.89 6.20 6.18 6.16 6.14 4.30 4.30 4.29 4.28 4.28 4.28 4.28 4.27 4.26 4.26 4.26 4.25 4.25 3.87 3.44 3.43 3.41 3.40 3.38 3.37 3.35
N3
N3
OMe
3g
(1H, CDCl3, 400 MHz)
1.0
1.0
1.0
1.0
1.0
1.0
1.0
3.2
2.1
f1 (ppm)

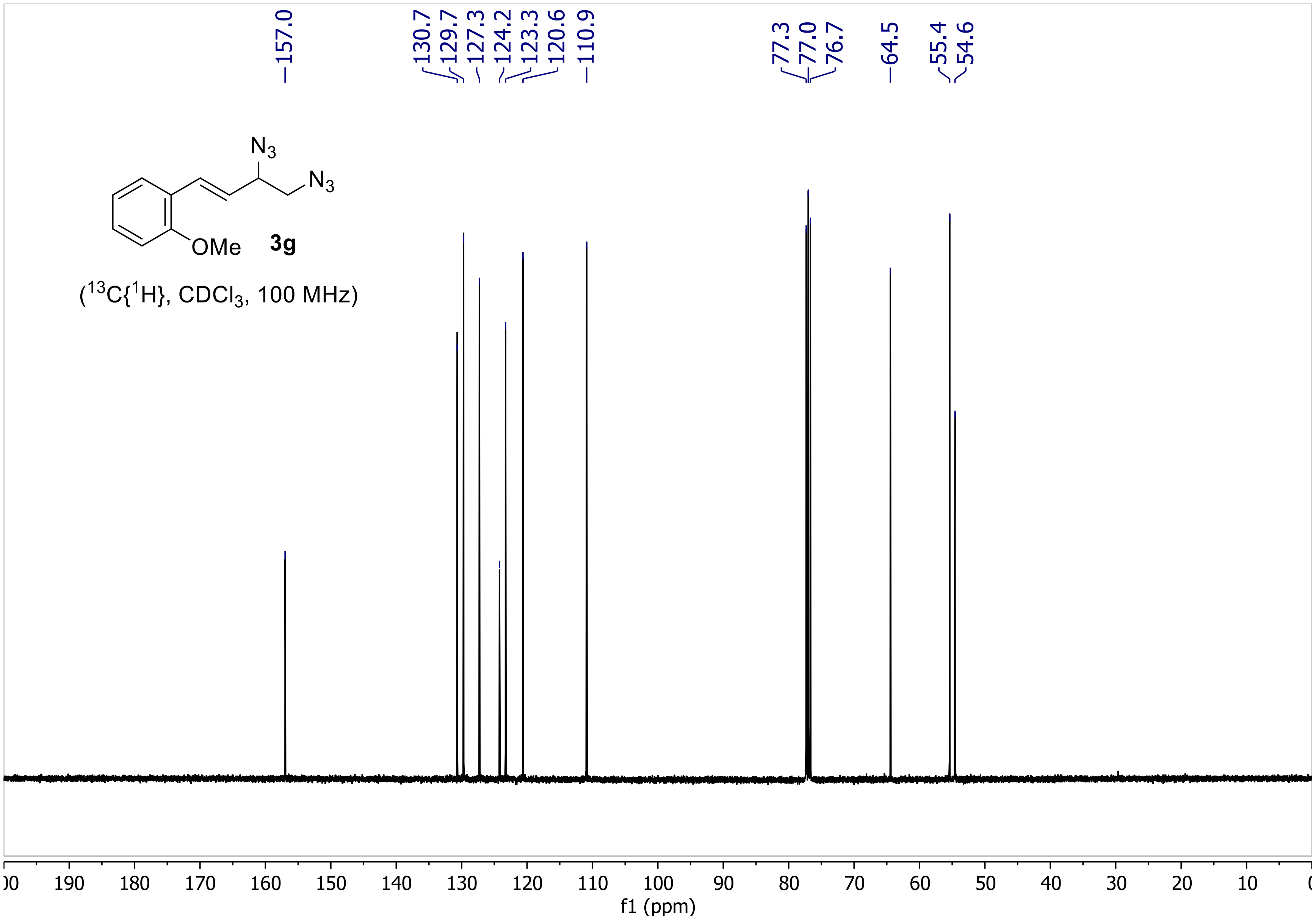
N3
N3
OMe
3g
(13C{1H}, CDCl3, 100 MHz)
157.0
130.7
129.7
127.3
124.2
123.3
120.6
110.9
77.3
77.0
76.7
64.5
55.4
54.6
f1 (ppm)

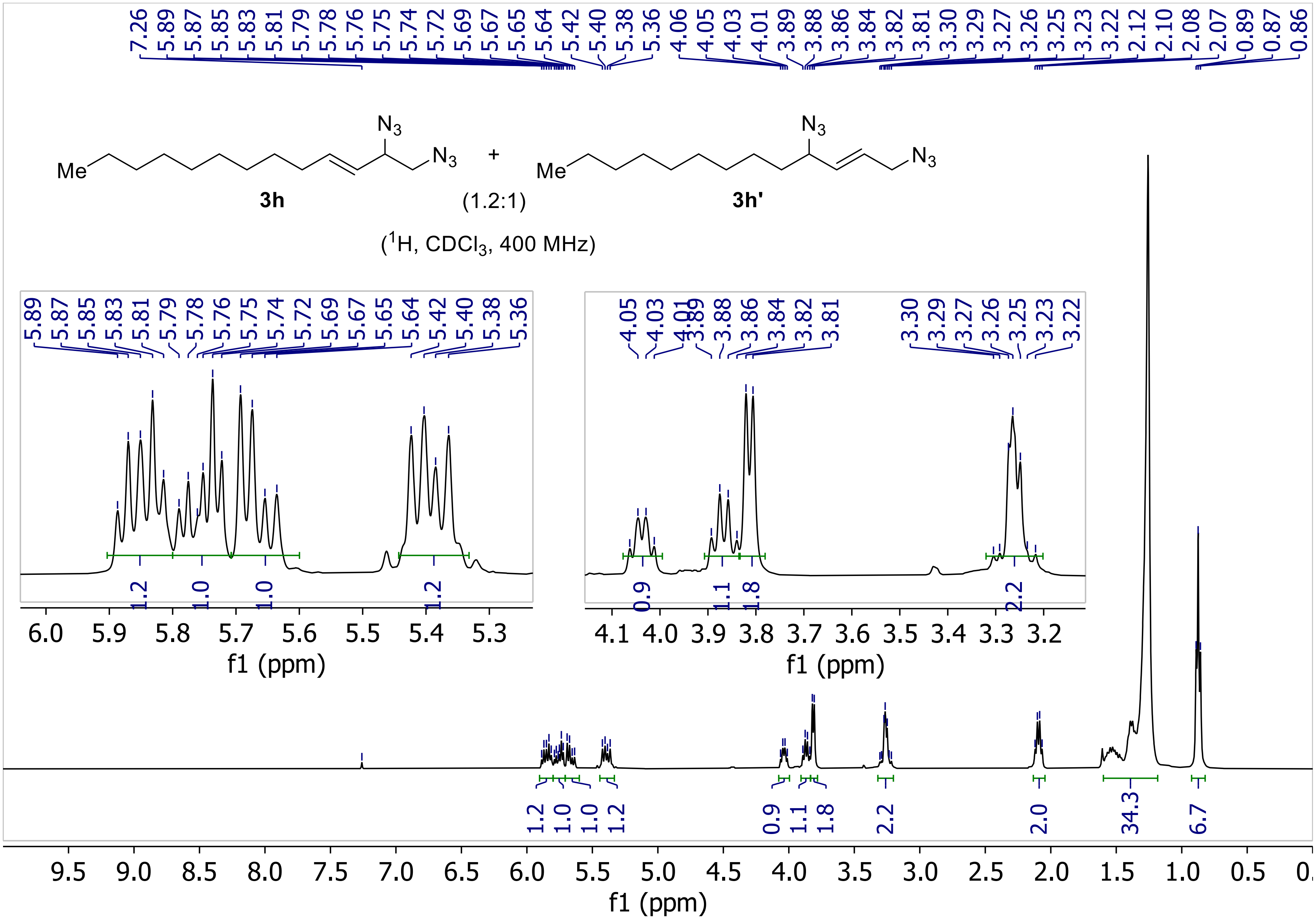
N3
Me
N3
3h
+
(1.2:1)
N3
Me
N3
3h'
(1H, CDCl3, 400 MHz)
f1 (ppm)
f1 (ppm)
f1 (ppm)

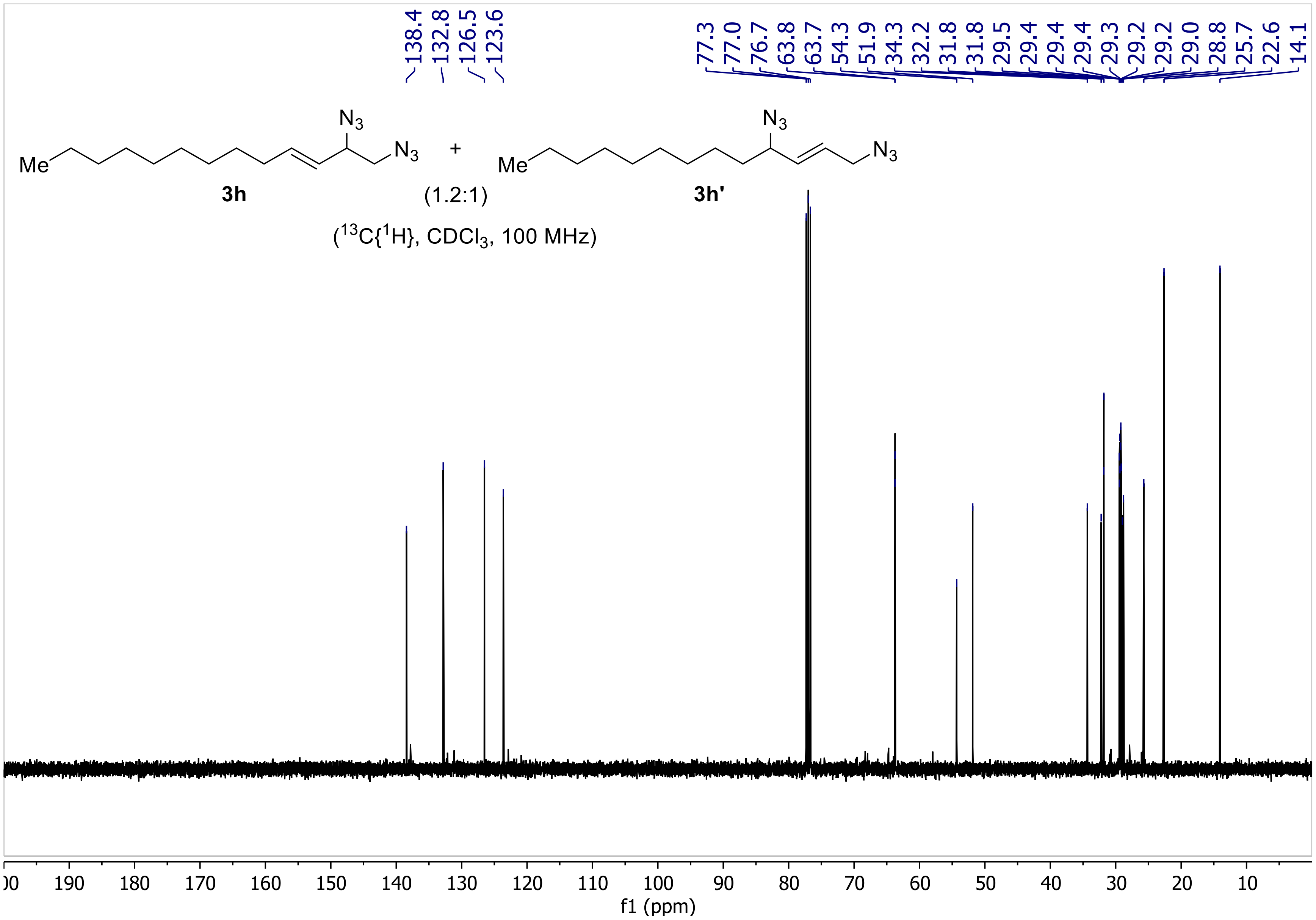
138.4
132.8
126.5
123.6
77.3
77.0
76.7
63.8
63.7
54.3
51.9
34.3
32.2
31.8
31.8
29.5
29.4
29.4
29.4
29.3
29.2
29.2
29.0
28.8
25.7
22.6
14.1
N3
Me
N3
+
3h
(1.2:1)
3h'
($^{13}C\{^1H\}$, $CDCl_3$, 100 MHz)
190
180
170
160
150
140
130
120
110
100
90
80
70
60
50
40
30
20
10
f1 (ppm)

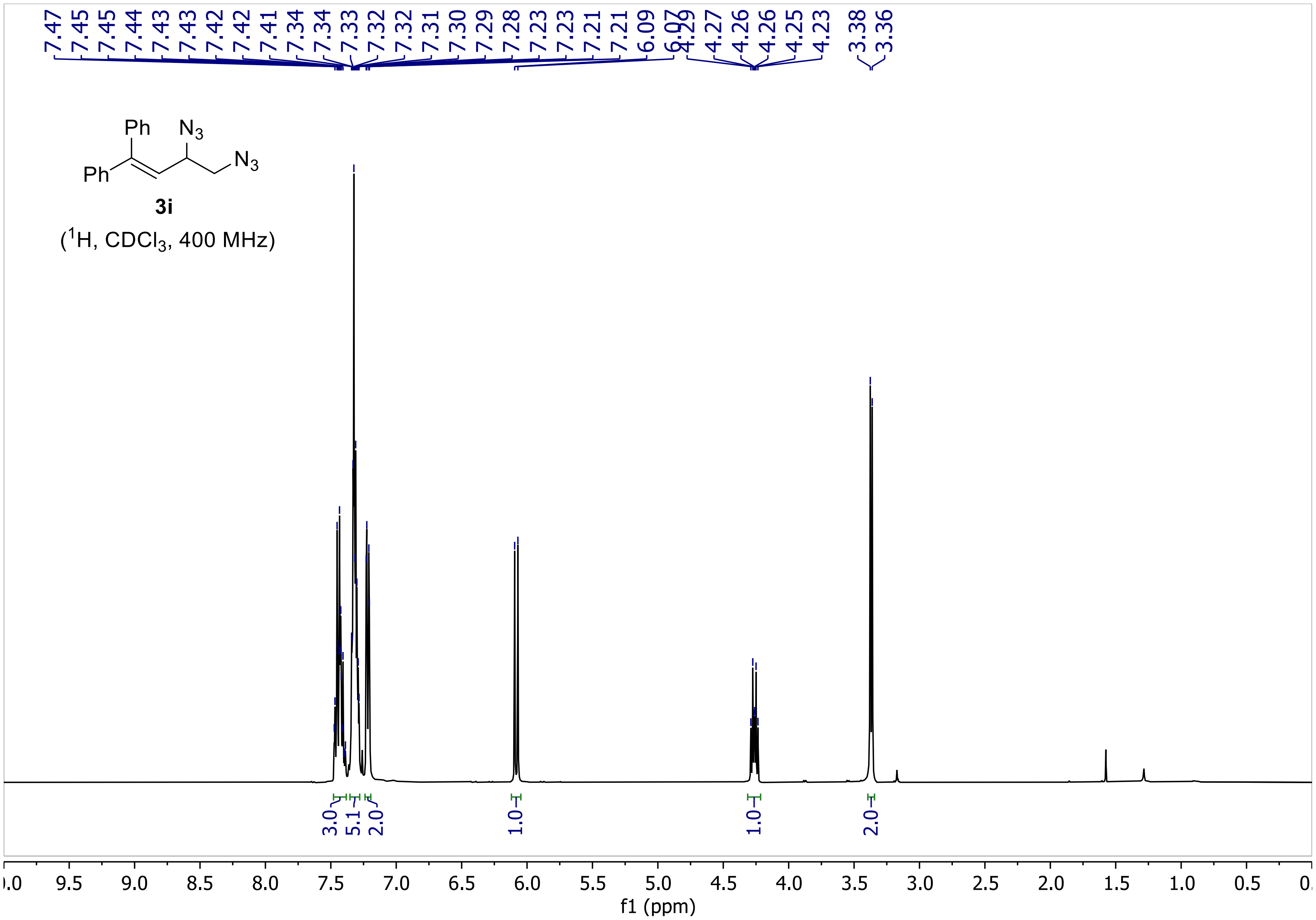
7.47
7.45
7.45
7.44
7.43
7.43
7.42
7.42
7.41
7.34
7.34
7.33
7.32
7.32
7.31
7.30
7.29
7.28
7.23
7.23
7.21
7.21
6.09
6.07
4.29
4.27
4.26
4.26
4.25
4.23
3.38
3.36
Ph
N3
Ph
N3
3i
(1H, CDCl3, 400 MHz)
3.0
5.1
2.0
1.0
1.0
2.0
f1 (ppm)

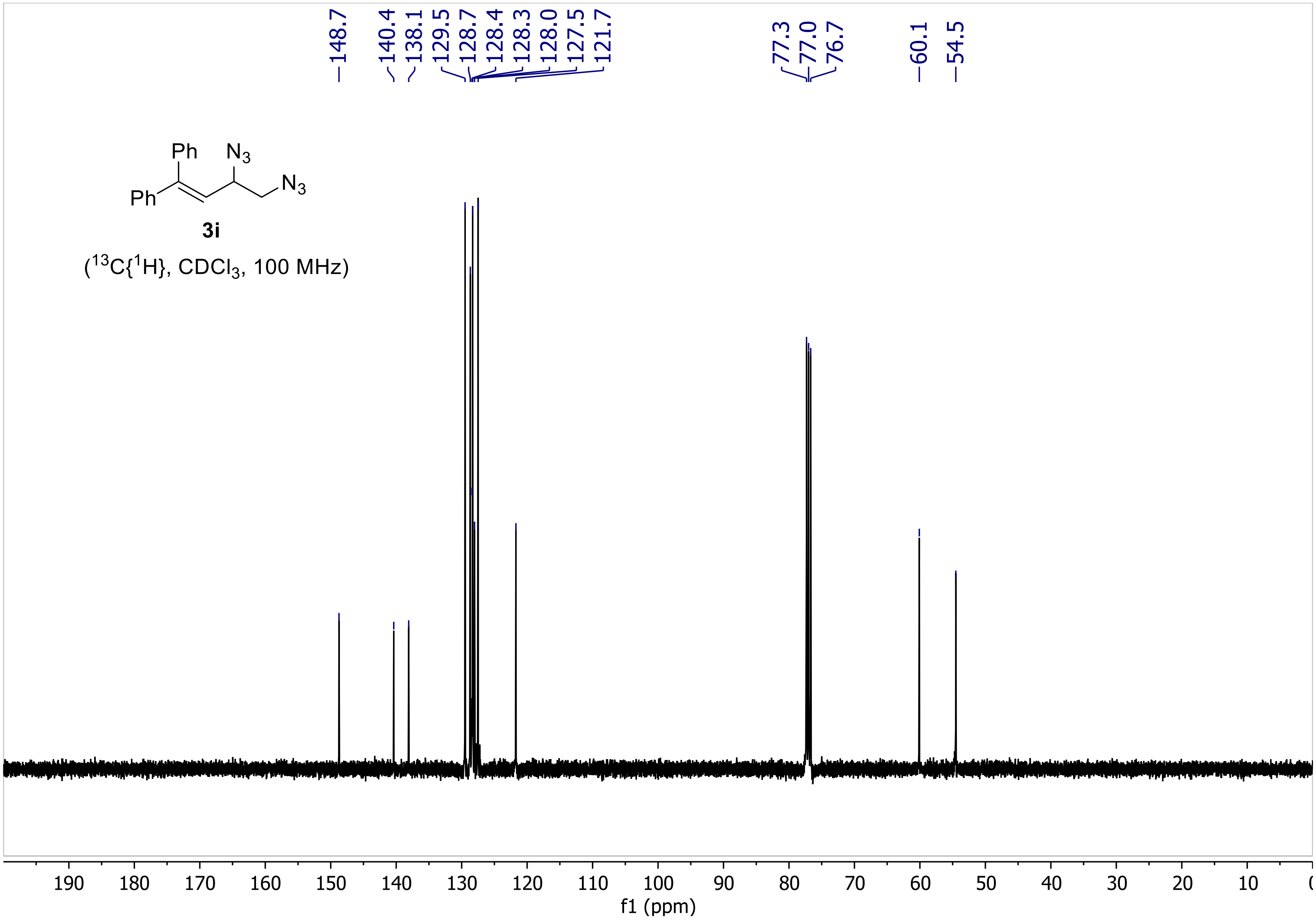
148.7
140.4
138.1
129.5
128.7
128.4
128.3
128.0
127.5
121.7
77.3
77.0
76.7
60.1
54.5
Ph
N3
Ph
N3
3i
(13C{1H}, CDCl3, 100 MHz)
190
180
170
160
150
140
130
120
110
100
90
80
70
60
50
40
30
20
10
f1 (ppm)

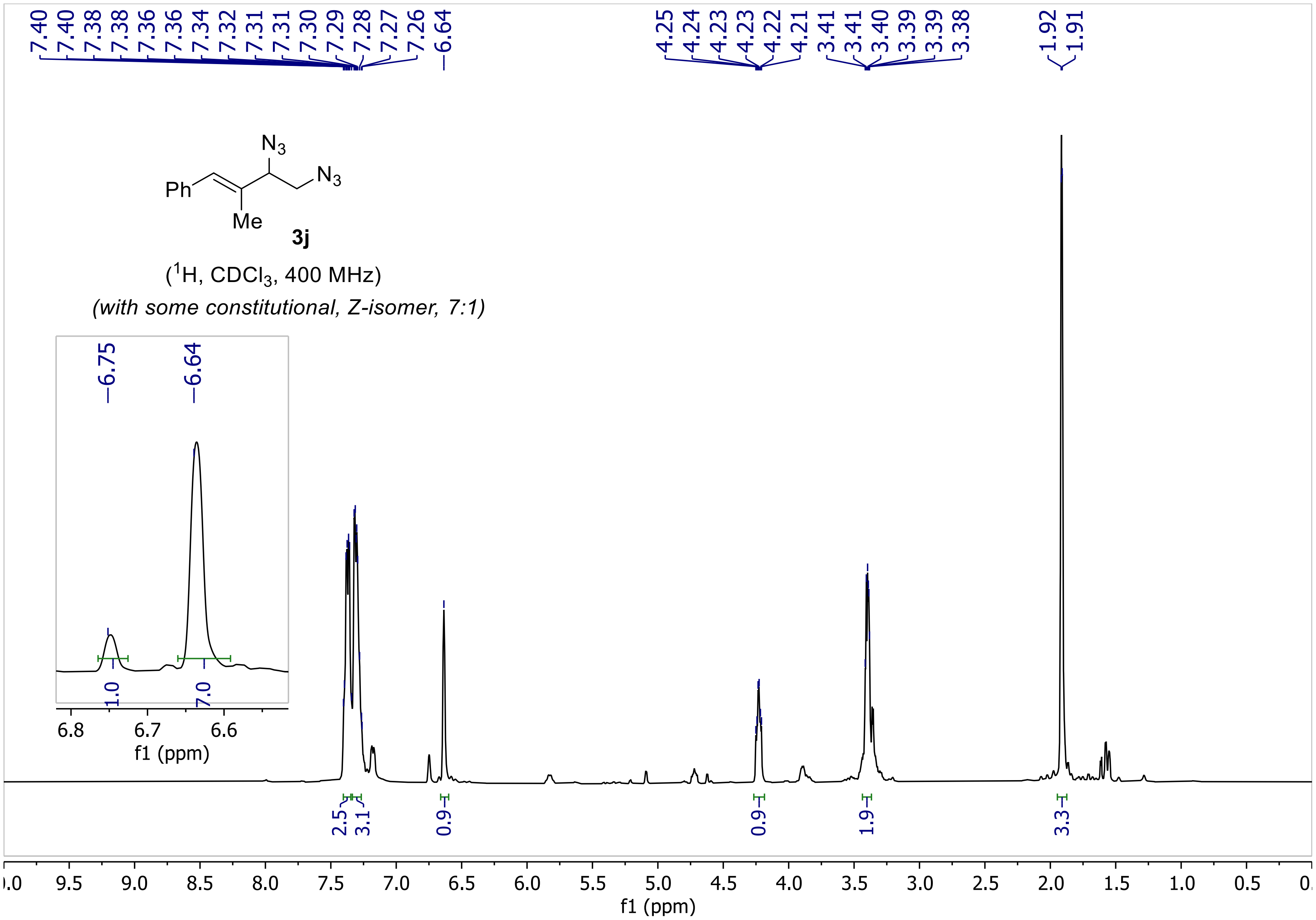

N3
N3
Ph
Me
3j
(1H, CDCl3, 400 MHz)
(with some constitutional, Z-isomer, 7:1)
7.40 7.40 7.38 7.38 7.36 7.36 7.34 7.32 7.31 7.31 7.30 7.29 7.28 7.27 7.26 6.64
4.25 4.24 4.23 4.23 4.22 4.21 3.41 3.41 3.40 3.39 3.39 3.38
1.92 1.91
6.75
6.64
1.0
7.0
6.8 6.7 6.6
f1 (ppm)
2.5 3.1 0.9 0.9 1.9 3.3
f1 (ppm)

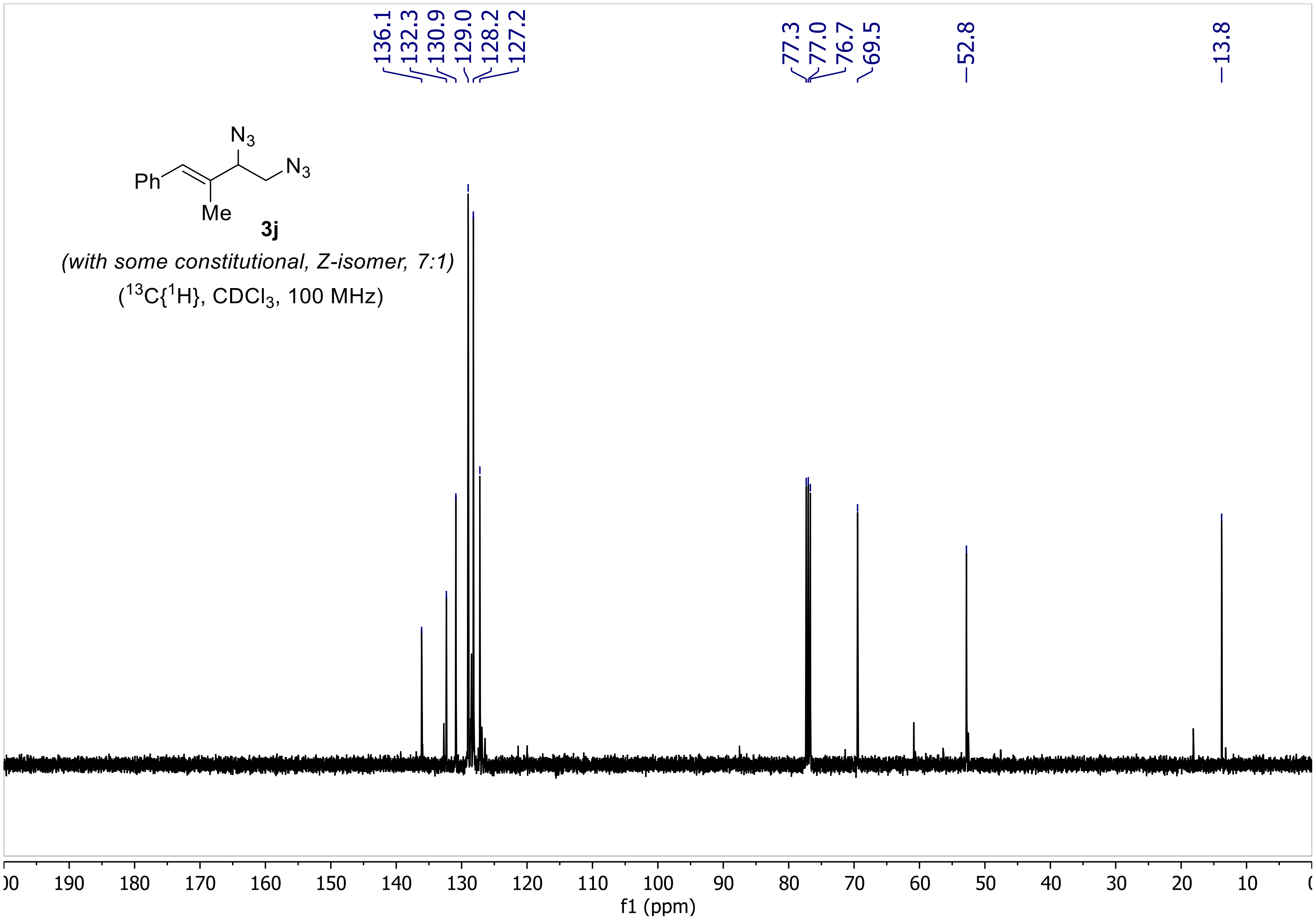
136.1
132.3
130.9
129.0
128.2
127.2
77.3
77.0
76.7
69.5
52.8
13.8
N3
N3
Ph
Me
3j
(with some constitutional, Z-isomer, 7:1)
(13C{1H}, CDCl3, 100 MHz)
190
180
170
160
150
140
130
120
110
100
90
80
70
60
50
40
30
20
10
f1 (ppm)

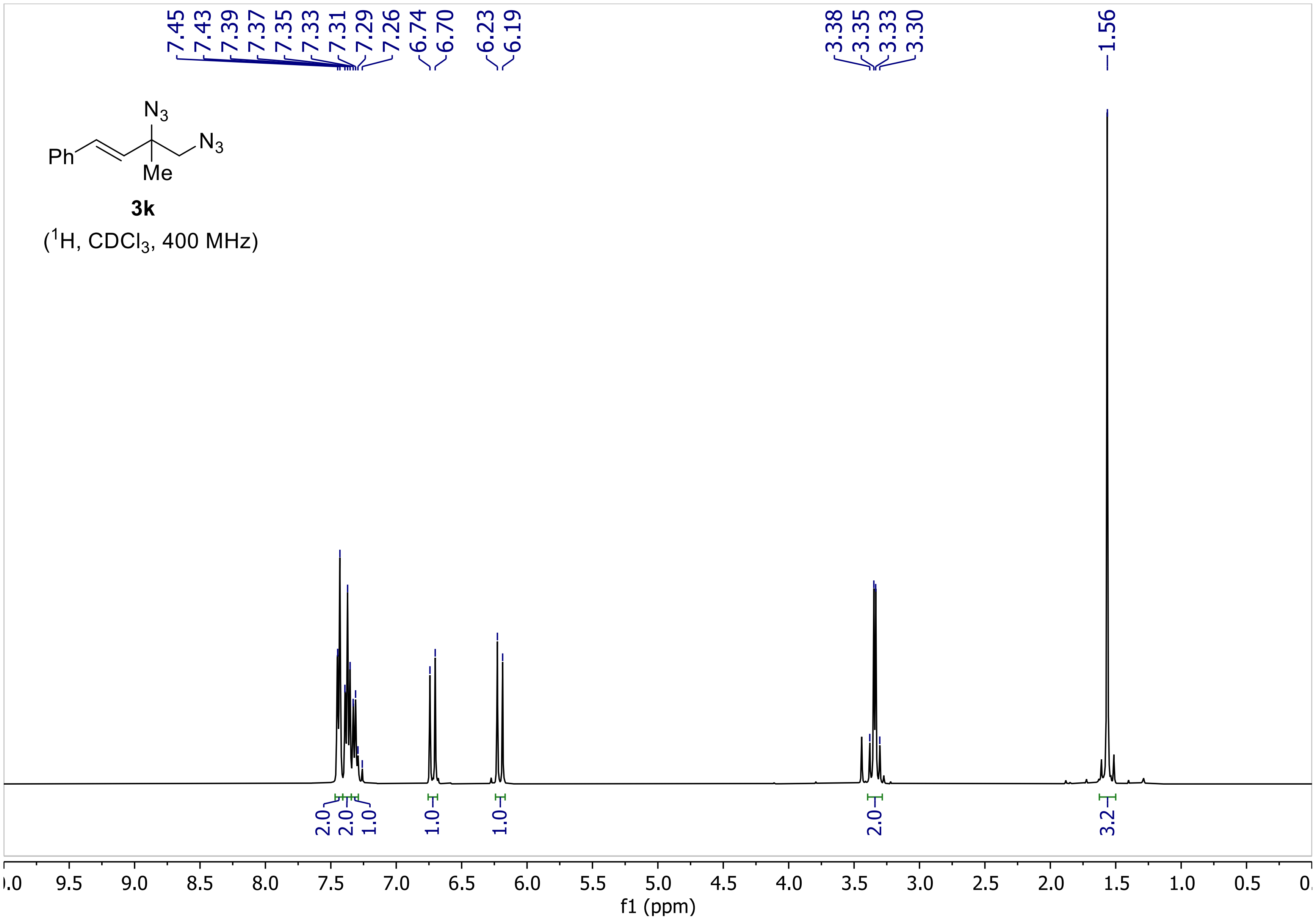
7.45
7.43
7.39
7.37
7.35
7.33
7.31
7.29
7.26
6.74
6.70
6.23
6.19
3.38
3.35
3.33
3.30
1.56
N3
N3
Ph
Me
3k
(1H, CDCl3, 400 MHz)
2.0
2.0
1.0
1.0
1.0
2.0
3.2
f1 (ppm)

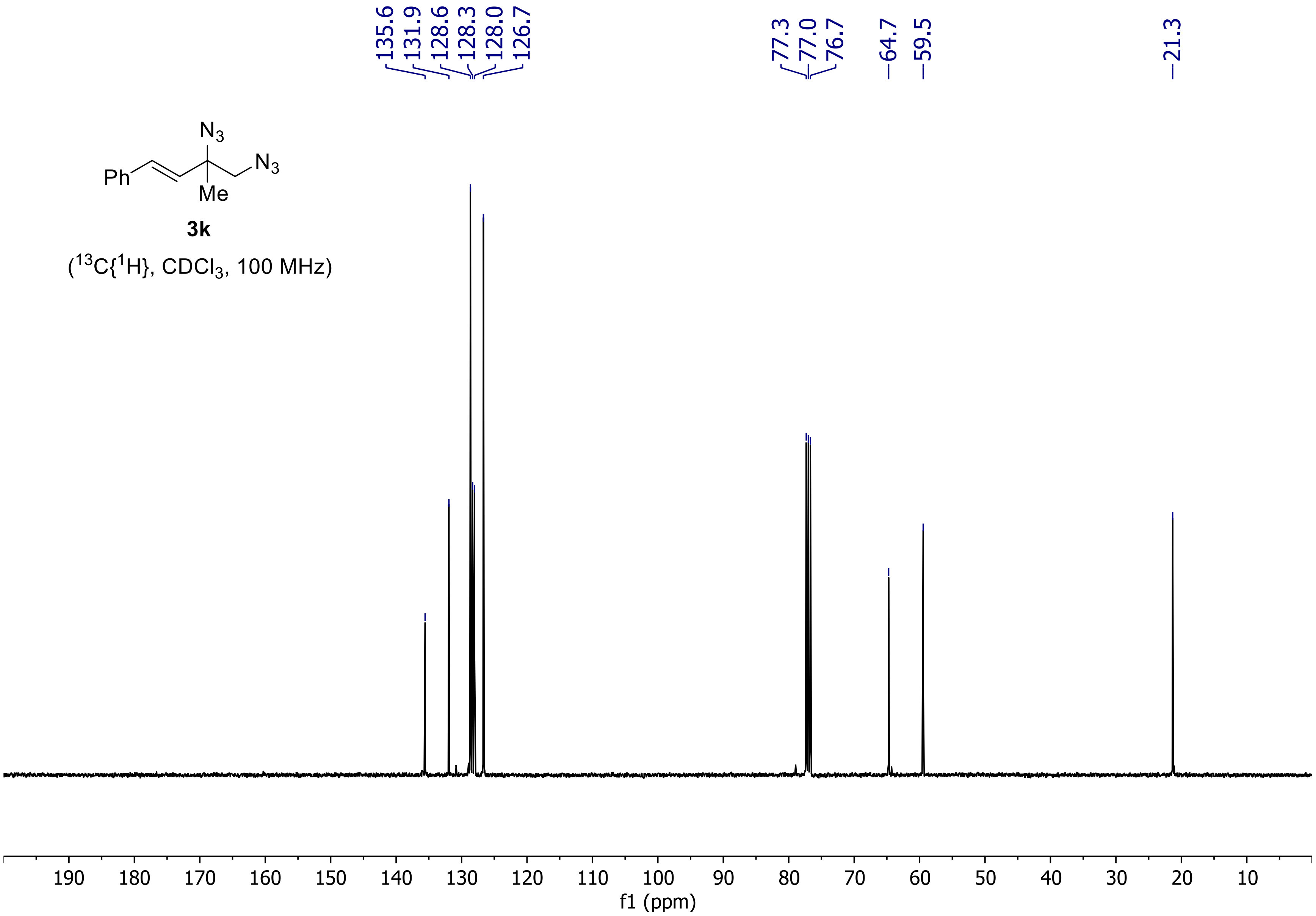

3k

($^{13}C\{^1H\}$, $CDCl_3$, 100 MHz)

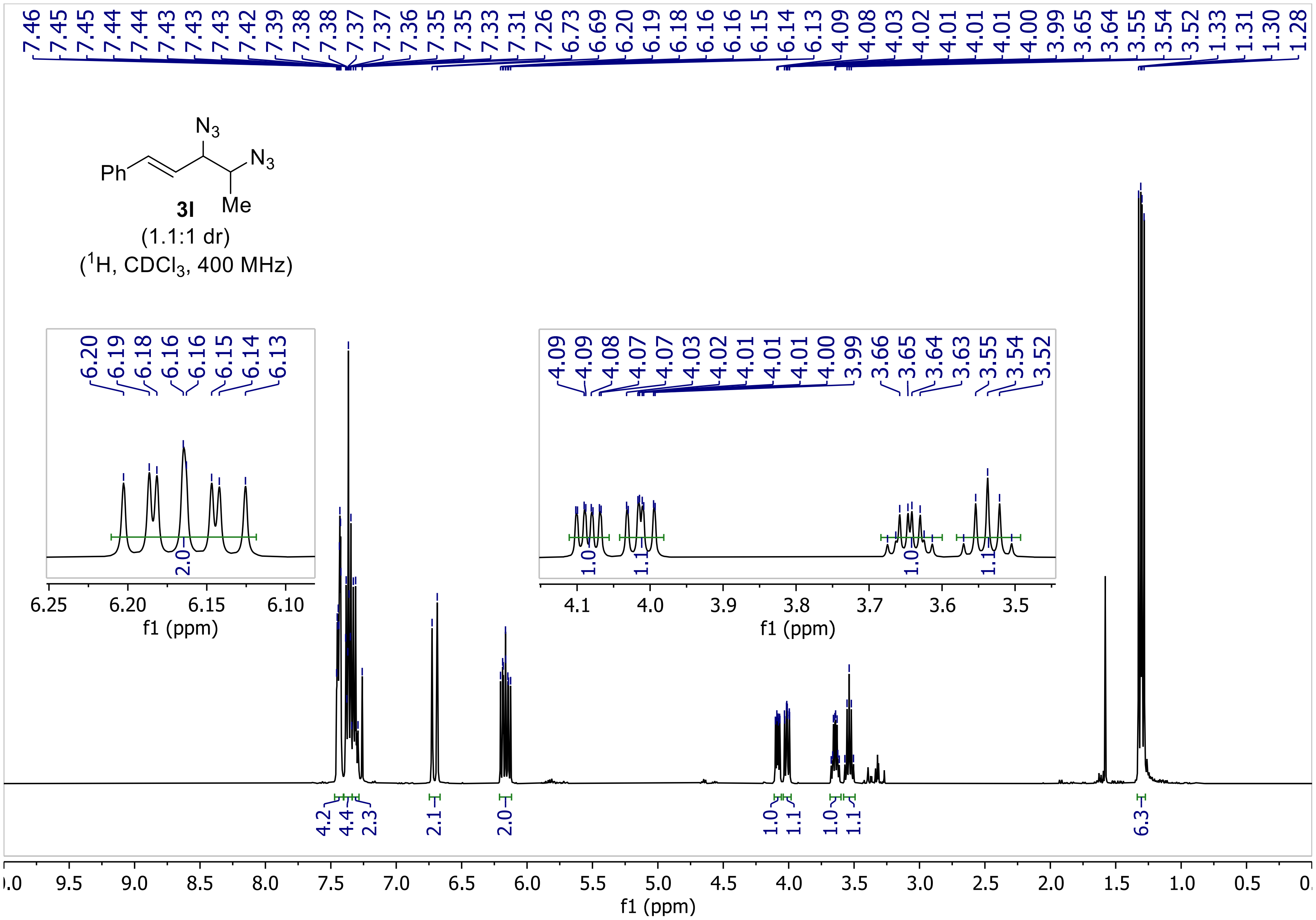
7.46 7.45 7.45 7.44 7.44 7.43 7.43 7.43 7.42 7.39 7.38 7.38 7.37 7.37 7.36 7.35 7.35 7.33 7.31 7.26 6.73 6.69 6.20 6.19 6.18 6.16 6.16 6.15 6.14 6.13 4.09 4.08 4.03 4.02 4.01 4.01 4.01 4.00 3.99 3.65 3.64 3.55 3.54 3.52 1.33 1.31 1.30 1.28
N3
N3
Ph
Me
3l
(1.1:1 dr)
(1H, CDCl3, 400 MHz)
6.20 6.19 6.18 6.16 6.16 6.15 6.14 6.13
2.0
4.09 4.09 4.08 4.07 4.07 4.03 4.02 4.01 4.01 4.01 4.00 3.99 3.66 3.65 3.64 3.63 3.55 3.54 3.52
1.0 1.1 1.0 1.1
f1 (ppm)
4.2 4.4 2.3 2.1 2.0 1.0 1.1 1.0 1.1 6.3
f1 (ppm)

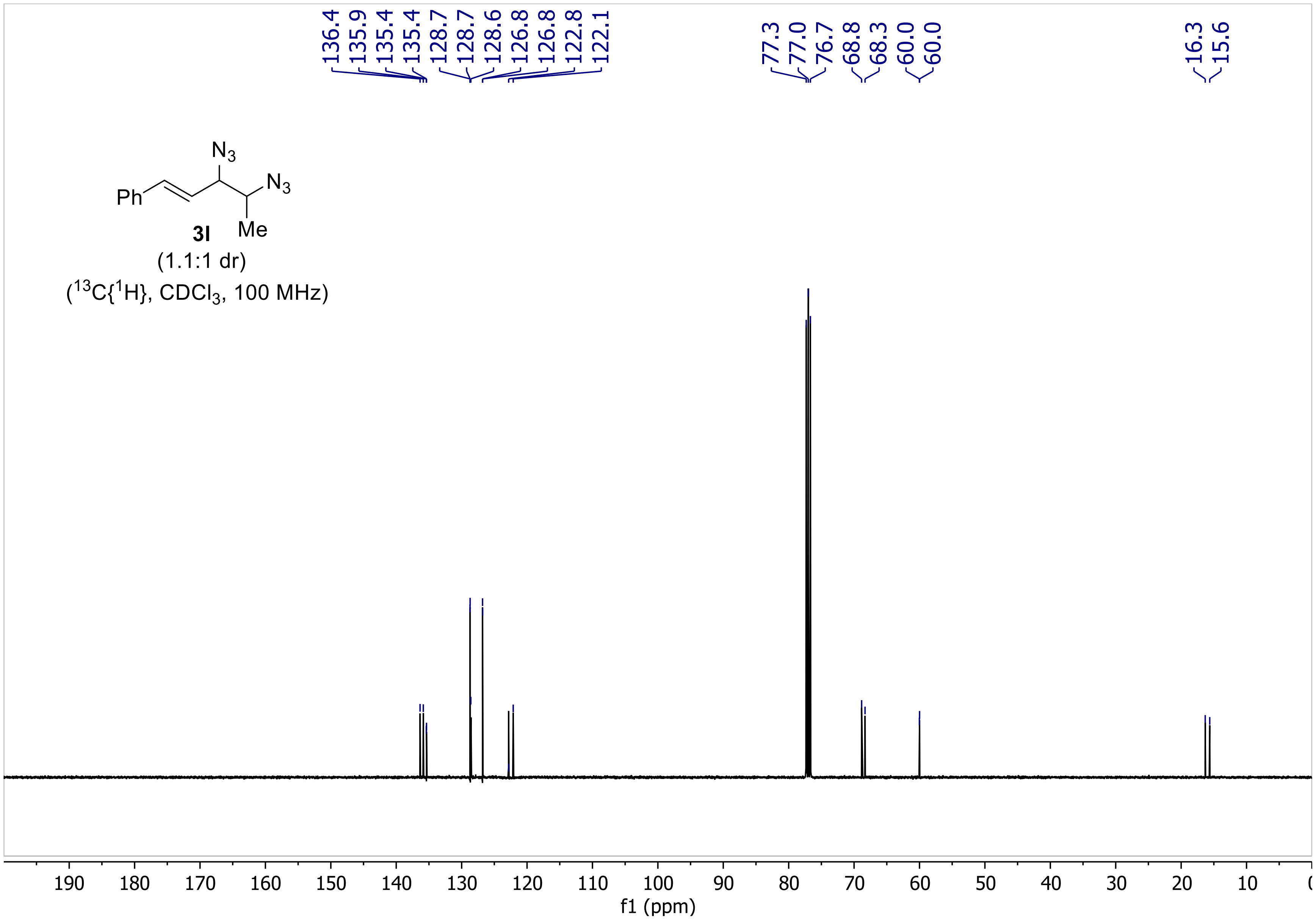
136.4
135.9
135.4
135.4
128.7
128.7
128.6
126.8
126.8
122.8
122.1
77.3
77.0
76.7
68.8
68.3
60.0
60.0
16.3
15.6
N3
Ph
N3
3l
Me
(1.1:1 dr)
(13C{1H}, CDCl3, 100 MHz)
190
180
170
160
150
140
130
120
110
100
90
80
70
60
50
40
30
20
10
f1 (ppm)

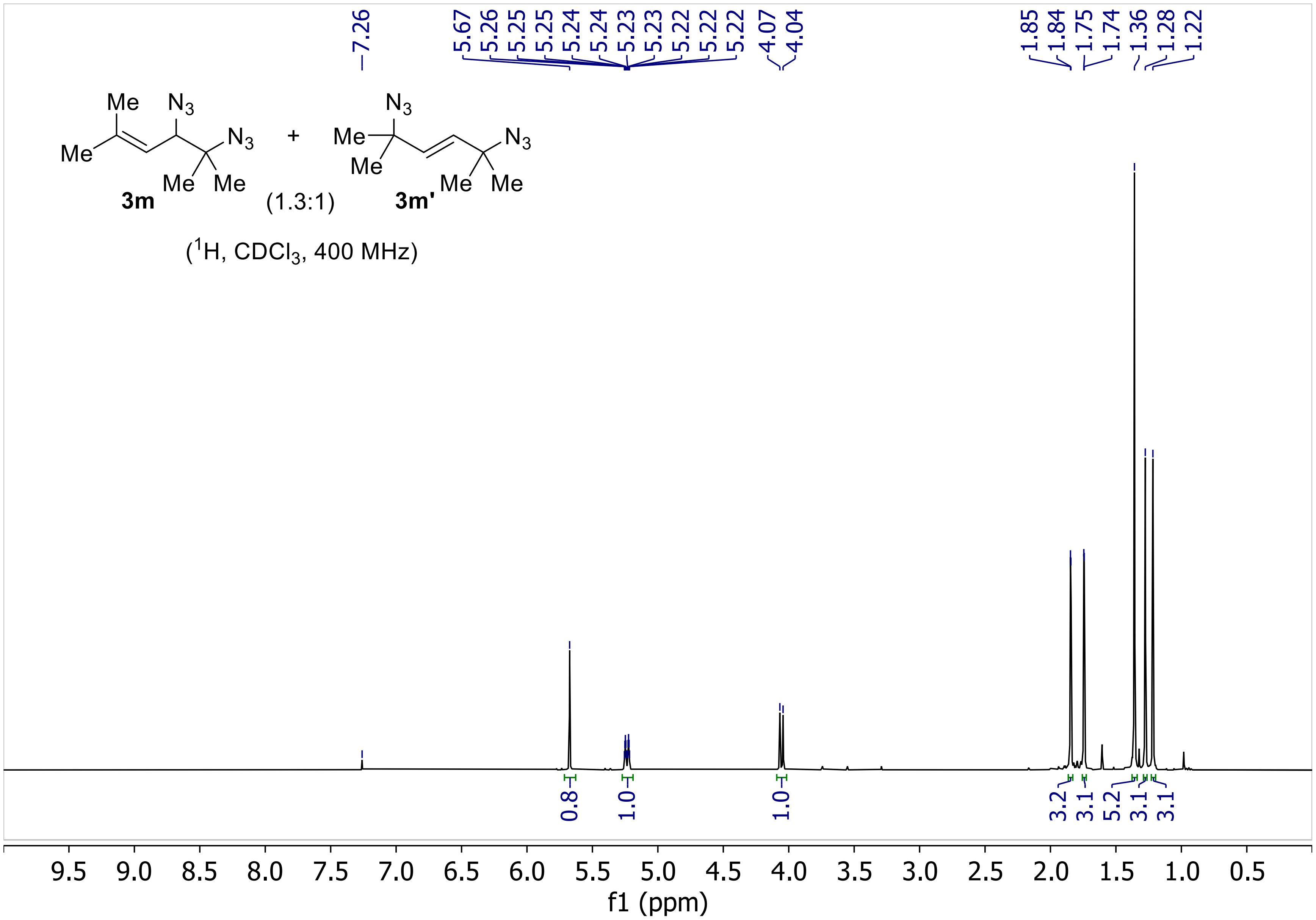

3m
(1.3:1)
3m'
(1H, CDCl3, 400 MHz)
7.26
5.67 5.26 5.25 5.25 5.24 5.24 5.23 5.23 5.22 5.22 5.22 4.07 4.04
1.85 1.84 1.75 1.74 1.36 1.28 1.22
0.8 1.0 1.0 3.2 3.1 5.2 3.1 3.1
f1 (ppm)

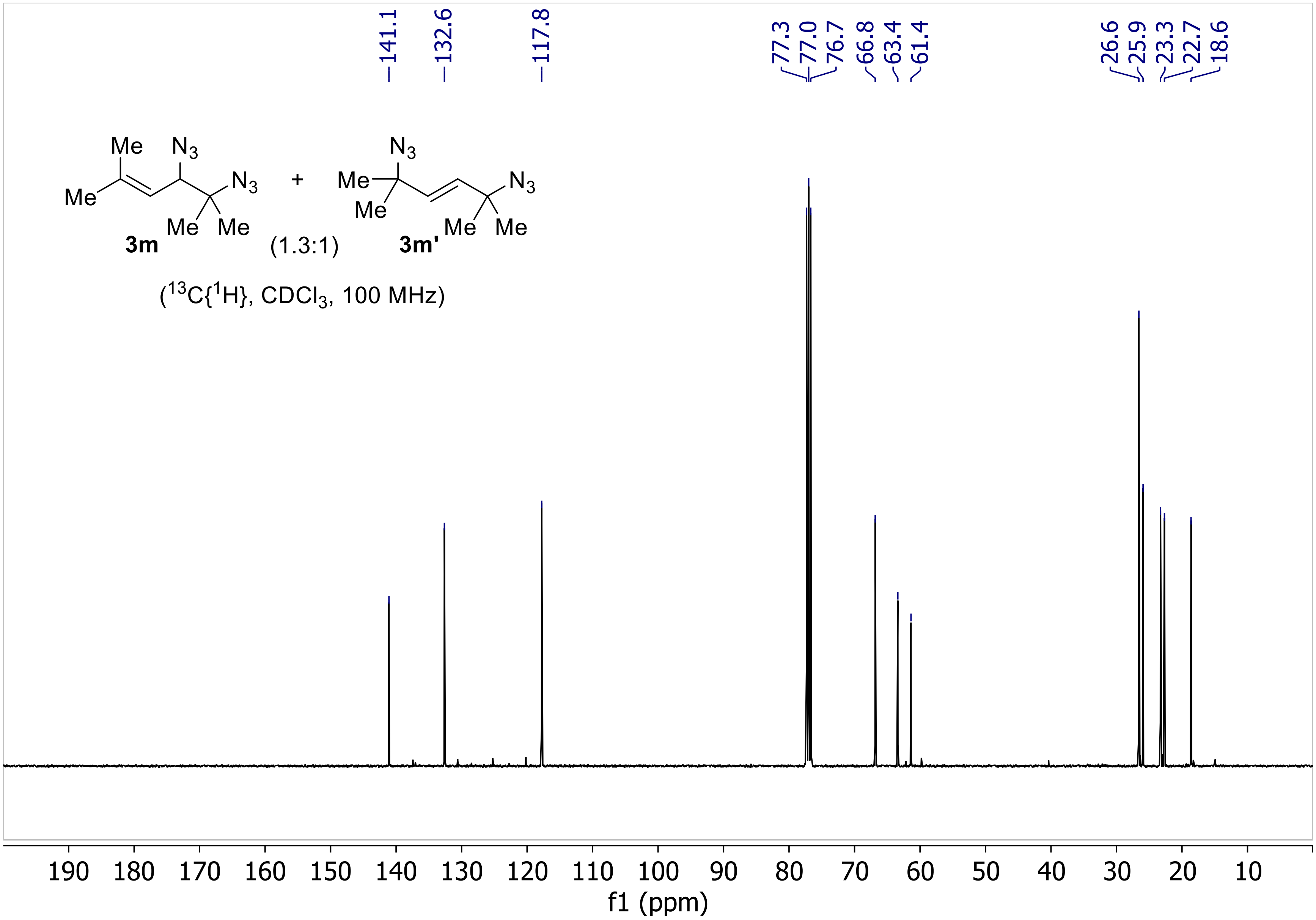
141.1
132.6
117.8
77.3
77.0
76.7
66.8
63.4
61.4
26.6
25.9
23.3
22.7
18.6
Me
N3
N3
Me
Me
Me
3m
+
N3
Me
Me
N3
Me
Me
3m'
(1.3:1)
(13C{1H}, CDCl3, 100 MHz)
190 180 170 160 150 140 130 120 110 100 90 80 70 60 50 40 30 20 10
f1 (ppm)

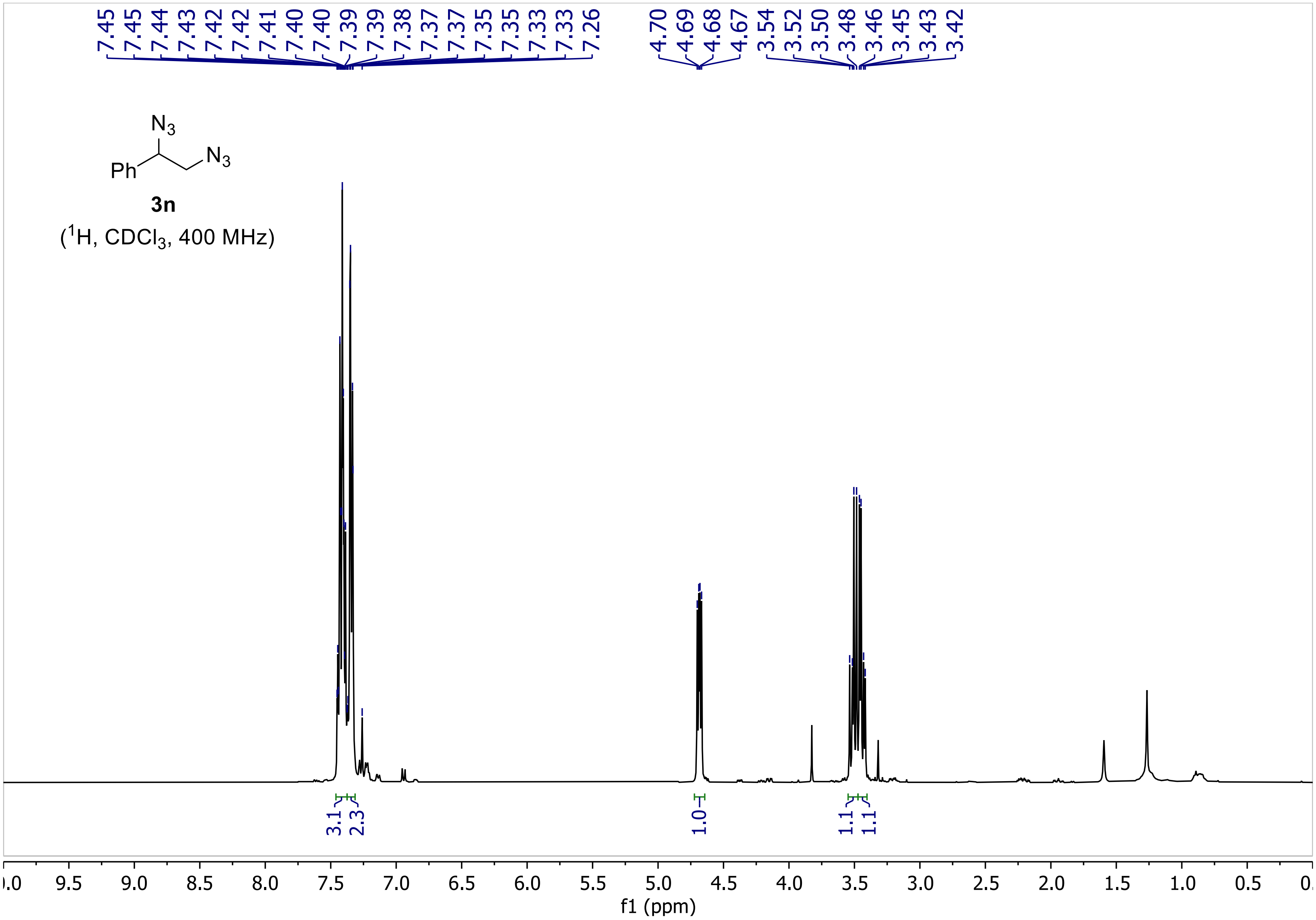
7.45
7.45
7.44
7.43
7.42
7.42
7.41
7.40
7.40
7.39
7.39
7.38
7.37
7.37
7.35
7.35
7.33
7.33
7.26
4.70
4.69
4.68
4.67
3.54
3.52
3.50
3.48
3.46
3.45
3.43
3.42
N3
Ph
N3
3n
(1H, CDCl3, 400 MHz)
3.1
2.3
1.0
1.1
1.1
9.5
9.0
8.5
8.0
7.5
7.0
6.5
6.0
5.5
5.0
4.5
4.0
3.5
3.0
2.5
2.0
1.5
1.0
0.5
f1 (ppm)

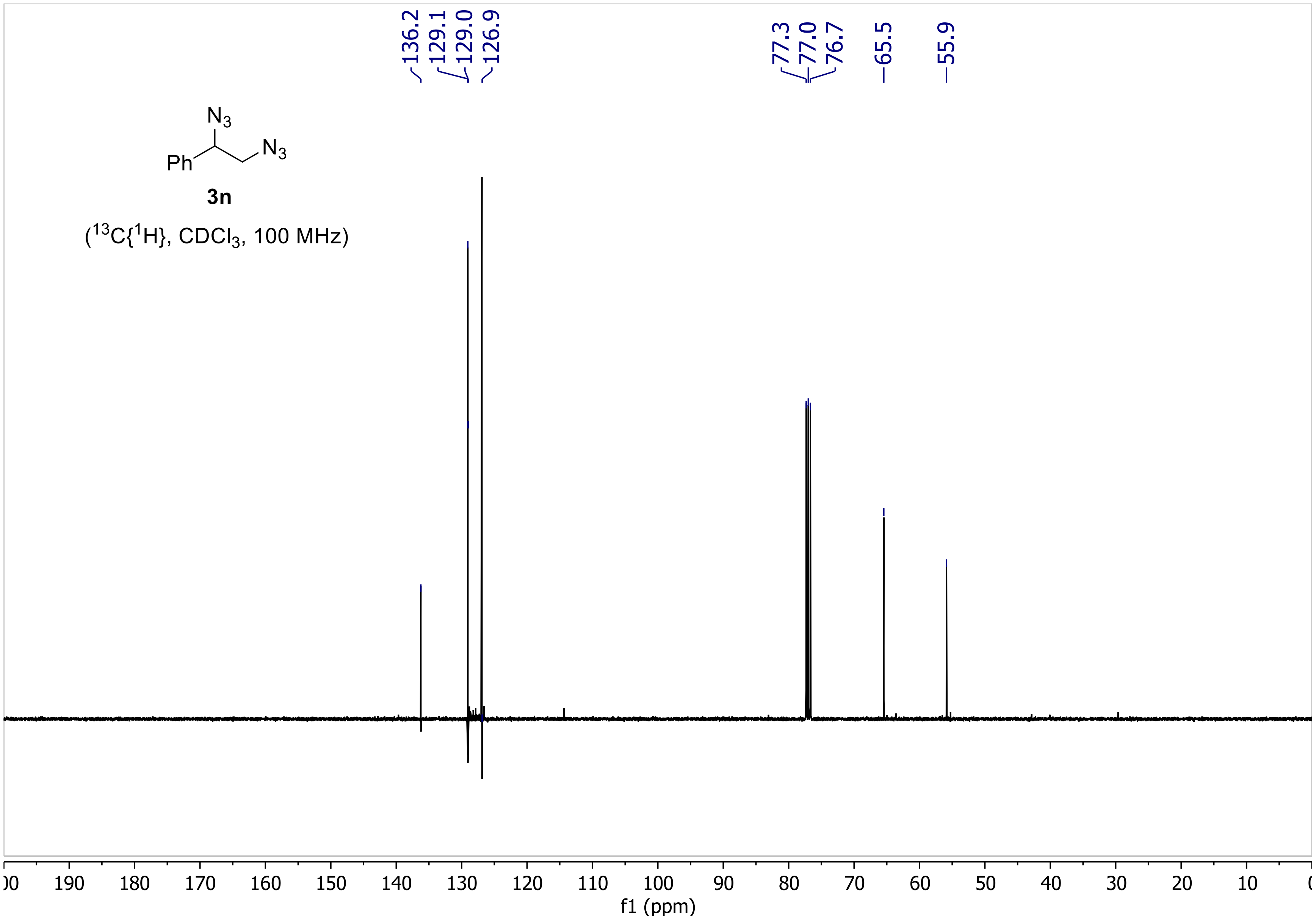
136.2
129.1
129.0
126.9
77.3
77.0
76.7
65.5
55.9
N3
N3
Ph
3n
(13C{1H}, CDCl3, 100 MHz)
190
180
170
160
150
140
130
120
110
100
90
80
70
60
50
40
30
20
10
f1 (ppm)

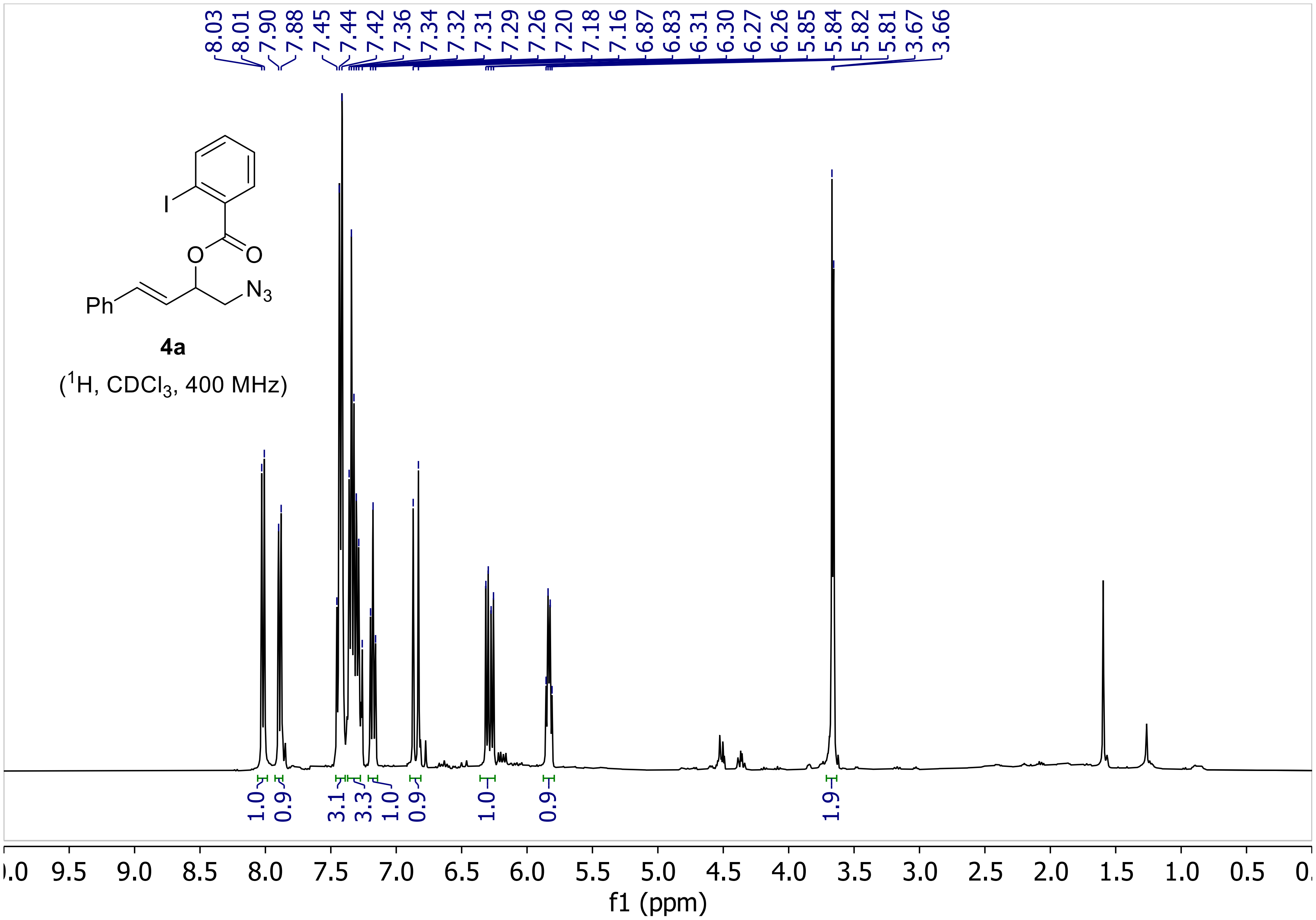

4a
(1H, CDCl3, 400 MHz)
f1 (ppm)

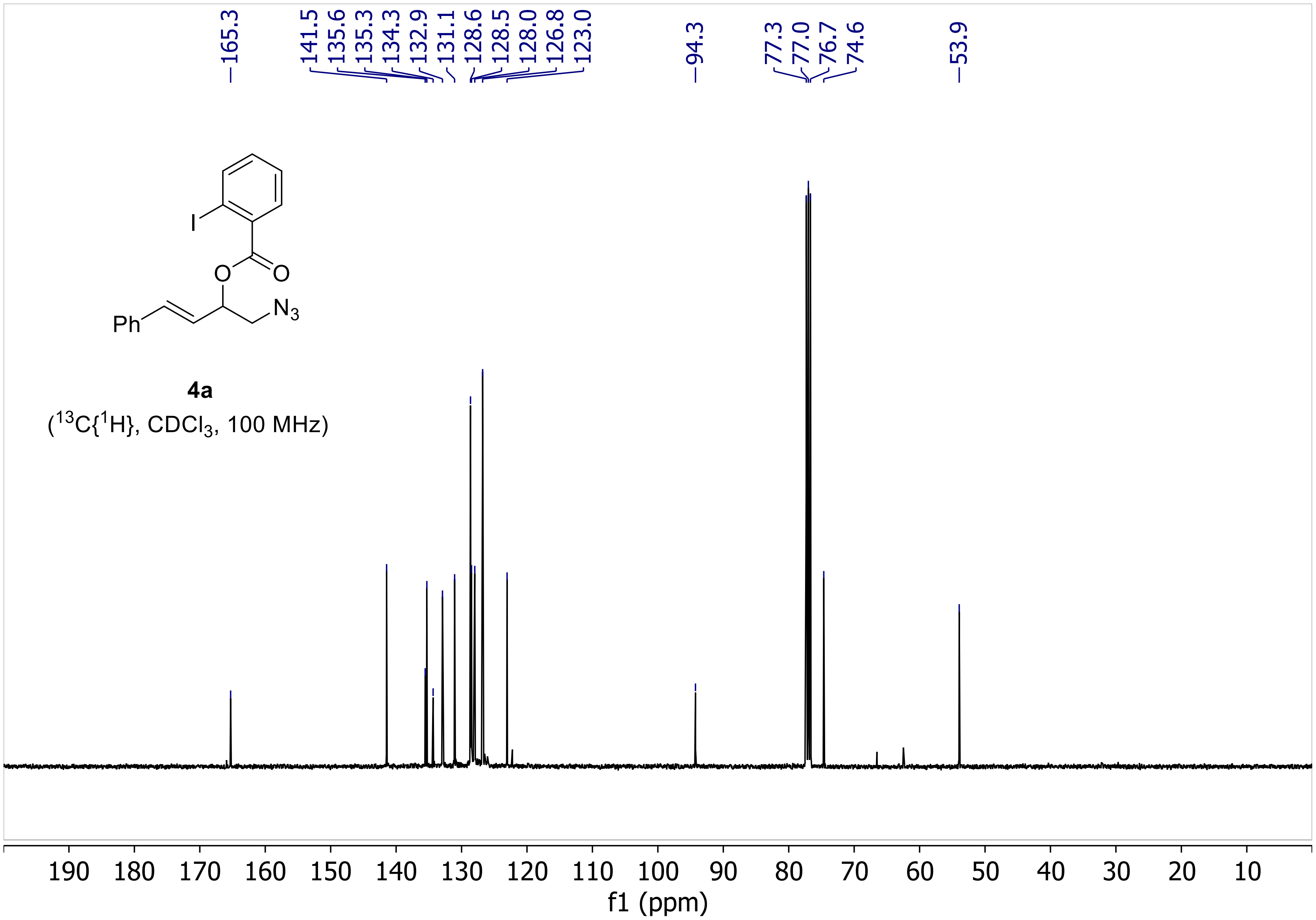
165.3
141.5
135.6
135.3
134.3
132.9
131.1
128.6
128.5
128.0
126.8
123.0
94.3
77.3
77.0
76.7
74.6
53.9
I
O
O
Ph
N3
4a
(13C{1H}, CDCl3, 100 MHz)
190 180 170 160 150 140 130 120 110 100 90 80 70 60 50 40 30 20 10
f1 (ppm)

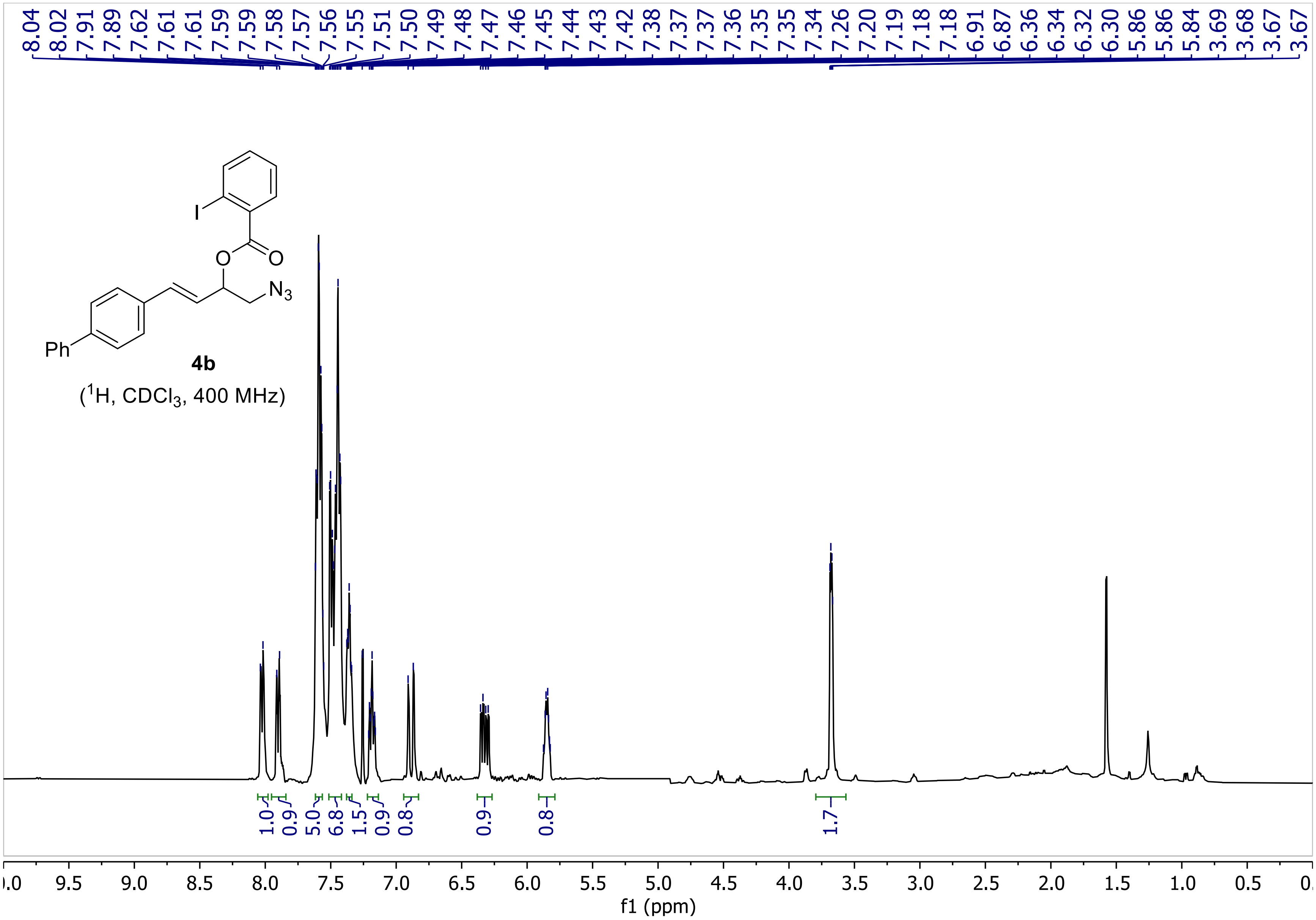

8.04
8.02
7.91
7.89
7.62
7.61
7.61
7.59
7.59
7.58
7.57
7.56
7.55
7.51
7.50
7.49
7.48
7.47
7.46
7.45
7.44
7.43
7.42
7.38
7.37
7.37
7.36
7.35
7.35
7.34
7.26
7.20
7.19
7.18
7.18
6.91
6.87
6.36
6.34
6.32
6.30
5.86
5.86
5.84
3.69
3.68
3.67
3.67
I
O
O
N3
Ph
4b
(1H, CDCl3, 400 MHz)
1.0
0.9
5.0
6.8
1.5
0.9
0.8
0.9
0.8
1.7
f1 (ppm)

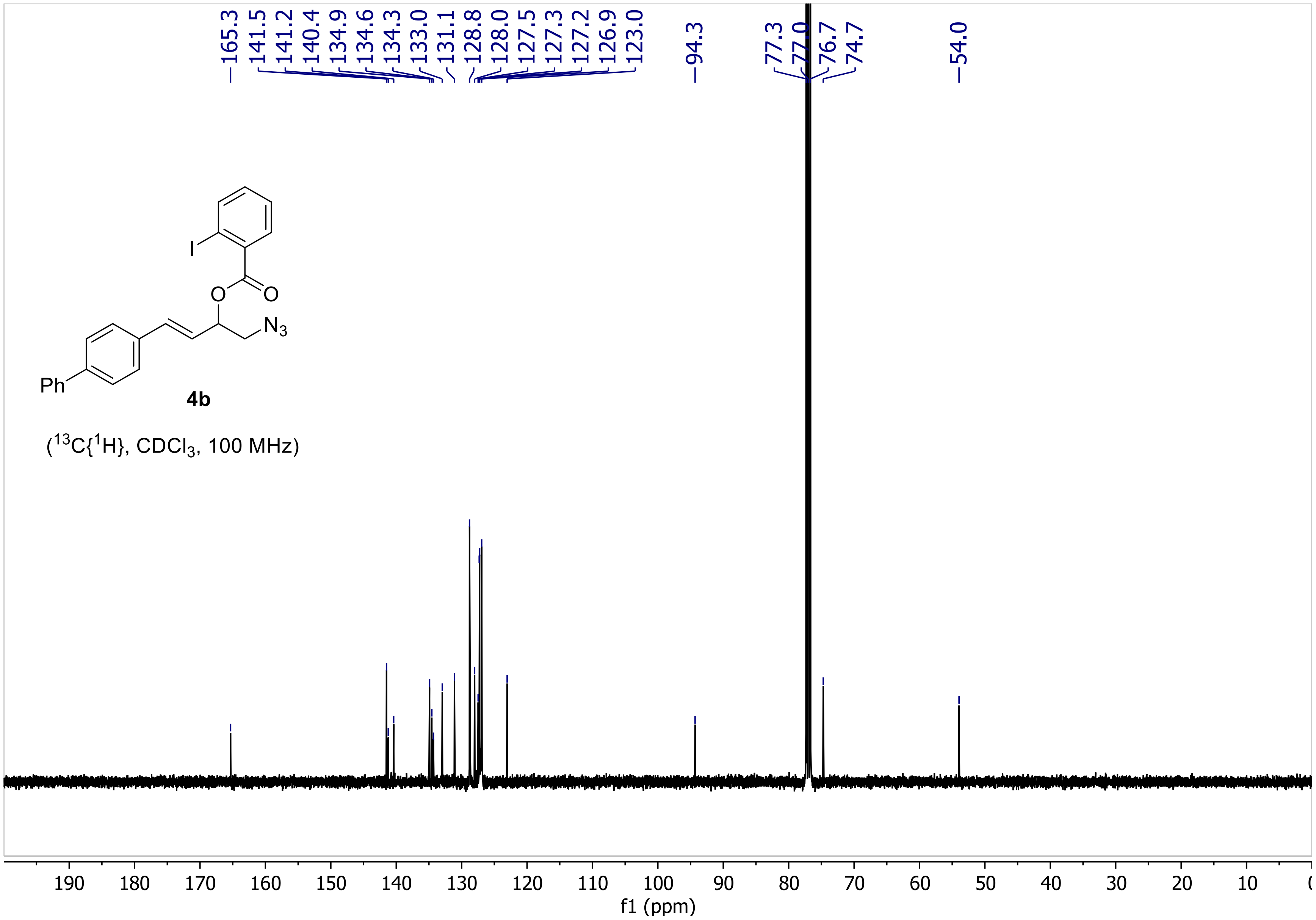

165.3
141.5
141.2
140.4
134.9
134.6
134.3
133.0
131.1
128.8
128.0
127.5
127.3
127.2
126.9
123.0
94.3
77.3
77.0
76.7
74.7
54.0
I
O
O
N3
Ph
4b
(^{13}C{^{1}H}, CDCl$_3$, 100 MHz)
190 180 170 160 150 140 130 120 110 100 90 80 70 60 50 40 30 20 10
f1 (ppm)

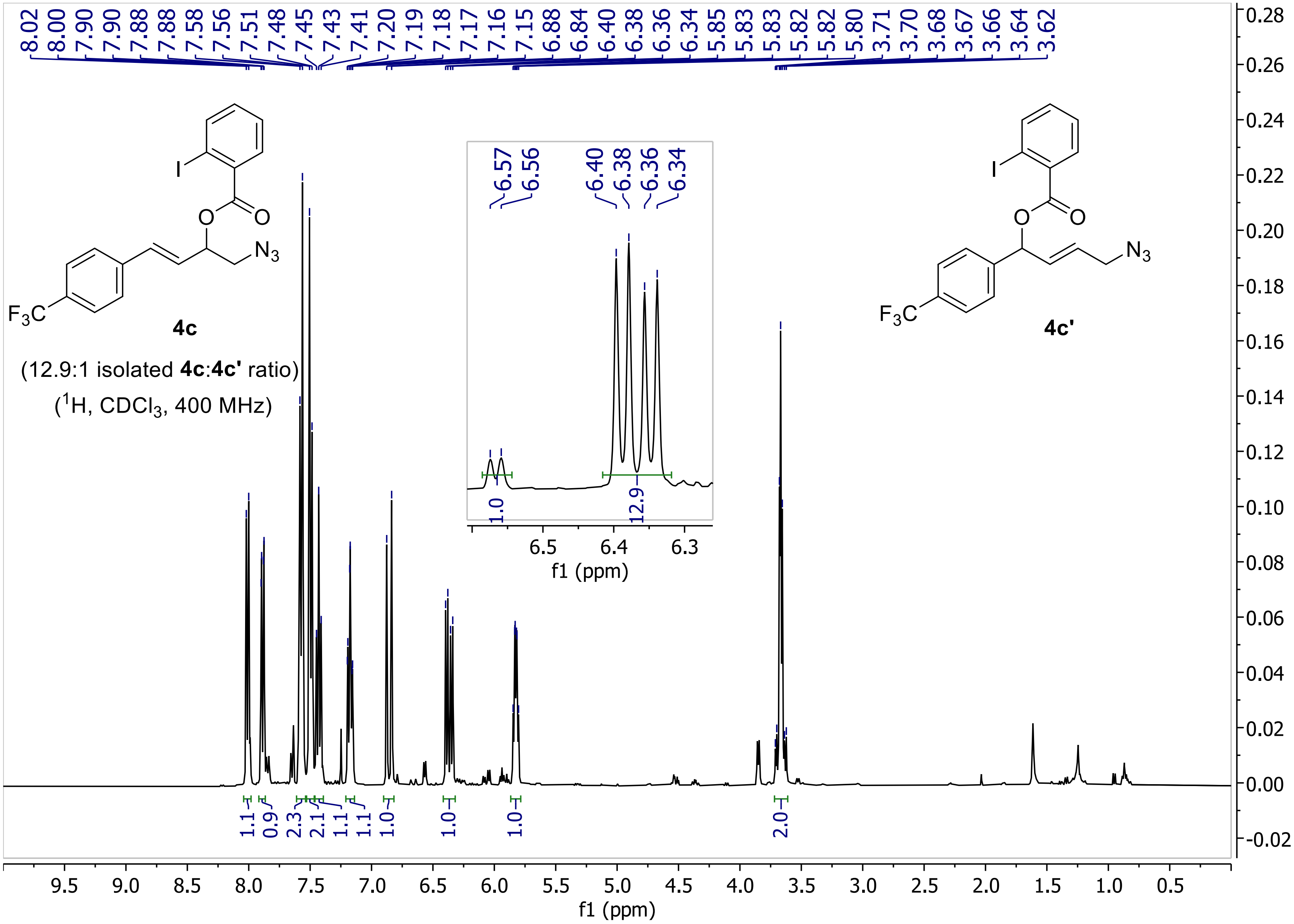
8.02 8.00 7.90 7.90 7.88 7.88 7.58 7.56 7.51 7.48 7.45 7.43 7.41 7.20 7.19 7.18 7.17 7.16 7.15 6.88 6.84 6.40 6.38 6.36 6.34 5.85 5.83 5.83 5.82 5.82 5.80 3.71 3.70 3.68 3.67 3.66 3.64 3.62
I
O
O
N3
F3C
4c
(12.9:1 isolated 4c:4c' ratio)
(1H, CDCl3, 400 MHz)
6.57 6.56 6.40 6.38 6.36 6.34
1.0
12.9
6.5 6.4 6.3
f1 (ppm)
4c'
1.1 0.9 2.3 2.1 1.1 1.1 1.0 1.0 1.0 2.0
9.5 9.0 8.5 8.0 7.5 7.0 6.5 6.0 5.5 5.0 4.5 4.0 3.5 3.0 2.5 2.0 1.5 1.0 0.5
f1 (ppm)
0.28 0.26 0.24 0.22 0.20 0.18 0.16 0.14 0.12 0.10 0.08 0.06 0.04 0.02 0.00 -0.02

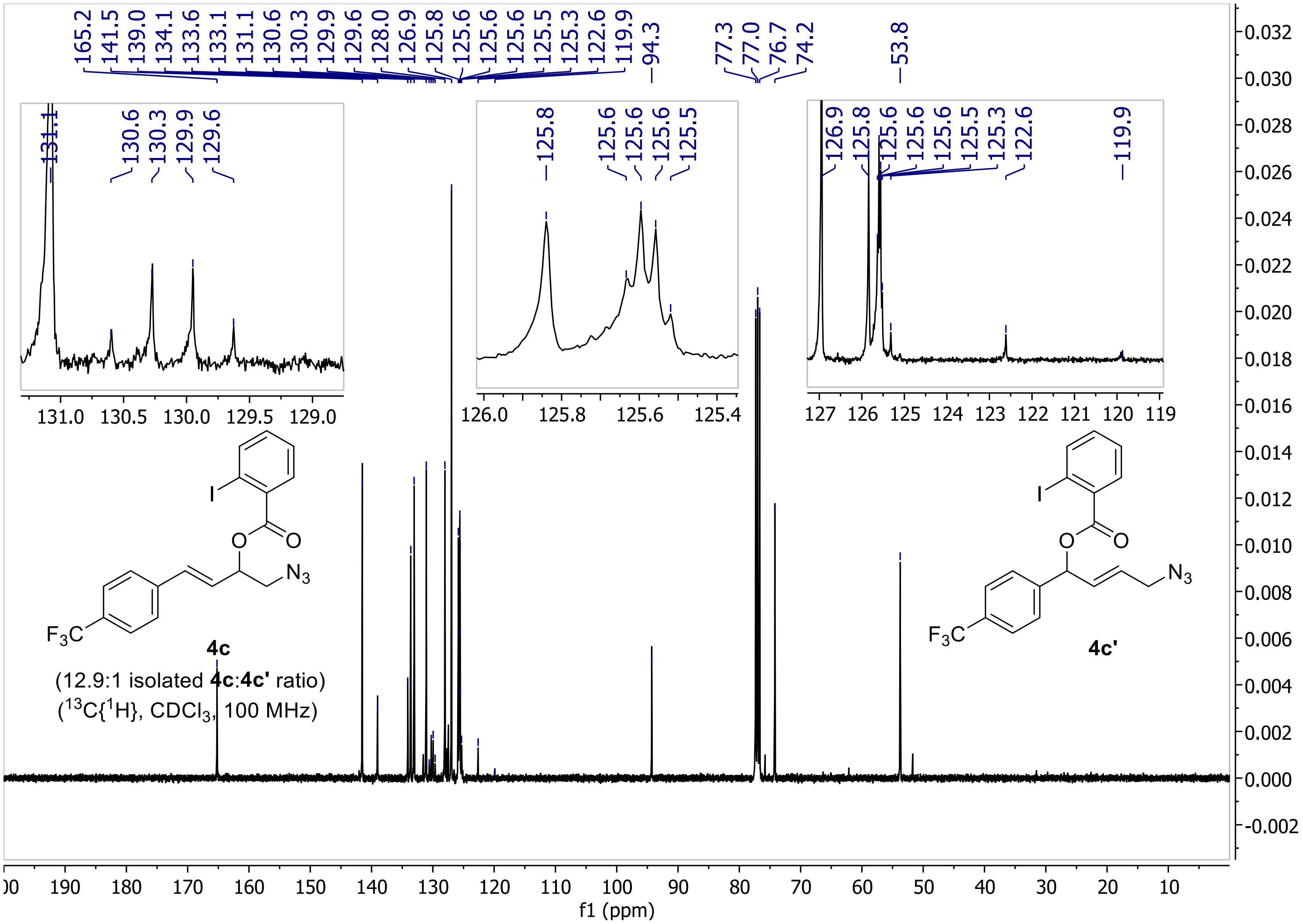
165.2 141.5 139.0 134.1 133.6 133.1 131.1 130.6 130.3 129.9 129.6 128.0 126.9 125.8 125.6 125.6 125.6 125.5 125.3 122.6 119.9
94.3
77.3 77.0 76.7 74.2
53.8
131.1 130.6 130.3 129.9 129.6
125.8 125.6 125.6 125.6 125.5
126.9 125.8 125.6 125.6 125.6 125.5 125.3 122.6 119.9
I
O
O
N_3
F_3C
4c
4c'
(12.9:1 isolated 4c:4c' ratio)
($^{13}C\{^1H\}$, $CDCl_3$, 100 MHz)
f1 (ppm)

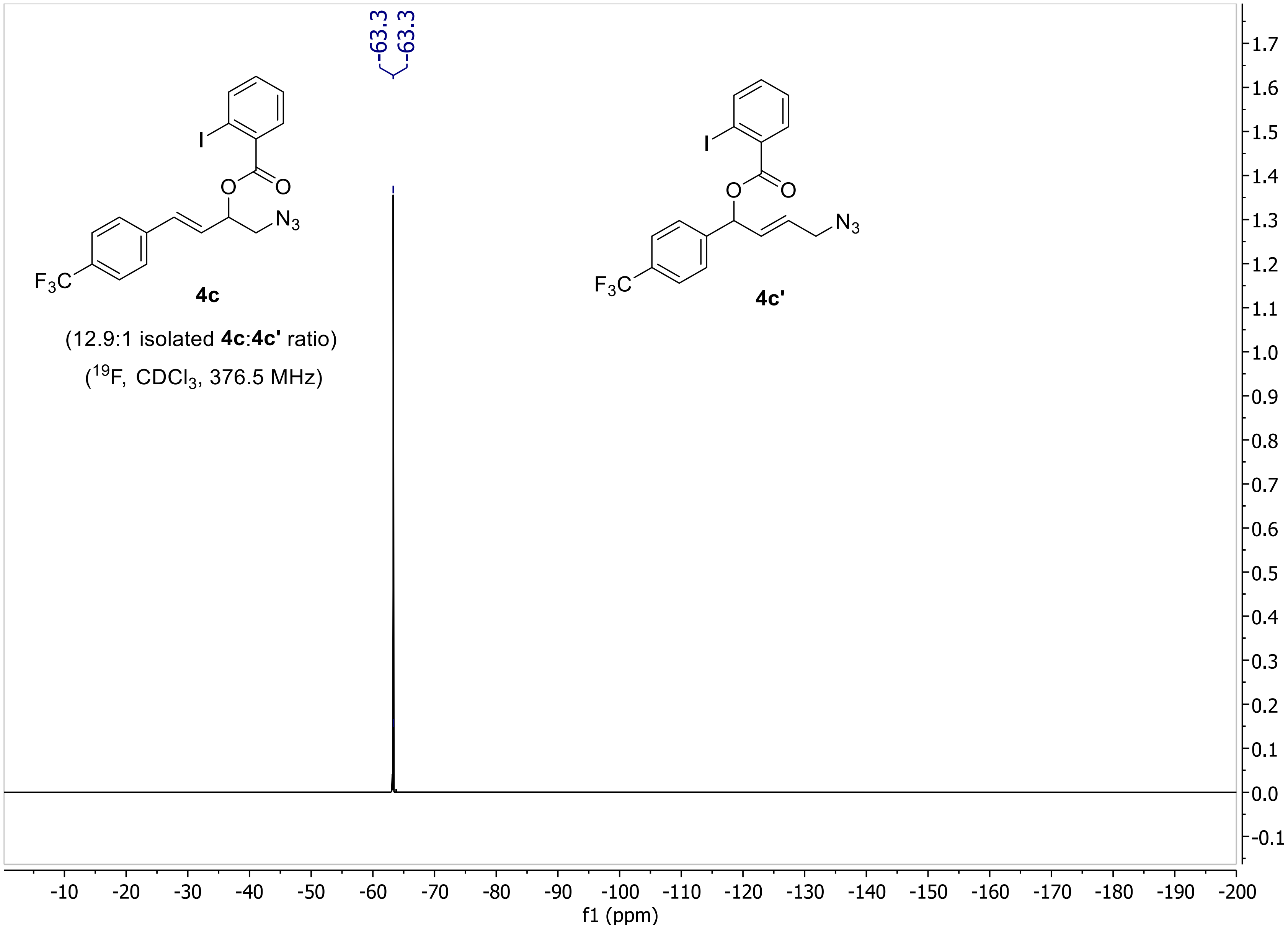

-63.3
-63.3
4c
4c'
(12.9:1 isolated 4c:4c' ratio)
(19F, CDCl3, 376.5 MHz)
f1 (ppm)

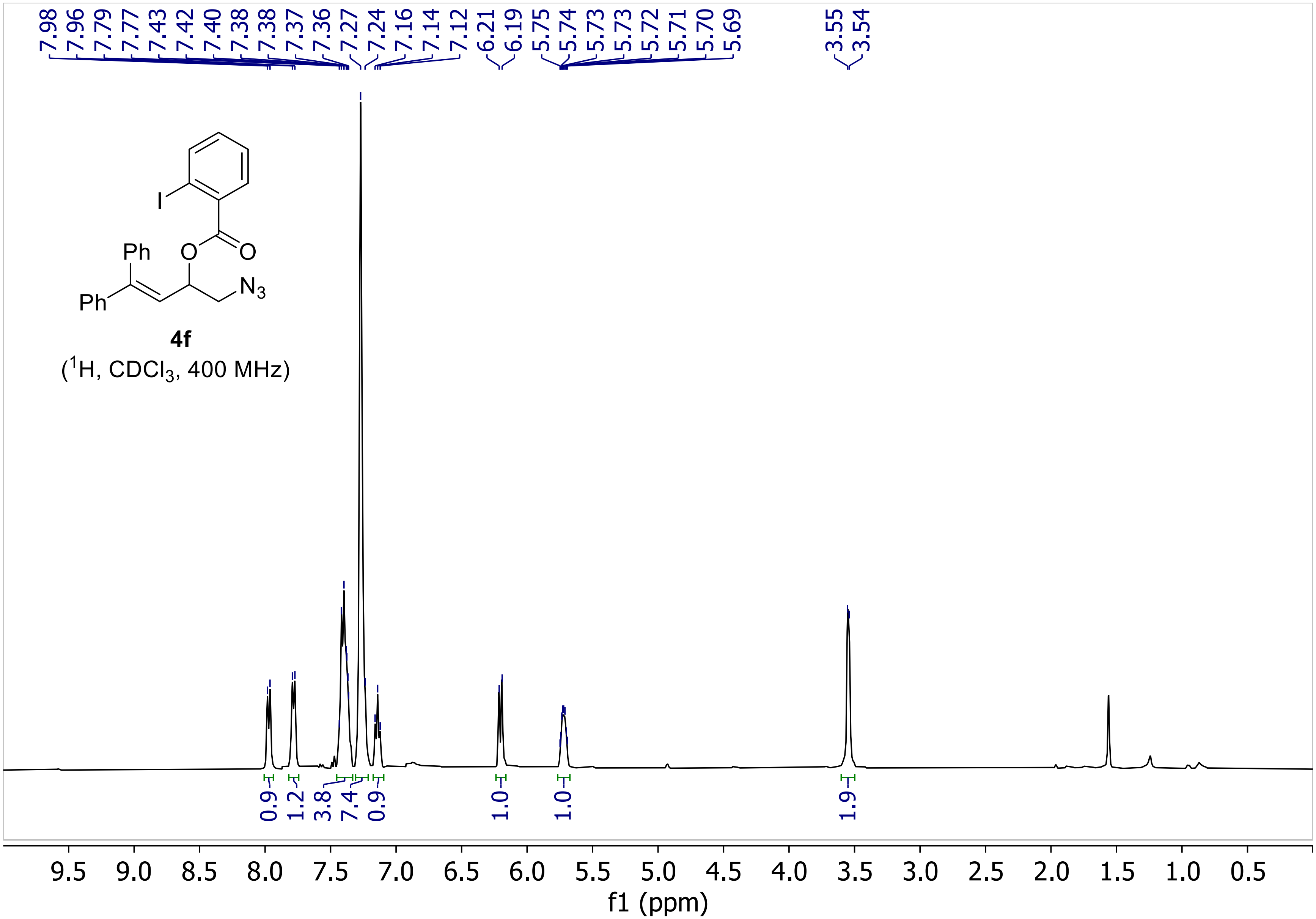

7.98 7.96 7.79 7.77 7.43 7.42 7.40 7.38 7.38 7.37 7.36 7.27 7.24 7.16 7.14 7.12 6.21 6.19 5.75 5.74 5.73 5.73 5.72 5.71 5.70 5.69 3.55 3.54
I
Ph
O
O
Ph
N3
4f
(1H, CDCl3, 400 MHz)
0.9 1.2 3.8 7.4 0.9 1.0 1.0 1.9
9.5 9.0 8.5 8.0 7.5 7.0 6.5 6.0 5.5 5.0 4.5 4.0 3.5 3.0 2.5 2.0 1.5 1.0 0.5
f1 (ppm)

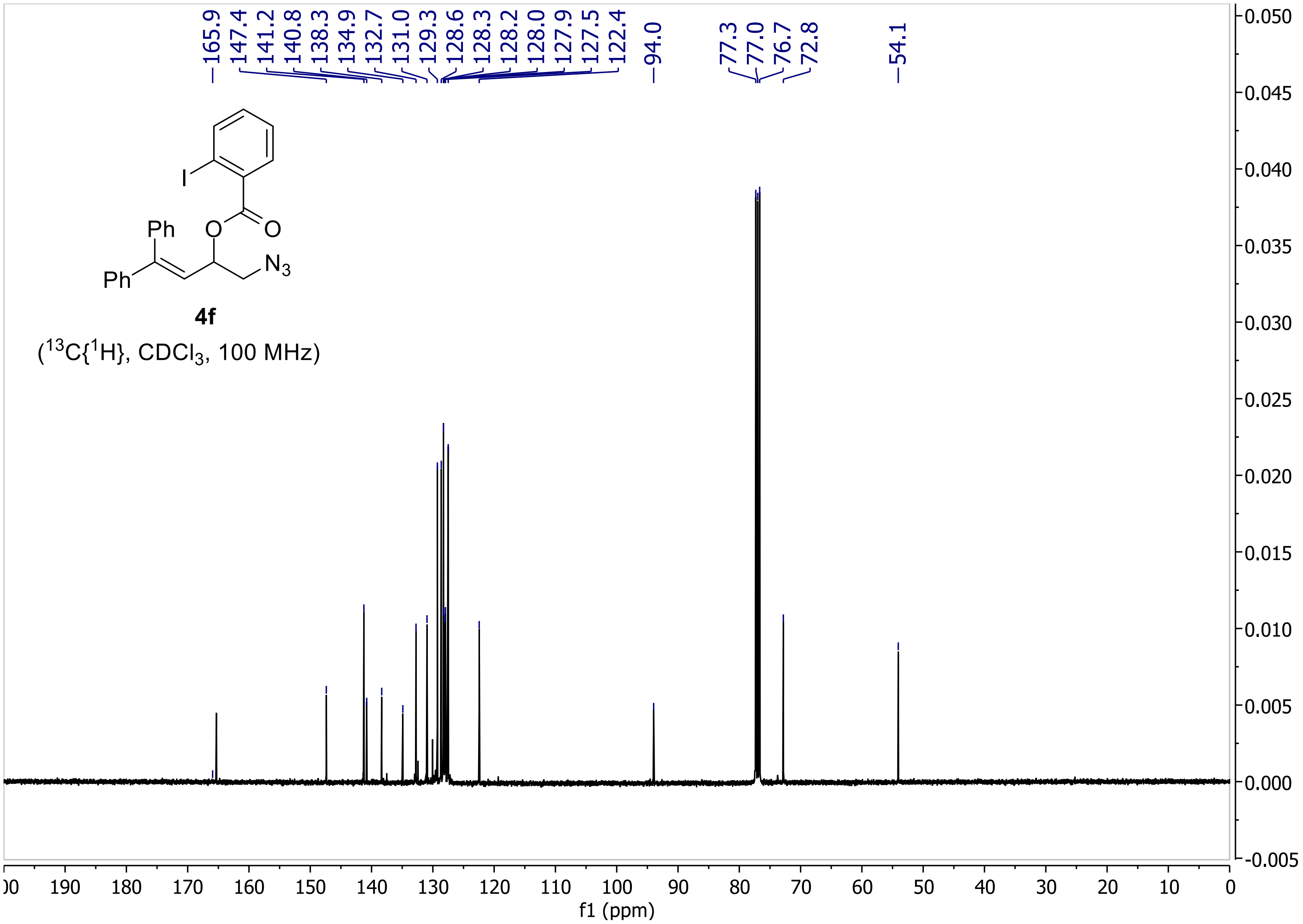
165.9
147.4
141.2
140.8
138.3
134.9
132.7
131.0
129.3
128.6
128.3
128.2
128.0
127.9
127.5
122.4
94.0
77.3
77.0
76.7
72.8
54.1
I
O
O
Ph
Ph
N3
4f
(13C{1H}, CDCl3, 100 MHz)
f1 (ppm)

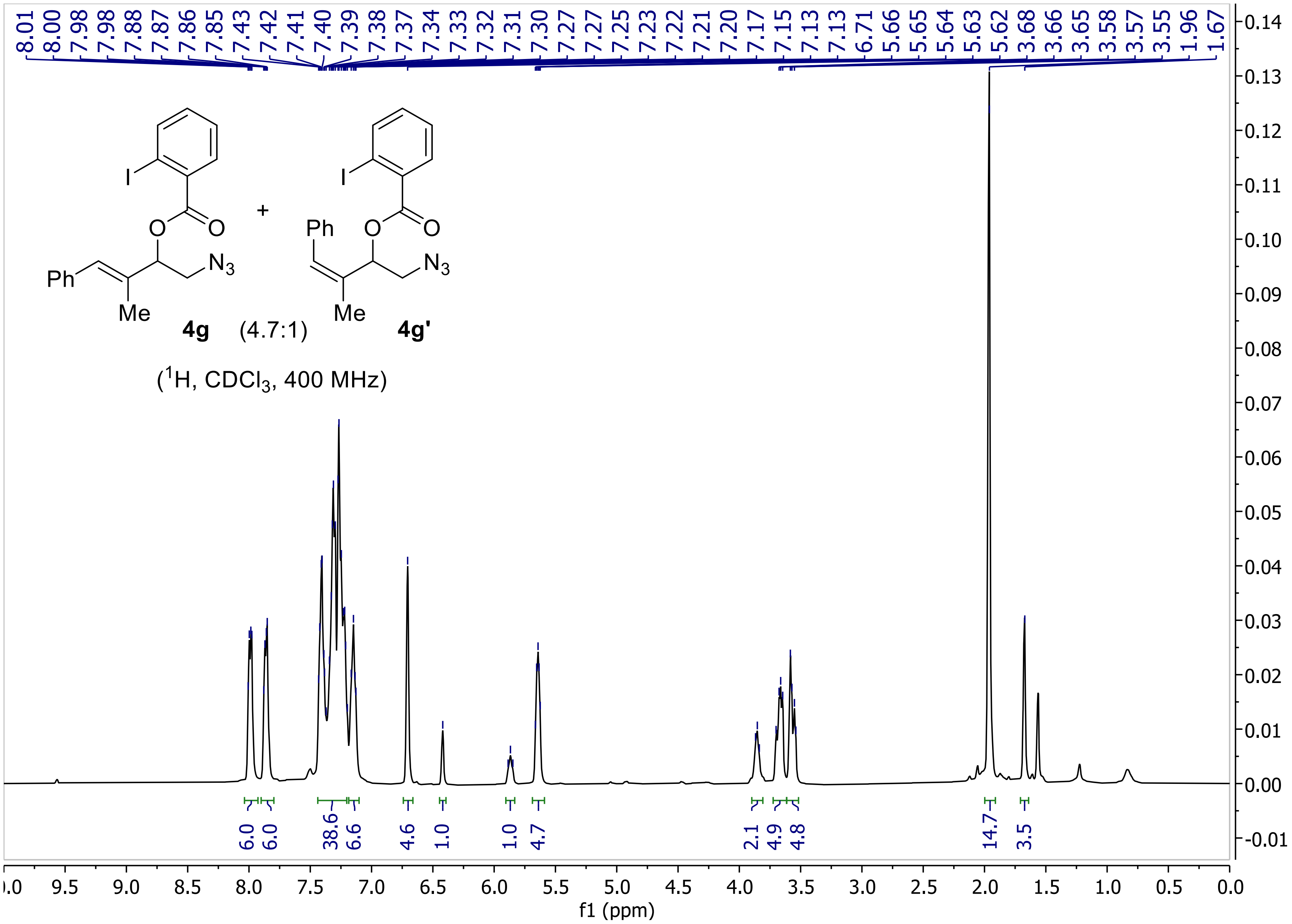

8.01 8.00 7.98 7.98 7.88 7.87 7.86 7.85 7.43 7.42 7.41 7.40 7.39 7.38 7.37 7.34 7.33 7.32 7.31 7.30 7.27 7.27 7.25 7.23 7.22 7.21 7.20 7.17 7.15 7.13 7.13 6.71 5.66 5.65 5.64 5.63 5.62 3.68 3.66 3.65 3.58 3.57 3.55 1.96 1.67
I
O
O
N3
Ph
Me
+
Ph
4g
(4.7:1)
4g'
(1H, CDCl3, 400 MHz)
6.0 6.0 38.6 6.6 4.6 1.0 1.0 4.7 2.1 4.9 4.8 14.7 3.5
f1 (ppm)

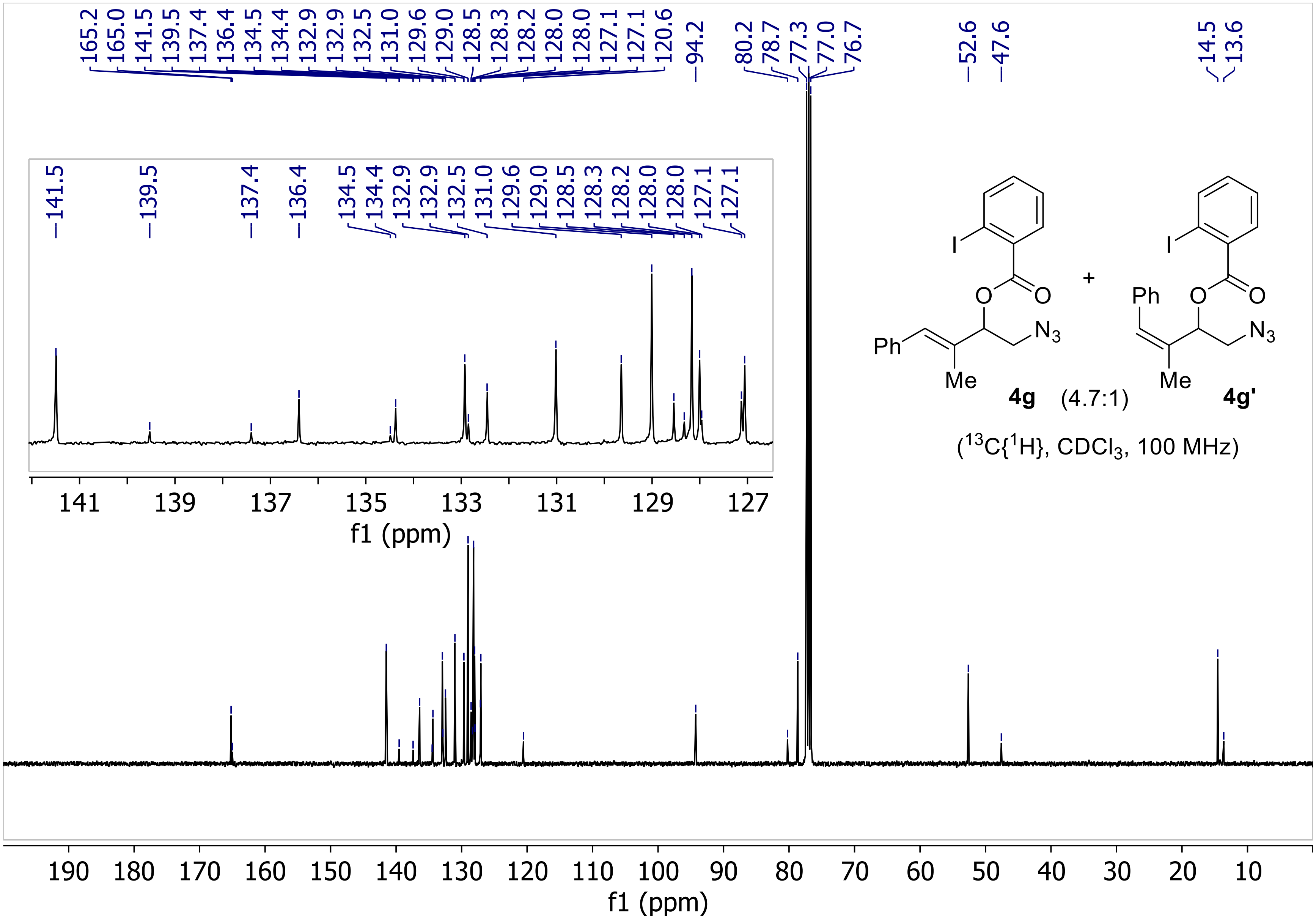
165.2
165.0
141.5
139.5
137.4
136.4
134.5
134.4
132.9
132.9
132.5
131.0
129.6
129.0
128.5
128.3
128.2
128.0
128.0
127.1
127.1
120.6
94.2
80.2
78.7
77.3
77.0
76.7
52.6
47.6
14.5
13.6
f1 (ppm)
I
O
O
Ph
N3
Me
4g
+
Ph
4g'
(4.7:1)
(13C{1H}, CDCl3, 100 MHz)

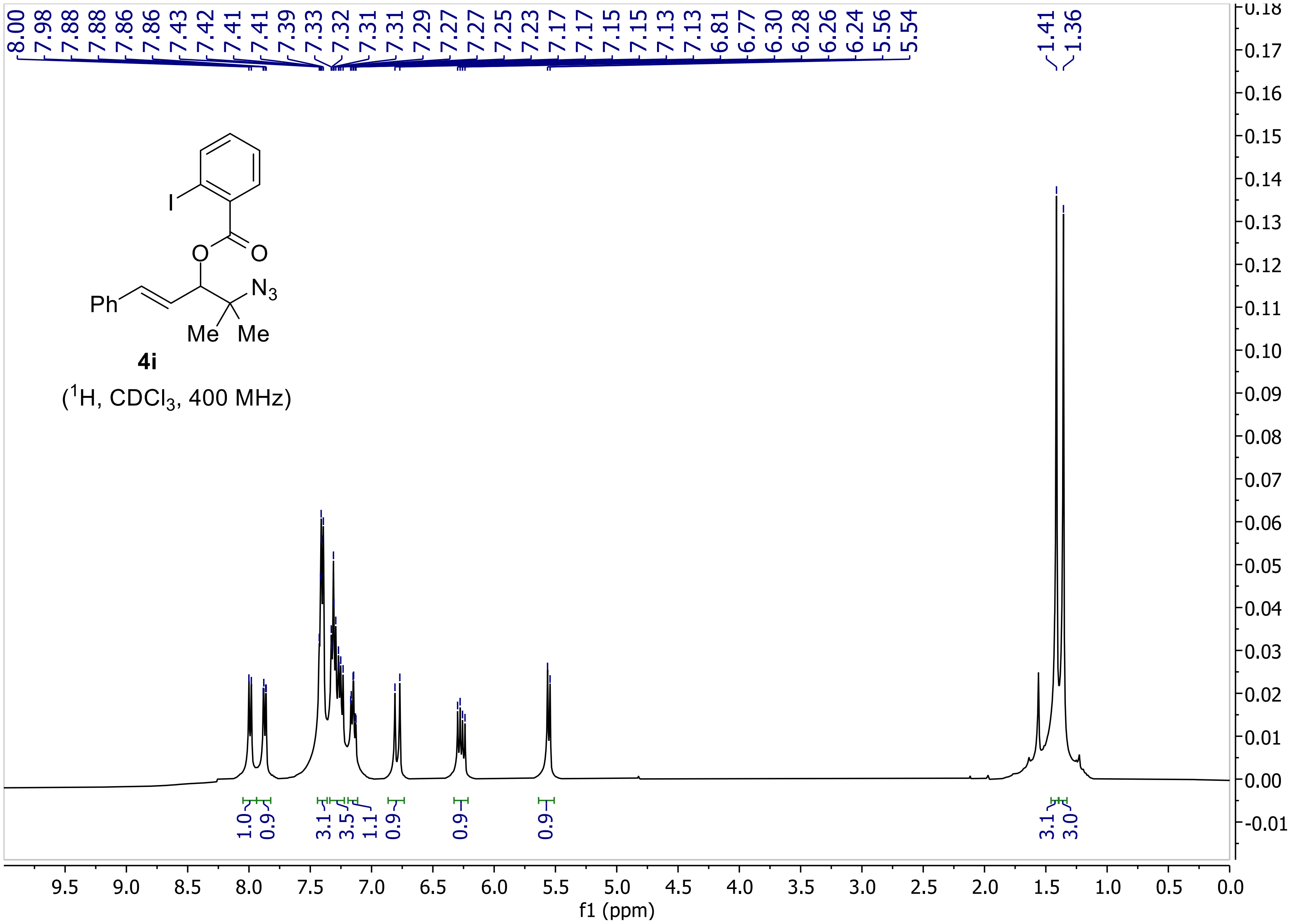

8.00
7.98
7.88
7.88
7.86
7.86
7.43
7.42
7.41
7.41
7.39
7.33
7.32
7.31
7.31
7.29
7.27
7.27
7.25
7.23
7.17
7.17
7.15
7.15
7.13
7.13
6.81
6.77
6.30
6.28
6.26
6.24
5.56
5.54
1.41
1.36
I
O
O
N3
Ph
Me
Me
4i
(1H, CDCl3, 400 MHz)
1.0
0.9
3.1
3.5
1.1
0.9
0.9
0.9
3.1
3.0
f1 (ppm)

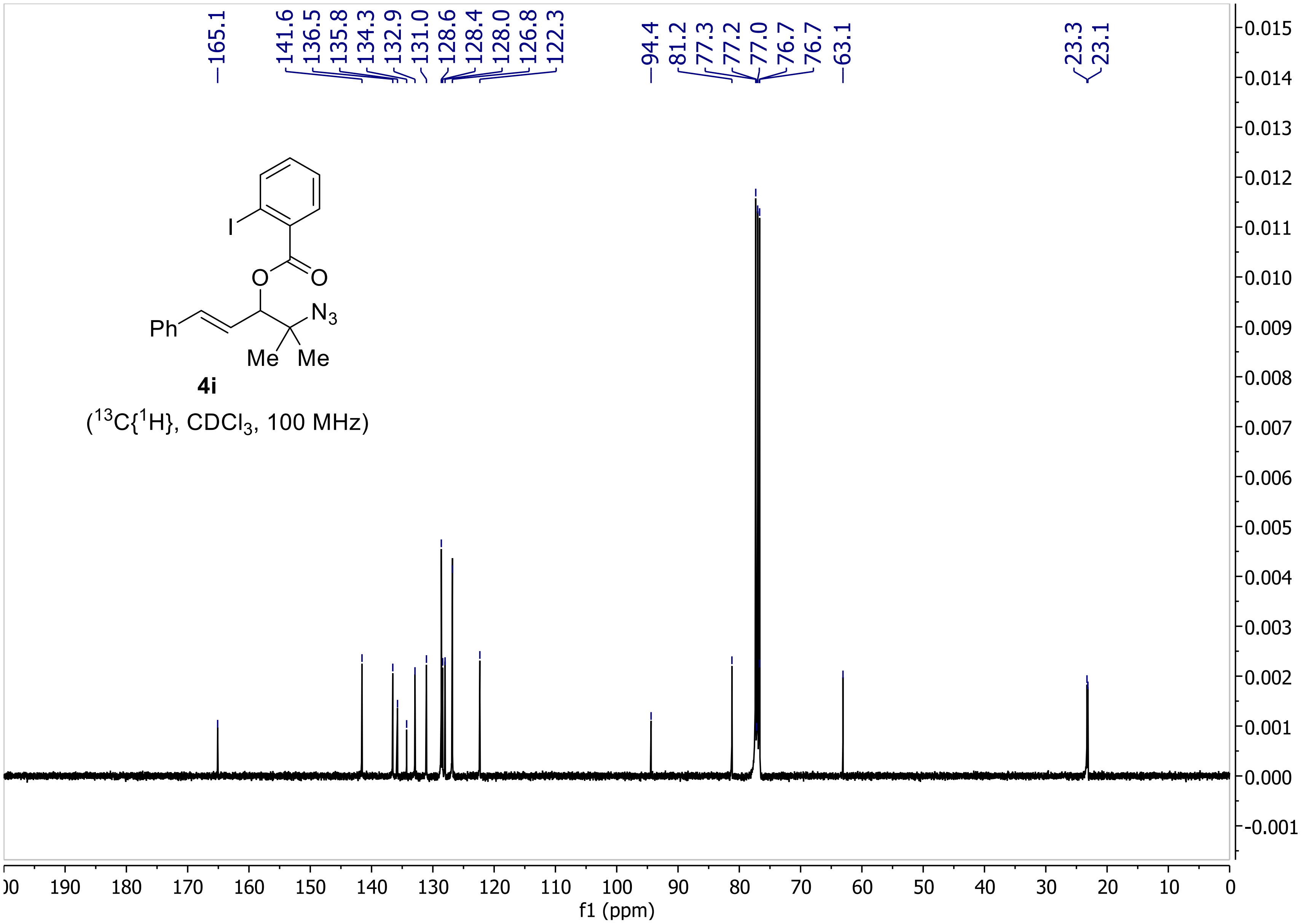

4i

($^{13}C\{^1H\}$, $CDCl_3$, 100 MHz)

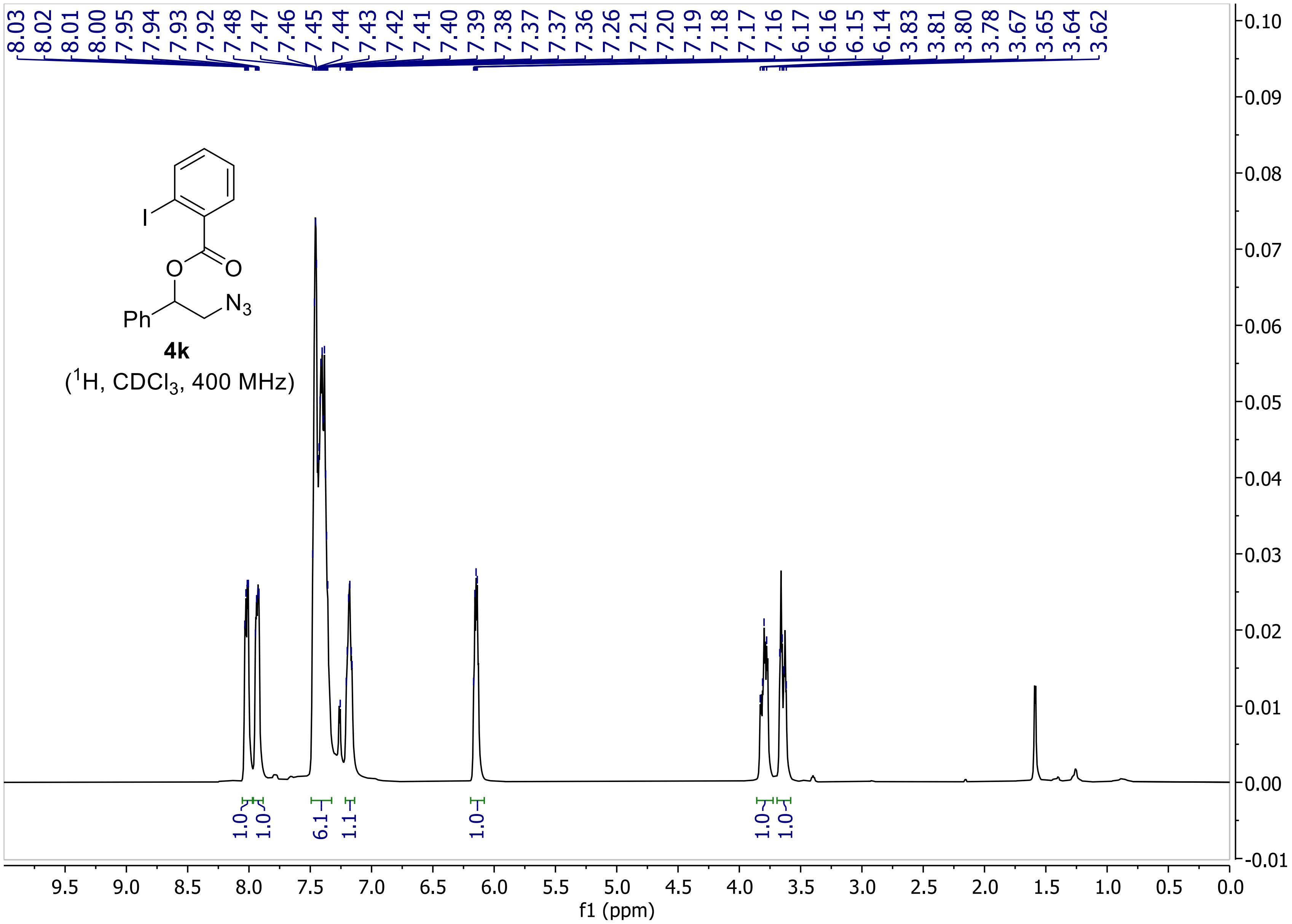
8.03
8.02
8.01
8.00
7.95
7.94
7.93
7.92
7.48
7.47
7.46
7.45
7.44
7.43
7.42
7.41
7.40
7.39
7.38
7.37
7.37
7.36
7.26
7.21
7.20
7.19
7.18
7.17
7.16
6.17
6.16
6.15
6.14
3.83
3.81
3.80
3.78
3.67
3.65
3.64
3.62
I
O
O
N3
Ph
4k
(1H, CDCl3, 400 MHz)
1.0
1.0
6.1
1.1
1.0
1.0
1.0
f1 (ppm)

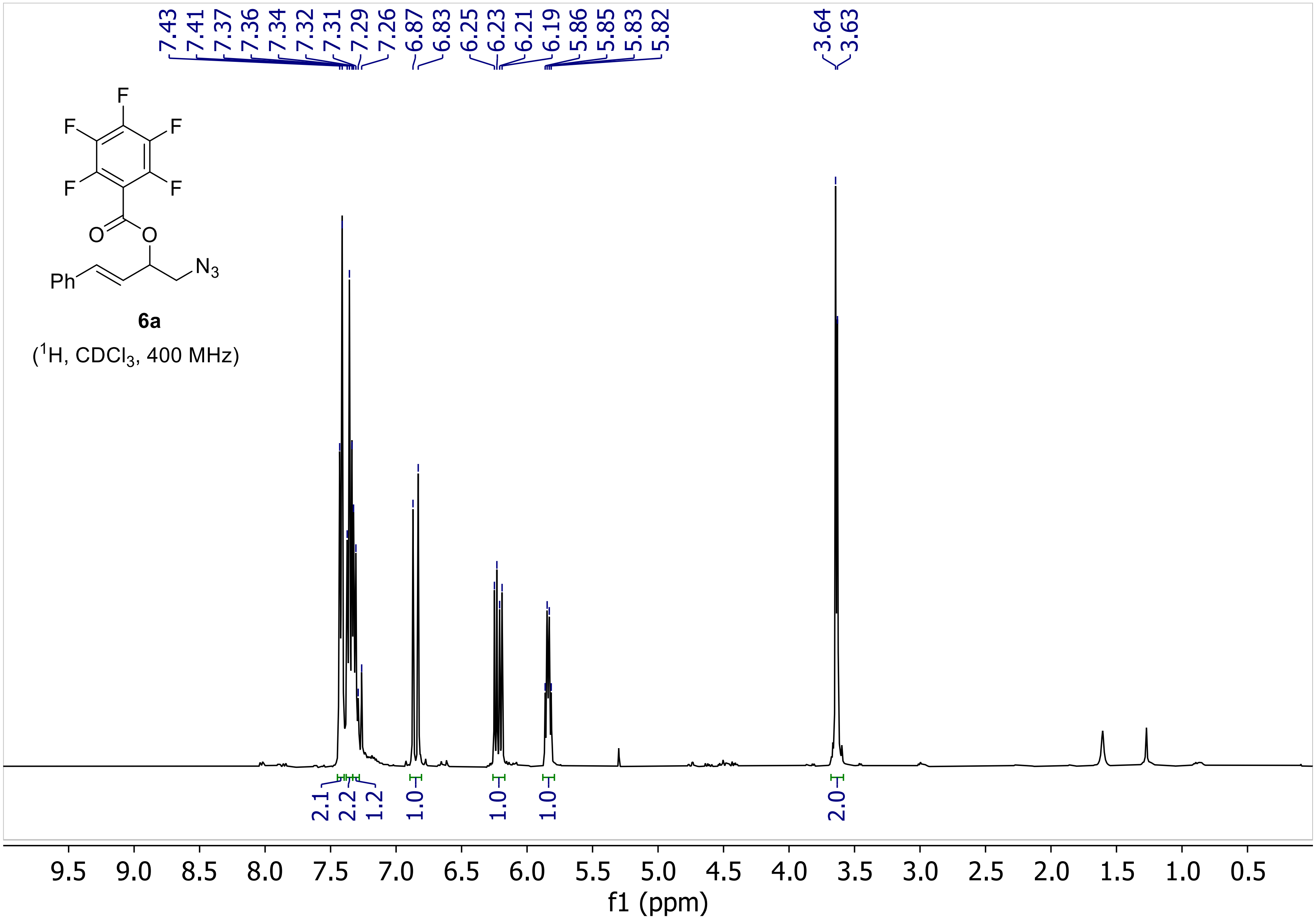
7.43
7.41
7.37
7.36
7.34
7.32
7.31
7.29
7.26
6.87
6.83
6.25
6.23
6.21
6.19
5.86
5.85
5.83
5.82
3.64
3.63
F
F
F
F
F
O
O
Ph
N3
6a
(1H, CDCl3, 400 MHz)
2.1
2.2
1.2
1.0
1.0
1.0
2.0
9.5 9.0 8.5 8.0 7.5 7.0 6.5 6.0 5.5 5.0 4.5 4.0 3.5 3.0 2.5 2.0 1.5 1.0 0.5
f1 (ppm)

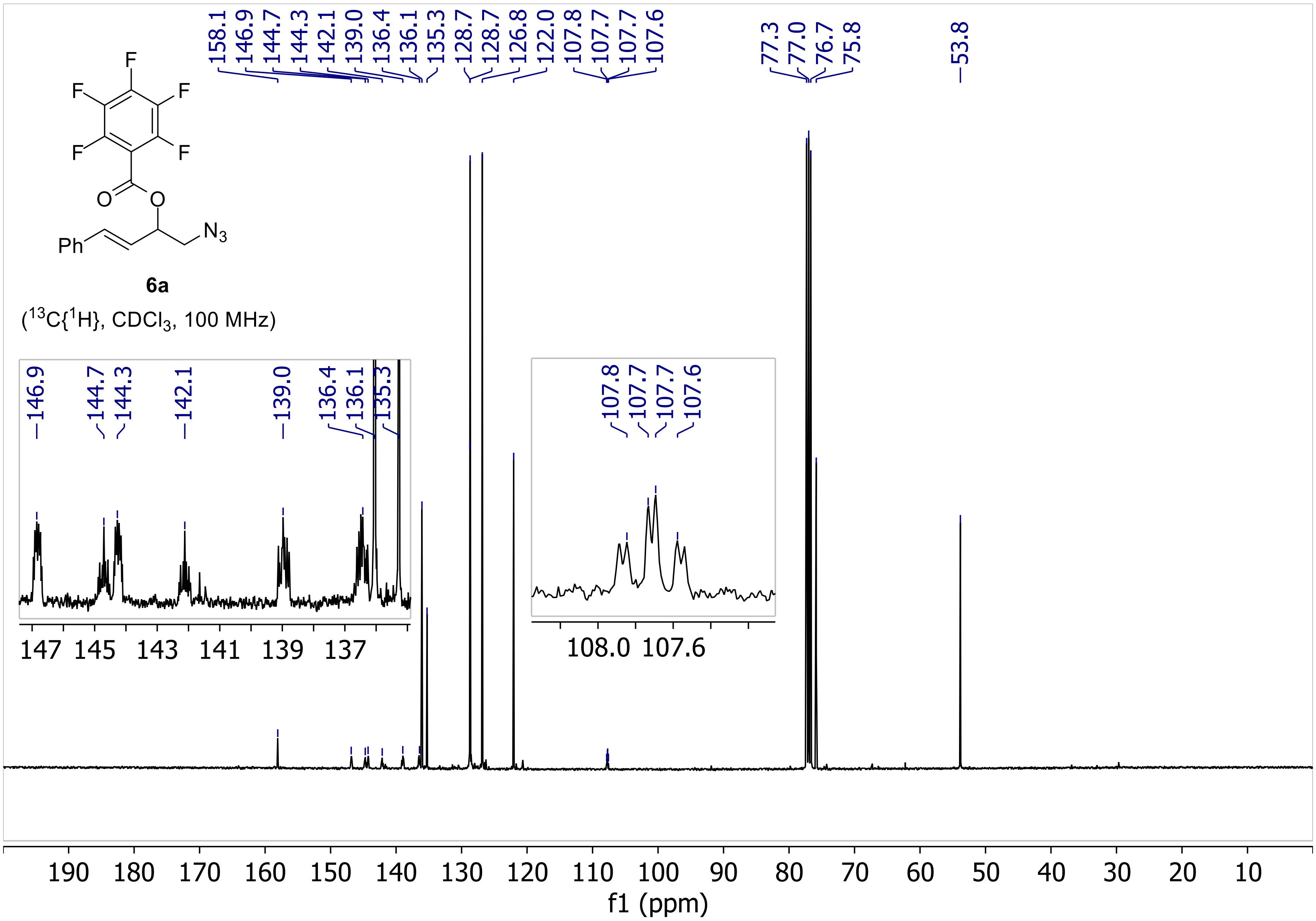
F
F
F
F
F
O
O
N3
Ph
6a
(13C{1H}, CDCl3, 100 MHz)
158.1
146.9
144.7
144.3
142.1
139.0
136.4
136.1
135.3
128.7
128.7
126.8
122.0
107.8
107.7
107.7
107.6
77.3
77.0
76.7
75.8
53.8
147 145 143 141 139 137
108.0 107.6
190 180 170 160 150 140 130 120 110 100 90 80 70 60 50 40 30 20 10
f1 (ppm)

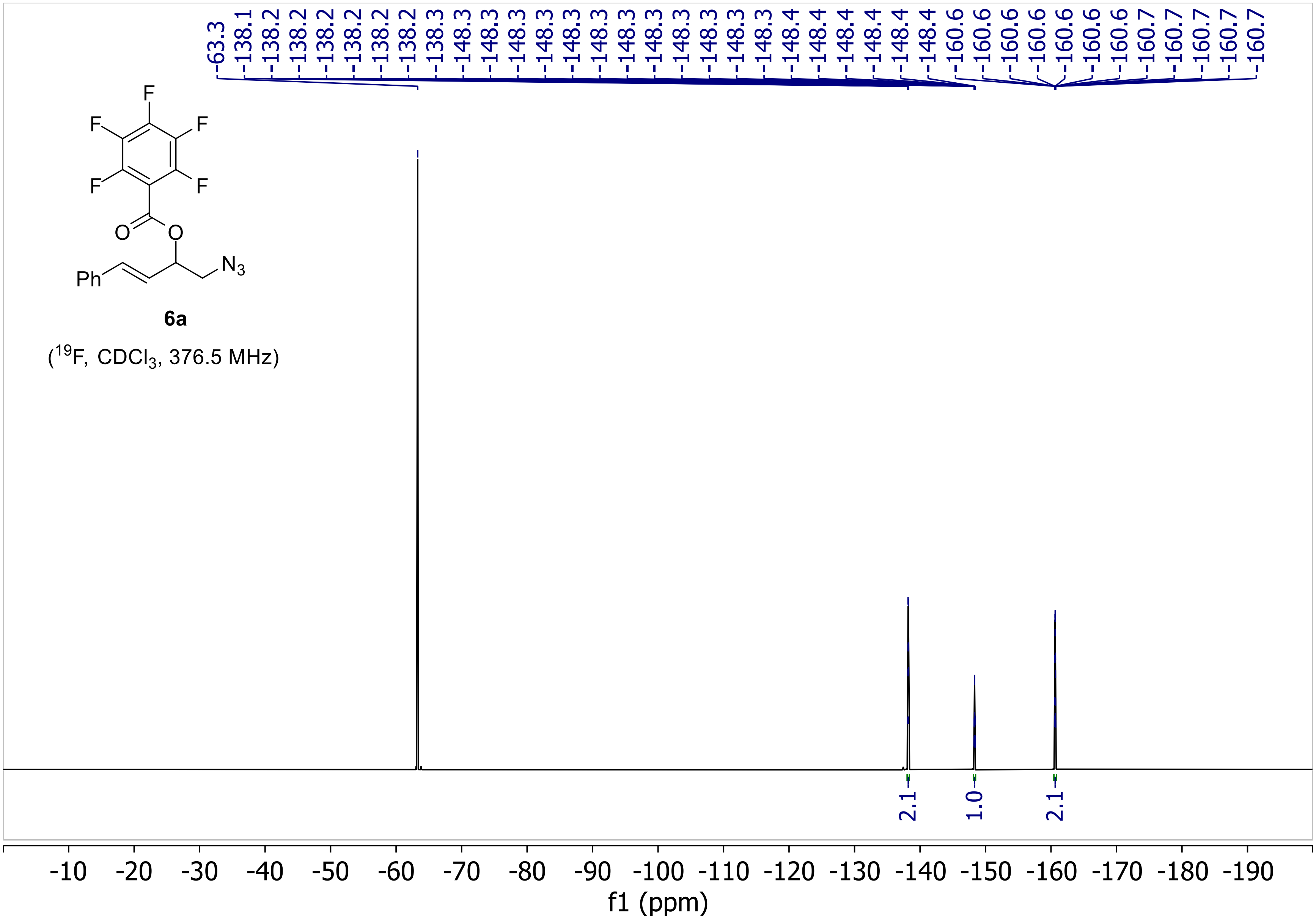
-63.3
-138.1
-138.2
-138.2
-138.2
-138.2
-138.2
-138.2
-138.3
-148.3
-148.3
-148.3
-148.3
-148.3
-148.3
-148.3
-148.3
-148.3
-148.3
-148.3
-148.3
-148.4
-148.4
-148.4
-148.4
-148.4
-148.4
-160.6
-160.6
-160.6
-160.6
-160.6
-160.6
-160.7
-160.7
-160.7
-160.7
-160.7
F
F
F
F
F
O
O
N3
Ph
6a
(19F, CDCl3, 376.5 MHz)
2.1
1.0
2.1
-10
-20
-30
-40
-50
-60
-70
-80
-90
-100
-110
-120
-130
-140
-150
-160
-170
-180
-190
f1 (ppm)

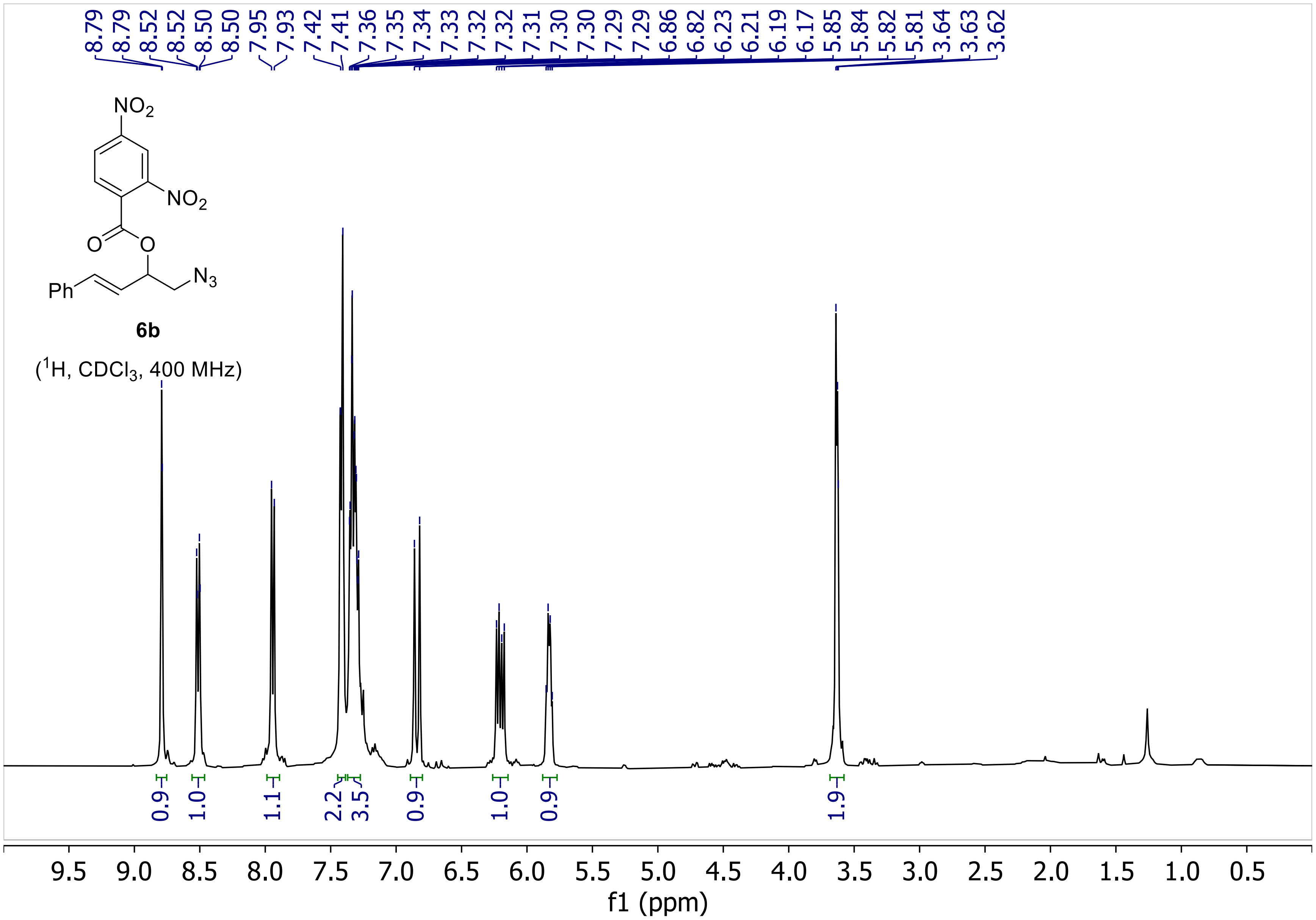

8.79 8.79 8.52 8.52 8.50 8.50 7.95 7.93 7.42 7.41 7.36 7.35 7.34 7.33 7.32 7.32 7.31 7.30 7.30 7.29 7.29 6.86 6.82 6.23 6.21 6.19 6.17 5.85 5.84 5.82 5.81 3.64 3.63 3.62
NO2
NO2
O
O
Ph
N3
6b
(1H, CDCl3, 400 MHz)
0.9 1.0 1.1 2.2 3.5 0.9 1.0 0.9 1.9
9.5 9.0 8.5 8.0 7.5 7.0 6.5 6.0 5.5 5.0 4.5 4.0 3.5 3.0 2.5 2.0 1.5 1.0 0.5
f1 (ppm)

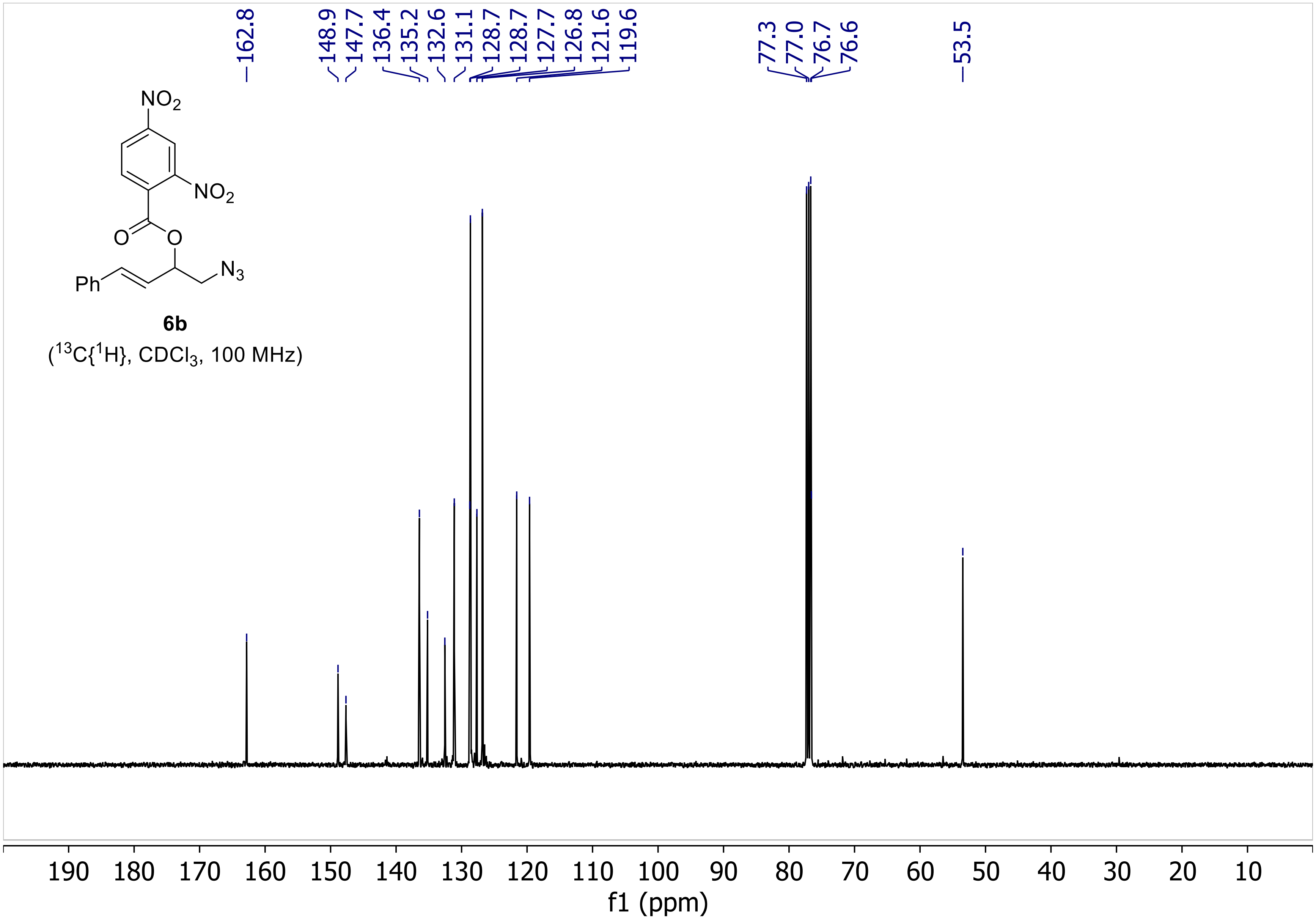
162.8
148.9
147.7
136.4
135.2
132.6
131.1
128.7
128.7
127.7
126.8
121.6
119.6
77.3
77.0
76.7
76.6
53.5
NO2
NO2
O
O
Ph
N3
6b
(13C{1H}, CDCl3, 100 MHz)
190 180 170 160 150 140 130 120 110 100 90 80 70 60 50 40 30 20 10
f1 (ppm)

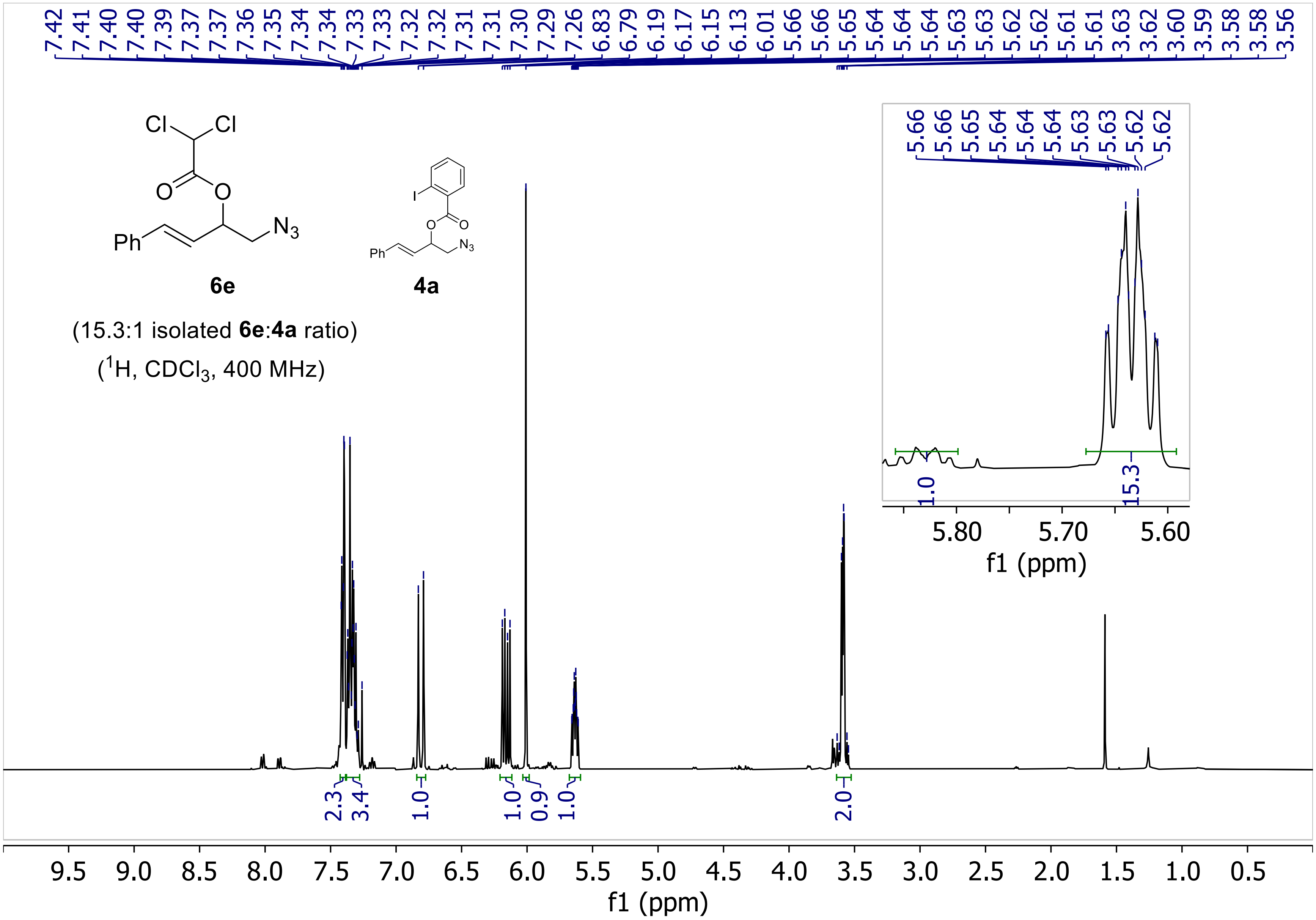
7.42 7.41 7.40 7.40 7.39 7.37 7.37 7.36 7.35 7.34 7.34 7.33 7.33 7.32 7.32 7.31 7.31 7.30 7.29 7.26 6.83 6.79 6.19 6.17 6.15 6.13 6.01 5.66 5.66 5.65 5.64 5.64 5.64 5.63 5.63 5.62 5.62 5.61 5.61 3.63 3.62 3.60 3.59 3.58 3.58 3.56
Cl Cl
O O
Ph N3
6e
I
O O
Ph N3
4a
(15.3:1 isolated 6e:4a ratio)
(1H, CDCl3, 400 MHz)
5.66 5.66 5.65 5.64 5.64 5.64 5.63 5.63 5.62 5.62
1.0
15.3
5.80 5.70 5.60
f1 (ppm)
2.3 3.4 1.0 1.0 0.9 1.0 2.0
9.5 9.0 8.5 8.0 7.5 7.0 6.5 6.0 5.5 5.0 4.5 4.0 3.5 3.0 2.5 2.0 1.5 1.0 0.5
f1 (ppm)

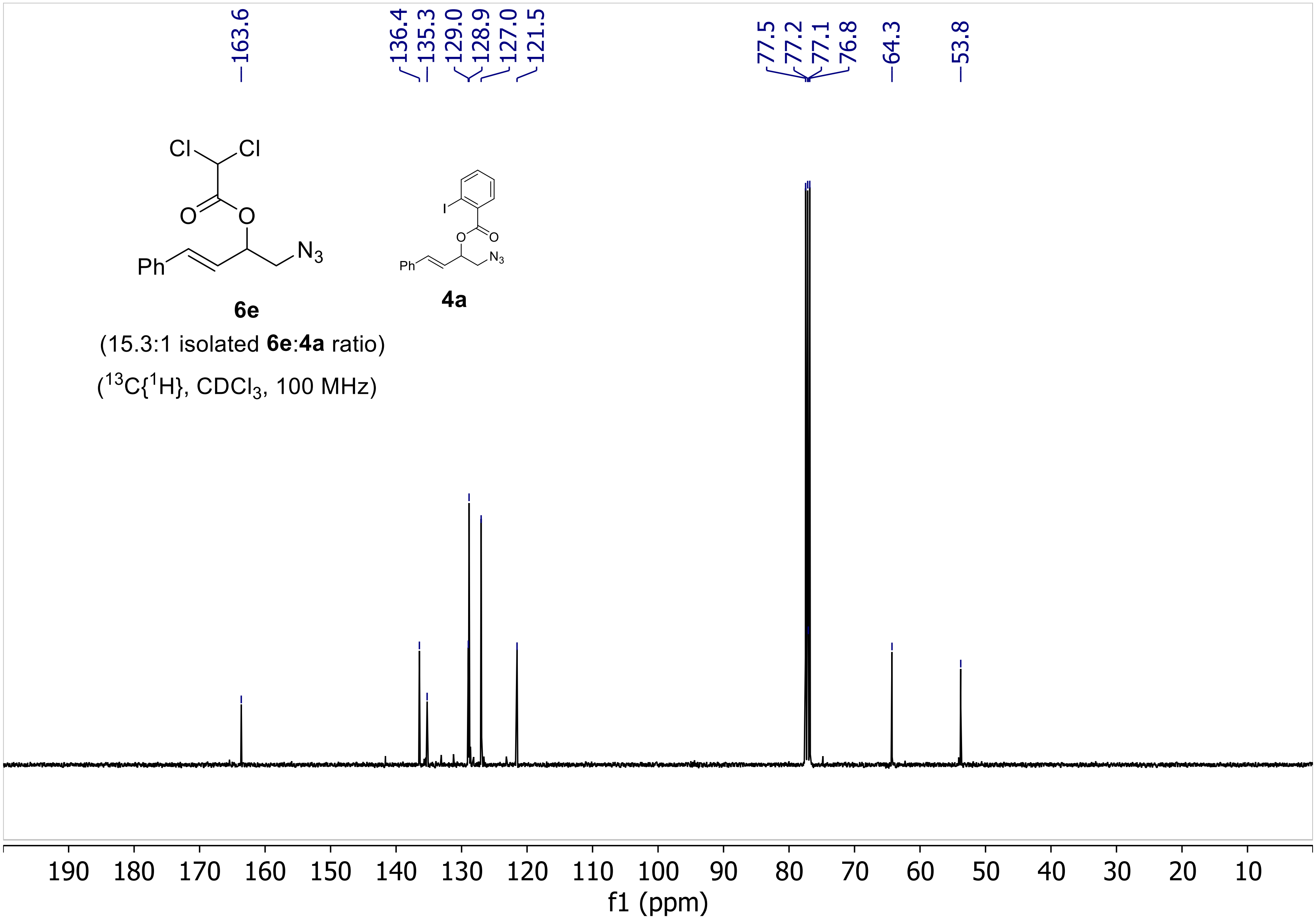
163.6
136.4
135.3
129.0
128.9
127.0
121.5
77.5
77.2
77.1
76.8
64.3
53.8
Cl
Cl
O
O
Ph
N_3
6e
I
O
O
Ph
N_3
4a
(15.3:1 isolated **6e**:**4a** ratio)
($^{13}C\{^{1}H\}$, $CDCl_3$, 100 MHz)
190 180 170 160 150 140 130 120 110 100 90 80 70 60 50 40 30 20 10
f1 (ppm)

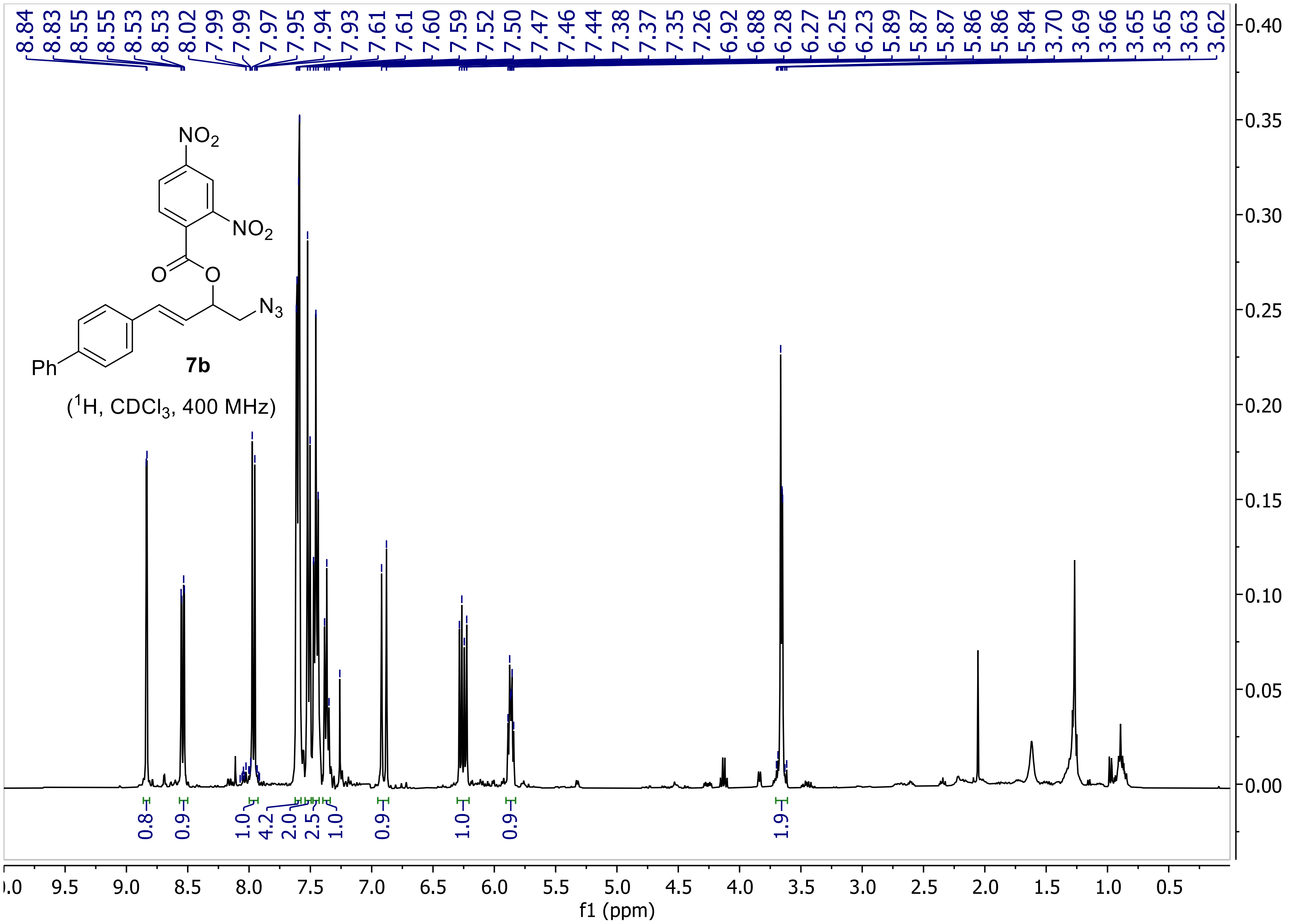

8.84 8.83 8.55 8.55 8.53 8.53 8.02 7.99 7.99 7.97 7.95 7.94 7.93 7.61 7.61 7.60 7.59 7.52 7.50 7.47 7.46 7.44 7.38 7.37 7.35 7.26 6.92 6.88 6.28 6.27 6.25 6.23 5.89 5.87 5.87 5.86 5.86 5.84 3.70 3.69 3.66 3.65 3.65 3.63 3.62
NO2
NO2
O
O
N3
Ph
7b
(1H, CDCl3, 400 MHz)
0.8 0.9 1.0 4.2 2.0 2.5 1.0 0.9 1.0 0.9 1.9
f1 (ppm)

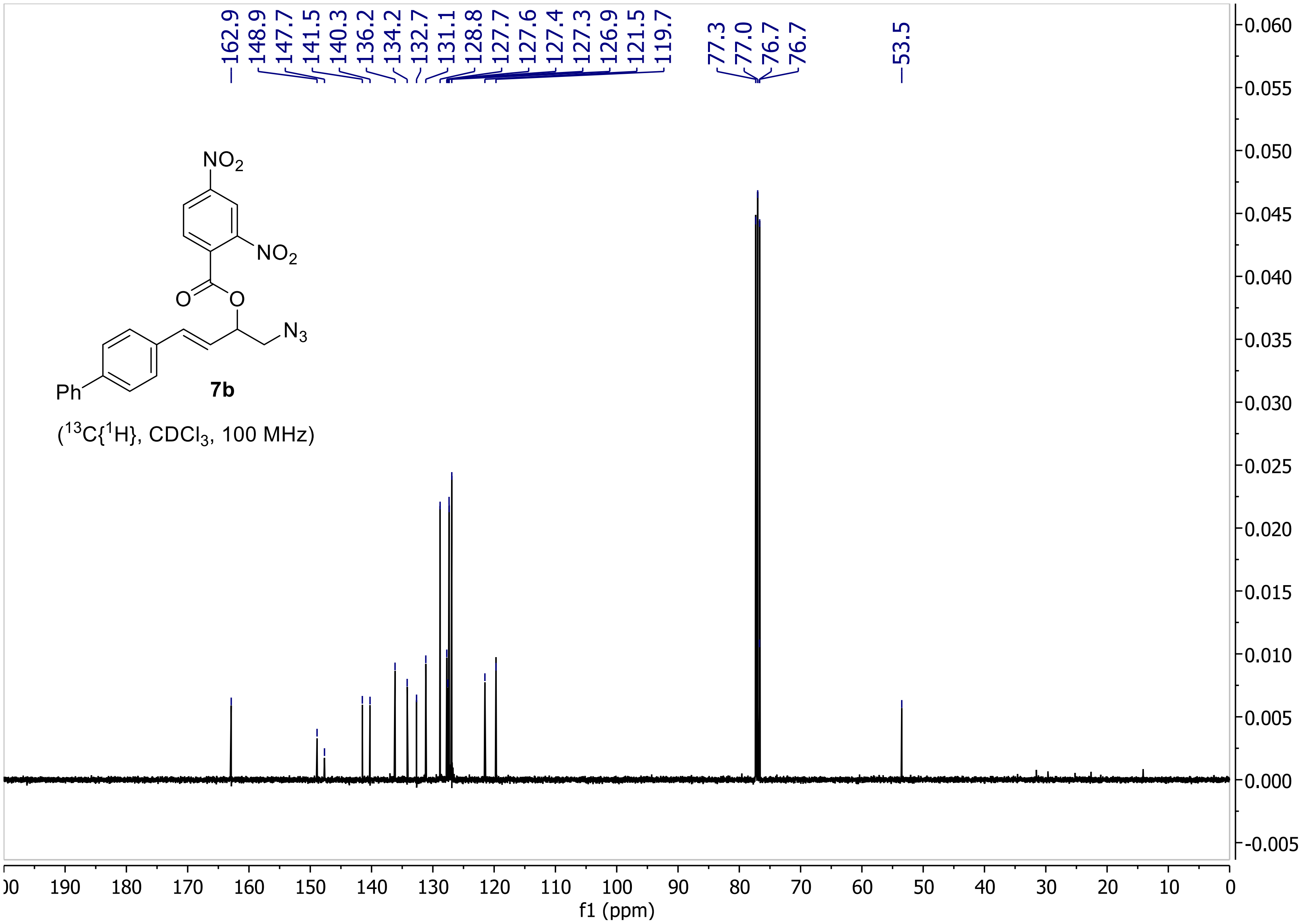
162.9
148.9
147.7
141.5
140.3
136.2
134.2
132.7
131.1
128.8
127.7
127.6
127.4
127.3
126.9
121.5
119.7
77.3
77.0
76.7
76.7
53.5
NO2
NO2
O
O
N3
Ph
7b
(13C{1H}, CDCl3, 100 MHz)
f1 (ppm)

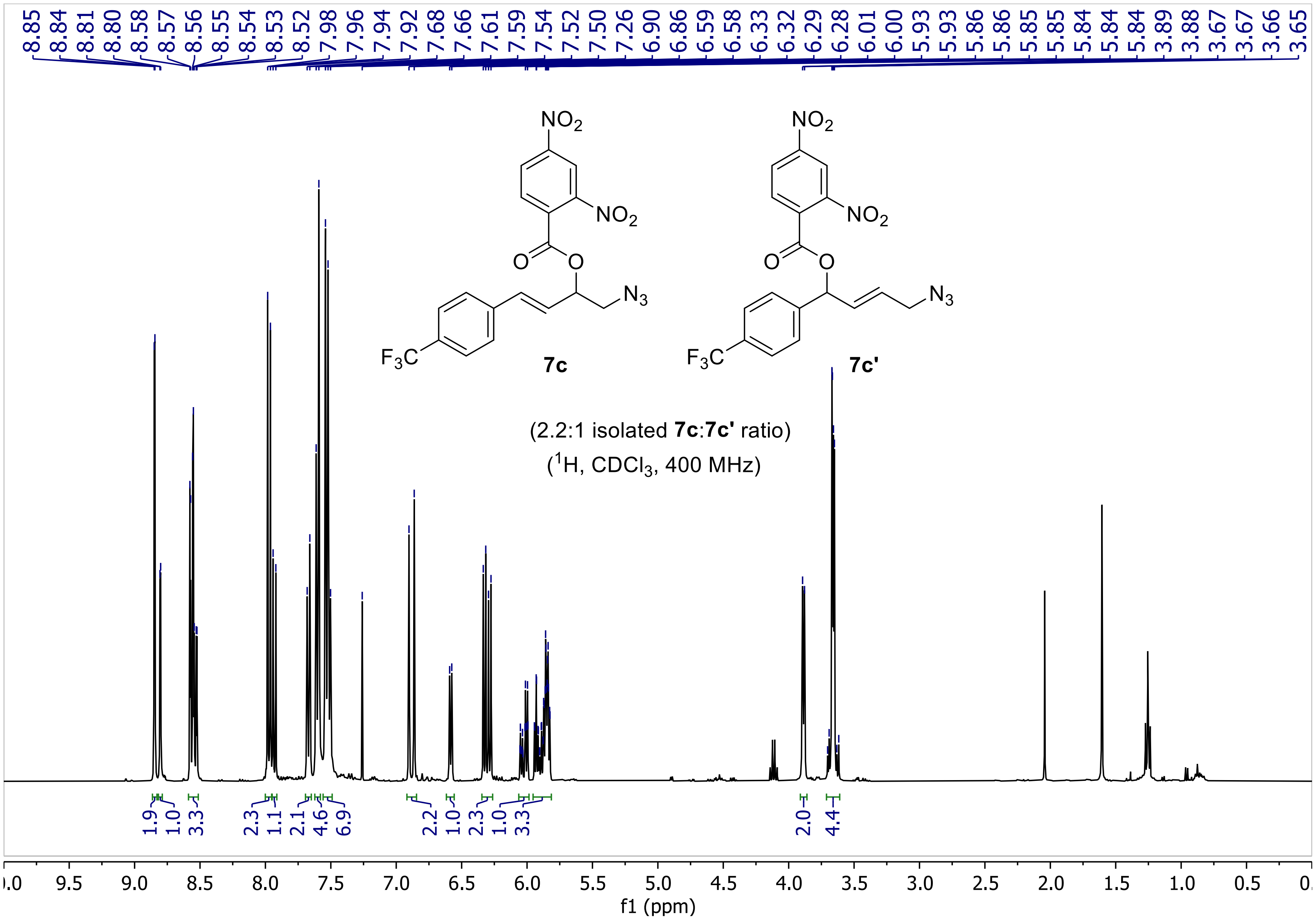

8.85 8.84 8.81 8.80 8.58 8.57 8.56 8.55 8.54 8.53 8.52 7.98 7.96 7.94 7.92 7.68 7.66 7.61 7.59 7.54 7.52 7.50 7.26 6.90 6.86 6.59 6.58 6.33 6.32 6.29 6.28 6.01 6.00 5.93 5.93 5.86 5.86 5.85 5.85 5.84 5.84 5.84 3.89 3.88 3.67 3.67 3.66 3.65
NO2
NO2
O
O
N3
F3C
7c
7c'
(2.2:1 isolated 7c:7c' ratio)
(1H, CDCl3, 400 MHz)
1.9 1.0 3.3 2.3 1.1 2.1 4.6 6.9 2.2 1.0 2.3 1.0 3.3 2.0 4.4
9.5 9.0 8.5 8.0 7.5 7.0 6.5 6.0 5.5 5.0 4.5 4.0 3.5 3.0 2.5 2.0 1.5 1.0 0.5
f1 (ppm)

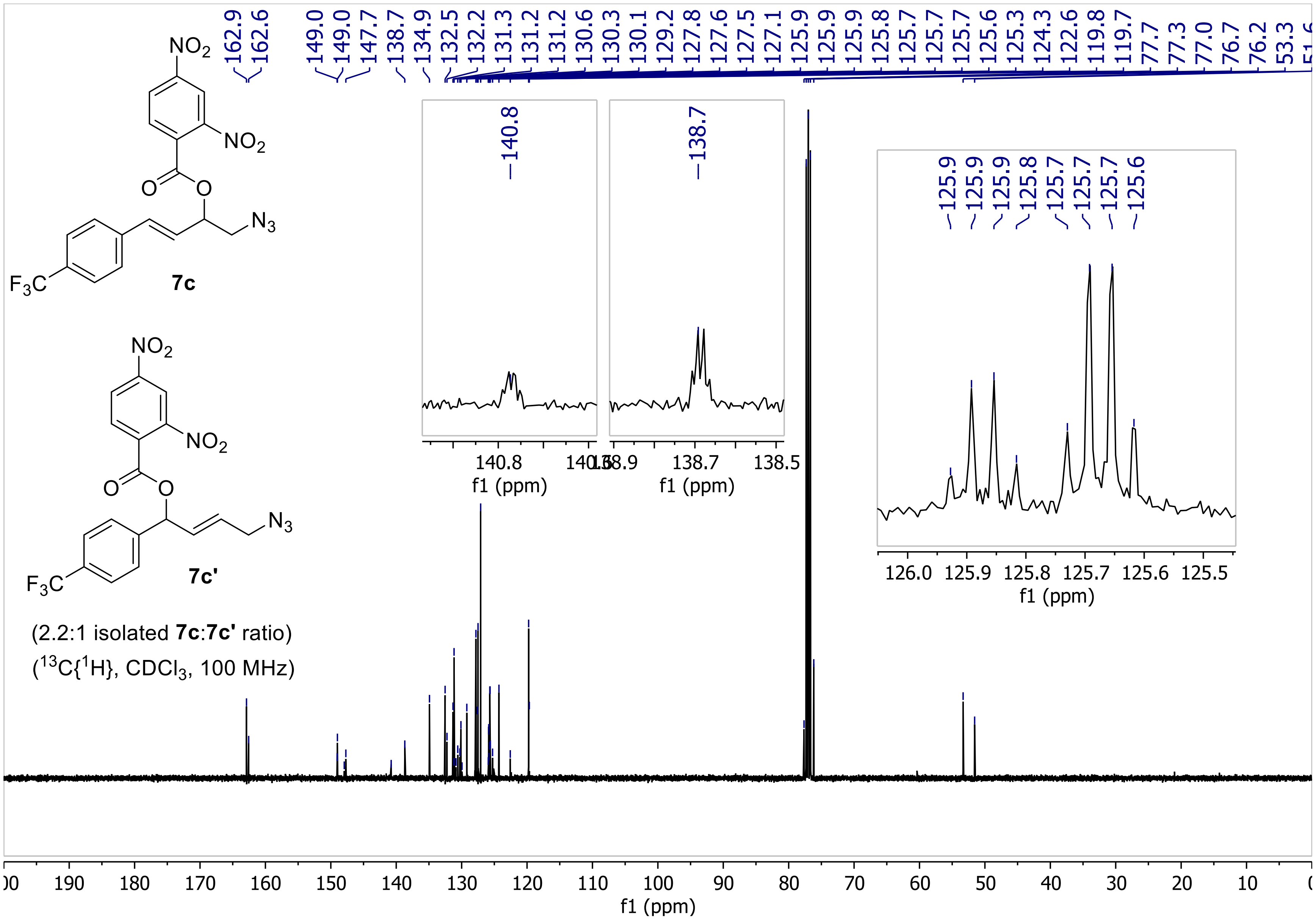

162.9
162.6
149.0
149.0
147.7
138.7
134.9
132.5
132.2
131.3
131.2
131.2
130.6
130.3
130.1
129.2
127.8
127.6
127.5
127.1
125.9
125.9
125.9
125.8
125.7
125.7
125.7
125.6
125.3
124.3
122.6
119.8
119.7
77.7
77.3
77.0
76.7
76.2
53.3
NO2
NO2
O
O
N3
F3C
7c
NO2
NO2
O
O
N3
F3C
7c'
(2.2:1 isolated 7c:7c' ratio)
(13C{1H}, CDCl3, 100 MHz)
140.8
138.7
f1 (ppm)

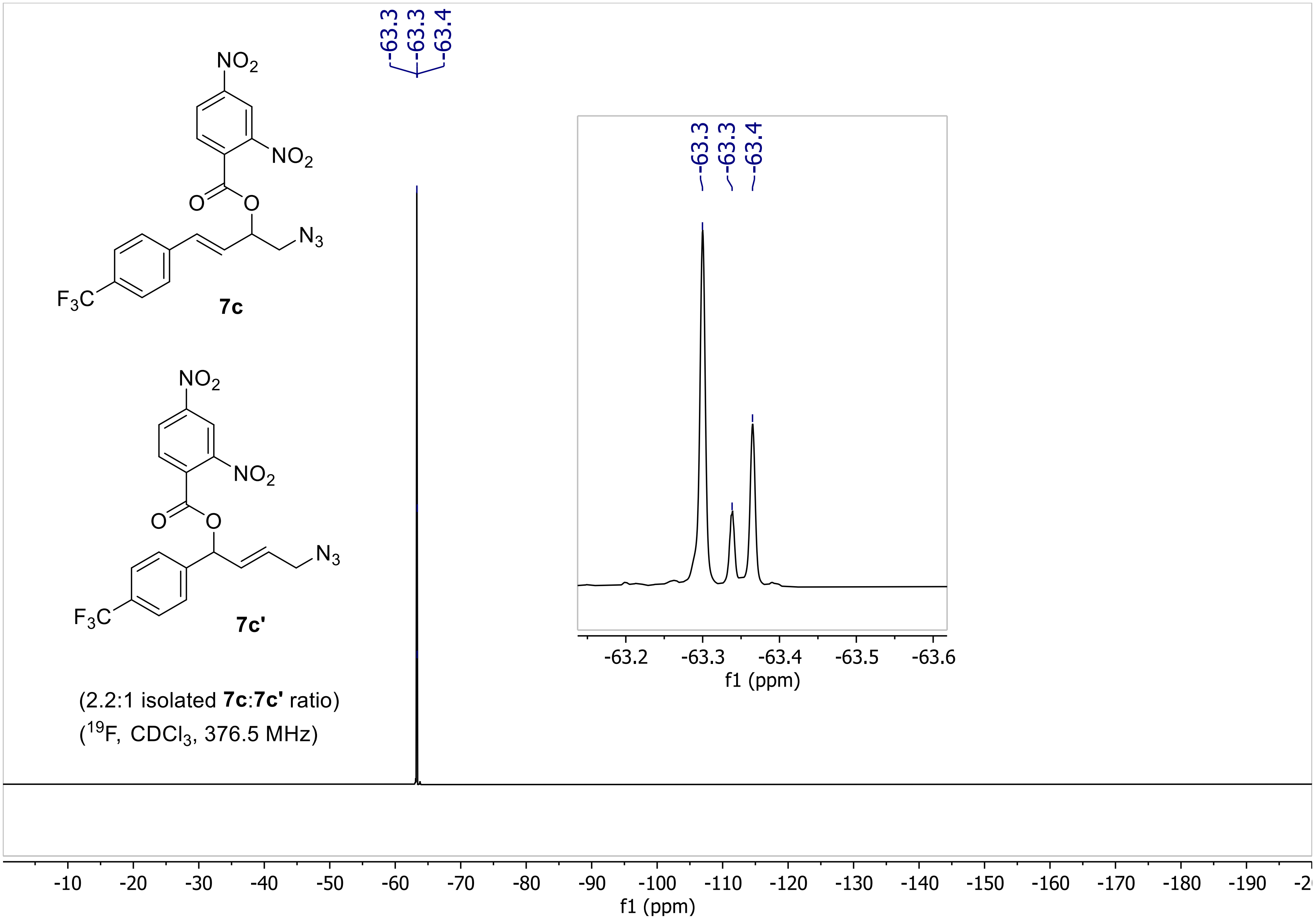

NO2
NO2
O
O
N3
F3C
7c
NO2
NO2
O
O
N3
F3C
7c'
(2.2:1 isolated 7c:7c' ratio)
(19F, CDCl3, 376.5 MHz)
-63.3
-63.3
-63.4
-63.2
-63.3
-63.4
-63.5
-63.6
f1 (ppm)
-10
-20
-30
-40
-50
-60
-70
-80
-90
-100
-110
-120
-130
-140
-150
-160
-170
-180
-190
f1 (ppm)

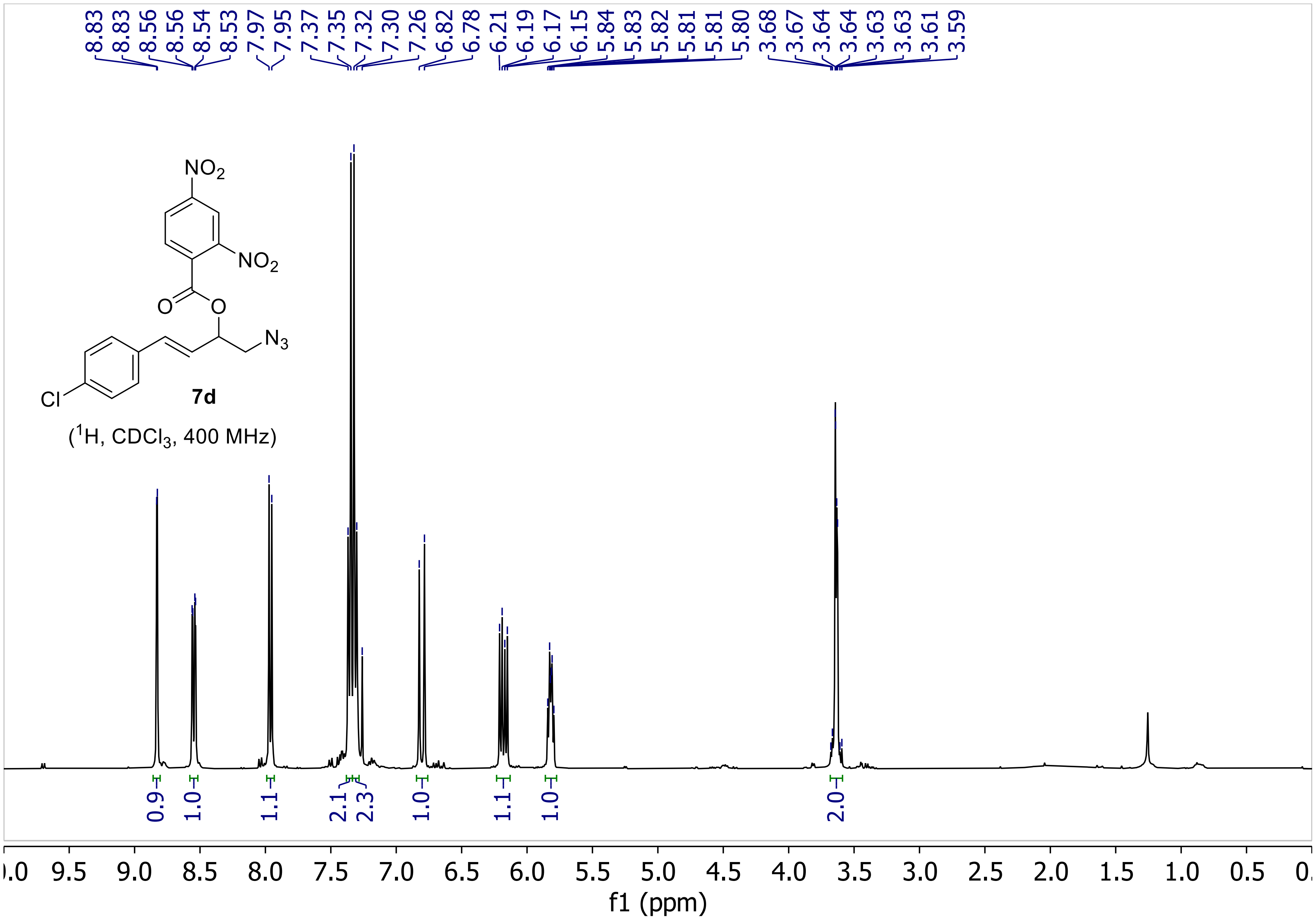
8.83 8.83 8.56 8.56 8.54 8.53 7.97 7.95 7.37 7.35 7.32 7.30 7.26 6.82 6.78 6.21 6.19 6.17 6.15 5.84 5.83 5.82 5.81 5.81 5.80 3.68 3.67 3.64 3.64 3.63 3.63 3.61 3.59
NO2
NO2
O
O
N3
Cl
7d
(1H, CDCl3, 400 MHz)
0.9 1.0 1.1 2.1 2.3 1.0 1.1 1.0 2.0
f1 (ppm)

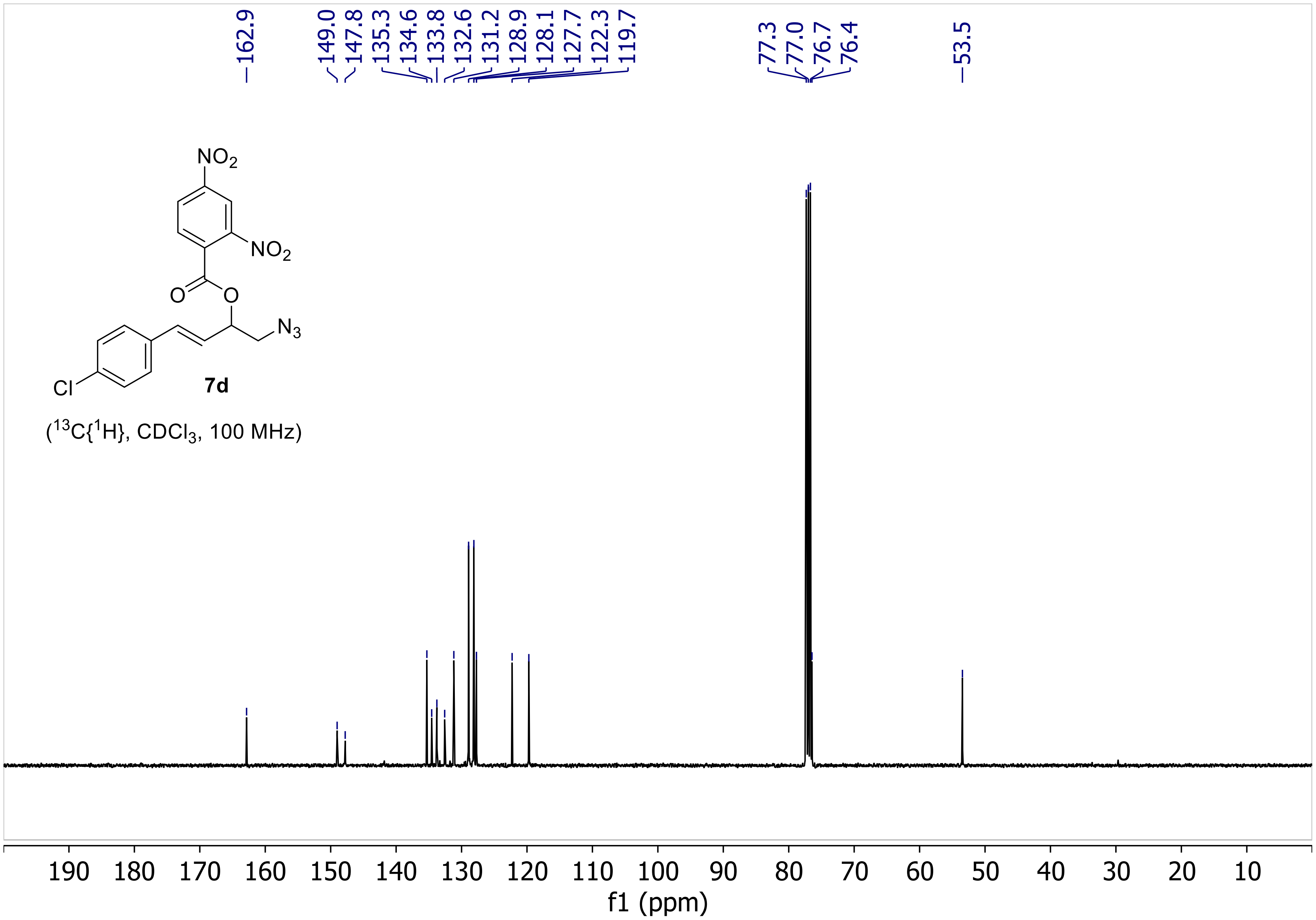
162.9
149.0
147.8
135.3
134.6
133.8
132.6
131.2
128.9
128.1
127.7
122.3
119.7
77.3
77.0
76.7
76.4
53.5
NO2
NO2
O
O
N3
Cl
7d
(13C{1H}, CDCl3, 100 MHz)
190 180 170 160 150 140 130 120 110 100 90 80 70 60 50 40 30 20 10
f1 (ppm)

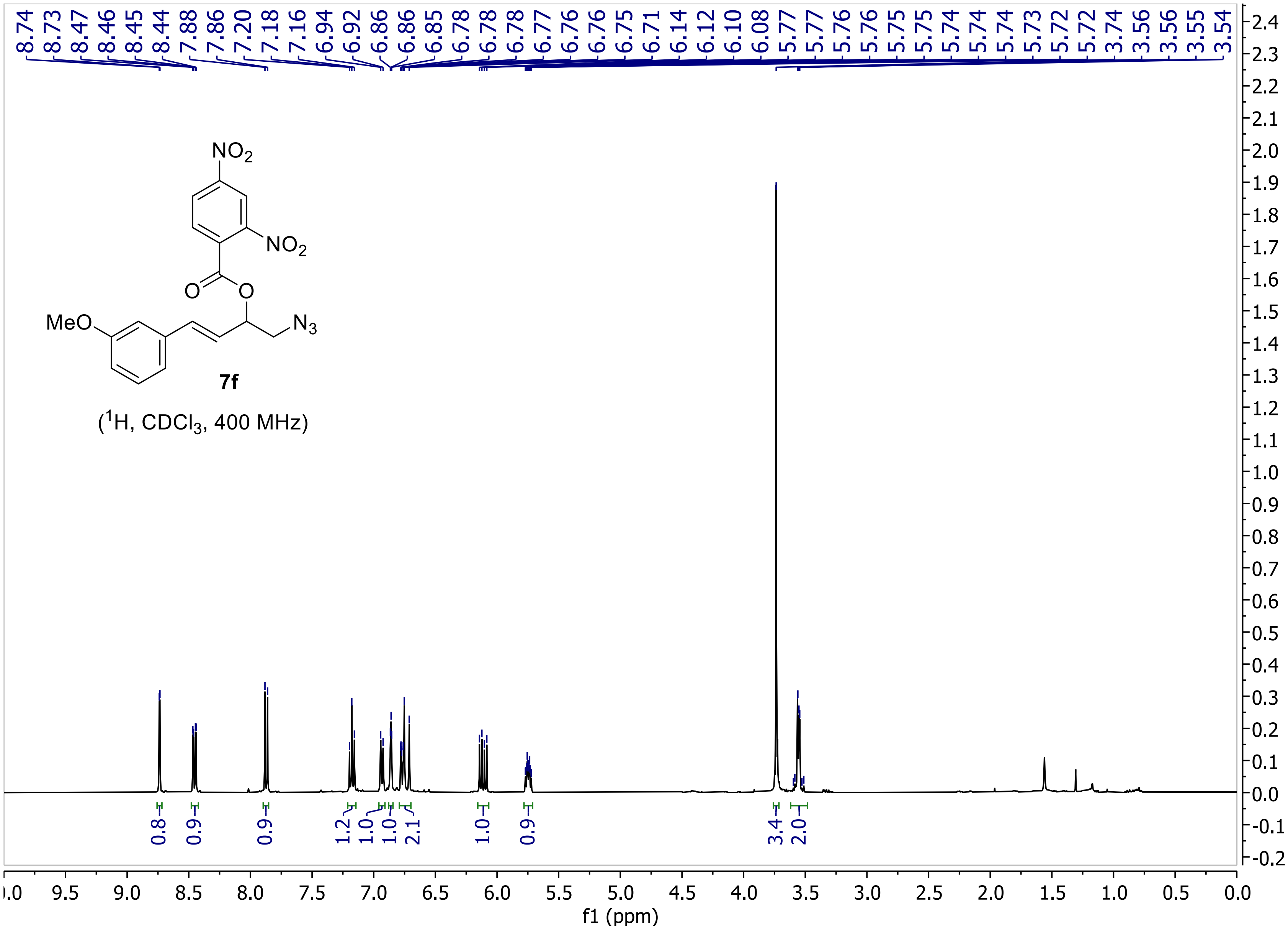
8.74
8.73
8.47
8.46
8.45
8.44
7.88
7.86
7.20
7.18
7.16
6.94
6.92
6.86
6.86
6.85
6.78
6.78
6.78
6.77
6.76
6.76
6.75
6.71
6.14
6.12
6.10
6.08
5.77
5.77
5.76
5.76
5.75
5.75
5.74
5.74
5.74
5.73
5.72
5.72
3.74
3.56
3.56
3.55
3.54
NO2
NO2
O
O
MeO
N3
7f
(1H, CDCl3, 400 MHz)
0.8
0.9
0.9
1.2
1.0
1.0
2.1
1.0
0.9
3.4
2.0
f1 (ppm)

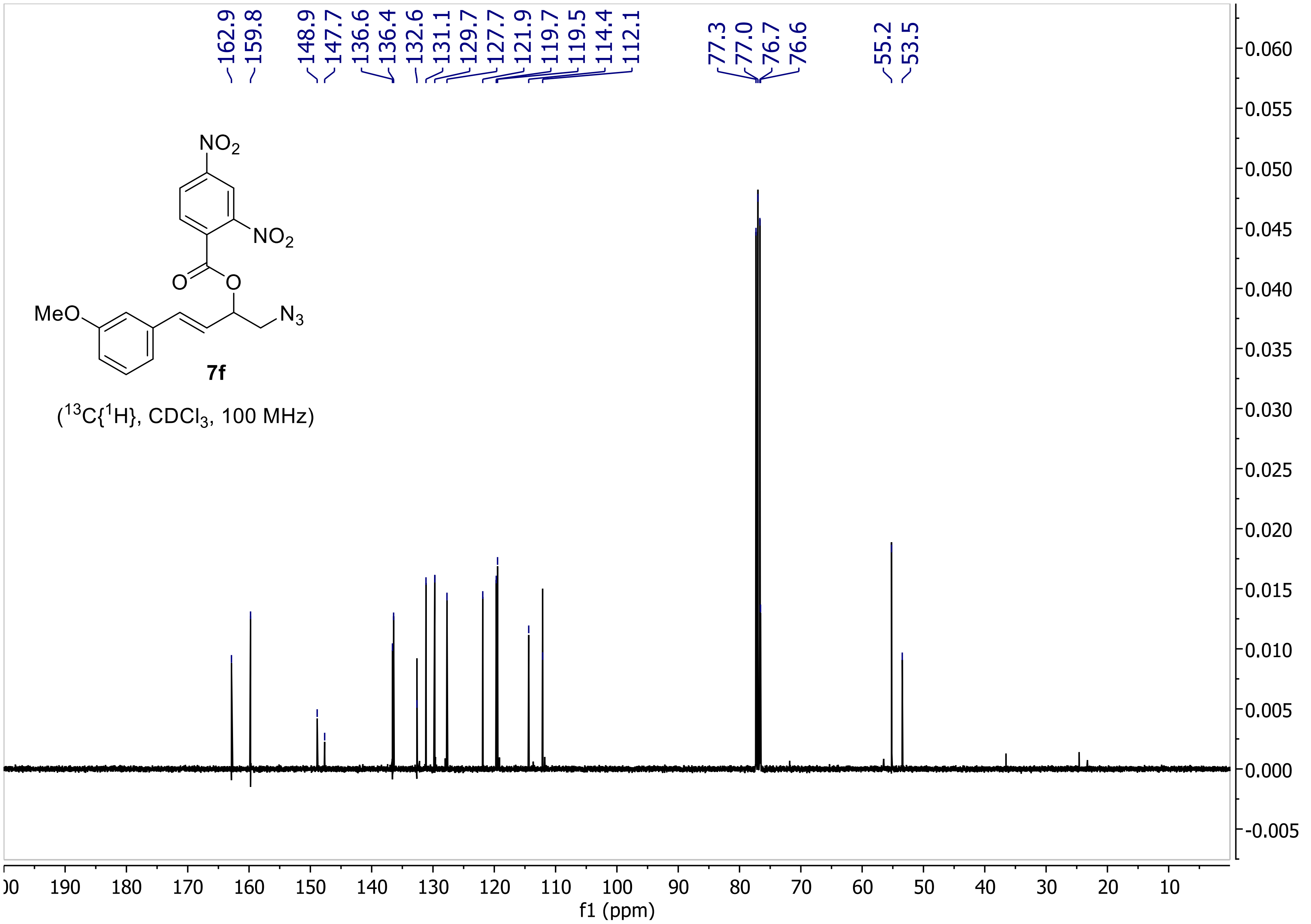
162.9
159.8
148.9
147.7
136.6
136.4
132.6
131.1
129.7
127.7
121.9
119.7
119.5
114.4
112.1
77.3
77.0
76.7
76.6
55.2
53.5
NO2
NO2
O
O
MeO
N3
7f
(13C{1H}, CDCl3, 100 MHz)
f1 (ppm)

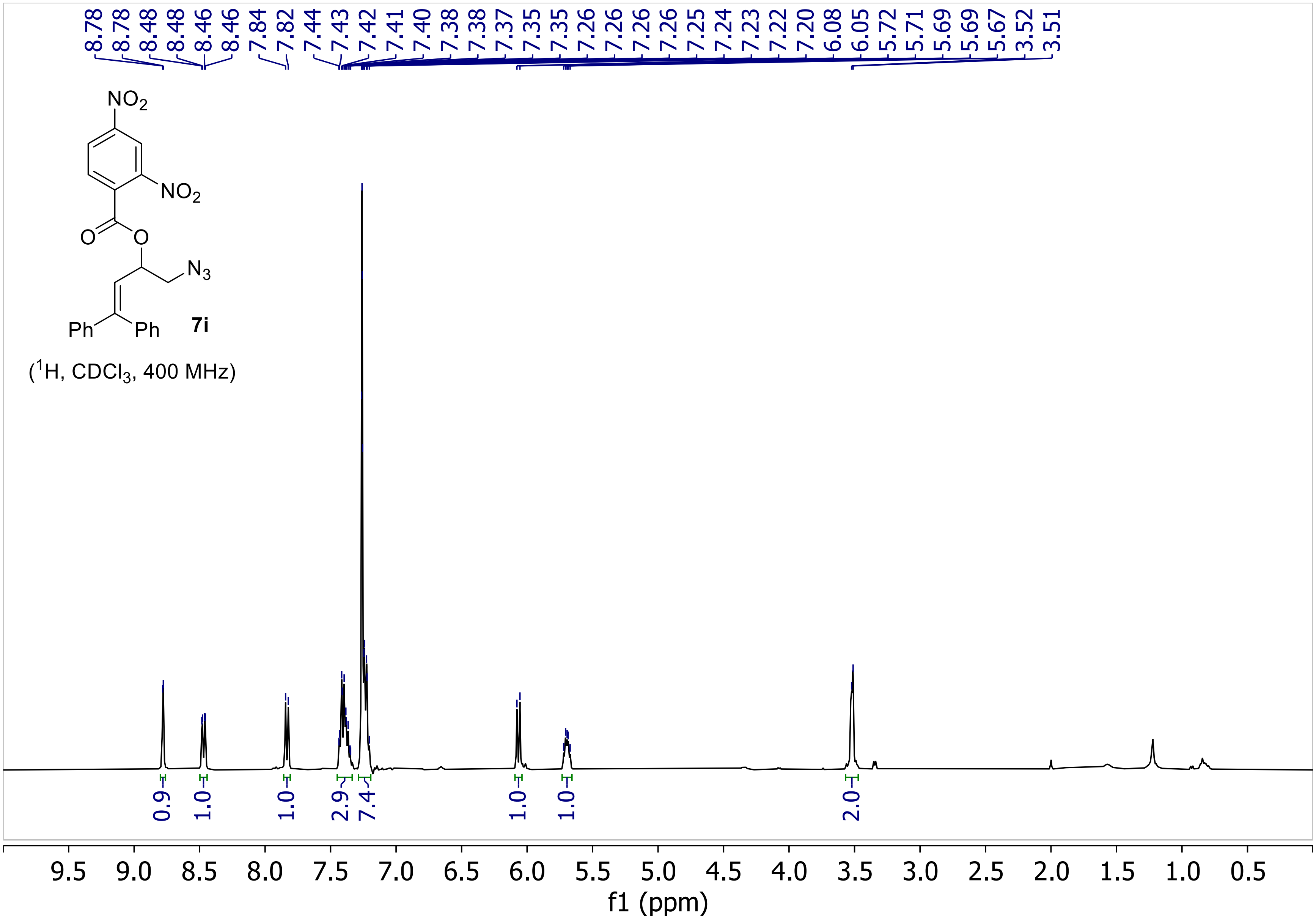

8.78
8.78
8.48
8.48
8.46
8.46
7.84
7.82
7.44
7.43
7.42
7.41
7.40
7.38
7.38
7.37
7.35
7.35
7.26
7.26
7.26
7.26
7.25
7.24
7.23
7.22
7.20
6.08
6.05
5.72
5.71
5.69
5.69
5.67
3.52
3.51
NO2
NO2
O
O
N3
Ph
Ph
7i
(1H, CDCl3, 400 MHz)
0.9
1.0
1.0
2.9
7.4
1.0
1.0
2.0
9.5 9.0 8.5 8.0 7.5 7.0 6.5 6.0 5.5 5.0 4.5 4.0 3.5 3.0 2.5 2.0 1.5 1.0 0.5
f1 (ppm)

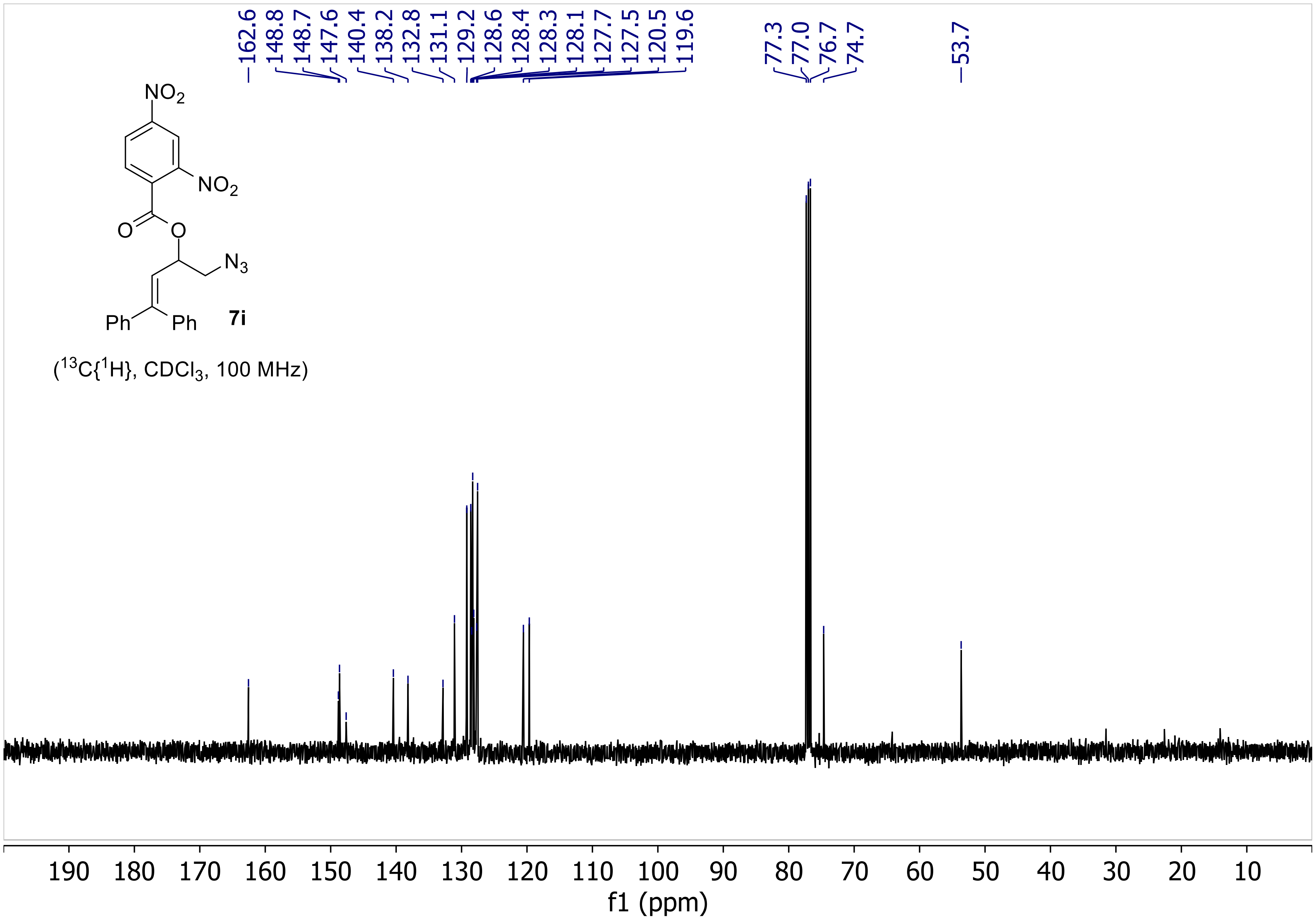
162.6
148.8
148.7
147.6
140.4
138.2
132.8
131.1
129.2
128.6
128.4
128.3
128.1
127.7
127.5
120.5
119.6
77.3
77.0
76.7
74.7
53.7
NO2
NO2
O
O
N3
Ph
Ph
7i
(13C{1H}, CDCl3, 100 MHz)
190 180 170 160 150 140 130 120 110 100 90 80 70 60 50 40 30 20 10
f1 (ppm)

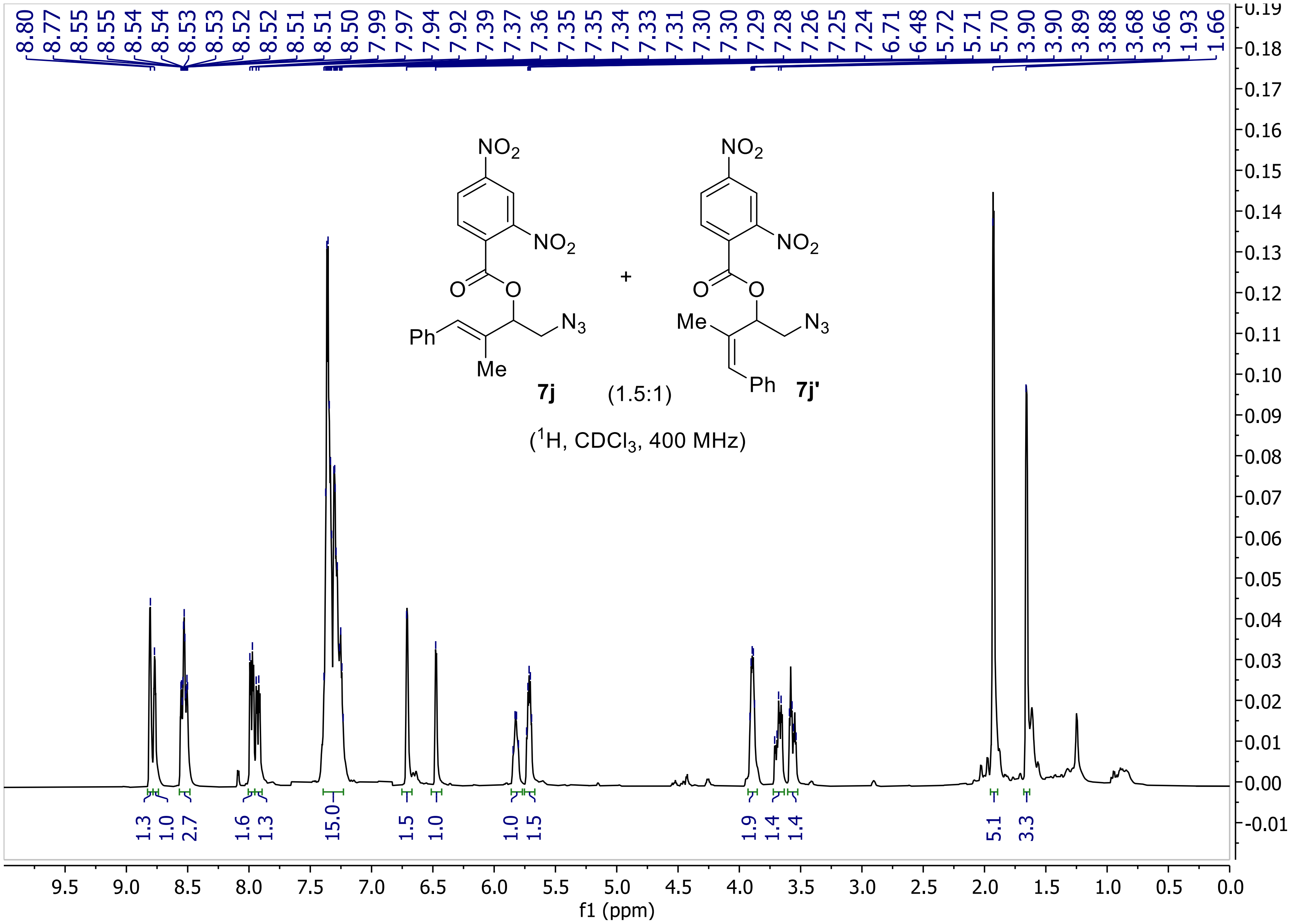

8.80 8.77 8.55 8.55 8.54 8.54 8.53 8.53 8.52 8.52 8.51 8.51 8.50 7.99 7.97 7.94 7.92 7.39 7.37 7.36 7.35 7.35 7.34 7.33 7.31 7.30 7.30 7.29 7.28 7.26 7.25 7.24 6.71 6.48 5.72 5.71 5.70 3.90 3.90 3.89 3.88 3.68 3.66 1.93 1.66
NO2
NO2
O
O
N3
Ph
Me
7j
+
Me
Ph
7j'
(1.5:1)
(1H, CDCl3, 400 MHz)
1.3 1.0 2.7 1.6 1.3 15.0 1.5 1.0 1.0 1.5 1.9 1.4 1.4 5.1 3.3
9.5 9.0 8.5 8.0 7.5 7.0 6.5 6.0 5.5 5.0 4.5 4.0 3.5 3.0 2.5 2.0 1.5 1.0 0.5 0.0
f1 (ppm)

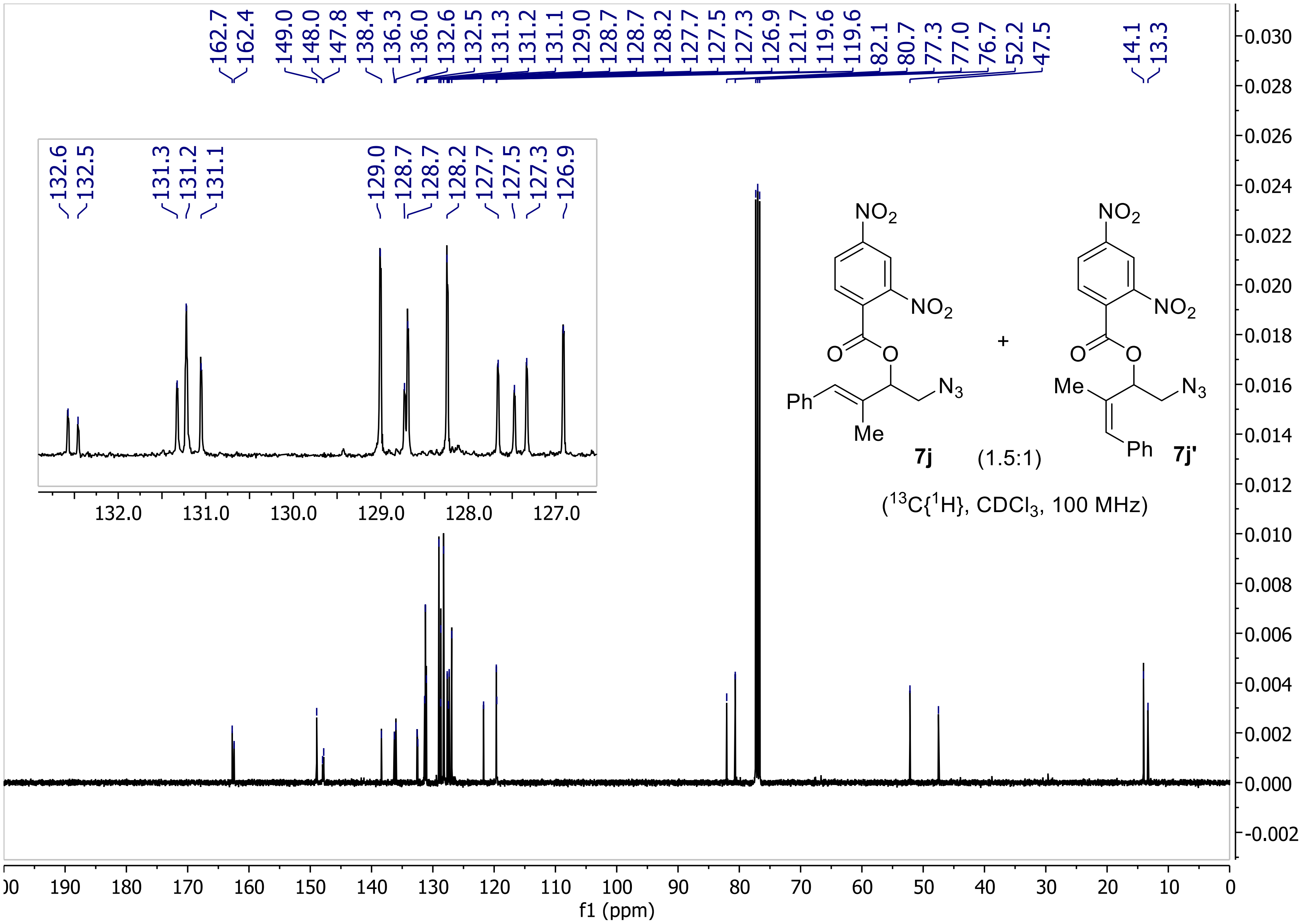
162.7
162.4
149.0
148.0
147.8
138.4
136.3
136.0
132.6
132.5
131.3
131.2
131.1
129.0
128.7
128.7
128.2
127.7
127.5
127.3
126.9
121.7
119.6
119.6
82.1
80.7
77.3
77.0
76.7
52.2
47.5
14.1
13.3
NO_2
NO_2
+
O
O
Ph
N_3
Me
Me
Ph
7j
(1.5:1)
7j'
($^{13}C\{^1H\}$, $CDCl_3$, 100 MHz)
f1 (ppm)

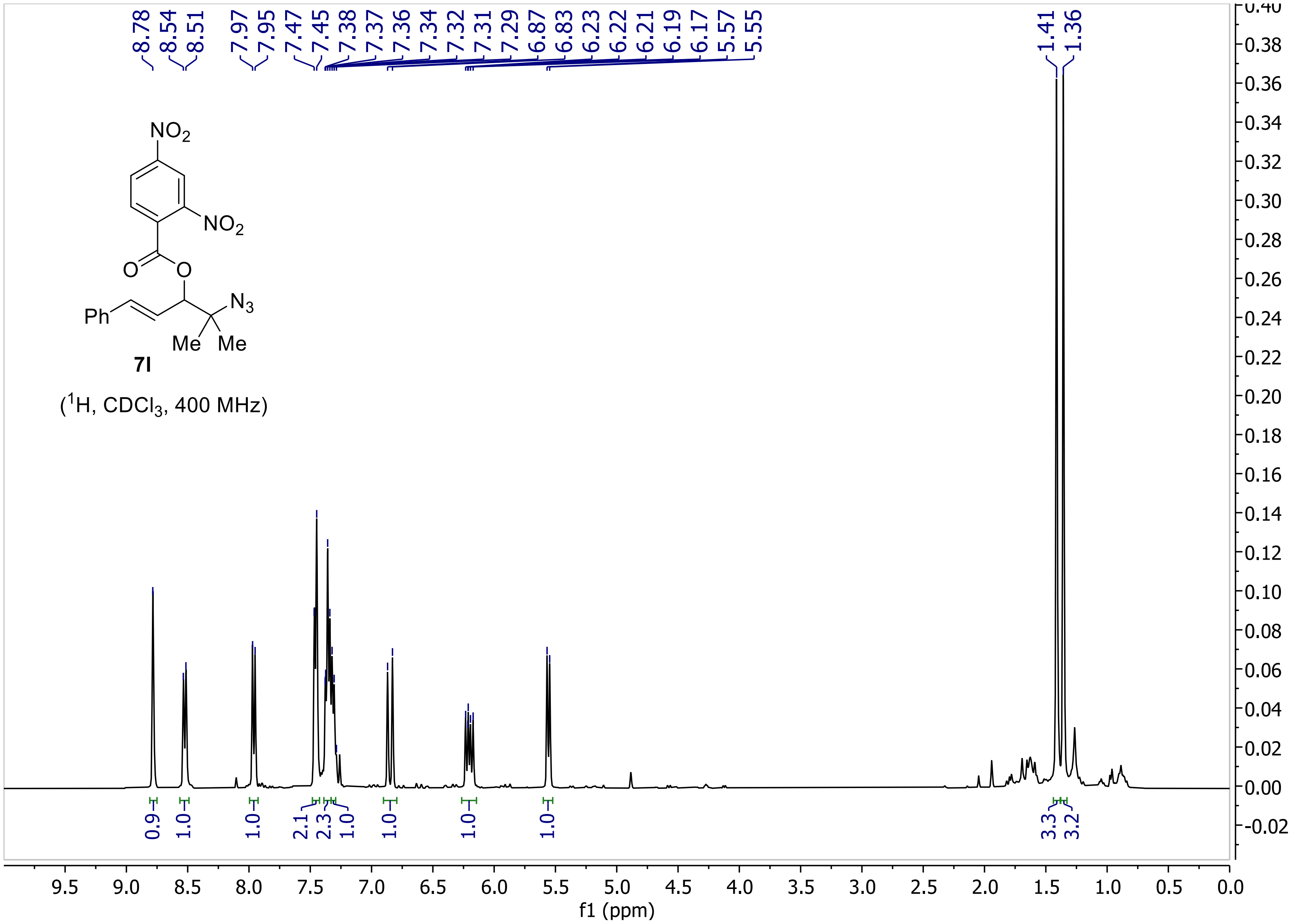
8.78
8.54
8.51
7.97
7.95
7.47
7.45
7.38
7.37
7.36
7.34
7.32
7.31
7.29
6.87
6.83
6.23
6.22
6.21
6.19
6.17
5.57
5.55
1.41
1.36
NO2
NO2
O
O
N3
Ph
Me
Me
7l
(1H, CDCl3, 400 MHz)
0.9
1.0
1.0
2.1
2.3
1.0
1.0
1.0
1.0
3.3
3.2
f1 (ppm)

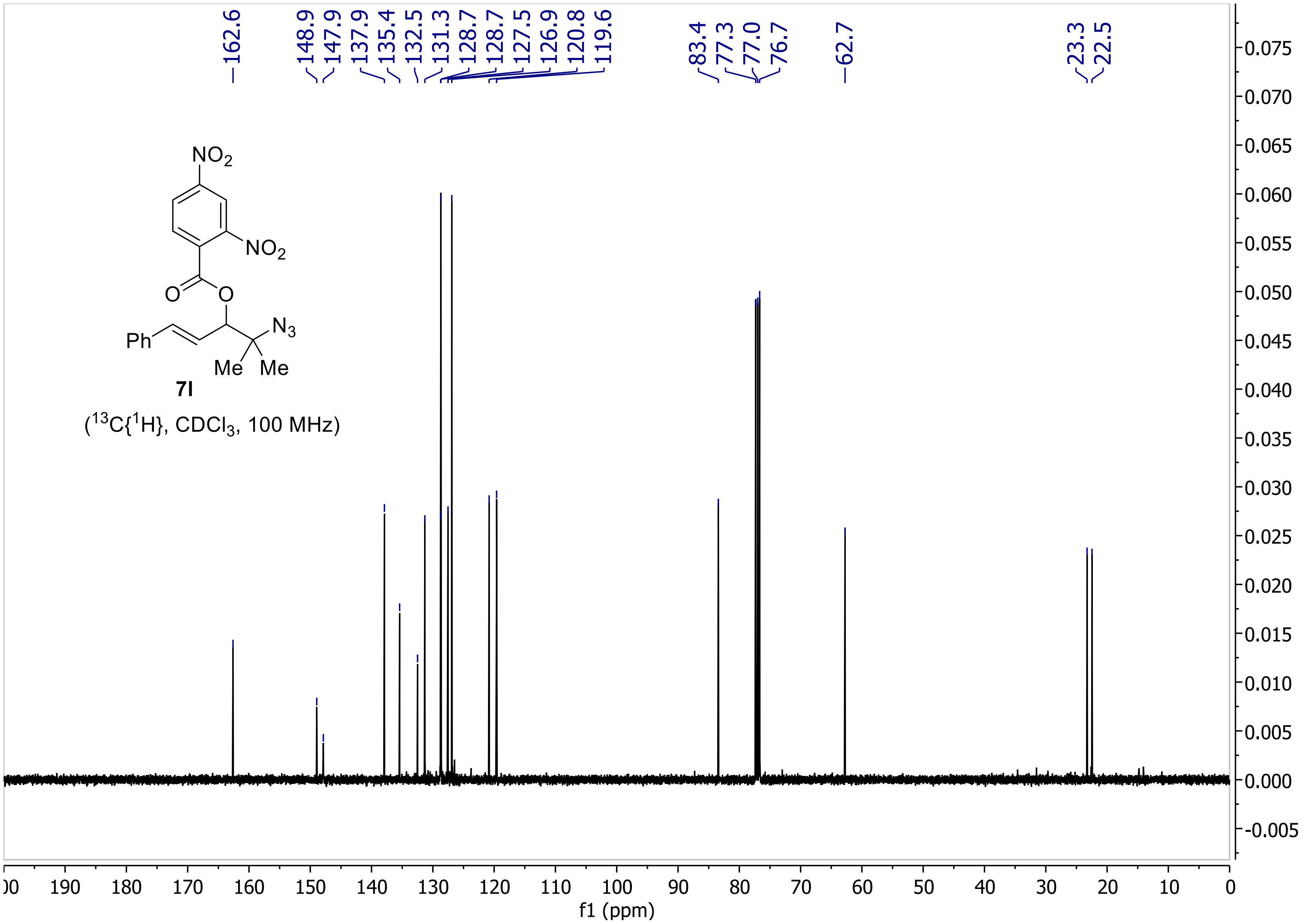
162.6
148.9
147.9
137.9
135.4
132.5
131.3
128.7
128.7
127.5
126.9
120.8
119.6
83.4
77.3
77.0
76.7
62.7
23.3
22.5
NO2
NO2
O
O
N3
Ph
Me
Me
7l
(13C{1H}, CDCl3, 100 MHz)
f1 (ppm)

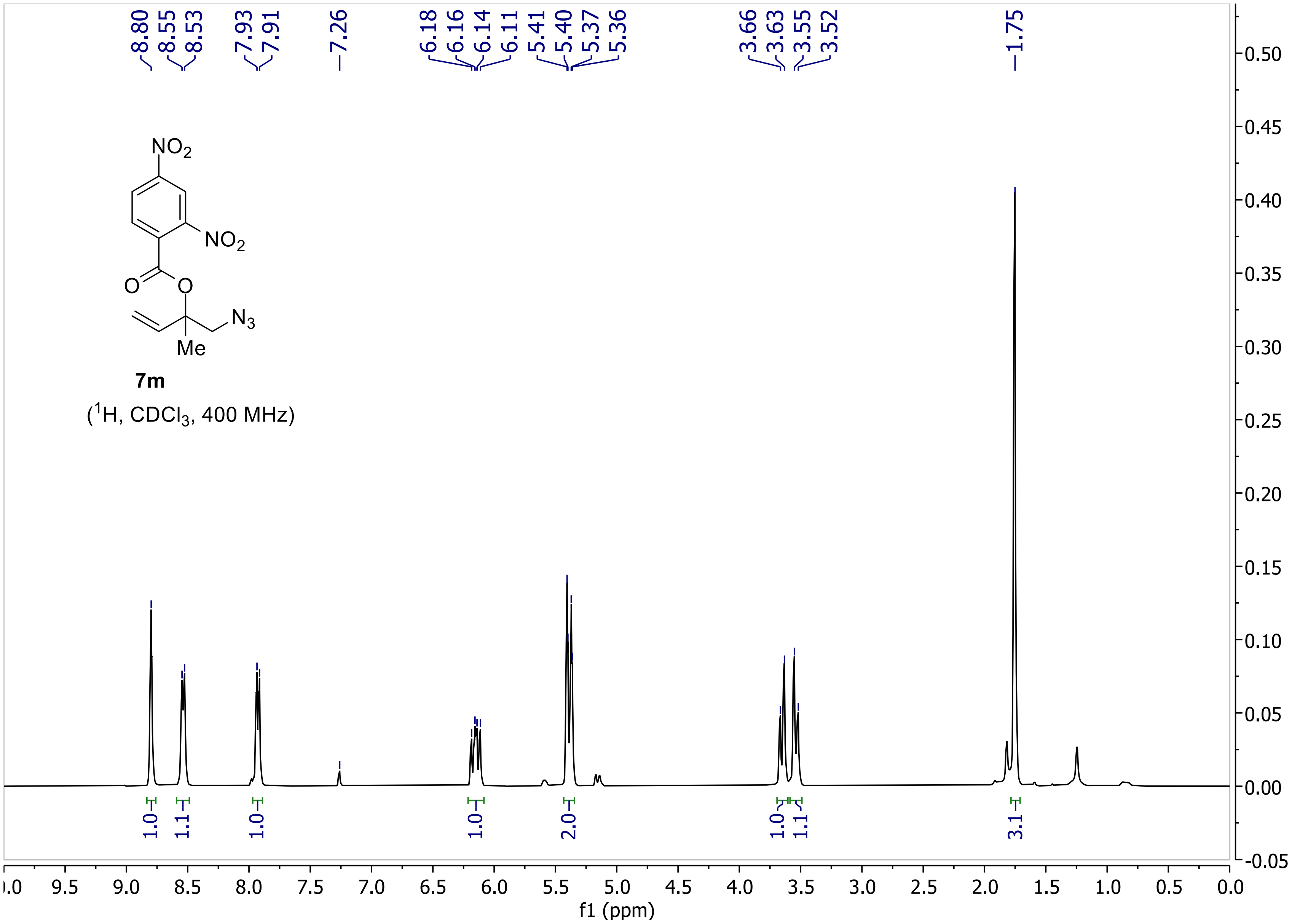

8.80
8.55
8.53
7.93
7.91
7.26
6.18
6.16
6.14
6.11
5.41
5.40
5.37
5.36
3.66
3.63
3.55
3.52
1.75
NO_2
NO_2
O
O
N_3
Me
7m
(^{1}H, $CDCl_3$, 400 MHz)
1.0
1.1
1.0
1.0
2.0
1.0
1.1
3.1
f1 (ppm)

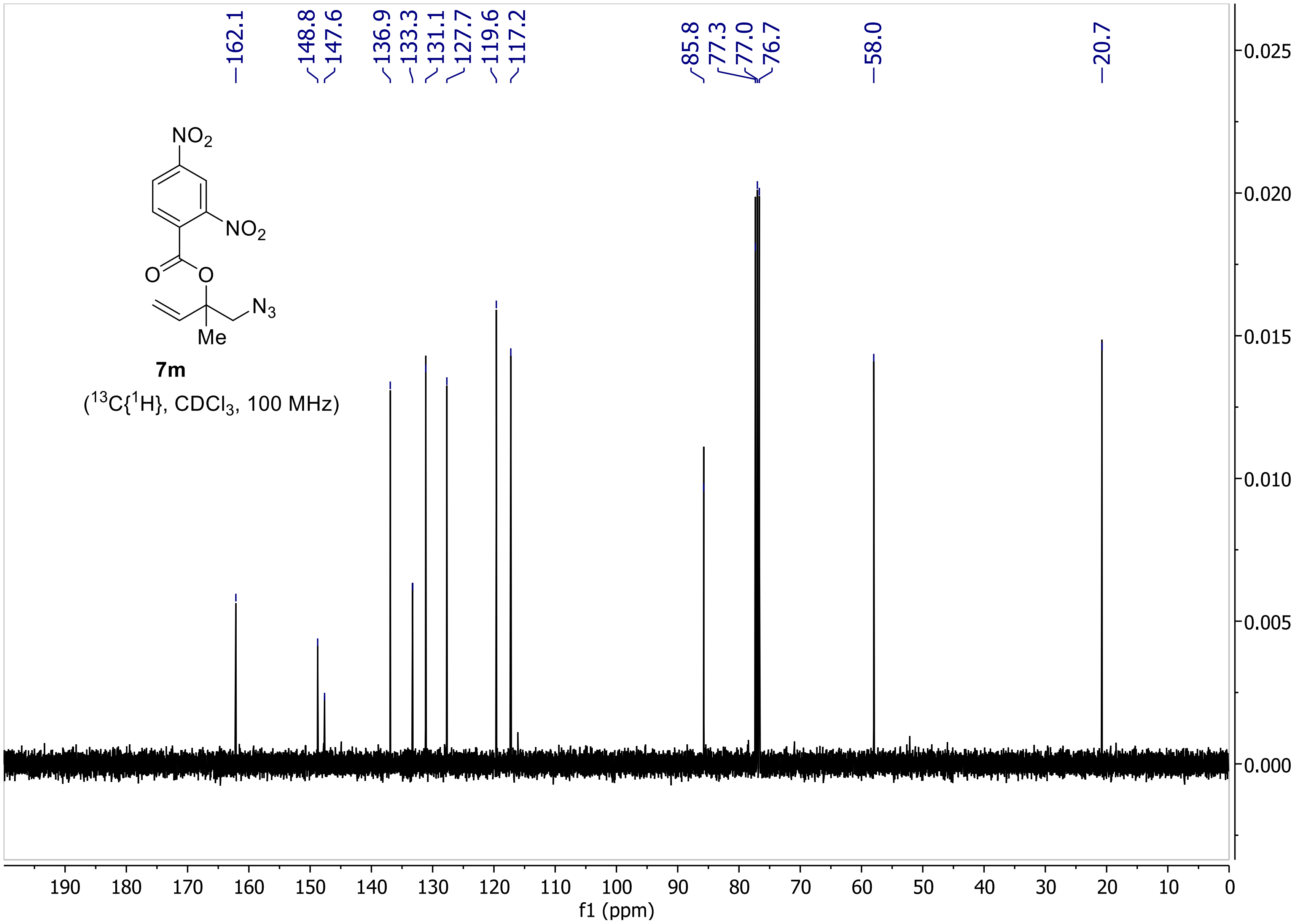
162.1
148.8
147.6
136.9
133.3
131.1
127.7
119.6
117.2
85.8
77.3
77.0
76.7
58.0
20.7
NO_2
NO_2
O
O
N_3
Me
7m
($^{13}C\{^{1}H\}$, $CDCl_3$, 100 MHz)
f1 (ppm)

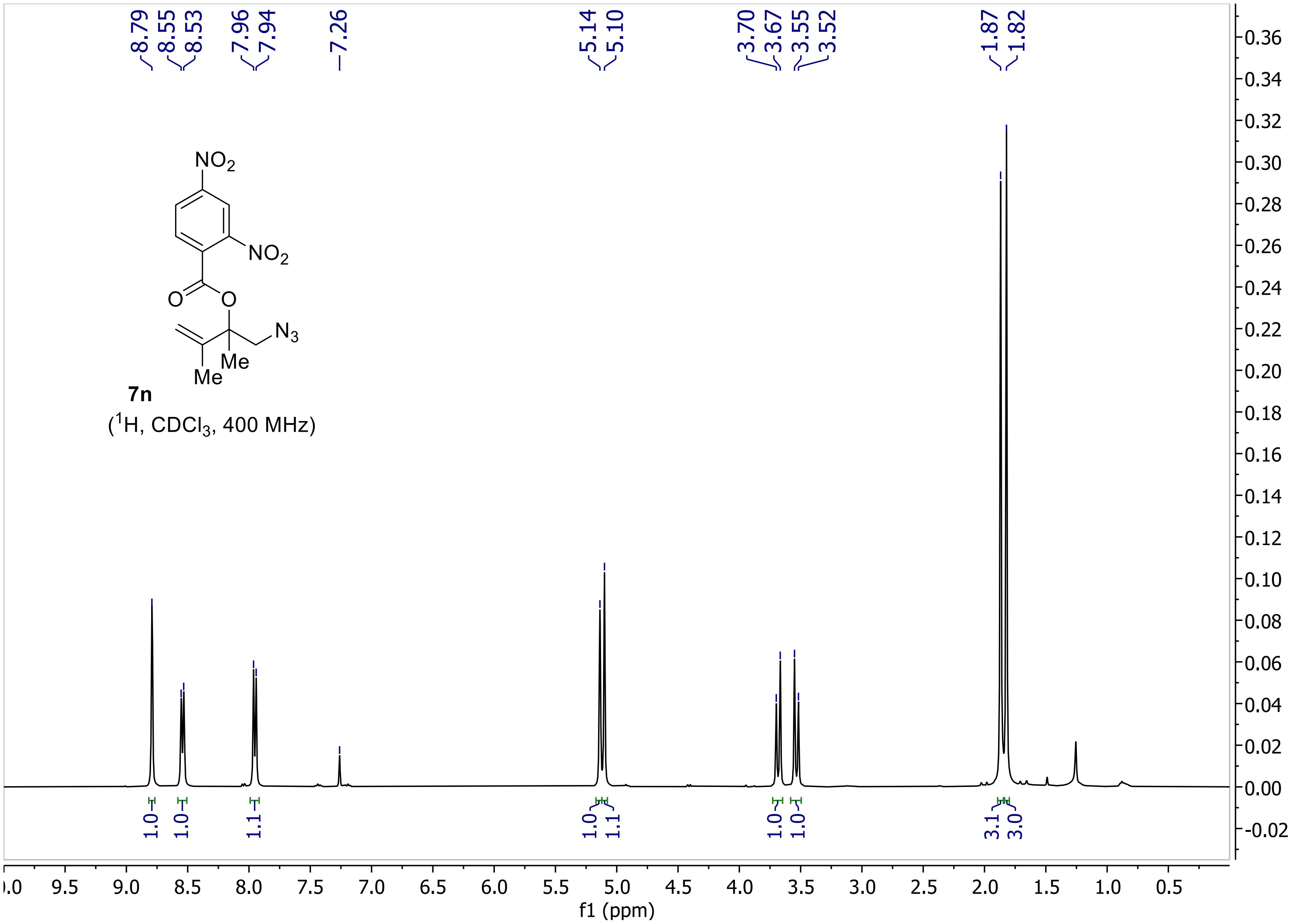

8.79
8.55
8.53
7.96
7.94
7.26
5.14
5.10
3.70
3.67
3.55
3.52
1.87
1.82
NO2
NO2
O
O
N3
Me
Me
7n
(1H, CDCl3, 400 MHz)
1.0
1.0
1.1
1.0
1.1
1.0
1.0
3.1
3.0
f1 (ppm)

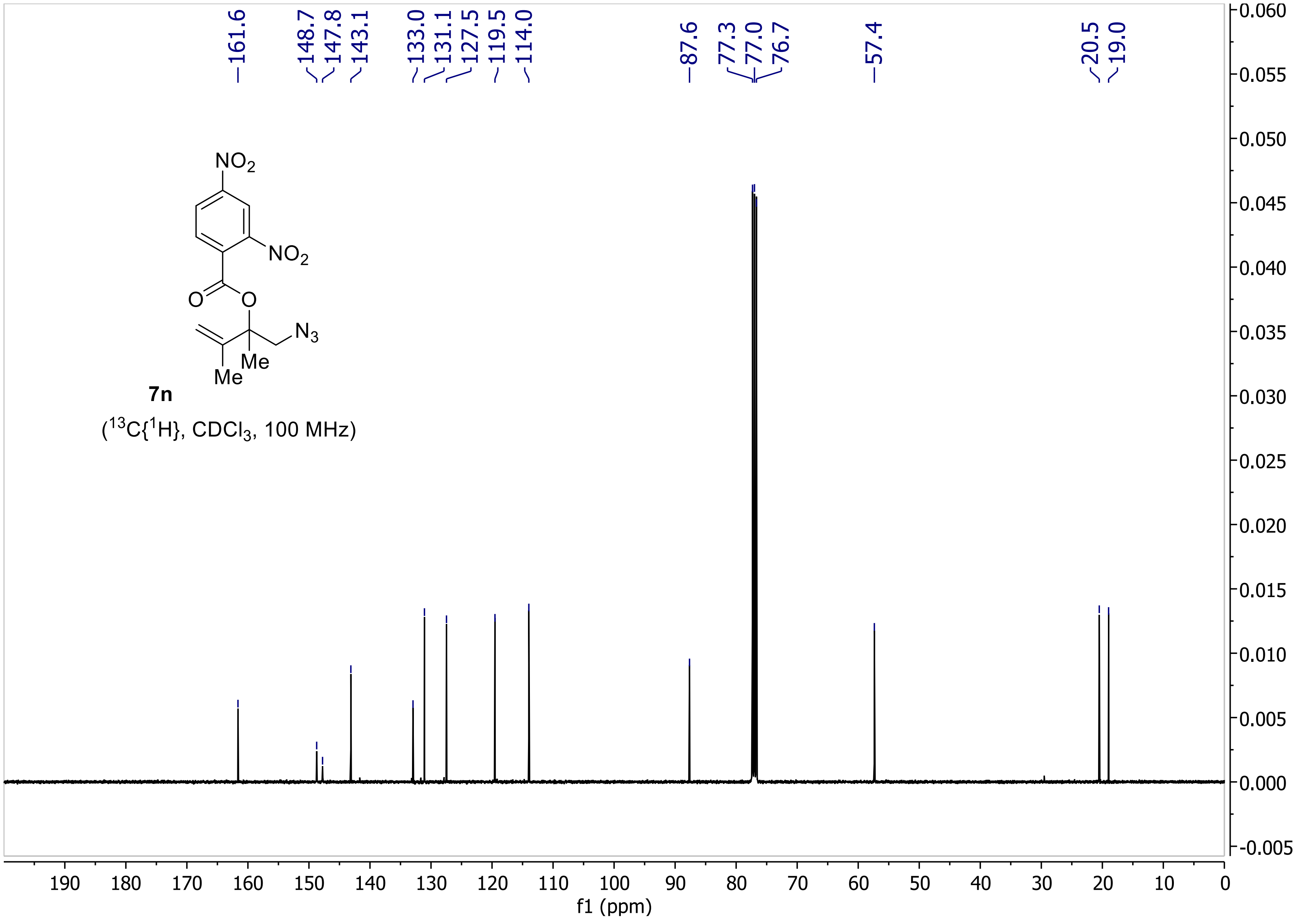

161.6
148.7
147.8
143.1
133.0
131.1
127.5
119.5
114.0
87.6
77.3
77.0
76.7
57.4
20.5
19.0
NO2
NO2
O
O
N3
Me
Me
7n
(13C{1H}, CDCl3, 100 MHz)
f1 (ppm)

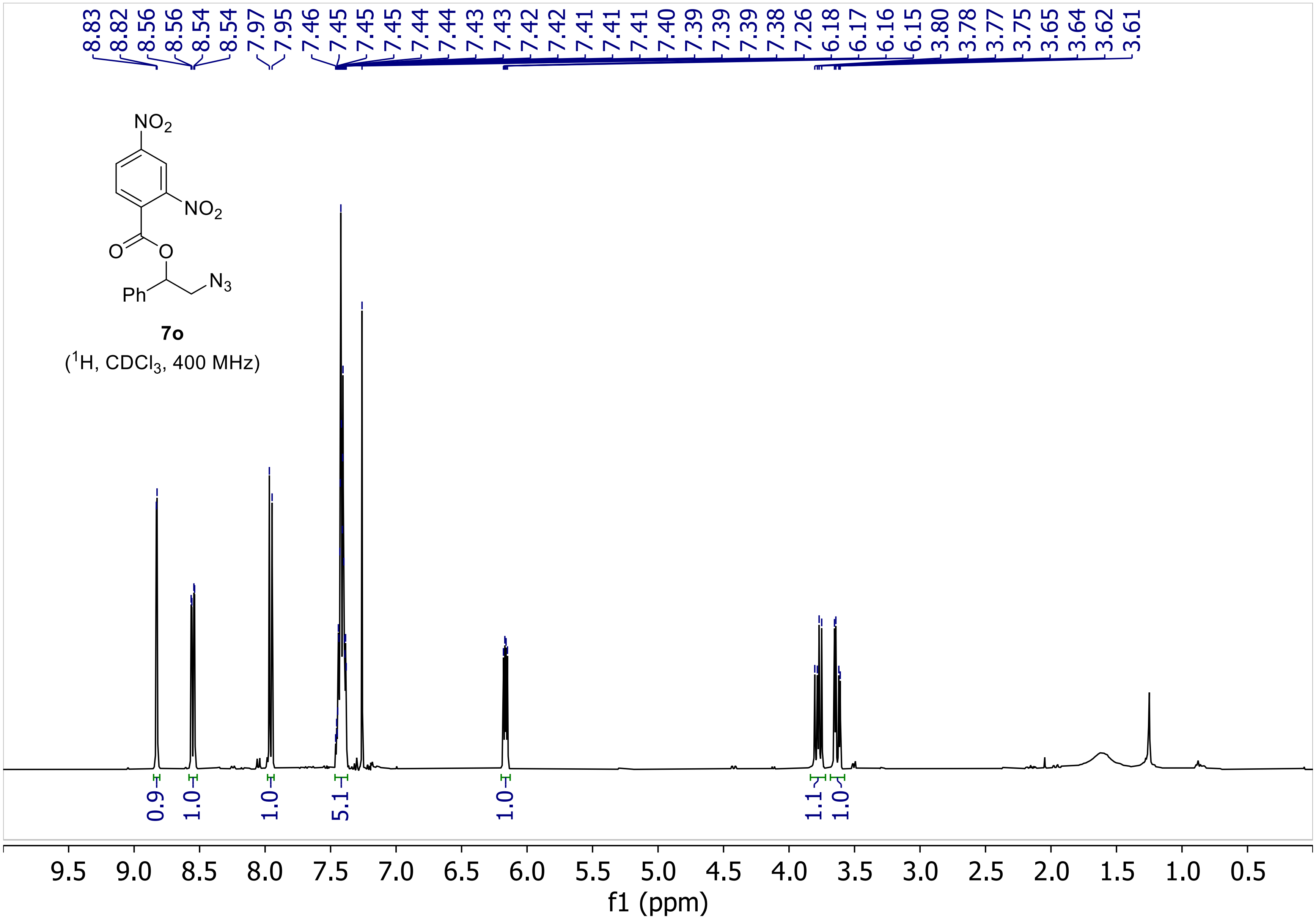
8.83
8.82
8.56
8.56
8.54
8.54
7.97
7.95
7.46
7.45
7.45
7.45
7.44
7.44
7.43
7.43
7.42
7.42
7.41
7.41
7.41
7.40
7.39
7.39
7.39
7.38
7.26
6.18
6.17
6.16
6.15
3.80
3.78
3.77
3.75
3.65
3.64
3.62
3.61
NO2
NO2
O
O
Ph
N3
7o
(1H, CDCl3, 400 MHz)
0.9
1.0
1.0
5.1
1.0
1.1
1.0
9.5
9.0
8.5
8.0
7.5
7.0
6.5
6.0
5.5
5.0
4.5
4.0
3.5
3.0
2.5
2.0
1.5
1.0
0.5
f1 (ppm)

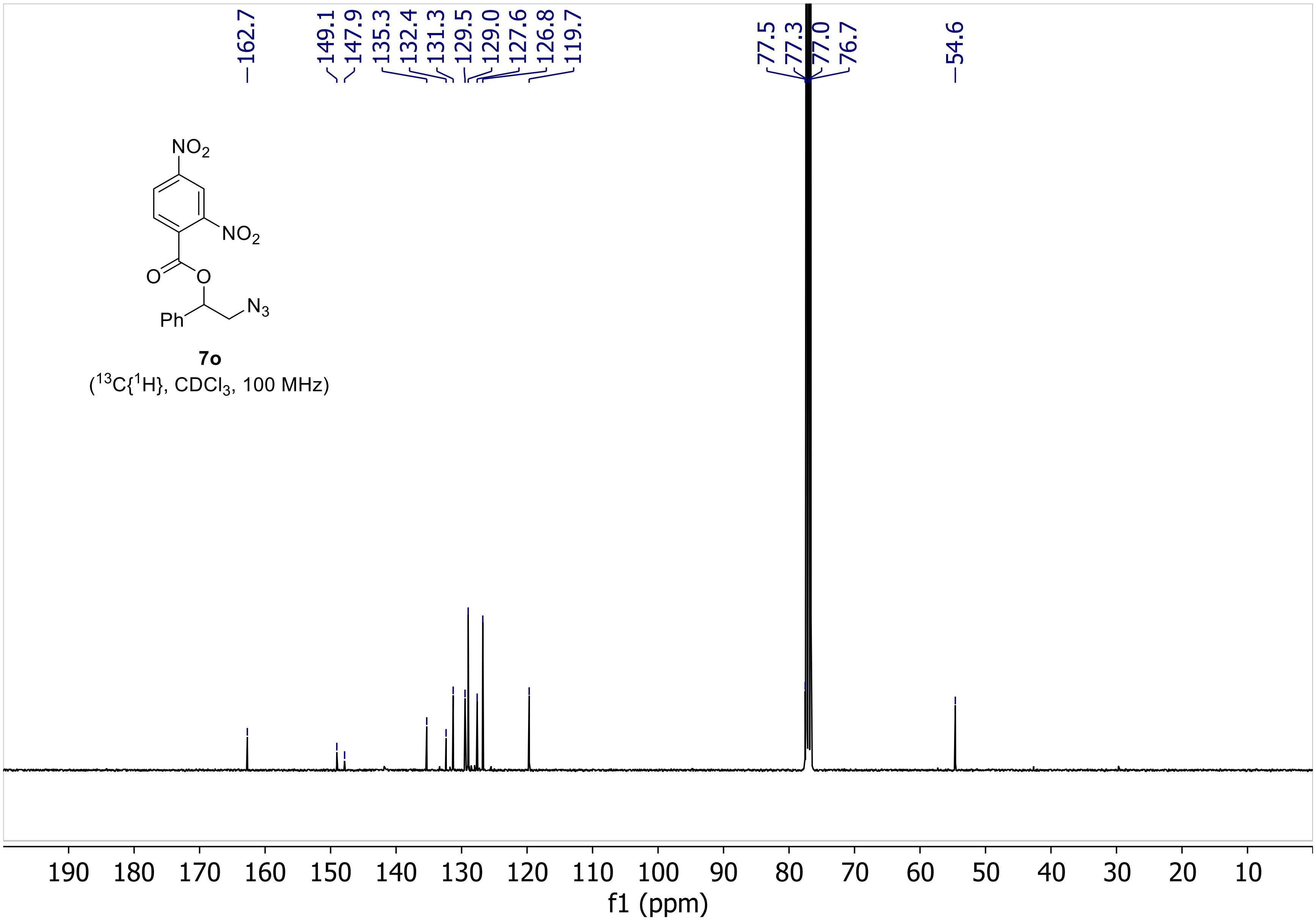

7o

($^{13}C\{^{1}H\}$, $CDCl_3$, 100 MHz)

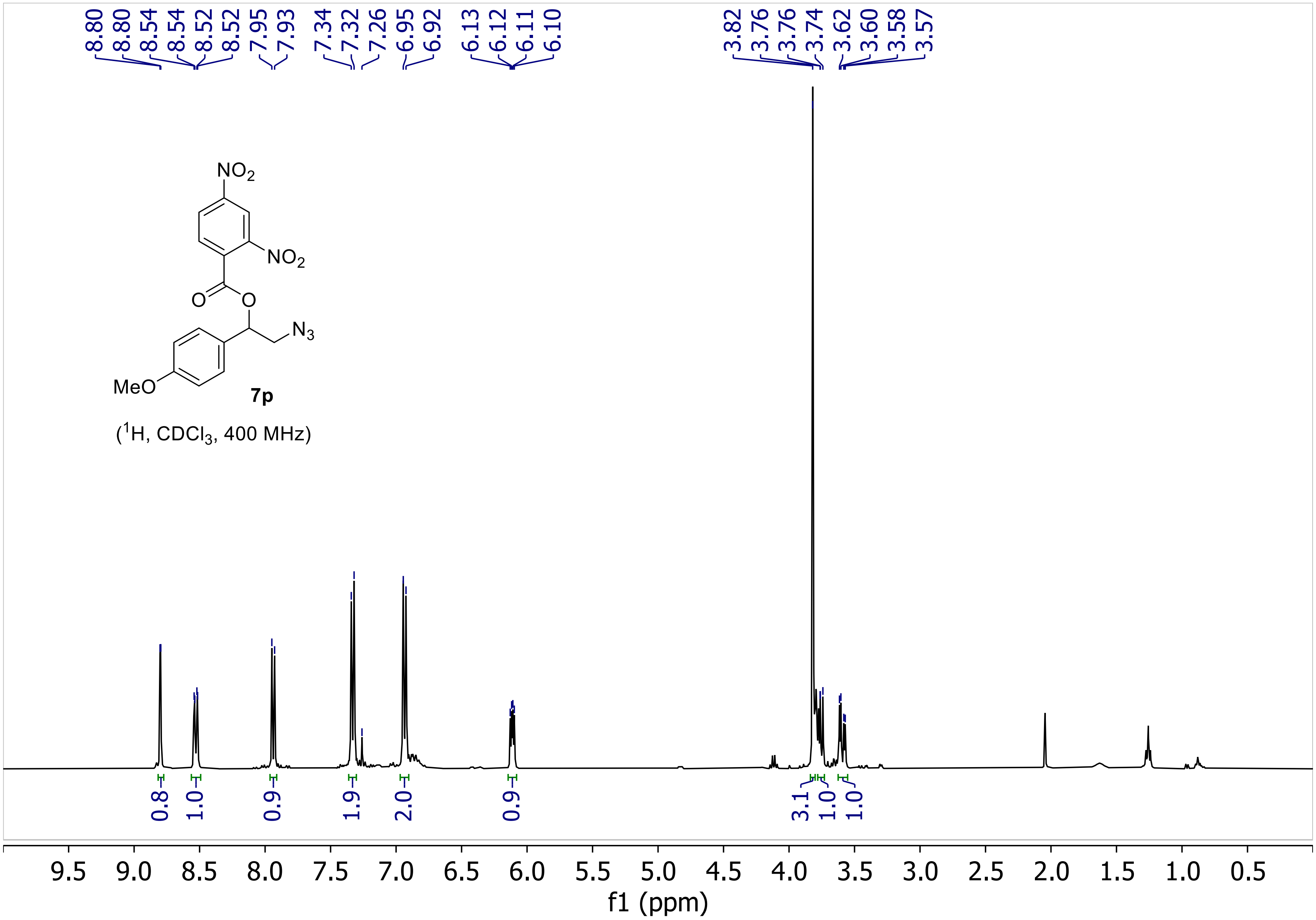
8.80
8.80
8.54
8.54
8.52
8.52
7.95
7.93
7.34
7.32
7.26
6.95
6.92
6.13
6.12
6.11
6.10
3.82
3.76
3.76
3.74
3.62
3.60
3.58
3.57
NO2
NO2
O
O
N3
MeO
7p
(1H, CDCl3, 400 MHz)
0.8
1.0
0.9
1.9
2.0
0.9
3.1
1.0
1.0
9.5
9.0
8.5
8.0
7.5
7.0
6.5
6.0
5.5
5.0
4.5
4.0
3.5
3.0
2.5
2.0
1.5
1.0
0.5
f1 (ppm)

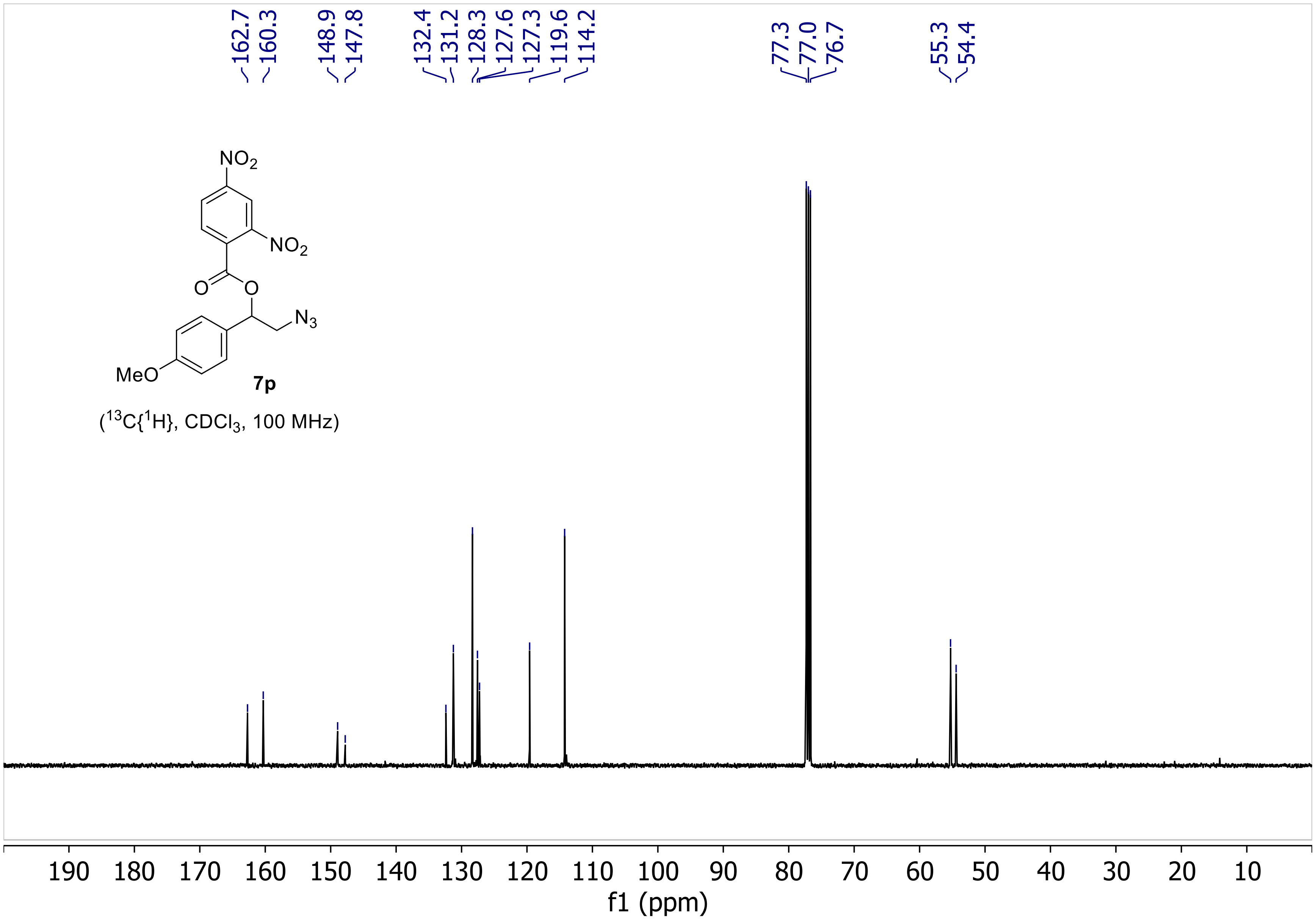
162.7
160.3
148.9
147.8
132.4
131.2
128.3
127.6
127.3
119.6
114.2
77.3
77.0
76.7
55.3
54.4
NO2
NO2
O
O
N3
MeO
7p
(13C{1H}, CDCl3, 100 MHz)
190 180 170 160 150 140 130 120 110 100 90 80 70 60 50 40 30 20 10
f1 (ppm)
